# Functional metagenomics reveals differential chitin degradation and utilization features across free-living and host-associated marine microbiomes

**DOI:** 10.1186/s40168-020-00970-2

**Published:** 2021-02-14

**Authors:** I. Raimundo, R. Silva, L. Meunier, S. M. Valente, A. Lago-Lestón, T. Keller-Costa, R. Costa

**Affiliations:** 1 Instituto de Bioengenharia e Biociências, Instituto Superior Técnico (IST), Universidade de Lisboa, Av. Rovisco Pais 1, Torre Sul, Piso 11, 11.6.11b, 1049-001 Lisbon, Portugal; 2 Laboratory of Aquatic Systems Ecology, Université Libre de Bruxelles, Brussels, Belgium; 3 Department of Medical Innovation, Centro de Investigación Científica y de Educación Superior de Ensenada (CICESE), 22860 Ensenada, Mexico; 4 Centro de Ciências do Mar (CCMAR), Universidade do Algarve, 8005-139 Faro, Portugal; 5 Department of Energy, Joint Genome Institute, Berkeley, CA 94720 USA; 6 Lawrence Berkeley National Laboratory, Berkeley, CA 94720 USA

**Keywords:** Chitinases, Chitosan, Metagenomics, Nitrogen cycling, Carbon cycling, Marine sponges, Octocorals, Host-microbe interactions

## Abstract

**Background:**

Chitin ranks as the most abundant polysaccharide in the oceans yet knowledge of shifts in structure and diversity of chitin-degrading communities across marine niches is scarce. Here, we integrate cultivation-dependent and -independent approaches to shed light on the chitin processing potential within the microbiomes of marine sponges, octocorals, sediments, and seawater.

**Results:**

We found that cultivatable host-associated bacteria in the genera *Aquimarina*, *Enterovibrio*, *Microbulbifer*, *Pseudoalteromonas*, *Shewanella*, and *Vibrio* were able to degrade colloidal chitin in vitro. Congruent with enzymatic activity bioassays, genome-wide inspection of cultivated symbionts revealed that *Vibrio* and *Aquimarina* species, particularly, possess several endo- and exo-chitinase-encoding genes underlying their ability to cleave the large chitin polymer into oligomers and dimers. Conversely, *Alphaproteobacteria* species were found to specialize in the utilization of the chitin monomer N-acetylglucosamine more often. Phylogenetic assessments uncovered a high degree of within-genome diversification of multiple, full-length endo-chitinase genes for *Aquimarina* and *Vibrio* strains, suggestive of a versatile chitin catabolism aptitude. We then analyzed the abundance distributions of chitin metabolism-related genes across 30 Illumina-sequenced microbial metagenomes and found that the endosymbiotic consortium of *Spongia officinalis* is enriched in polysaccharide deacetylases, suggesting the ability of the marine sponge microbiome to convert chitin into its deacetylated—and biotechnologically versatile—form chitosan. Instead, the abundance of endo-chitinase and chitin-binding protein-encoding genes in healthy octocorals leveled up with those from the surrounding environment but was found to be depleted in necrotic octocoral tissue. Using cultivation-independent, taxonomic assignments of endo-chitinase encoding genes, we unveiled previously unsuspected richness and divergent structures of chitinolytic communities across host-associated and free-living biotopes, revealing putative roles for uncultivated *Gammaproteobacteria* and *Chloroflexi* symbionts in chitin processing within sessile marine invertebrates.

**Conclusions:**

Our findings suggest that differential chitin degradation pathways, utilization, and turnover dictate the processing of chitin across marine micro-niches and support the hypothesis that inter-species cross-feeding could facilitate the co-existence of chitin utilizers within marine invertebrate microbiomes. We further identified chitin metabolism functions which may serve as indicators of microbiome integrity/dysbiosis in corals and reveal putative novel chitinolytic enzymes in the genus *Aquimarina* that may find applications in the blue biotechnology sector.

**Video abstract**

**Supplementary Information:**

The online version contains supplementary material available at 10.1186/s40168-020-00970-2.

## Background

Chitin, the polymer of (1 → 4)-β-linked N-acetylglucosamine (GlcNAc), is the most abundant polysaccharide in the marine environment [1]. Chitin does not accumulate in marine habitats as it is hydrolyzed by microorganisms that can use it as a carbon, nitrogen, and/or energy source [2]. This process is often mediated by chitinolytic enzymes, named chitinases, that hydrolyze the β-1,4 glycosidic bonds between the GlcNAc residues, producing chito-oligosaccharides (COSs). There are two types of chitinases: endo-chitinases (EC 3.2.1.14) that cleave chitin randomly at internal sites, generating diverse oligomers of GlcNAc such as chitotriose and chitotetraose; and exo-chitinases (EC 3.2.1.52) that can be further divided into two subtypes: chitobiosidases, which catalyze the progressive release of chitobiose, starting at the non-reducing end of the chitin microfibril; and N-acetyl-β-glucosaminidases or chitobiases, which cleave the oligomeric products of endo-chitinases and chitobiosidases, generating monomers of GlcNAc [3]. Endo-chitinases are classified into two glycoside hydrolase families, GH18 and GH19, based on amino acid sequence homology ([4] and refs. therein). They are commonly extracellular enzymes while the exo-chitinase N-acetyl-β-glucosaminidase frequently acts inside the bacterial cell [2]. The chitin derivative chitosan, formed via deacetylation, can be as well partially hydrolyzed by endo-chitinases if acetylated units remain in the polymer. Both endo- and exo-chitinases and their products have properties that bear promise for the development of new appliances in the food, medical, and agricultural sectors (for an overview see refs. [5–12]).

Despite our awareness of the relevance of chitin degradation to biogeochemical cycling across marine [2], freshwater [2, 13], and land [14, 15] ecosystems, current understanding of the abundance, diversity, and composition of chitin-degrading microorganisms across distinct biotopes is scarce. For marine biomes, particularly, we lack accurate documentation of how chitinolytic microbial communities—and prevailing chitin degradation pathways—may shift across environmental gradients and host-associated versus free-living settings, limiting our ability to envision and model patterns of nitrogen and carbon cycling in the oceans.

Given their remarkable filter- and detritus-feeding activities and complex microbiomes, it is tempting to hypothesize that sessile marine invertebrates host-microbial symbionts which either degrade chitin or utilize its degradation products. Indeed, detectable levels of exo-chitinase activity were found in crude extracts of the octocoral *Gorgonia ventalina* [16]. Further, Yoshioka and colleagues identified two chitinase-like genes in the genome of the scleractinian coral *Acropora digitifera* and reported chitinolytic activity in seven coral species [17]*.* Therefore, chitinases may be widely distributed in the coral holobiont and could play a role in the animals’ immune response against fungal infections as suggested elsewhere [18]. *Streptomyces* sp. strain DA11, retrieved from the marine sponge *Craniella australiensis*, was found to produce antifungal chitinases [19]. Recently, chitinase-encoding genes have been identified in *Aquimarina* strains from marine sponges, corals, sediments, and seawater [20, 21]. In addition, it is known that the remarkable chitin-degradation capacity of well-studied taxa such as *Vibrio* species is a key factor underlying their global patterns of distribution in the oceans [22] and, eventually, a generalist behavior across free-living and host-associated habitats. Taken together, these trends support the contention that the microbiomes of sessile marine invertebrates may contribute to ecosystem functioning by serving as natural settings for chitin/COSs degradation. However, this hypothesis remains largely underexplored despite the importance of chitin breakdown for carbon and nitrogen fluxes in the marine realm.

In this study, we integrate cultivation-dependent and -independent analyses to shed light on the potential degradation and utilization of chitin and its derivatives by the microbiomes of marine sponges, octocorals, sediments, and seawater (hereafter designated “biotopes”) and to determine whether chitin degrading assemblages within these microbiomes are taxonomically and metabolically distinct.

## Results

### Chitin degradation assays

Of the 41 marine sponge and octocoral bacterial associates tested in this study, 24 were found to degrade colloidal chitin on agar plates (Table 1). Among these, 12 were isolated from marine sponges and 12 from octocorals, and high reproducibility was recorded among replicates (*n* = 8 per strain). Results were highly dependent on bacterial taxonomy instead of host origin. All *Aquimarina* strains (*n* = 6) (phylum *Bacteroidetes*, class *Flavobacteria*), and nearly all strains in the class *Gammaproteobacteria*—including all *Enterovibrio* (*n* = 2) and *Vibrio* (*n* = 13) strains—were able to degrade colloidal chitin regardless of their origin (Table 1). In contrast, none of the *Alphaproteobacteria* strains, encompassing eight formally described genera and three unclassified *Rhodobacteraceae* spp., as well as *Micrococcus* sp. Mc110 (*Actinobacteria*), showed any chitin-degrading activity on the agar plates.

**Table 1:** Chitin and chitin-derivative degradation capacities of marine sponge and octocoral-derived bacterial isolates

Strain	Identity	Isolation source^**a**^	Chitin degradation^**b**^	Chitinase activity (units/mL)^**c**^			***chiA*** PCR^**d**^	Genome sequence^**e**^
				EndoS3	ExoS1	ExoS2		
**Mc110**	*Micrococcus* sp.	*Sarcotragus spinosulus*	**−**	**−**	**−**	**−**	**−**	**No**
**EL33**	*Aquimarina* sp.	*Eunicella labiata*	**++**	**+**	**++**	**−**	**+**	FLRG00000000.1
**EL43**	*Aquimarina* sp.	*Eunicella labiata*	**++**	**+**	**++**	**−**	**+**	**No**
**Aq349**	*Aquimarina* sp.	*Sarcotragus spinosulus*	**++**	**+**	**+**	**−**	**+**	OMKB00000000.1
**Aq78**	*Aquimarina* sp.	*Sarcotragus spinosulus*	**++**	**+**	**+**	**−**	**+**	OMKA00000000.1
**Aq135**	*Aquimarina* sp.	*Ircinia variabilis*	**+**	**+**	**−**	**+**	**+**	OMKE00000000.1
**Aq107**	*Aquimarina* sp.	*Sarcotragus spinosulus*	**+**	**−**	**−**	**−**	**+**	OMKC00000000.1
**EL57**	*Aliivibrio* sp.	*Eunicella labiata*	**−**	**+**	**+**	**−**	**+**	**No**
**EL58**	*Aliivibrio* sp.	*Eunicella labiata*	**−**	**++**	**+**	**−**	**−**	OMPC00000000.1
**EL24**	*Enterovibrio* sp.	*Eunicella labiata*	**++**	**+**	**+**	**+**	**+**	**No**
**EL37**	*Enterovibrio* sp*.*	*Eunicella labiata*	**++**	**+**	**+**	**+**	**+**	**No**
**EL22**	*Vibrio* sp.	*Eunicella labiata*	**++**	**++**	**+**	**+**	**+**	**No**
**EL36**	*Vibrio* sp.	*Eunicella labiata*	**+**	**+**	**+**	**+**	**+**	**No**
**EL38**	*Vibrio* sp.	*Eunicella labiata*	**++**	**++**	**+**	**+**	**+**	**No**
**EL41**	*Vibrio* sp.	*Eunicella labiata*	**++**	**+**	**+**	**+**	**−**	**No**
**EL49**	*Vibrio* sp.	*Eunicella labiata*	**++**	**+**	**+**	**+**	**+**	**No**
**EL62**	*Vibrio* sp.	*Eunicella labiata*	**+**	**+**	**+**	**+**	**+**	**No**
**EL67**	*Vibrio* sp.	*Eunicella labiata*	**+**	**++**	**+**	**+**	**+**	**No**
**EL112**	*Vibrio* sp.	*Eunicella labiata*	**++**	**+**	**+**	**+**	**+**	**No**
**Vb255**	*Vibrio* sp.	*Sarcotragus spinosulus*	**++**	**+**	**++**	**+**	**+**	**Yes**^**e**^
**Vb258**	*Vibrio* sp.	*Sarcotragus spinosulus*	**++**	**+**	**++**	**+**	**+**	**Yes**^**f**^
**Vb341**	*Vibrio* sp.	*Sarcotragus spinosulus*	**+**	**+**	**+**	**+**	**-**	**No**
**Vb339**	*Vibrio* sp.	*Sarcotragus spinosulus*	**+**	**+**	**+**	**+**	**+**	GCA_902751245.1
**Vb278**	*Vibrio* sp.	*Sarcotragus spinosulus*	**++**	**++**	**+**	**+**	**+**	CVNE00000000.1
**Cw315**	*Colwellia* sp.	*Sarcotragus spinosulus*	**−**	**−**	**+**	**−**	**−**	**No**
**Pa284**	*Pseudoalteromonas* sp.	*Sarcotragus spinosulus*	**+**	**+**	**++**	**−**	**−**	**No**
**EL12**	*Shewanella* sp*.*	*Eunicella labiata*	**−**	**+**	**++**	**+**	**−**	**No**
**Sw66**	*Shewanella* sp.	*Sarcotragus spinosulus*	**+**	**+**	**+**	**++**	**−**	**No**
**Mb45**	*Microbulbifer* sp.	*Sarcotragus spinosulus*	**++**	**+**	**+**	**+**	**−**	**No**
**EL27**	*Pseudophaeobacter* sp.	*Eunicella labiata*	**−**	**−**	**−**	**−**	**−**	OMPQ00000000.1
**EL26**	*Roseovarius* sp.	*Eunicella labiata*	**−**	**−**	**−**	**−**	**−**	OUMZ00000000.1
**EL01**	*Ruegeria* sp.	*Eunicella labiata*	**−**	**−**	**−**	**−**	**−**	OMPS00000000.1
**Rg50**	*Ruegeria* sp.	*Sarcotragus spinosulus*	**−**	**−**	**+**	**−**	**−**	**No**
**EL44**	*Sulfitobacter* sp.	*Eunicella labiata*	**−**	**−**	**−**	**−**	**−**	OMPT00000000.1
**EL53**	uncl. *Rhodobacteraceae*	*Eunicella labiata*	**−**	**−**	**+**	**−**	**−**	OMPR00000000.1
**EL129**	uncl*. Rhodobacteraceae*	*Eunicella labiata*	**−**	**−**	**−**	**−**	**−**	ONZJ00000000.1
**Ph222**	uncl. *Rhodobacteraceae*	*Ircinia variabilis*	**−**	**−**	**−**	**−**	**−**	**No**
**EL143**	*Labrenzia alba*	*Eunicella labiata*	**−**	**+**	**+**	**−**	**−**	OGUZ00000000.1
**Pv125**	*Pseudovibrio* sp.	*Sarcotragus spinosulus*	**−**	**−**	**++**	**−**	**−**	**No**
**EL199**	*Kiloniella* sp.	*Eunicella labiata*	**−**	**−**	**+**	**+**	**−**	OMPU00000000.1
**EL138**	*Sphingorhabdus* sp.	*Eunicella labiata*	**−**	**−**	**−**	**−**	**−**	OGVD00000000.1

^a^*Eunicella labiata*: octocoral; *Sarcotragus spinosulus* and *Ircinia variabilis*: marine sponges

^b^Chitin degradation observed as halo formation on colloidal chitin-containing agar plates: **++** halo-radius ≥ 8 mm, **+** halo-radius ≥ 1 mm and < 8 mm

^c^Chitinase activity measured in Units/ml on bacterial culture supernatants (EndoS3 = endo-chitinase activity) or cell extracts (ExoS1 = exo-chitinase β-N-acetylglucosaminidase activity; ExoS2 = exo-chitinase chitobiosidase activity): **++** ≥ 1 U/mL, **+** ≥ 0.01 and < 1 U/mL

^d^PCR-based amplification of the *chiA* gene fragments. ^e^Accession numbers are provided for genome sequences available on NCBI

^e,f^Unpublished draft genomes

### Chitinase activity assays

Endo-chitinase (EC 3.2.1.14) activity and colloidal chitin degradation results were overall congruent (Table 1). Endo-chitinase activity was registered for 23 of the 24 strains that degraded chitin on agar plates, whereas no endo-chitinase activity was recorded for 13 of the 17 strains unable to degrade colloidal chitin on agar plates (Table 1). These few incongruencies likely result from eventual sub-optimal experimental conditions for specific strains. All *Vibrio* and *Enterovibrio* strains displayed both endo- and exo-chitinolytic activities and the capacity to utilize all three substrates used in the enzymatic bioassays (Table 1). Endo- and exo-chitinolytic activity was also recorded for most *Aquimarina* strains (Table 1). N-acetylglucosaminidase activity was documented for five of the 12 *Alphaproteobacteria* strains tested, encompassing members of the genera *Ruegeria*, *Pseudovibrio*, *Labrenzia*, and *Kiloniella* (Table 1). PCR amplification of *chiA* gene fragments—targeting “group A” within glycosyl hydrolase family 18 (GH18) endo-chitinases (see the “Methods” section) was considered a good indicator of endo-chitinolytic activity by *Vibrionaceae* and *Aquimarina* strains (Table 1). However, no *chiA* gene amplicons could be retrieved for gammaproteobacterial genera out of the *Vibrionaceae* family, namely, chitin-degrading strains *Shewanella* Sw66, *Microbulbifer* Mb45, and *Pseudoalteromonas* Pa284 (Table 1).

### Chitin metabolism encoded on the genomes of bacterial associates of marine sponges and octocorals

**Fig. 1:**
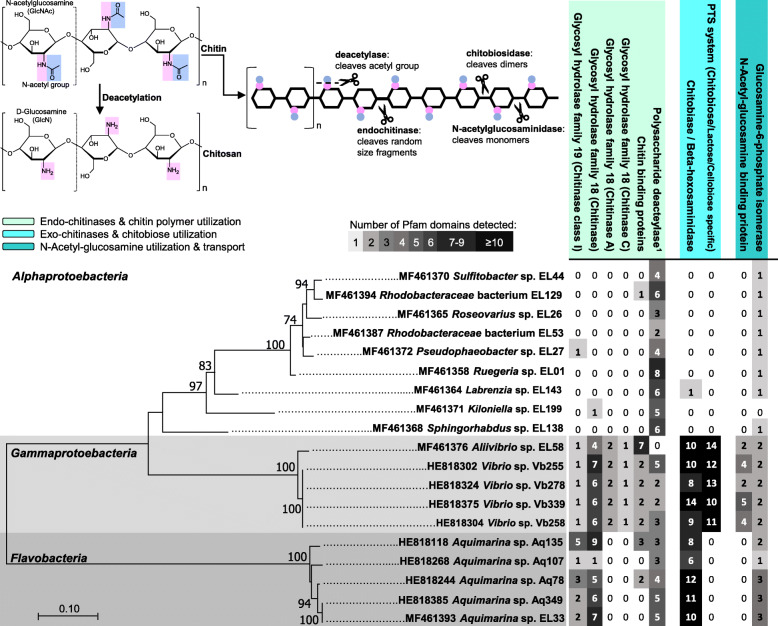
Annotation of chitin and chitin-derivative degradation and utilization genes in cultivated bacterial symbionts of sponges and octocorals. Nineteen genomes available from the panel of 41 strains examined in this study are portrayed, spanning ten formally described and two potentially novel genera across three bacterial classes. The phylogenetic tree on the bottom left is based on a maximum likelihood analysis (Generalized Time Reversible model) of the 16S rRNA gene (16S rRNA gene accession numbers are given next to each strain name). Numbers at nodes represent values based on 300 bootstrap replicates. The table on the right shows Pfam-based annotations for predicted protein domains involved in hydrolysis (endo-chitinases of GH families 18 and 19—EC 3.2.1.14, chitin-binding proteins) and deacetylation (polysaccharide deacetylases) of the of the large chitin polymer (light green panel), hydrolysis (exo-chitinases, EC 3.2.1.52) of chitin non-reducing ends and chitobiose transport (light blue panel), and N-acetylglucosamine binding and utilization (blue panel); the latter function represented by the presence of glucosamine 6-phosphate isomerases (EC 3.5.99.6). These functional categories have been as well used to estimate the relative abundance of CDSs involved in the breakdown and utilization of chitin and chitin derivatives across the sponge and octocoral metagenome datasets (Fig. 3). Values in each cell of the table correspond to the number of Pfam domains detected for each functional category in each genome, whereby higher numbers are highlighted in dark-gray shading. The upper left panel shows the molecular structure of chitin and chitosan. It highlights the N-acetyl group characteristic of the chitin polymer, which is cleaved by deacetylases in the process of chitosan formation, and the cleaving sites of endo- and exo-chitinases along the chitin chain. Superscript “1” corresponds to InterPro database entry IPR002509 (see also Fig. 3) which describes the metal-dependent deacetylation of O- and N-acetylated polysaccharides such as chitin, peptidoglycan, and acetylxylan

**Fig. 2:**
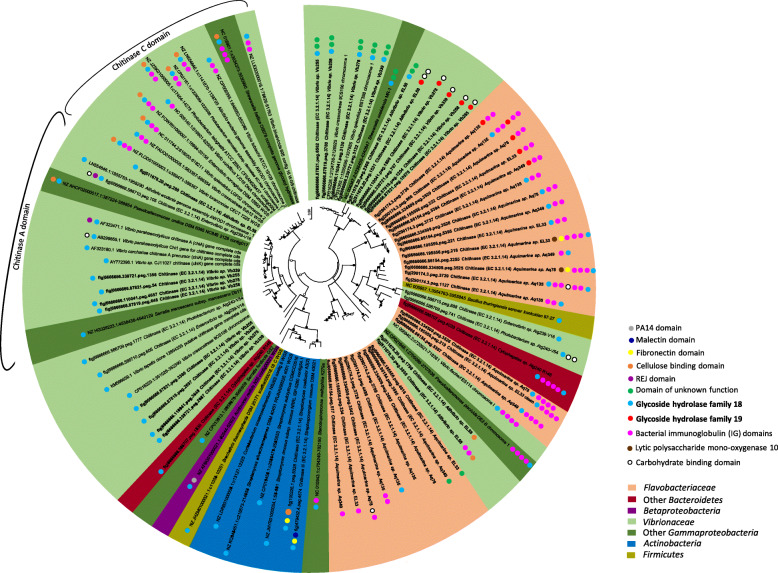
Phylogeny of full-length endo-chitinase encoding genes from cultivated bacteria. The evolutionary history was inferred using the Maximum Likelihood method based on the Generalized Time Reversible model (GTR). The tree with the highest log likelihood (−69520.19) is shown. A discrete Gamma distribution was used to model evolutionary rate differences among sites (5 categories (+G, parameter = 5.2345)). The tree is drawn to scale, with branch lengths measured in the number of nucleotide substitutions per site. The percentage (≥ 70%) of trees in which the associated taxa clustered together is shown next to the tree nodes (1000 bootstrap repetitions), with solid and open circles representing ≥ 90% and between 70 and 89% bootstrap support, respectively. Codon positions included were 1st + 2nd + 3rd + noncoding, and a partial deletion model with 85% site coverage was employed in tree construction. The analysis involved 90 nucleotide sequences mostly retrieved from fully sequenced bacterial genomes (*n* = 85), including the 19 genomes examined in detail in this study (Fig. 1, Table 1), and span 16 bacterial genera across four phyla. There were a total of 1189 positions in the final dataset. Coding sequences containing GH18 (*n* = 69) and/or GH19 (*n* = 10) domains (endo-chitinases—EC 3.2.1.14), as revealed by Pfam annotations, are highlighted with blue and red dots next to tree labels, respectively. Eleven further sequences have been included for which neither GH18 nor GH19 domains could be identified using Pfam-based annotations, but which showed significant levels of homology with endo-chitinase sequences present in NCBI’s protein database. Other domains annotated within the analyzed coding sequences were as well labeled with colored dots and are identified on the right panel next to the tree. Trans-membrane and signal peptide domains could be annotated for nearly all analyzed coding sequences. For further details on the distribution of protein domains across all sequences and the closest phylogenetic relative to each sequence query, see Additional file 1: Table S4

In general agreement with phenotypic assays, while the predicted proteomes (i.e., Pfam annotations) of *Vibrio*, *Aliivibrio*, and *Aquimarina* species all possessed several endo- (EC 3.2.1.14) and exo-chitinase (EC 3.2.1.52) catalytic domains, those of *Alphaproteobacteria* species usually lacked them (Fig. 1). Noteworthy was the high number of endo-chitinases of the GH18 family predicted for the three abovementioned genera, with *Vibrio* and *Aliivibrio* strains possessing types A and C GH18 chitinases, not verified among *Aquimarina* strains. Chitinases of the GH19 family were likewise found for all strains from these three genera (see also Fig. 2). *Vibrio* and *Aliivibrio* species possessed the most versatile genetic machinery for the utilization of chitin and its derivatives according to Pfam-based annotations. Both genera displayed genes encoding diverse protein domains required for chitin and chitobiose cleavage as well as N-acetylglucosamine utilization (Fig. 1), including numerous chitobiose-specific transport systems and N-acetylglucosamine binding proteins not documented for *Aquimarina*. Interestingly, potential for chitin deacetylation into chitosan could be inferred for all strains (except *Aliivibrio* sp. EL58) due to the presence of polysaccharide deacetylases in their predicted proteomes (Fig. 1). Likewise, using glucosamine-6-phosphate isomerase detection as proxy, Pfam annotations revealed the potential of all strains (except *Kiloniella* sp. EL199) to utilize N-acetyl glucosamine (Fig. 1). Overall, the type and number of protein domains involved in the metabolism of chitin and its derivatives were found to differ in a taxon-dependent manner. While deacetylation and GlcNAc utilization potential were traits common to all genomes, chitin degradation capacities (endo- and exo-) were pronounced features of *Vibrio*, *Aliivibrio*, and *Aquimarina* genomes (Fig. 1). Details on all Pfam entries employed to build Fig. 1 and their distributions across the examined genomes are provided as Supplementary information (Additional file 1: Table S1). In contrast with Pfam-based annotations, RAST annotations revealed potential exo-chitinase activity for three (instead of one) *Alphaproteobacteria* genomes, showing as well that *Alphaproteobacteria* spp. often carried genes involved in N-acetylglucosamine utilization and transport with specific GlcNAc ABC transporters (Additional file 2, Figure S1).

### Relative abundance of culturable chitin degraders in sponge and octocoral microbiomes

In agreement with previous studies [23–25], 16S rRNA gene-based estimates of relative abundance revealed that the culturable bacterial genera analyzed here correspond to a minor portion of the total microbial metagenome in sponges and octocorals (Additional file 1: Table S2). This seems particularly true for sponges where such estimates did not surpass the 0.02% threshold, while genera such as *Pseudoalteromonas*, (1.05%), *Shewanella* (0.75%), *Vibrio* (0.42%), and *Aquimarina* (0.34%), all possessing chitinolytic activity, did amount to much higher proportions in the healthy octocoral microbiome. Interestingly, several cultivated taxa displayed increased abundances in necrotic versus healthy octocoral tissue (Additional file 1: Table S2), of which we highlight *Aquimarina* (20-fold increase), *Vibrio* (2-fold), *Ruegeria* (6-fold), and unclassified *Rhodobacteraceae* (7-fold). These trends were corroborated by strain-specific estimates of relative abundance based on genome-metagenome mapping (Additional file 1: Table S3), carried out for all chitinolytic strains with sequenced genomes (Fig. 1). Indeed, both estimates of percent abundance and genome coverage suggest that cultured symbionts correspond to low abundance populations usually more abundant in the octocoral than in the sponge microbiome. Further, sharp increases in genome-wide estimates of abundance were observed for *Aquimarina* and diverse alphaproteobacterial strains in necrotic versus healthy octocoral tissue (Additional file 1: Table S3).

### Phylogenetic analysis of endo-chitinase-encoding genes

We assessed the phylogenetic diversification of genes encoding for GH18 and GH19 endo-chitinases (EC 3.2.1.14) spanning at least 16 bacterial genera across four phyla, with emphasis on coding sequences identified in the genomes investigated in this study and their closest relatives (Fig. 2, Additional file 1: Table S4).

The obtained tree topology is consistent with the known heterogeneity of endo-chitinase genes, which contain regions encoding for multiple protein domains—e.g., chitin-, cellulose- and carbohydrate-binding, fibronectin III, and immunoglobulin domains—arranged in various modes of synteny [4]. Consequently, owing to the low sequence homology between the major clades in the tree, levels of phylogenetic relatedness inferred for such clades must be considered with much caution. Still, at a coarse level of phylogenetic resolution, these major clades (usually presenting bootstrap support > 90%) were found to split the tree into coherent taxonomic or functional categories, discriminating endo-chitinases of *Gammaproteobacteria* (dominated by *Vibrio* spp.), *Flavobacteriia* (dominated by *Aquimarina* spp.), and *Actinobacteria* origins reasonably well (Fig. 2). Furthermore, the affiliation of sequences within major clades was congruent with Pfam-based annotations regarding the detection of GH18 versus GH19 chitinase families, and of group A versus group C GH18 chitinases (in this case, among about half of the *Gammaproteobacteria* sequences). Noticeably, the extent of within-genome diversification of chitinase genes was high for strains of the genera *Aquimarina* and *Vibrio*, with coding sequences from the same genome often spread across distinct clades. This was the case for endo-chitinase gene distributions from, e.g., *Vibrio* sp. strains Vb255, Vb258, Vb278, and Vb339 and *Aquimarina* sp. strains Aq135, Aq78 and Aq349, and EL33. Conversely, highly homologous endo-chitinase encoding sequences from different strains or species of the same genus were as well consistently found across the tree. For example, *Aquimarina* strains EL33, Aq78, and Aq349, which likely represent different species within the genus based on phylogenomic assessments [21], shared endo-chitinase-encoding genes with > 95% sequence homology (including GH19 CDSs). The same pattern was observed for *Vibrio* sp. strains Vb339, Vb255, Vb258, and Vb278 (all closely related to the species *V. crassostreae*, see, e.g., [26]), which possess multiple phylogenetically close chitinase coding sequences (including GH19 CDSs). Likewise, *Streptomyces rimosus*, *S. autolyticus*, and *S. olivochromogenes* shared highly homologous chitinase-encoding genes (Fig. 2). Some examples of disparate taxa (i.e., belonging to different classes) forming well-supported clades incongruent with their expected (16S rRNA gene-based) phylogenies have been found. Pairwise levels of homology between sequences from these taxa were never close to 100%, preventing hypotheses to be made on recent horizontal gene transfer events underlying these patterns. This was the case of the chitinase-encoding genes from *Streptosporangium roseum* DSM 43021, *Streptomyces coelicolor* A3 (both *Actinobacteria*), and *Stenotrophomonas maltophilia* K279a (*Gammaproteobacteria*), which formed a solid cluster (99% bootstrap support) within the larger clade dominated by *Actinobacteria* strains (Fig. 2). There were eight coding sequences from *Aquimarina* strains for which no GH18 or GH19 domains could be annotated, although they shared resemblance with endo-chitinase sequences based on homology searches (Additional file 1: Table S4). These sequences were clustered together into the same phylogenetic clade, suggesting that additional diversity of endo-chitinase domains exist within *Aquimarina* spp. which escapes detection by current Pfam-based annotation.

### Relative abundance of chitin metabolism-encoding genes across marine biotopes

**Fig. 3:**
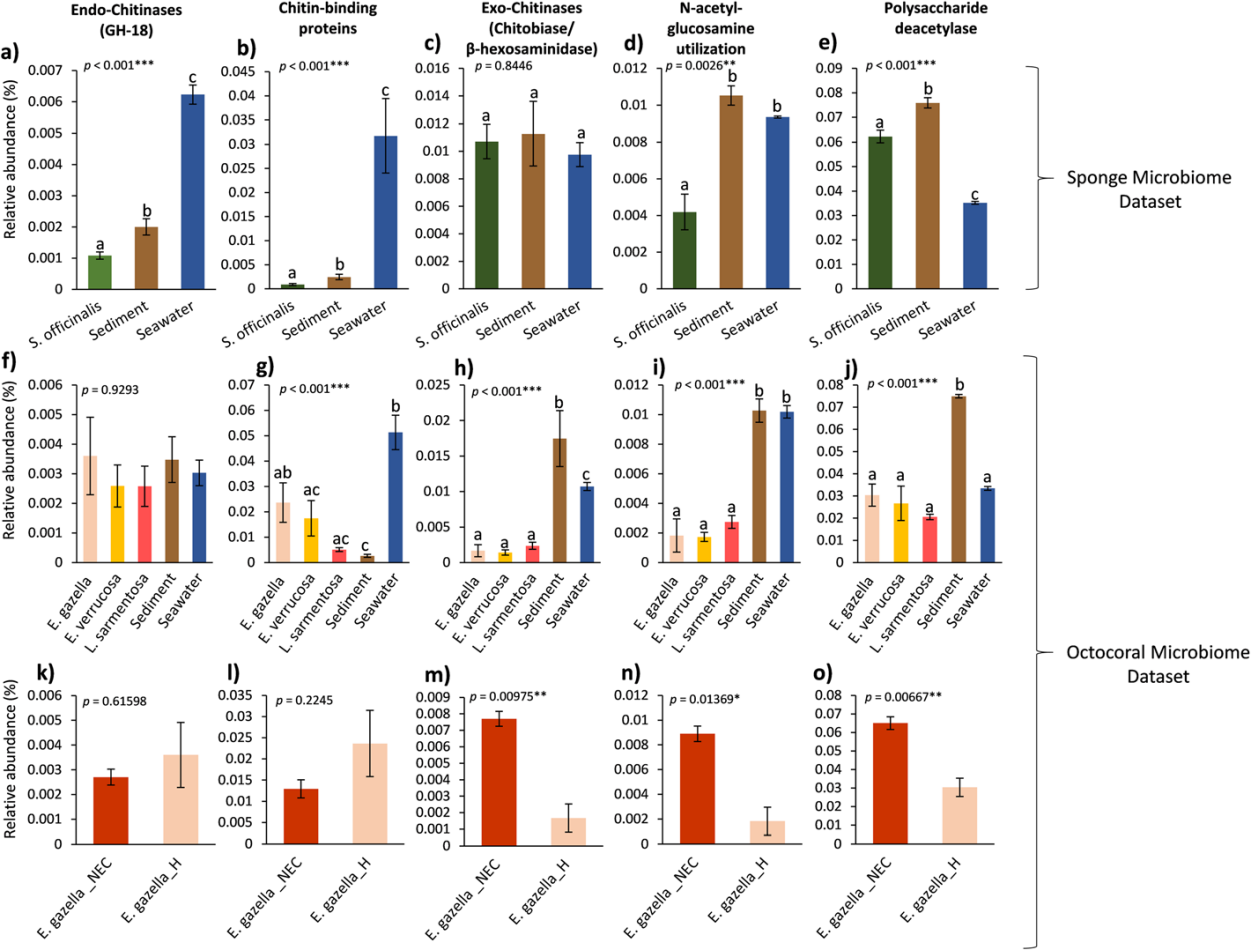
Relative abundance of chitin processing-encoding genes across marine biotopes. The analysis involved the screening of all microbial metagenomes for InterPro (IPR) database entries corresponding to the presence of GH18 endo-chitinase (**a**, **f**, **k**), chitin-binding protein (**b**, **g**, **l**), exo-chitinase (**c**, **h**, **m**), N-acetylglucosamine utilization (**d**, **i**, **n**), and polysaccharide deacetylase (**e**, **j**, **o**) coding sequences (CDSs) (see Additional file 1: Table S5 for details on the InterPro entries used here). The relative abundances (mean ± SE) of IPR entries pertaining to each functional category (calculated as “sum of all CDSs assigned to functional category x/total number CDSs with function”) are shown on *Y*-axes. One-way ANOVAs, followed by Tukey’s post hoc tests if significant (**a** to **j**) or *t* tests (**k** to **o**) were used to test for statistical differences between sample groups. Statistical analyses were performed after Hellinger transformation of the data (square root of relative abundances). Respective *p* values are given in each panel for general differences among groups and letters (**a** to **j**) or asterisks (**k** to **o**) above error bars indicate significant differences (*p* < 0.05). Panels (**a** to **e**) represent the sponge metagenome dataset (project ID PRJEB11585, [27] while panels (**f** to **o**) represent the octocoral metagenome dataset (project ID PRJEB13222, [28]. Panels (**f** to **j**) compare the healthy microbiome of octocoral species with those from their environmental vicinities, while panels (**k** to **o**) present relative abundances of chitin degradation-encoding genes in healthy versus necrotic tissue of *Eunicella gazella*

**Fig. 4:**
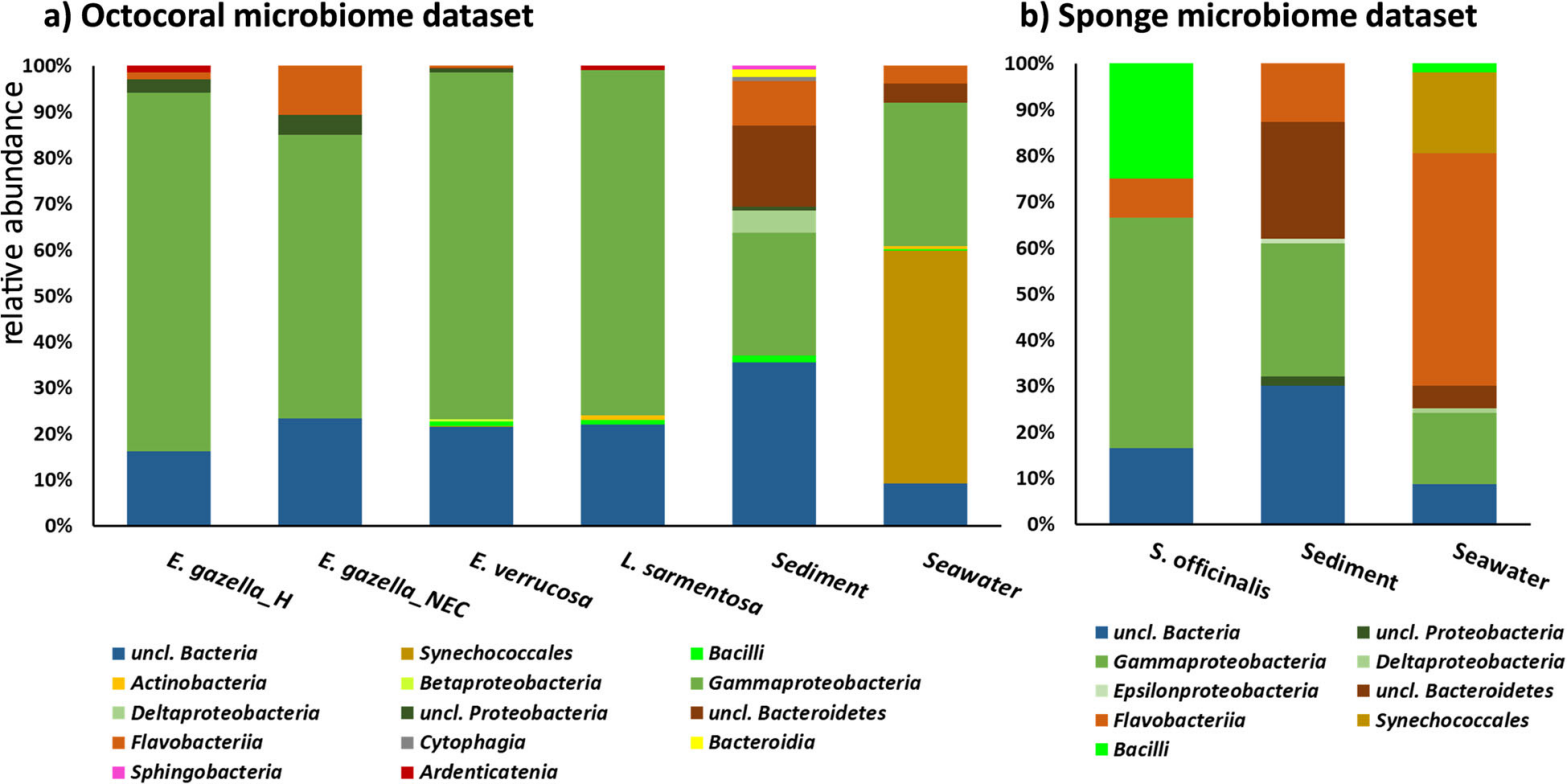
Class-level taxonomic composition of chitinolytic microbial communities across marine biotopes. Microbial metagenome mining and assignment of chitinase-encoding genes were performed on unassembled metagenome samples of seawater, marine sediments, octocorals, and marine sponges. Panels (**a** and **b)** represent the octocoral metagenome dataset (project PRJEB13222 [28] and the sponge metagenome dataset (project PRJEB11585 [27], respectively. Putative endo-chitinase nucleotide sequences were retrieved from both datasets using the MG-RAST metagenomics analysis platform [30] and then subjected to a stringent NCBI blastx search for taxonomic and functional assignments. Only those sequence reads which returned chitinases as closest hits and could be assigned to bacterial taxa after NCBI blastx procedures were used in taxonomic profiling. *E. gazella*_H—healthy *Eunicella gazella* tissue; *E. gazella_*NEC—necrotic *Eunicella gazella* tissue. For genus-level taxonomic profiling of sponge and octocoral samples, see Additional file S1: Table S7

We examined whether marine sponge and octocoral microbiomes host genes involved in chitin/COSs degradation and N-acetylglucosamine utilization in comparable proportions with those of the environmental surroundings. To this end, a “marine sponge metagenome” [27] and an “octocoral metagenome” [28] dataset was used to compare, in a cultivation-independent manner, the relative abundance of genomic features involved in breakdown and utilization of chitin and its derivatives (Fig. 3) and the taxonomic composition of endo-chitinase-encoding genes (Fig. 4) across host-associated and free-living biotopes.

For relative abundance analysis of chitin-breakdown related features (Fig. 3), we used InterPro-based annotations of unassembled metagenomic reads (Additional file 1: Table S5) retrieved with the MGnify v. 2.0 metagenomics pipeline from the European Bioinformatics Institute (EMBL-EBI) [29]. For details on the general features of all metagenome samples analyzed, including sequencing depth and number of CDSs with function per sample, see Additional file 2: Detailed methodology. The panel of functions portrayed in Fig. 3 encapsulates a range of IPR entries detectable across the samples (Additional file 1: Table S5) which collectively serve as proxies for hydrolysis and deacetylation of the chitin polymer, hydrolysis of chitin oligomers (COSs), and utilization of the chitin monomer N-acetylglucosamine, also explored in Fig. 1 to examine chitin metabolism traits among bacterial cultures.

While the relative abundance of endo-chitinase (EC 3.2.1.14) encoding genes was higher in sediments and seawater than in the *Spongia officinalis* endosymbiotic consortium (Fig. 3a), significant differences were neither observed when the microbiomes of three octocoral species were compared with those of the environmental surroundings (Fig. 3f), nor when the microbiomes of healthy and necrotic octocoral (*Eunicella gazella*) tissues were contrasted (Fig. 3k). When the relative abundances of chitin-binding protein (CBP) encoding genes were considered, we found that seawater microbiomes clearly presented significantly higher proportions than sediment, sponge, and octocoral microbiomes (Fig. 3b and g). The relative abundance of CBP encoding genes dropped considerably in the microbiome of necrotic *E. gazella* tissues in comparison with that of their healthy counterparts yet differences were deemed not statistically significant (Fig. 3l). As for exo-chitinase (EC 3.2.1.52) encoding genes, equivalent abundances were found between sponge-associated and free-living microbiomes (Fig. 3c), whereas higher relative abundances were registered for free-living microbiomes in comparison with octocoral microbiomes (Fig. 3h). Higher relative abundance of exo-chitinase encoding genes were also recorded in the microbiomes of necrotic in comparison with healthy *E. gazella* tissue (Fig. 3m). Abundance distributions of N-acetyl-glucosamine utilization genes (Fig. 3d, i, n) were highly congruent with those described for exo-chitinase genes, except for the *S. officinallis* microbiome where N-acetyl-glucosamine utilization genes displayed lower abundance than in seawater and sediments (Fig. 3d). Finally, the frequency distributions of polysaccharide deacetylases (which catalyze the formation of chitosan from chitin) in both the sponge and octocoral metagenome datasets (Fig. 3e, j, o) followed the trends observed for the exo-chitinase (EC 3.2.1.52) encoding genes. The relative abundances of polysaccharide deacetylases were overall higher in comparison with those of endo- or exo-chitinase encoding genes in all the biotopes surveyed.

### Taxonomic classification of endo-chitinase encoding genes from host-associated and free-living biotopes

Among all features involved in chitin metabolism, this study places focus on the heterogeneity and taxonomy of endo-chitinase encoding genes because of their historical use as indicators of potential chitin degradation across nature’s microbiomes [2]. To explore the taxonomic composition of chitin-degrading microbiomes across the biotopes studied here, we first fetched potential endo-chitinase encoding gene sequences (EC 3.2.1.14) from the samples using the MG-RAST analysis server [30, 31] with default parameters. The retrieved sequences were thereafter curated through stringent blastx procedures for the selection of reliable entries to be used in taxonomic profiling (Table 2; see Additional file 1: Table S6 to access each sequence read). The proportion reads identified by MG-RAST which returned chitinase-specific closest hits from bacteria after blastx searches on NCBI varied considerably across biotopes, ranging from 22.6% in sediment samples of the sponge metagenome dataset to 79.7% in the necrotic tissues of the octocoral *E. gazella* (Table 2). Taxonomic assignments portrayed here only consider sequence reads which returned chitinase-specific closest hits assigned to the domain Bacteria using NCBI Blastx (Fig. 4, Additional file 1, Table S7).

**Table 2:** Metagenomic reads classified as chitinase-encoding gene sequences with MG-RAST

Dataset	Sample category	# Reads MG-RAST^**a**^	# Blast^b^ (%)
Octocoral metagenome	*Eunicella gazella* (healthy)	94	68 (72.3%)
	*Eunicella gazella* (necrotized)	59	47 (79.7%)
	*Eunicella verrucosa*	1458	795 (54.5%)
	*Leptogorgia sarmentosa*	127	100 (79.4%)
	Sediments	401	124 (30.9%)
	Seawater	599	317 (52.9%)
Sponge metagenome	*Spongia officinalis*	48	12 (25.0%)
	Sediments	455	103 (22.6%)
	Seawater	257	103 (40.1%)

^a^Number of unassembled metagenomic reads classified by MG-RAST as endo-chitinase (EC 3.1.2.14) coding sequences

^b^Proportion of chitinase reads classified by MG-RAST which were assigned to the domain Bacteria and returned chitinase-specific closest hits after blastx searches on NCBI. Figure 4 shows the taxonomic affiliation of these reads in detail

For both the octocoral and sponge metagenome datasets, changes in taxonomic composition of chitinolytic communities across host-associated and free-living biotopes were already evident at the class level (Fig. 4), strongly supporting the hypothesis of divergent chitinolytic community structures in these settings. Within both datasets, sediments were found to host the highest number of bacterial classes, followed by seawater (Fig. 4a, b). All biotopes housed considerable proportions of bacterial endo-chitinase reads not classifiable at the phylum level (from *c.* 10% in seawater to remarkable 30% in sediments), warranting further bioprospection for chitinolytic activities/endo-chitinase diversity in these systems. Interestingly, while the seawater microbiome in the octocoral dataset (samples collected at 18 m depth close to the summer solstice, 2014) was dominated by *Synechococalles*-derived chitinases, in the sponge metagenome dataset (samples collected at 20 m depth in spring 2014), the dominant reads belonged to *Flavobacteriia*. In agreement with the total taxonomic profiling of healthy octocorals [28] (Additional file 2: Figure S2), chitinolytic communities were found to be conserved, at the class level, across different host species (Fig. 4a), presenting a remarkable dominance of *Gammaproteobacteria*. Also congruent with the total microbiome make-up of octocorals, we observed an enrichment of *Flavobacteriia-*derived chitinases, with consequent reduction in the abundance of *Gammaproteobacteria*-derived chitinases, in necrotic as compared to healthy tissues of the octocoral *E. gazella*. Further divergence between healthy versus necrotic octocoral tissue or octocoral versus sponge chitinolytic assemblages was as well identified, as expected, at lower taxonomic ranks (Additional file S1: Table S7). For instance, the healthy tissues of all octocoral species had higher proportions of chitinase sequences from unclassifiable *Gammaproteobacteria* (between 33% and 44%) than that observed for necrotic *E. gazella* tissue (20%), again reinforcing trends observed for the total microbiome of octocorals (Additional file 2: Figure S2). Further, from the pool of *Gammaproteobacteria* endo-chitinase reads that could be further classified into the order *Vibrionales*, higher proportions were found in necrotic (27%) than in healthy *E. gazella* tissues (from 6 to 15%), corroborating our estimates of *Vibrio* relative abundances in these samples (Additional file 1: Table S2). Moreover, the healthy microbiome of octocorals contained chitinase reads affiliated with other *Gammaproteobacteria* genera such as *Aliivibrio*, *Enterovibrio*, *Shewanella*, and *Pseudoalteromonas*, all of which could be isolated from healthy octocorals and were shown to possess in vitro endo-chitinase activity (Table 1). Few endo-chitinase reads belonging to the recently described genus *Ardenticatena* (class *Ardenticatenia*) [32] were found in healthy octocorals (Fig. 4a, Additional file 1: Table S7). *Gammaproteobacteria* chitinase reads from the sponge microbial metagenome which were classifiable at the genus level affiliated with the genera *Grimontia*, *Vibrio*, and *Microbulbifer*, the latter two also representing chitin-degrading taxa that we could cultivate from marine sponges (Table 1).

## Discussion

Chitin degradation is a keystone process in the oceans. Yet our knowledge of the prevailing microorganisms and metabolic pathways mediating the breakdown of chitin and its derivatives across the highly heterogeneous marine environment is scant. Beier et al. [33] revealed that the structure of endo-chitinase encoding genes within aquatic microbiomes responded significantly to salinity gradients, suggesting that chitinolytic processes, although ubiquitous, are influenced by abiotic factors. In this study, we integrated cultivation-independent and -dependent techniques to approach the ecology of chitin and COSs degradation within the microbiomes of foundational sessile marine invertebrates, placing emphasis on the organisms, genes, and enzymes involved in these processes and addressing the hypotheses of divergent chitin catabolism pathways and chitinolytic communities across host-associated and free-living marine biotopes.

### Chitin degradation capacities revealed for cultivatable symbionts of sponges and octocorals

The observation made in this study that a range of culturable octocoral and marine sponge-associated bacteria possess chitin/COSs-degrading abilities allows hypotheses to be raised on the relevance of chitin/COSs breakdown within complex marine symbioses and their role in C and N cycling in marine ecosystems. Such hypotheses are particularly intriguing given the enormous filter- and suspension-feeding capacities of marine sponges and corals, which may lead to high turnover rates of dissolved and particulate organic matter [34]. Caution is needed when drawing conclusions on microbiome-driven processes and functions solely based on the activity of cultivated microorganisms. Most of the dominant bacterial symbionts of marine sponges are recalcitrant to cultivation in the laboratory, and cultured representatives have been previously suggested to belong to the “rare biosphere” within these systems [24, 25, 35]. Conversely, higher cultivability has been observed for octocoral-associated bacterial communities as several moderately abundant/dominant bacterial associates of *Eunicella labiata*, except for the canonical coral symbionts belonging to the family *Endozoicomonadaceae* (*Gammaproteobacteria*), could be recently retrieved in culture [23]. Estimates of relative abundance of our chitin/COSs degrading isolates corroborate the trends above, suggesting that, collectively, they may represent a minority portion of the total microbiomes of sponges and octocorals, being particularly less abundant in the former host. For details on chitin degradation by the bacterial taxa analyzed in this study, including a thorough assessment of genomics traits involved in chitin metabolism among *Aquimarina* species, see Additional file 2: Extended discussion.

### Evidence of substrate cross-feeding among chitin degraders and utilizers in host-associated microbiomes

In highly diverse and complex microbiomes, the release of hydrolysis products by extracellular enzymes can trigger several modes of inter-specific cross-feeding (reviewed recently by Smith et al. [36]. One such mode, referred to as “substrate cross-feeding,” reflects the utilization, by one given organism, of substrates or molecules produced by the metabolism of another organism, being that either organism can still metabolize these products [36]. Regarding chitin degradation, the establishment of interspecific substrate cross-feeding cascades has been considered plausible [2] since some bacteria that grow on GlcNAc [37] or (GlcNAc)_2_ [38] do not possess enzymes for chitinolytic activity, such as most of the sponge- and octocoral-associated *Alphaproteobacteria* cultivated in this study. Thus, a potential coupling between *Gammaproteobacteria* and *Aquimarina* spp. (or *Flavobacteriia* in general) with *Alphaproteobacteria* in the cycling of chitin could be envisioned where the former two are catabolizing the polymer, while the latter benefit from excess hydrolysis products to further process COSs and use GlcNAc residues. The balance between chitin “degraders” and “consumers” has been suggested to influence the chitin destination in a given setting, whereby the former may use GlcNac to produce energy and the latter to build their cell wall [2]. Diverse and abundant lineages within these three major bacterial classes are indeed present in marine sponge and octocoral microbiomes [18, 23, 25, 39], and potential substrate cross-feeding mechanisms between them could lead to continuous turnover of in/ad-host organic carbon and nitrogen, hence, affecting the functioning of the microbiomes they belong to and the surrounding ecosystem.

Altogether, potential for chitin and COSs degradation, along with utilization of GlcNac derived from these processes, could be identified for diverse, culturable symbionts of these animals. These organisms possess a generalist pattern of occurrence across sediments, seawater, and invertebrate hosts [21, 24, 40] and refs. therein). The diversity in domain architecture and sequence of their endo-chitinases likely equip them with a versatile metabolism fine tuned to process varied, eventually biotope-dependent forms of chitin microfibrils. Their usually low abundance in marine invertebrates suggests participation of “rare biosphere,” transient symbionts in chitin metabolism within these microbiomes, opening questions regarding the chitin-degrading capacities of, and potential substrate cross-feeding among, the pool of more dominant and obligate symbionts which remain uncultured (see below). Previous studies unveiled incongruent *chiA* and 16S rRNA gene tree topologies, suggesting that chitinase-encoding genes are subjected to horizontal gene transfer and duplication events which make them less suitable as phylogenetic markers [41, 42]. Our assessment of full-length endo-chitinase genes is in overall agreement with this perspective, and we provide context to their potential spread within marine invertebrate microbiomes in Additional file 2: Extended discussion.

### Chitin-degrading microbiomes are ubiquitous but possess divergent structures across marine biotopes

Shotgun metagenome sequencing and analyses of unassembled reads from seawater, sediments, octocorals, and marine sponges confirmed the presence of endo-chitinases, exo-chitinases, and polysaccharide deacetylases in all these microbiomes, favoring the notion of chitin degradation as a ubiquitous process in the oceans [43]. We also found evidence for distinct chitin-degrading microbiomes across biotopes, due to differences in both the proportions of key chitin metabolism traits and composition of chitinolytic taxa identified across host-associated and free-living microbiomes.

Endo-chitinase (EC 3.2.1.14) and chitin-binding protein-encoding genes were clearly less abundant in the sponge-associated microbiome as compared to surrounding environments. Yet, exo-chitinase (EC 3.2.1.52) and polysaccharide deacetylase-encoding genes were of similar or even higher abundance in *S. officinalis* in comparison with seawater and sediment, suggesting that inside the sponge mesohyl the processing of small oligomers and GlcNac prevails, together with the transformation of chitin to chitosan, rather than the hydrolysis of chitin polymers. This seems to be in contrast with the dynamics predicted for octocorals, where we found equivalent relative abundances of endo-chitinase encoding genes in the microbiomes of healthy octocoral tissue, sediment, and seawater. Further, although the proportion of chitin-binding protein CDSs in healthy octocoral tissue was often lower than that of seawater, in *E. gazella* and *E. verrucosa* specimens, they represent about 20-fold increase in comparison with the proportions registered for the sponge microbiome. Conversely, increased abundances of genes involved in the processing of small oligomers, GlcNac utilization, and in the deacetylation of polysaccharides were registered for free-living biotopes and necrotic octocoral tissue compared with healthy octocoral samples.

It is important to note that, in the octocoral metagenome dataset, both the epi- and endo-symbiotic consortium were sampled from octocoral tissue, whereas in the sponge metagenome dataset, only the endo-symbiotic consortium was sampled. Therefore, it cannot be ruled out that the epibiotic microbiome on octocorals and marine sponges may be more fit to hydrolyze the large chitin polymer whereas deep inside the animal tissue, the processing of oligomers is favored. Otherwise, the healthy octocoral microbiome could indeed possess a higher chitin-degrading efficiency, as evidenced by their higher proportions of endo-chitinase and chitin-binding protein-encoding genes. The latter are known to enhance the cells’ binding capacity to chitin substrates, enabling more efficient chitin degradation to occur. An explanation for this observation may be based on differential dietary preferences of sponges versus octocorals. Octocorals are suspension-feeders that capture organic detrital particles, phyto- and zooplankton, including diatoms, protists, and small crustaceans and their larvae [18, 44], which are naturally rich in chitin [43, 45]. A microbial community well-adapted to chitinous food processing and chitin polymer hydrolysis could therefore be beneficial for the octocoral holobiont. The same may be less relevant within the marine sponge mesohyl where the processing of bacterioplankton and dissolved or small particulate organic matter prevails [46, 47]. Moreover, it is known that glass sponges (Hexactinellida) and multiple keratose sponge species (Demospongia, Dictyoceratida; the group to which *S. officinalis* belongs) contain endogenous chitin as a structural component in their skeletons [45, 48]. The presence of a highly active chitinolytic endosymbiotic microbiome may thus be less favored in the inner sponge mesohyl as it could compromise sponge health and growth (if the sponge structures would become too much of a food source). Future, dedicated analyses of epibiotic microbiomes will be fundamental for a more comprehensive understanding of the chitin and COSs degradation potential of the marine sponge holobiont.

In agreement with the notion that chitinolytic communities make-up a small fraction of the total microbiome [2] and with abundance estimates shown in this study for cultivated, chitinolytic bacterial symbionts, relative abundance values for CDSs involved endo- and exo-chitinase activities were considerably low. This outcome also reflects the inherent nature of shotgun sequencing approaches whereby primary metabolism genes common to all community members dominate the data. Despite this limitation, the shotgun, primer-less strategy employed here enabled sufficient data retrieval for the comparative analysis of key functions involved in chitin metabolism. Although we addressed only the taxonomy of endo-chitinase encoding genes in this study (see below), the exploration of the total chitin-degrading assemblage, through the simultaneous inspection of deacetylase and exo-chitinase encoding genes, holds potential in further revealing the diversity and potential interactive networks mediating the process of chitin across marine settings.

Cottrell et al. had previously suggested that the taxonomic composition of cultivated and uncultivated chitin degrading microbiomes from the seawater surface is similar [42, 49]. Our results are in partial agreement with this perspective since most endo-chitinase metagenomic reads identified in this study affiliate with bacterial taxa well represented by our panel of culturable symbionts. However, the primer-less, cultivation-independent approach employed here also revealed a considerable portion of classifiable and unclassifiable bacterial chitinase reads across all host-associated and free-living biotopes, representative of bacterial clades which apparently evade current cultivation attempts. The most remarkable example of this outcome is the affiliation of most octocoral-derived endo-chitinase reads with unclassified, uncultivated *Gammaproteobacteria* (Fig. 4, see Additional file 1, Table S7 for details).

In fact, the taxonomic composition of the chitinolytic communities in octocorals seemed to closely follow the overall microbiome composition in these animals where uncultivated *Gammaproteobacteria*—often affiliated with the order *Oceanospirillales*, family *Endozoicomonadaceae* based on 16S rRNA gene assessments [23]—largely dominate the healthy octocoral tissue, while in necrotic octocoral tissue *Flavobacteriia*, including *Aquimarina* spp., strongly increase in abundance [28]. This outcome supports the hypothesis that dysbiosis of the octocoral holobiont involves depletion of thus-far unculturable and unclassifiable, typical coral-associated *Gammaproteobacteria* which may play an important role as chitin degraders in this system. Indeed, the chitinolytic microbiomes of necrotic octocoral tissues seemed to resemble much more those of seawater and sediments, where exo-chitinase- and polysaccharide deacetylase-encoding genes were more abundant.

As mentioned above, the healthy tissues of all octocoral species had higher proportions of chitinase sequences from unclassifiable *Gammaproteobacteria*, allowing hypotheses to be raised on a potential role for these elusive symbionts in C and N turnover within corals. Ongoing research on metagenome-assembled genomes (MAGs) retrieved from our octocoral specimens supports this hypothesis as endo-chitinase genes were detected on MAGs belonging to the typical coral symbiont family *Endozoicomonadaceae* (Keller-Costa et al., unpublished data). Likewise, the detection of endo-chitinase gene reads belonging to the genus *Ardenticatena* [32, 50] suggests an unanticipated, potential participation of *Choloroflexi* symbionts in chitin degradation within animal invertebrates. Strengthening this notion is our own documentation of *chiA* genes on *Chloroflexi* MAGs retrieved from marine sponges (Silva et al., unpublished data) and the recent observation that *Chloroflexi* spp. contribute to chitin degradation in freshwater sediments [13]. Clearly, advanced techniques to link microbial identity and function such as single-cell genomics and metagenome-resolved genomics hold great potential to further disentangle the diversity of symbiotic microorganisms involved in chitin degradation and utilization processes, strengthening and validating predictions based on metagenome functional profiling. Future, direct estimates of chitinolytic activity in samples collected in situ (either “holobiont” or host-derived microbial cells) bear promise in solidifying the status of host-associated microbiomes as important chitin degradation settings in the marine environment.

Our data suggest that marine host-associated microbiomes do possess potential for chitin hydrolysis (particularly octocorals) and chitin deacetylation (more pronouncedly in sponges but also in octocorals), leading to the production of COSs or chitosan, respectively, and for COSs breakdown and utilization (particularly sponges). However, we emphasize that none of the genomic features underlying the functions above were found to be pronouncedly enriched in host-associated microbiomes in comparison with their environmental vicinities. Therefore, the data reported here do not support the notion of sponges and octocorals as “fast processing hubs” of chitin, COSs, or chitosan when contrasted with free-living microbiomes. Marine sponges and corals are a part of benthic, suspension-feeding communities which are known to regulate carbon flux between pelagic and benthic zones and affect the biogeochemical cycling of key nutrients [34, 51]. By removing large amounts of particulate or dissolved organic matter from the water column, these holobionts are among the most efficient in uptaking and processing energy in marine ecosystems [34]. It seems hence plausible that sponges and octocorals, given the genomic and metagenomic features revealed in this study, are players in elemental turnover through their chitin degradative ability, since chitin presents a significant and critical connection between the carbon and nitrogen cycles in the marine environment [43]. Our study proposes that unique chitin-degrading communities characterize distinct marine biotopes. Thus, a differential capacity to process chitin and its derivatives is likely to exist even though the abundance of the genes involved in chitin breakdown may not significantly differ from one micro-habitat to the other in some cases.

## Conclusion

Our study provides evidence for the existence of biotope-specific chitin-degrading communities in the marine realm. This suggests that differential substrate affinities, polymer versus oligomer uptake and degradation aptitude, and carbon and nitrogen turnover rates dictate multiple processing modes of chitin and chitin-derivatives across distinct micro-niches in the oceans. It is yet to be verified whether such patterns are applicable to a broad range of coral and sponge holobionts and marine environments. Moreover, the multiphasic approach employed in this study enabled us to infer possible substrate cross-feeding patterns among symbionts which may support chitin turnover within sessile marine invertebrates, contributing to the co-existence of chitin and COSs degrading bacteria in symbiotic communities. We further highlight *Aquimarina* species as source of putative novel chitinolytic enzymes and break new ground regarding the potential chitin degradation roles of hallmark *Gammaproteobacteria* symbionts of corals and understudied symbionts in the *Choloroflexi* phylum. Future research shall tackle their fundamental properties, hopefully opening new opportunities to further explore marine biomes and understudied microbial clades for biocatalysts of interest in applied-oriented research.

## Methods

### Biological resources and approach

Forty-one bacterial isolates from two previously established culture collections derived from the octocoral *Eunicella labiata* [23] and the marine sponges *Sarcotragus spinosulus* and *Ircinia variabilis* [40] were used in this study to address the chitin degradation capacities of culturable symbionts of octocorals and marine sponges. The isolates were subjected to chitin degradation and chitinase activity bioassays, PCR-amplification of chitinase-encoding genes, and Pfam-based annotations to mine for protein domains involved in chitin and COSs degradation, chitin deacetylation and GlcNac utilization (when genome sequences were available—Table 1) underlying chitin degradation ability. To address the relative abundance of the abovementioned functional features mediating chitin consumption and the taxonomic composition of chitinase-encoding genes in seawater, marine sediments, sponges, and octocorals, 30 Illumina-sequenced microbial metagenomes representing two different datasets were examined using dedicated in silico analyses. These datasets are herein termed (1) the “sponge metagenome dataset,” already published elsewhere [27], and the “octocoral metagenome dataset” which is an original contribution of this study (chitin metabolism features) and of a parallel study (in press) on the taxonomy and function of the total microbiome [28]. Briefly, the sponge metagenome dataset consists of four microbiomes sampled from the inner body of four independent *Spongia officinalis* specimens, three independent microbiome samples from seawater, and three independent microbiome samples from sediments [27]. The octocoral metagenome dataset comprises 13 microbial metagenomes sampled from the tissues of three octocoral species (3× healthy *Eunicella gazella* tissue, 3× necrotic *E. gazella* tissue (same three specimens), 4× healthy *E. verrucosa*, and 3× healthy *Leptogorgia sarmentosa*) specimens along with four microbial metagenomes from seawater and three from sediments see [28]. All samples, from both datasets, have been collected in the same location off the coast of Algarve, South Portugal (“Pedra da Greta”: Lat. 36° 58′ 47.2 N, Long. 7° 59′ 20.8 W”). Detailed procedures regarding sampling, metagenome DNA extraction and sequencing, and general features of all metagenome samples from the abovementioned datasets are provided as Supplementary information (Additional file 2, Detailed methodology).

### Bacterial strains

Of the 41 marine bacterial strains screened for chitinolytic activities in this study, 24 were retrieved from the octocoral *Eunicella labiata* by Keller-Costa et al. [23], while 17 were obtained from Irciniidae sponges by Esteves et al. [40] (Table 1). Each isolate represents a unique phylotype/genotype in its corresponding source study and makes part of an in-house collection of microbial symbionts. Isolates are available upon request. Prior to chitinolytic activity assays, all strains were re-activated from glycerol stocks and grown in half-strength Marine Broth (MB 1:2; ROTH Navarra, Spain) made with 1:1 v/v dH_2_O: artificial seawater (for composition, see [40]).

### Chitin degradation activity screening

Chitin degradation by the target isolates was tested with a Petri dish assay on colloidal chitin (CC) agar medium prepared with sterile artificial seawater. Eight replicates per isolate were used in the bioassays. After inoculation, CC plates were incubated at RT for 14 days. The whitish turbidity of the CC medium allows for visual evaluation of chitin degradation through clearing zones (haloes) around the inoculation spot. A semi-quantitative analysis of chitin-degrading activity was performed by measuring the radius of the haloes produced (see legend to Table 1 for details). For specifics on CC preparation, medium composition, and inoculation procedures, see Additional file 2: Detailed methodology.

### Endo- and exo-chitinase activity assays

Chitinolytic enzyme activity was determined fluorometrically for the 41 strains studied using the chitinase assay kit (CS0980) from Sigma-Aldrich/Merck, following the manufacturer’s instructions, and a multi-mode microplate reader (Filter Max F5, Molecular Devices). For specifics on sample preparation prior to endo-chitinase and exo-chitinase activity assays, please see Additional file 2: Detailed methodology. In brief, enzymatic activities were measured as the release of 4-methylumbelliferone (4-MU) from various 4-MU labeled substrates. Exo-chitinase (EC 3.2.1.52) activities were detected using the substrates 4-methylumbelliferyl N-acetyl-β-D-glucosaminide and 4-methylumbelliferyl N,N′-diacetyl-β-D-chitobioside hydrate to detect N-acetyl-β-glucosaminidase (release of GlcNAc monomers) and chitobiosidase (release of GlcNAc dimers) activity, respectively. Endo-chitinase (EC 3.2.1.14) activity was detected using 4-methylumbelliferyl β-D-N,N′,N″-triacetylchitotriose as substrate (release of GlcNAc trimers). All assays were performed at substrate concentrations of 0.5 mg/mL and sample volumes of 10 μL. Further details on physical-chemical parameters used in the assays and registration of results are provided in Additional file 2: Detailed methodology.

### PCR amplification of *chiA* gene fragments

PCR amplification of *chiA* gene fragments—targeting “group A” glycoside hydrolase family 18 endo-chitinases (EC 3.2.1.14), based on amino acid sequences of this catalytic domain [52], was carried out on genomic DNA of each strain analyzed in this study. The primer pair *chiA*_F2/*chiA*_R2 (*chiA*_F2, 5′-CGT GGA CAT CGA CTG GGA RTW YCC-3′ and *chiA*_R2, 5′-CCC AGG CGC CGT AGA RRT CRT ARS WCA-3′) was employed, which generates amplicons of approximately 240 bp [53]. Details on thermal cycling, Sanger sequencing, and phylogenetic inference of the *chiA* sequences obtained are provided in Additional file 2: Extended results; Figure S3.

### Genome-wide assessment of chitin metabolism traits in bacterial isolates from sponges and octocorals

Sixteen of the 19 bacterial genomes (available from the panel of 41 strains) investigated in this study for chitin/COSs breakdown and utilization features have been published elsewhere (for octocoral-derived bacterial genomes see [20, 54–56]; for marine sponge-derived bacterial genomes, see [26, 57]), while the three *Vibrio* sp. genomes Vb255, Vb258, and Vb339 are original to this study. Genomic DNA was extracted using the Wizard genomic DNA purification kit (Promega, Madison, USA) from a pure, culture freshly grown in MB 1:2 and was sequenced on an Illumina MiSeq platform, as described elsewhere [26]. The sequence reads were assembled de novo into contigs with the NGen DNA assembly software by DNAStar, Inc., and the contigs underwent taxonomic identification and quality checks as described in Karimi et al. [24]. Coding sequence predictions were performed with the Rapid Annotation using Subsystem Technology (RAST) prokaryotic genome annotation server, version 2.0 [58]. Amino acid fasta files obtained from RAST were used as input data for protein families (Pfam)-based annotations using the WebMGA server [59] as explained in detail by Silva et al. [21]. We mined the data for Pfam entries underlying endo- and exo-chitnase activities, chitin deacetylation into chitosan (polysaccharide deacetylases), transport of chitin oligosaccharides, and N-acetylglucosamine utilization (Fig. 1; see Additional file 1: Table S1 for a complete list of the Pfam entries used).

### Phylogenetic analysis of chitinase encoding genes in bacterial strains

We used the Rapid Annotation Using Subsystem Technology (RAST) v2.0 server (http://rast.nmpdr.org) [60] to identify full endo-chitinase (EC 3.2.1.14) gene sequences from the 19 bacterial symbionts examined here for which whole genomes are available [20, 26, 54–57] (Table 1, Fig. 1). This resulted in 96 predicted full-length endo-chitinase CDSs annotated by RAST across the 19 genomes. These CDSs were then subjected to translation followed by Pfam annotations using the EMBOSS Transeq (https://www.ebi.ac.uk/Tools/st/emboss_transeq) and hmmscan (https://www.ebi.ac.uk/Tools/hmmer/search/hmmscan) algorithms of EMBL-EBI. Forty-seven of the 96 CDSs were found to encode for either a GH18 or GH19 endo-chitinase domain according to Pfam annotations. These were selected for tree construction along with further 11 CDSs from our genomes which presented high levels of homology with endo-chitinase sequences present in NCBI’s protein database. Closest and moderately close endo-chitinase gene relatives (*n* = 32) to the abovementioned sequences were included in the analysis, totaling 90 full endo-chitinase gene sequences spanning four bacterial phyla (*Proteobacteria*, *Bacteroidetes*, *Actinobacteria*, and *Firmicutes*) and 16 formally described genera.

The phylogenetic tree was constructed using the MEGAX software package [61]. Chitinase sequences were aligned using ClustalW, after which the most suitable evolutionary model for each dataset was inferred. The Generalized Time Reversible model (GTR) was considered the best fit in both cases and was used for phylogenetic inference with the Maximum Likelihood method. Tests of phylogeny consisted of 1000 bootstrap repetitions. Initial tree(s) for the heuristic search were obtained automatically by applying Neighbor-Joining and BioNJ algorithms to a matrix of pairwise distances estimated using the Maximum Composite Likelihood (MCL) approach, and then selecting the topology with superior log likelihood value. Trees were drawn to scale, with branch lengths measured in the number of substitutions per site [61].

### Relative abundance of chitin metabolism coding sequences across host-associated and free-living microbiomes

To determine whether the relative abundance of genes involved in chitin/COSs degradation and GlcNAc utilization (the same functional categories and enzymes as addressed in Fig. 1) differed between biotopes, we explored InterPro (IPR) functional annotations obtained from unassembled reads (101 bp) of the marine sponge [27] and octocoral metagenome [28] datasets using the European Bioinformatics Institute (EMBL-EBI) metagenomics analysis pipeline MGnify [29]. Shortly, within MGnify, reads are subjected to coding sequences (CDSs) prediction using FragGeneScan [62]. The InterProScan procedure is then employed for functional annotation of CDSs against the latest release of the IPR database, which integrates several protein sequence databases such as Pfam, TIGRFams, and PANTHER. Contingency IPR versus sample tables were retrieved for each dataset and examined for IPR entries involved in key chitin metabolism processes: chitin and chitin-oligosaccharide hydrolysis, using relative abundances of endo-chitinase (EC 3.2.1.14) and exo-chitinase (EC 3.2.1.52) encoding genes as proxies; chitin-binding ability, using the relative abundance of chitin-binding proteins as proxies; potential chitin deacetylation, using the relative abundances of polysaccharide deacetylases as proxies; and N-acetylglucosamine utilization, using the relative abundance of glucosamine-6-phosphate isomerase (EC 3.5.99.6) encoding genes as proxies (see Additional file 1: Table S5 for details on IPR entries used). Individual IPR entries related to each of the chitin metabolism functions named above were compiled and summed together to represent the abundance of each inspected function in the corresponding metagenome. To normalize the data, the absolute numbers of CDSs assigned to IPR entries were subjected to Hellinger transformation (i.e., calculation of relative abundance values followed by square root transformation of relative abundances). Thereafter, mean Hellinger-transformed abundance values and standard errors were calculated for each analyzed function in each biotope and statistical analyses were carried out. Normality was confirmed using the Shapiro-Wilk test. For the analysis of the sponge and the octocoral metagenome datasets encompassing all healthy octocoral species plus sediment and seawater samples, one-way ANOVA was used followed by a Tukey’s post hoc test if significant. For the analysis of microbial metagenomes from healthy versus necrotic *Eunicella gazella* tissue, a Student’s *t* test was used.

### Taxonomic classification of chitinase-encoding genes from microbial metagenomes

To examine the taxonomic composition and structure of chitinolytic microbiomes across the studied biotopes in a cultivation-independent manner, we used the Meta-Genome Rapid Annotation using Subsystems Technology server (MG-RAST) v3.0 [29] with default search parameters. This tool was used to mine for endo-chitinase protein sequences (EC 3.2.1.14) from unassembled reads across all samples in both datasets. Briefly, default MG-RAST procedures comprise gene calling with FragGeneScan [62] and translation of predicted CDSs into proteins with clustering set at 90% homology using the Uclust algorithm [63]. Translated reads are then annotated using the best-hit annotation tool against the M5NR database [64]. The stringency of the BLAST parameter is a maximum *e* value of 1e-5, a minimum sequence identity of 60%, and a minimum alignment length of 15 aa for the predicted proteins. All sequence entries from each of the 30 surveyed metagenomes (10 from the sponge metagenome and 20 from the octocoral metagenome dataset) assigned as endo-chitinases (EC 3.2.1.14) by MG-RAST under the abovementioned parameters were downloaded. Within each dataset, endo-chitinase reads from replicate samples of the same biotope were pooled and then blasted against the NCBI protein sequence database using the blastx algorithm with an *e* value cut-off of 10. The resulting alignment files were analyzed using the MEGAN6 software package [65] to obtain taxonomic assignments. During this analysis, we found that not all the sequences that were classified as endo-chitinases (EC 3.2.1.14) by MG-RAST also had necessarily an endo-chitinase sequence as their closest blastx hit (Table 2). Therefore, taxonomic assignments shown in this study considered only those reads identified by MG-RAST that also had an endo-chitinase sequence as their closest blastx hit and that could as well be taxonomically assigned to the domain Bacteria.

In this study, inference of gene relative abundances (Fig. 3) and taxonomic assignments (Fig. 4) were performed using unassembled reads to make best use of the total sequencing effort employed in the generation of the sponge and octocoral metagenome datasets. This way, we could integrate sediment samples in the comparative scheme, since metagenome assemblies of this biotope are usually poor due to its extremely high microbial diversity—resulting in few and short contigs and usage of less than 5% of the generated reads [27]. Preliminary analyses revealed, moreover, that taxonomic and functional assignments of assembled and unassembled metagenomes were highly congruent for seawater, octocoral [28], and sponge [27] samples, thus supporting our choice to assess unassembled reads as a suitable and robust means to achieve the goals established in this study.

## Supplementary Information

**Additional file 1: Table S1.** Protein family (Pfam) entries involved in chitin and chito-oligosaccharides degradation and N-acetylglucosamine utilization used in Figure 1. **Table S2.** 16S rRNA gene-based estimates of relative abundance of culturable bacterial genera in the microbiomes of octocorals and sponges. **Table S3.** Genome-based estimates of relative abundance for culturable, chitinolytic symbionts of sponges and corals in their respective microbiomes. **Table S4.** Features of 90 (nearly) full-length coding sequences displaying endo-chitinase catalytic domains (from glycoside hydrolase families 18 and 19, Pfam-based annotations) used for phylogenetic inferences in Figure 2. **Table S5.** InterPro (IPR) abundance of coding sequences involved in chitin and chito-oligosaccharides degradation and N-acetyl-glucosamine utilization in (a) the marine sponge metagenome dataset (project accession number: PRJEB11585) and (b) the octocoral metagenome dataset (project accession number: PRJEB13222), retrieved using the MGNify (EMBL-EBI) pipeline. **Table S6.** Endo-chitinase (EC 3.2.1.14) nucleotide sequences retrieved from (a) the marine sponge (*Spongia officinalis*) metagenome dataset (project accession number: PRJEB11585) and (b) the octocoral metagenome dataset (project accession number: PRJEB13222), using the MG-RAST metagenomics analysis server (version 4.0.3). **Table S7.** Genus-level taxonomy of endo-chitinase gene reads in the microbial metagenomes of sponges and corals.

**Additional file 2: **Detailed Methodology, Extended Results and Discussion. **Supplementary Figure S1.** RAST annotation of chitin and chitin-derivative degradation and utilization genes in cultivated bacterial symbionts of sponges and octocorals. For details on strains and phylogenetic tree, see legend to Fig. 1. The table on the right side shows chitin degradation (including both hydrolysis and deacetylation processes) and N-acetylglucosamine transport and utilization encoding genes detected on each bacterial genome using **RAST-based classification**, in contrast with Pfam annotations show in Fig. 1. Values in each cell correspond to the respective coding sequence (CDS) numbers present in each genome, whereby higher CDS numbers are highlighted in dark-gray shading. Entries highlighted in bold represent chitin processing functions examined across the sponge and octocoral metagenome datasets (Fig. 3), while the phylogeny, diversity and taxonomic composition of endo-chitinase encoding genes (EC 3.2.14) are examined in Figs. 2 and 4. For each functional entry, enzyme commission (EC) numbers and specific terminology are given in brackets, when appropriate. ^**1**^Chitinases that hydrolyse chitin oligosaccharides - (GlcNAc)_4_ to (GlcNAc)_2_ and (GlcNAc)_5,6_ to (GlcNAc)_2_ and (GlcNAc)_3_ but are inactive toward chitin (UniProtKB P96156). ^**2**^Corresponds to InterPro database entry IPR002509 (see also Fig. 3) which describes the metal-dependent deacetylation of O- and N- acetylated polysaccharides such as chitin, peptidoglycan and acetylxylan. **Supplementary Figure S2.** Class-level prokaryotic community profiles of healthy (EG_H) and diseased (EG_N) *Eunicella gazella* tissue, healthy *Eunicella verrucosa* (EV01-EV04) and *Leptogorgia sarmentosa* (LS06-LS08) specimens as well as seawater (SW01-SW04) and sediment samples (SD01-SD03). Taxonomic assignments are based on 16S rRNA gene reads retrieved from unassembled metagenomes using the MGnify metagenomics pipeline version 2.0 (EMBL-EBI) for the octocoral metagenome dataset (project PRJEB13222). Relative abundances are displayed for taxa representing more than 1% of the total dataset reads. Taxa with abundances below 1% across the data are collectively labelled as “rare classes”. **Supplementary Figure S3**. Maximum Likelihood phylogenetic tree of *chiA* gene sequences amplified from bacterial isolates. Sequences were obtained for eight marine sponge and 11 octocoral-derived bacterial isolates through PCR amplification from their respective genomic DNA. The evolutionary history was inferred using the General Time Reversible model. The tree with the highest log likelihood (-892.58) is shown. The percentage of trees in which the associated taxa clustered together is shown next to the branches (1,000 bootstrap replicates). A discrete Gamma distribution was used to model evolutionary rate differences among sites (5 categories (+*G*, parameter = 3.1129)). The rate variation model allowed for some sites to be evolutionarily invariable ([+*I*], 19.83% sites). The tree is drawn to scale, with branch lengths measured in the number of substitutions per site. The analysis involved 19 nucleotide sequences. Codon positions included were 1st+2nd+3rd+Noncoding. All positions containing gaps and missing data were eliminated. There was a total of 164 positions in the final dataset.

## Data Availability

The 16S rRNA gene sequences of marine sponge and octocoral bacterial isolates were deposited at NCBI GenBank under the accession numbers HE818111–HE818389 [40] and MF461358-MF461394 [23]. *ChiA* gene sequences of sponge and octocoral bacterial isolates were also deposited at NCBI GenBank under the accession numbers MK570943-MK570958 and MK675637-MK675639. Full genome sequences of sponge and octocoral bacterial isolates were deposited in public databases by Raimundo et al. [54] (bacterial genomes retrieved from *Eunicella labiata*); Díez-Vives et al. [57] (*Aquimarina* genomes from marine sponges); and Gonçalves et al. [26] (*Vibrio* sp. Vb278), and accession numbers are provided in Table 1. The genome sequence of *Vibrio* sp. Vb339 has been submitted in this study to the European Nucleotide Archive/European Molecular Biology Laboratory (ENA/EMBL) under the assembly accession number GCA_902751245.1. Shotgun microbial metagenome sequences examined here were deposited at the European Nucleotide Archive (ENA/EMBL): the octocoral dataset (20 metagenomes [28]) under the study accession number PRJEB13222 and the sample accession numbers SAMEA3913358 to SAMEA3913367, and the sponge dataset (10 metagenomes [27]) under the study accession number PRJEB11585 and the sample accession numbers SAMEA3642063 to SAMEA3642072.
