# Supplementary material for: Functional metagenomics reveals differential chitin degradation and utilization features across free-living and host-associated marine microbiomes
Source: Microbiome. 2021 Feb 14;9:43. doi: 10.1186/s40168-020-00970-2 (PMC7883442; doi:10.1186/s40168-020-00970-2)
Supplement: Supplementary file 3 — Additional file 2: Detailed Methodology, Extended Results and Discussion. Supplementary Figure S1. RAST annotation of chitin and chitin-derivative degradation and utilization genes in cultivated bacterial symbionts of sponges and octocorals. For details on strains and phylogenetic tree, see legend to Fig. 1. The table on the right side shows chitin degradation (including both hydrolysis and deacetylation processes) and N-acetylglucosamine transport and utilization encoding genes detected on each bacterial genome using RAST-based classification, in contrast with Pfam annotations show in Fig. 1. Values in each cell correspond to the respective coding sequence (CDS) numbers present in each genome, whereby higher CDS numbers are highlighted in dark-gray shading. Entries highlighted in bold represent chitin processing functions examined across the sponge and octocoral metagenome datasets (Fig. 3), while the phylogeny, diversity and taxonomic composition of endo-chitinase encoding genes (EC 3.2.14) are examined in Figs. 2 and 4. For each functional entry, enzyme commission (EC) numbers and specific terminology are given in brackets, when appropriate. 1Chitinases that hydrolyse chitin oligosaccharides - (GlcNAc)4 to (GlcNAc)2 and (GlcNAc)5,6 to (GlcNAc)2 and (GlcNAc)3 but are inactive toward chitin (UniProtKB P96156). 2Corresponds to InterPro database entry IPR002509 (see also Fig. 3) which describes the metal-dependent deacetylation of O- and N- acetylated polysaccharides such as chitin, peptidoglycan and acetylxylan. Supplementary Figure S2. Class-level prokaryotic community profiles of healthy (EG_H) and diseased (EG_N) Eunicella gazella tissue, healthy Eunicella verrucosa (EV01-EV04) and Leptogorgia sarmentosa (LS06-LS08) specimens as well as seawater (SW01-SW04) and sediment samples (SD01-SD03). Taxonomic assignments are based on 16S rRNA gene reads retrieved from unassembled metagenomes using the MGnify metagenomics pipeline version 2.0 (EMBL-EBI) for the octocoral m [file 40168_2020_970_MOESM3_ESM.docx]

**Supplementary Information – Additional File 2**

**Title: Evidence for specificity of chitin-degrading microbiomes across free-living and host-associated marine biotopes**

**Running title:** Chitin-degrading microbiomes in marine habitats.

**Authors:**

Raimundo, I.^1$^, Silva, R. ^1$^, Meunier, L.^1,2^, Valente, S.M.^1^, Lago-Lestón, A.^3^, Keller-Costa, T.^1*^ and Costa, R.^1,4,5*^

**Affiliations:**

^1^Instituto de Bioengenharia e Biociências, Instituto Superior Técnico (IST), Universidade de Lisboa, 1049-001 Lisbon, Portugal

# ^2^Laboratory of Aquatic Systems Ecology, Université Libre de Bruxelles, Brussels, Belgium

^3^Centro de Investigación Científica y de Educación Superior de Ensenada (CICESE), 22860, Ensenada, Mexico

^4^Centro de Ciências do Mar (CCMAR), Universidade do Algarve, 8005-139 Faro, Portugal

^5^Department of Energy, Joint Genome Institute, Walnut Creek, California 94598, USA, and Lawrence Berkeley National Laboratory, Berkeley, California 94720, USA.

**E-mail contacts:**

Raimundo, Inês [ines.goncalvesraimundo@kaust.edu.sa](mailto:ines.goncalvesraimundo@kaust.edu.sa)

Silva, Rúben [rubenrsilva@tecnico.ulisboa.pt](mailto:rubenrsilva@tecnico.ulisboa.pt)

Meunier, Laurence Laurence.Meunier@ulb.ac.be

Valente, Sara M. sara.martinez@tecnico.ulisboa.pt

Lago-Lestón, Asunción: alago@cicese.mx

Keller-Costa, Tina: tinakellercosta@tecnico.ulisboa.pt

Costa, Rodrigo: rodrigoscosta@tecnico.ulisboa.pt

^*^Address correspondence to Rodrigo Costa ([rodrigoscosta@tecnico.ulisboa.pt](mailto:rodrigoscosta@tecnico.ulisboa.pt)) and Tina Keller-Costa ([tinakellercosta@tecnico.ulisboa.pt](mailto:tinakellercosta@tecnico.ulisboa.pt)); Instituto de Bioengenharia e Biociências, Instituto Superior Técnico (IST), Universidade de Lisboa, Av. Rovisco Pais 1, Torre Sul, Piso 11, 11.6.11b, 1049-001 Lisbon, Portugal, Tel: (+351) 21 841 9167.

^$^these authors contributed equally to this work

**Running title:** Chitin-degrading microbiomes in marine habitats.

**Detailed Methodology**

**Generation of the octocoral metagenome dataset - PRJEB13222 [1]**

Sampling of octocoral specimens, seawater and sediments for microbial metagenome sequencing took place on the 17^th^ of June 2014 by SCUBA diving at 18 m depth off the coast of Faro, Algarve, Portugal (lat./lon.: 36.979778/-7.989111). It comprised three independent specimens (individuals) of the octocoral species *Eunicella gazella* (from which healthy and necrotic tissue samples could be obtained, herein termed “healthy” and “necrotic” samples), four specimens of *Eunicella verrucosa* (healthy tissue only), and three specimens of *Leptogorgia sarmentosa* (healthy tissue only), along with four and three independent samples of seawater and sediment, respectively. Overall, sample collection, transportation, processing, microbial metagenome DNA extraction and sequencing followed the procedures used by Karimi et al., 2017 [2] for the generation of the sponge metagenome dataset, with slight modifications to increment metagenomic DNA yields from octocoral specimens. Shortly, for each sampled specimen the internal gorgonin skeleton was removed and *c.* 2.5 g of octocoral tissue was macerated in calcium-magnesium-free sterile artificial seawater [3] with mortar and pestle, after which the resulting cell homogenates were subjected to a differential centrifugation step [3] to discard host-derived cells and obtain a total-community, octocoral-derived microbial cell pellet (MCP). Seawater (2L) samples were taken 1 m above the sampled octocoral specimens and filtered through 0.22 uM nitrocellulose filters, which were cut aseptically into small pieces and stored at -80 ^o^C. Sediment samples (c. 50 g) were taken independently from one another at about 1 m distance from the octocoral specimens. From each homogenized sediment sample, 0.25 g portions were taken and stored separately at -80 ^o^C. Metagenomic DNA extraction was carried out from octocoral-derived microbial pellets, nitrocellulose filters and 0.25 g sediments obtained as described above using the UltraClean DNA isolation kit (MO BIO, Carlsbad, CA, United States) according with the supplier’s instructions. DNA concentrations were determined using the Qubit dsDNA HS Assay Kit (Life Technologies Qubit 2.0®), after which Illumina sequencing was carried out on a Hiseq 2500 apparatus at Mr. DNA (Shallowater, TX, United States). In brief, DNA libraries were prepared with the Nextera DNA sample preparation kit (Illumina) following the manufacturer’s procedures and sequenced paired end for 200 cycles with sequence depth calibrated at c. 20 million 101-bp reads per sample.

**Table AF2.1.** Number of sequence reads per quality control step using the MGnify metagenomics pipeline (EMBL-EBI), version 2.0 (project PRJEB13222).

| **Sample ID** | **Sample description** | **Raw reads^1^** | **Initial reads^2^** | | | **Trimming^3^** | **Length filtering^4^** | | **Predicted 16S rRNAs^5^** | | **Predicted CDSs^6^** | | **% CDSs in HQ reads^7^** | **CDS with known function^8^** | **% CDSs with known function^9^** |
| --- | --- | --- | --- | --- | --- | --- | --- | --- | --- | --- | --- | --- | --- | --- | --- |
| EG15_H | *Eunicella gazella* 15 healthy | 28382458 | 21 737 057 | | | 21 610 704 | 15 367 812 | | 16 702 | | 12 589 125 | | 81,92 | 1 742 039 | 13,84 |
| EG15_N | *Eunicella gazella* 15 necrotic | 26751376 | 22 696 306 | | | 22 596 103 | 15 832 554 | | 11 270 | | 14 144 189 | | 89,34 | 2 601 241 | 18,39 |
| EG16_H | *Eunicella gazella* 16 healthy | 24746844 | 20 709 280 | | | 20 636 335 | 16 027 594 | | 15 318 | | 12 519 737 | | 78,11 | 976 189 | 7,80 |
| EG16_N | *Eunicella gazella* 16 necrotic | 19955560 | 18 882 110 | | | 18 767 466 | 13 130 887 | | 9 371 | | 12 187 829 | | 92,82 | 2 767 319 | 22,71 |
| EG18_H | *Eunicella gazella* 18 healthy | 22871484 | 21 061 751 | | | 20 984 435 | 16 652 694 | | 19 425 | | 14 652 694 | | 87,99 | 2 618 552 | 17,87 |
| EG18_N | *Eunicella gazella* 18 necrotic | 23802132 | 21 655 435 | | | 21 558 545 | 15 229 725 | | 10 771 | | 14 150 842 | | 92,92 | 3 254 999 | 23,00 |
| EV01 | *Eunicella verrucosa* 01 | 30132532 | 26 567 319 | | | 26 430 738 | 19 941 462 | | 21 715 | | 16 240 113 | | 81,44 | 2 631 133 | 16,20 |
| EV02 | *Eunicella verrucosa* 02 | 26648958 | 19 703 231 | | | 19 592 364 | 14 245 239 | | 10 400 | | 12 160 791 | | 85,37 | 1 767 915 | 14,54 |
| EV03 | *Eunicella verrucosa* 03 | 23773716 | 20 258 584 | | | 20 177 043 | 15 424 249 | | 12 445 | | 12 449 446 | | 80,71 | 1 154 130 | 9,27 |
| EV04 | *Eunicella verrucosa* 04 | 25167420 | 21 644 480 | | | 21 547 755 | 16 321 442 | | 19 796 | | 13 314 974 | | 81,58 | 2 363 625 | 17,75 |
| LS06 | *Leptogorgoa sarmentosa* 06 | 24171428 | 21 141 187 | | | 21 039 027 | 16 165 170 | | 16 218 | | 12 949 420 | | 80,11 | 2 052 982 | 15,85 |
| LS07 | *Leptogorgoa sarmentosa* 07 | 24392748 | 21 578 283 | | | 21 497 361 | 16 835 831 | | 15 450 | | 13 134 753 | | 78,02 | 1 316 964 | 10,03 |
| LS08 | *Leptogorgoa sarmentosa* 08 | 22433880 | 19 748 765 | | | 19 666 941 | 15 257 325 | | 12 022 | | 11 972 370 | | 78,47 | 1 338 305 | 11,18 |
| SW01 | Seawater 01 | 19043178 | 18 312 913 | | | 18 206 133 | 12 209 791 | | 8 643 | | 11 423 684 | | 93,56 | 1 683 323 | 14,74 |
| SW02 | Seawater 02 | 19957002 | 18 816 960 | | | 18 733 912 | 13 282 952 | | 8 620 | | 12 379 681 | | 93,20 | 1 999 679 | 16,15 |
| SW03 | Seawater 03 | 13681574 | 12 204 324 | | | 12 137 536 | 8 403 580 | | 3 632 | | 7 834 540 | | 93,23 | 1 090 233 | 13,92 |
| SW04 | Seawater 04 | 22667082 | 21 204 642 | | | 21 074 638 | 14 702 183 | | 10 271 | | 13 808 448 | | 93,92 | 2 443 457 | 17,70 |
| SD01 | Sediment 01 | 21994176 | 20 672 569 | | | 20 584 951 | 13 160 391 | | 6 026 | | 12 429 930 | | 94,45 | 2 569 703 | 20,67 |
| SD02 | Sediment 02 | 24688072 | 23 170 163 | | | 23 070 279 | 14 823 669 | | 5 585 | | 13 885 176 | | 93,67 | 2 695 099 | 19,41 |
| SD03 | Sediment 03 | 15917046 | 15 343 014 | | | 15 237 088 | 8 582 471 | | 2 881 | | 8 109 246 | | 94,49 | 1 608 527 | 19,84 |
|  | **SUM** | **461 178 666** | **407 108 373** | | | **405 149 354** | **291 597 021** | | **236 561** | | **252 336 988** | |  | **40 675 414** |  |
| ^1^Total number of paired end reads retrieved per sample (FastQC-analysis) | | | | |  | | |  | |  | |  |  |  |  |
| ^2^ Total number of reads per sample after merging overlapping paired end Illumina reads with SeqPrep (MGnify-pipeline v. 2.0) | | | | | | | | | |  | |  |  |  |  |
| ^3^ Total number of reads per sample after removal of reads with low quality ends and > 10% undetermined nucleotides (MGnify-pipeline v. 2.0) | | | | | | | | | | | |  |  |  |  |
| ^4^ Number of reads per sample after removal of reads with less than 100 nucleotides in length (MGnify-pipeline v. 2.0) | | | | | | | | | |  | |  |  |  |  |
| ^5^ Taxonomic analysis output of the MGnify-pipeline v. 2.0 | | | |  |  | | |  | |  | |  |  |  |  |
| ^6^Total number of predicted coding sequences CDSs, i.e. functional, protein-encoding genes (MGnify-pipeline v. 2.0) | | | | | | | | | |  | |  |  |  |  |
| ^7^Percentage of CDSs identified in all high-quality reads per sample | | | | |  | | |  | |  | |  |  |  |  |
| ^8^Total number of predicted coding sequences with InterProScan (IPR) match, i.e. annotated protein-encoding genes (MGnify-pipeline v. 2.0) | | | | | | | | | | | |  |  |  |  |
| ^9^Percentage of CDSs with IPR match, i.e. known function | | | |  |  | | |  | |  | |  |  |  |  |

**Generation of the marine sponge metagenome dataset - PRJEB13222 [2]**

Sampling of *S. officinalis* specimens (c. 10 g, n = 4), seawater (2 L, n = 3) and sediments (c. 50 g of upper 5 cm layer, n = 3) took place in May 2014 by SCUBA diving at 20 m depth off the coast of Faro, Algarve, Portugal (lat./lon.: 36.979778; -7.989111), sample sampling spot as for the octocoral metagenome dataset. Seawater samples were taken 1 m above the sponge specimens, while sediment samples were taken 1 m away from the sampled sponge specimens. For the analysis of the sponge-associated endosymbiotic community, microbial cell pellets were retrieved from 2.5 g of the inner sponge body as detailed previously [2]. Briefly, cell homogenates obtained from the samples by maceration in calcium/magnesium free sterile, artificial seawater were subjected to a differential centrifugation step as described above. Seawater samples (2 L) were passed through 0.22 mM nitrocellulose membranes which were thereafter cut into small pieces, whereas 0.25 g of sediment were retrieved from each sample after aseptic sieving (1 mm mesh) and thorough homogenization. TC-DNA was extracted with the UltraClean DNA isolation kit (MO BIO, Carlsbad, CA, United States) following the manufacturer’s instructions. TC-DNA quantity and concentration were determined using the Qubit dsDNA HS Assay Kit (Life Technologies Qubit 2.0®). Next generation TC-DNA sequencing was performed on an Illumina Hiseq 2500 device at Mr. DNA (Shallowater, TX, United States). DNA libraries were prepared for sequencing using the Nextera DNA sample preparation kit (Illumina) after the manufacturer’s instructions, and sequenced paired end for 200 cycles with sequence depth calibrated at c. 15 million 101-bp reads per sample.

| **Table AF2.2.** Number of sequence reads per quality control steps using the MGnify metagenomics pipeline (EMBL-EBI), version 2.0. (Project PRJEB13222) | | | | | |  |  |
| --- | --- | --- | --- | --- | --- | --- | --- |
| **Samples^a^** | **Raw reads^b^** | **Initial Reads^c^** | **Trimming^d^** | **Length filtering^e^** | **CDS with function** | **% reads with function** | **16SrRNA genes** |
| Sp_230 | 15752748 | 14439237 | 14354691 | 10231826 | 2303599 | 22,51405565 | 4759 |
| Sp_231 | 15905666 | 14997209 | 14882923 | 10002841 | 2275507 | 22,74860712 | 4151 |
| Sp_232 | 14212346 | 13124759 | 13055642 | 9300530 | 2027995 | 21,80515519 | 3407 |
| Sp_233 | 17124880 | 16247027 | 16128196 | 10942309 | 2446913 | 22,3619439 | 4672 |
| Sd_01 | 15144624 | 14615441 | 14489411 | 10103957 | 2056521 | 20,35361987 | 4267 |
| Sd_02 | 13139860 | 12736218 | 12628923 | 8564575 | 1808840 | 21,12002055 | 3156 |
| Sd_03 | 15930976 | 15239557 | 15127139 | 10910409 | 2199313 | 20,15793358 | 4512 |
| Sw_01 | 14690976 | 13907188 | 13786953 | 10963554 | 2426144 | 22,12917454 | 8188 |
| Sw_02 | 15262092 | 14119537 | 14008334 | 11063807 | 2298500 | 20,77494663 | 7538 |
| Sw_03 | 15330766 | 14409724 | 14278407 | 11020193 | 2312854 | 20,98741828 | 8901 |
| Totals | 152494934 | 143835897 | 142740619 | 103104001 | 22156186 | n.a. | 53551 |
| ^a^ Sp, Sponges; Sd, Sediments; Sw, Seawater. | | | |  |  |  |  |
| ^b^ Total number of high-quality, 100bp paired end reads retrieved per sample. | | | | | |  |  |
| ^c^ Total number of reads per sample after merging overlapping paired end Illumina reads with SeqPrep. | | | | | | |  |
| ^d^ Total number of reads per sample after removal of reads with low quality ends and > 10% undetermined nucleotides. | | | | | | |  |
| ^e^ Number of reads per sample after removal of reads with less than 100 nucleotides in length. | | | | | |  |  |

**Colloidal chitin preparation**

Colloidal chitin (CC) was prepared from β-chitin powder (Sigma-Aldrich/Merck) following the procedures of Hsu and Lockwood, 1975 [4] and Kuddus and Ahmad, 2013 [5] with slight modifications. Briefly, 5 g of chitin powder were carefully added to 150 mL HCl (37%) and kept for 40 min at room temperature (RT), with rigorous stirring. The solution was then placed into ice-cold distilled water (dH_2_O) for CC precipitation. CC was filtered through coarse filter paper and washed by re-suspending it in 2L of dH_2_O. The pH of this solution was thereafter adjusted to 3.5 using 10M NaOH. A sterile “CC cake” was then produced by filtering the solution through coarse filter paper using a vacuum filtration system followed by autoclavation of the retentate at 121ºC for 15 min. The sterile CC was stored at 4ºC until it was used as a substrate.

**Chitin degradation activity screening**

Chitin degradation by the target isolates was tested with a Petri dish assay on colloidal chitin (CC) agar medium containing 0.15% potassium dihydrogen phosphate (KH2PO4), 0.05 % yeast extract, 0.5% colloidal chitin and 1.5% agar, prepared with sterile artificial seawater. For details on CC preparation, see Additional file 2. The CC agar plates were divided into four quadrants where a small slot was made with the aid of a sterile micropipette tip. Each of the four slots on one CC agar plate was then inoculated with 10 μL of a liquid culture (grown until late exponential phase on MB 1:2) of the same marine bacterial strain (4 replicates per plate). For each strain, at least two CC plates were prepared and monitored, making in total 8 replicates per isolate. One CC agar plate without any inoculation was kept as negative control to monitor eventual contaminations. All plates were incubated at RT for 14 days. The whitish turbidity of the CC medium allows for visual evaluation of chitin degradation through clearing zones (haloes) around the inoculation spot. A semi-quantitative analysis of chitin-degrading activity was performed by measuring the radius of the haloes produced (see legend to **Table 1** for details).

**Endo- and exo-chitinase activity assays**

For each of the 41 strains, 200 μL from a freshly-grown liquid culture (“pre-inoculum”) were re-inoculated into 20 mL of MB 1:2, followed by RT incubation at 45 rpm on an orbital shaker (Fisher Scientific) for 96 h, until late stationary phase (monitored by measuring optical densities at 600 nm (OD600)). To prepare samples for enzyme activity measurements, 1 mL of each bacterial culture was harvested daily for four consecutive days (after 24h, 48h, 72h and 96h, respectively) and samples centrifuged for 15 min at 10,000 g. Supernatants were collected into new 1.5 mL sterile microtubes and stored at -20 oC until extracellular endo-chitinase activity determination. For intracellular exo-chitinase activity measurements, cell pellets were washed with 500 µL of Dulbecco’s Phosphate Buffered Saline (Sigma-Aldrich/Merck) and then lysed by adding 500 µL of the CelLytic™ MT Cell Lysis Reagent (Sigma-Aldrich/Merck) and shaking at 45 rpm at RT for 15 min. Suspensions were centrifuged at 10,000 g for 15 min and the intracellular protein-containing supernatants collected into 1.5ml microtubes and stored at -20 ºC until further use.

Chitinolytic enzyme activities were determined fluorometrically using the chitinase assay kit (CS0980) from Sigma-Aldrich/Merck, following the manufacturer’s instructions, and a multi-mode microplate reader (Filter Max F5, Molecular Devices). All assays were performed at pH 5 in 0.15 M citrate-phosphate (McIlvaine) assay buffer for 60 min at 37 °C in a total reaction volume of 100 μL. The reaction was stopped by the addition of 200 μL of 0.4 M sodium carbonate solution and the fluorescence of released 4-MU was measured on the microplate reader immediately after the end of the reaction, at excitation (λex) and emission (λem) wavelengths of 360 and 465 nm, respectively. Enzyme activity was expressed as the amount of 4-methylumbelliferone released per minute, where one Unit of chitinase activity corresponds to the release of 1 μmole of 4-MU from the appropriate substrate per minute, per mL.

For all readings, the released amount of 4-MU (MW=176,17 g/mol) was converted to units of chitinase activity / mL using a 4-MU standard curve. To generate this standard curve, a 4-MU standard stock solution (40 mg/mL) was diluted 100, 1,000 and 10,000 times, to prepare three standard working solutions with concentrations of 400 μg/mL, 40 μg/mL and 4 μg/mL, respectively. The 4 μg/mL and the 40 μg/mL working solutions were then used to produce the standard curve with final concentrations of 0,076 nmol/mL (4ng in 0.3mL assay vol.), 0,152 nmol/mL (8ng in 0.3mL assay vol.), 0,76 nmol/mL (40ng in 0.3mL assay vol.) and 1,52 nmol/mL (80ng in 0.3mL assay vol.), respectively. The respective fluorescence values (at 465nm) were subtracted by a standard (assay buffer) blank to produce the standard curve that was then used to quantify enzyme activity (Units/mL) in all samples. All fluorescence values of the samples were first subtracted by a substrate blank (substrate solution of 0.5mg/mL). An exo- and endo-chitinase mixture purified from the fungus *Trichoderma viride* and provided with the chitinase assay kit served as a positive control. Enzymatic activity results were also registered semi-quantitatively, whereby values ≥ 1 U/mL were scored as ++, values ≥ 0.01 and < 1 U/mL were scored as + and values < 0.01 were considered negative (**Table 1**). For each sample and substrate, the results presented correspond to the sampling day (1-4) that showed the highest enzymatic activity, respectively.

**PCR amplification of *chiA* gene fragments**

PCR amplification of *chiA* gene fragments - targeting “group A” bacterial endo-chitinases (EC 3.2.1.14) within glycoside hydrolase family 18 (GH18), based on amino acid sequences of their catalytic domains [6], was carried out on genomic DNA of each strain analysed in this study. The primer pair *chiA*_F2 / *chiA*_R2 (*chiA*_F2, 5’-CGT GGA CAT CGA CTG GGA RTW YCC-3’ and *chiA*_R2, 5’-CCC AGG CGC CGT AGA RRT CRT ARS WCA-3’) was employed, which generates amplicons of approximately 240 bp [7].

Each reaction mixture consisted of 27,76 μL ultrapure water, 5 μL of 10x reaction buffer (Bioline®), 5 μL of 2mM deoxynucleoside triphosphates (dNTPs), 3.74 μL of 50 mM MgCl_2_ (Bioline®), 4 μL of 50% acetamide, 1 μL of 10 μM of each primer, 0.5 μL of 5 U/μL Taq DNA polymerase (Bioline®), and 2 μL of template DNA in a final volume of 50 μL. For all strains, c. 20 ng of template DNA were used in the reactions. The reaction mixture was then denatured at 95ºC after which 35 thermal cycles followed with 45 sec of denaturation at 95°C, 45 sec of primer annealing at 47°C, and 90 sec of elongation at 72°C. A final extension was performed at 72ºC, for 8 min. PCR products were visualized under UV light after agarose gel electrophoresis, and amplicons of the expected size purified and subjected to Sanger sequencing as described elsewhere [8].

Partial “*chiA*” gene sequences (c. 240 bp) obtained from our bacterial isolates as explained above were used in phylogenetic tree construction as explained thoroughly in the methods section for the analysis of full-length endo-chitinase sequences. For the smaller group of short sequences described here, we did assess patterns of nucleotide heterogeneity within the bacterial groups displaying positive *chiA* PCR results, namely *Aquimaria*, *Aliivibrio*, *Enterovibrio* and *Vibrio* strains and verified whether the *chiA*-based tree topology was congruent with the taxonomic affiliation of the taxa studied (Supplementary figure S3).

**Extended results**


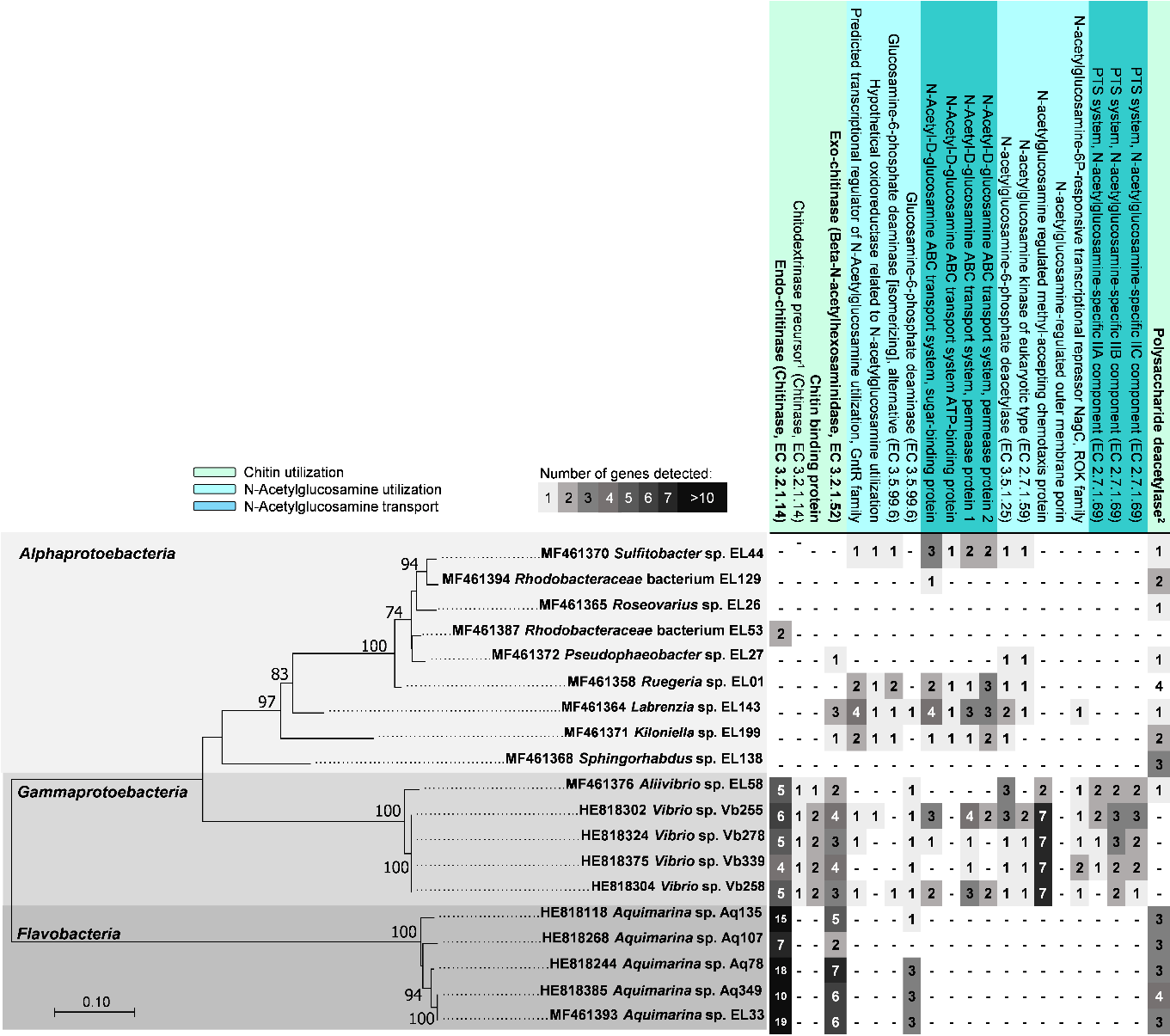


**Supplementary Figure S1. RAST annotation of chitin and chitin-derivative degradation and utilization genes in cultivated bacterial symbionts of sponges and octocorals.** For details on strains and phylogenetic tree, see legend to Figure 1. The table on the right side shows chitin degradation (including both hydrolysis and deacetylation processes) and N-acetylglucosamine transport and utilization encoding genes detected on each bacterial genome using **RAST-based classification**, in contrast with Pfam annotations show in Figure 1. Values in each cell correspond to the respective coding sequence (CDS) numbers present in each genome, whereby higher CDS numbers are highlighted in dark-grey shading. Entries highlighted in bold represent chitin processing functions examined across the sponge and octocoral metagenome datasets (Figure 3), while the phylogeny, diversity and taxonomic composition of endo-chitinase encoding genes (EC 3.2.14) are examined in Figures 2 and 4. For each functional entry, enzyme commission (EC) numbers and specific terminology are given in brackets, when appropriate. **^1^**Chitinases that hydrolyse chitin oligosaccharides - (GlcNAc)_4_ to (GlcNAc)_2_ and (GlcNAc)_5,6_ to (GlcNAc)_2_ and (GlcNAc)_3_ but are inactive towards chitin (UniProtKB P96156). **^2^**Corresponds to InterPro database entry IPR002509 (see also Figure 3) which describes the metal-dependent deacetylation of O- and N- acetylated polysaccharides such as chitin, peptidoglycan and acetylxylan.


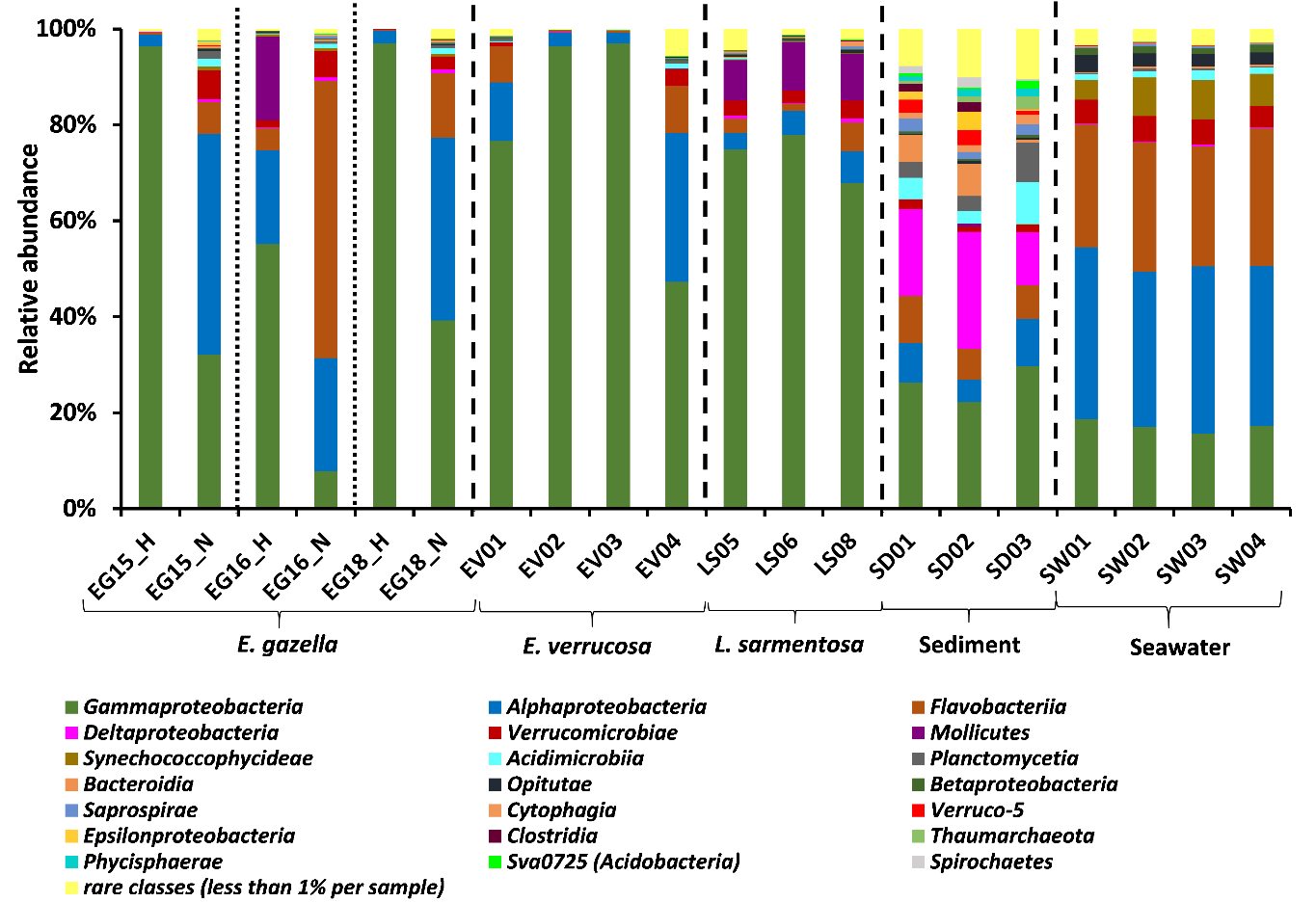


**Supplementary Figure S2.** Class-level prokaryotic community profiles of healthy (EG_H) and diseased (EG_N) *Eunicella gazella* tissue, healthy *Eunicella verrucosa* (EV01-EV04) and *Leptogorgia sarmentosa* (LS06-LS08) specimens as well as seawater (SW01-SW04) and sediment samples (SD01-SD03). Taxonomic assignments are based on 16S rRNA gene reads retrieved from unassembled metagenomes using the MGnify metagenomics pipeline version 2.0 (EMBL-EBI) for the octocoral metagenome dataset (project PRJEB13222). Relative abundances are displayed for taxa representing more than 1% of the total dataset reads. Taxa with abundances below 1% across the data are collectively labelled as “rare classes”.


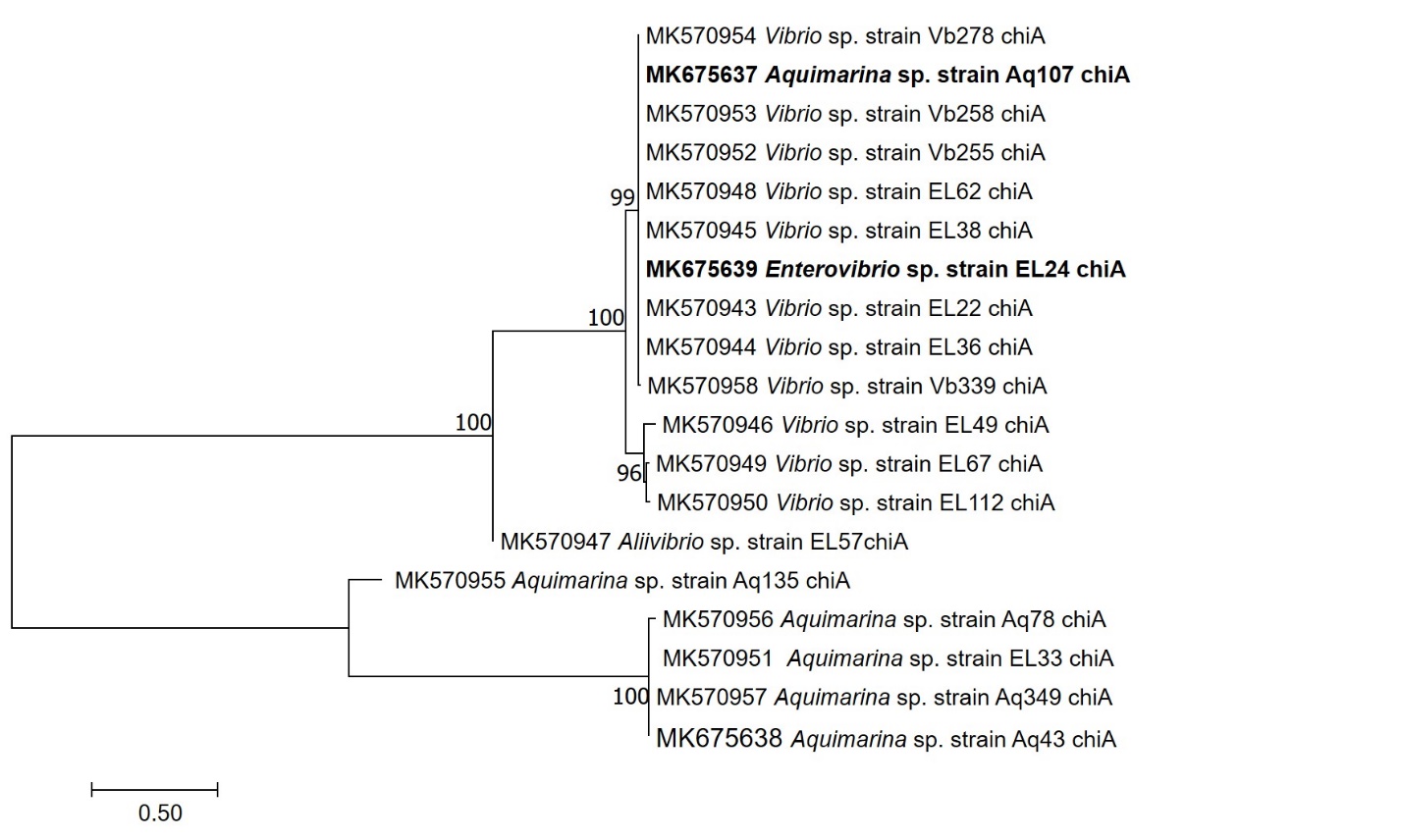


**Supplementary Figure S3** - **Maximum Likelihood phylogenetic tree of *chiA* gene amplicon sequences amplified from bacterial isolates**. Sequences were obtained for eight marine sponge and 11 octocoral-derived bacterial isolates through PCR amplification from their respective genomic DNA. The evolutionary history was inferred using the General Time Reversible model. The tree with the highest log likelihood (-892.58) is shown. The percentage of trees in which the associated taxa clustered together is shown next to the branches (1,000 bootstrap replicates). A discrete Gamma distribution was used to model evolutionary rate differences among sites (5 categories (+*G*, parameter = 3.1129)). The rate variation model allowed for some sites to be evolutionarily invariable ([+*I*], 19.83% sites). The tree is drawn to scale, with branch lengths measured in the number of substitutions per site. The analysis involved 19 nucleotide sequences. Codon positions included were 1st+2nd+3rd+Noncoding. All positions containing gaps and missing data were eliminated. There was a total of 164 positions in the final dataset.

Phylogenetic inference of *chiA* gene fragments amplified from the isolates resulted in a tree topology mostly in agreement with the taxonomic position of the organisms, except for *Aquimarina* Aq107 and *Enterovibrio* EL24 whose sequences showed high homology with those from *Vibrio* spp.

**Extended discussion**

**Chitin degradation capacities revealed for cultivatable symbionts of sponges and octocorals**

In agreement with our results, earlier surveys based on agar-plate assays have documented chitin degradation abilities by *Vibrio* [9-11], *Aquimarina* [12, 13], *Shewanella* [14], *Microbulbifer* [15] and *Pseudoalteromonas* [16] isolated from other sources. Yet no previous records of chitin degradation ability have been found for *Enterovibrio* spp., registered here as one of the strongest *in vitro* degraders of colloidal chitin. Moreover, except for *Vibrio* species, current knowledge of chitin catabolism and its genetic determinants in various marine bacteria remains limited. Here, we provide firm experimental evidence for, and solidify the status of, less-studied genera such as *Aquimarina*, *Microbulbifer*, *Pseudoalteromonas*, *Shewanella* and *Enterovibrio* as chitin consumers with putative relevance for carbon and nitrogen cycling in association with sessile marine invertebrates.

***Aquimarina* species possess distinct genomic signatures for chitin metabolism**

Among the abovementioned taxa, the *Aquimarina* strains were distinct regarding several phenotypic and genotypic parameters evaluated. These organisms consistently presented high chitinase gene copy numbers and heterogeneity per genome, comparable to those usually observed for *Vibrio* spp. Besides the high number of endo-chitinase encoding genes and the existence of exo-chitinase and glucosamine-6-phosphate deaminase-encoding genes observed for the cultivated *Aquimarina* strains, their genomes lacked the common PTS- or ABC-transport systems for chitin monomers, dimers and / or oligomers. An alternative mode for chitin binding and transport of chitin derivatives to the known proteins described for *Proteobacteria* is most likely to exist in this *Bacteroidetes* genus. Genomic studies of *Flavobacteriaceae* species have shown that many so-called Polysaccharide Utilization Loci (PULs) are typically involved in cell surface polysaccharide capture and hydrolysis [17]. The most studied PUL-encoded polysaccharide uptake system is the Starch Uptake System (Sus) encoded in the Sus operon [17, 18]. The Sus is to date unique to the Gram-negative *Bacteroidetes* phylum and various analogues of the system exist that seem to target numerous, diverse polysaccharides, while PTS- or ABC-transport systems seem to be widely absent in this taxonomic group [17, 18]. We recently found several genes of the Sus operon on all *Aquimarina* genomes described to date [19], and an *in-silico* analysis of the genomes of the five *Aquimarina* strains of this study on RAST confirmed the presence of the SusA, SusC and SusD genes in all of them except SusA in *Aquimarina* sp. strain Aq135. Moreover, the *Flavobacteriaceae* species Flavobacterium johnsoniae specifically deploys a chitin utilization locus (ChiUL) that consists of eleven contiguous genes encoding for surface glycan-binding proteins, porins, regulatory proteins, enzymes (including a multi-domain *ChiA*-type chitinase), and a two-component sensor–regulator system to efficiently metabolize chitin. Homologous genes of that system are present on the genome of *Aquimarina macrocephali* type strain DSM 22623 [20], which is a species that is phylogenetically close to the here studied strains *Aquimarina* sp. Aq135 and Aq78. The type IX secretion system (T9SS), common to members of the *Bacteroidetes* phylum, is required for chitinase [21] secretion and is simultaneously involved in gliding motility, whereby mutations in *gld* genes are known to lead to deficient chitin utilization [22]. We have recently shown that the full genetic machinery required for gliding motility among the *Flavobacteriaceae* (genes *gldA* through *gldN*) as well as the presence of several proteins containing T9SS C-terminal domains is a remarkable feature of the core *Aquimarina* genome [19].

**Phylogeny and potential horizontal gene transfer of endo-chitinase encoding genes**

Previous studies [23-26] suggest that chitinase-encoding genes are subjected to horizontal gene transfer, but to this date no actual documentation of their spread across members of coral or sponge microbiomes has been made. From the phylogenetic inference performed here for full-length endo-chitinase encoding genes from culturable symbionts of sponges and octocorals, although we did find divergent bacterial taxa belonging to the same endochitinase gene clusters, their low levels of homology prevent us to hypothesize that these could be deemed as signals of recent horizontal gene transfer (HGT) events. Yet close inspection of short, *chiA* gene fragments amplified from our cultures (Figure S2) suggest that HGT could explain the high homology observed for a few pairs of sequences from disparate taxa. Curiously, the genome of the temperate phage Smp131 of *Stenotrophomonas maltophilia* encodes for a lysin belonging to family 19 chitinases which can be usually found in plants and certain bacterial taxa [23]. Also, the temperate Sinorhizobium bacteriophage ФLM21 genome encodes for a lytic enzyme which is a putative chitinase (COG3179) [24]. This hints at a possible role of bacteriophages in the transferability of polysaccharide degrading genes within microbial communities. Future studies of the sponge and coral holobiont shall focus on the gene content that usually traffics about on plasmids and phages (“the metamobilome”) to enlighten our knowledge of the functional traits of mobile genetic elements in marine host-associated settings. Indeed, a recent study revealed that sponge viromes mediate the traffic of ankyrin repeats across sponge microbiome members, thereby mediating patterns of phagocytosis evasion by symbionts within this system [27] and illustrating the potential of modern virome-based studies in sharpening our understanding of complex host-microbiome interactions.

**Supplementary Dataset S1:** Coding sequences containing endo-chitinase catalytic domains analysed in **Figure 2**

> fig|6666666.195556.peg.218 Chitinase (EC 3.2.1.14) [Aquimarina sp. EL33 clean (20 contigs)]

atgtttaaaaaacacacaaactcagtaaattttattaaaattaattgttcatggagacag

ttaatttgtacgaccttatttctaacagtaattctatctgtttttccccatggcacagta

acataccctccaagtcgtgtttggaattgttttcaggaagatcctgaaagtccagattct

gcggcttgtgttgcagcagtagcctctcatggcacacagccattgtatgattggagtgaa

attaatcaagcgaatgctaatggtaatcatcagcaatatgtgatggatggaaatctggct

agcggcggcaggcccgaaaaatacggaggcatggatcaagttagatctgattgggtgtcg

acaaaagtttctccagggccatttacagttacttggacaaatcatgctcctcatgcaact

gcttattatgaagtttatataaccaaagcaagctggacaccggatcagccattaacgtgg

gatagccttgaacttttagtacgaacacctccaagtggagcagaaagaattgttaatatt

ccggtaacacttcctgttaggacaggtaaacatgttatttatagtgtttggcaaagatca

gacagtccagaagcattttattcgactagtgacatcaattttgatggagggggtactgat

acacaggcaccttctgttcctactggtttaggggcatcaaatgctacacaaactacagta

gatcttgcctggaatgctgctaccgataatgtagctgtaactggatatgatatataccag

ggaaatactattgttgctaccgcaactggtacttctcatcaagtaactggattaacagca

aacacctcgtattcttttagaataaaagcaaaggatgctgcaaataatcaatctggtttt

agtaatactgcaactgcaactacgcttgctgatactggaggaggaaactgtacaggaata

ccacaatatgtagcaggtacttcttatagcaaagatcaggaagtacaaaatgaaggagaa

aaatttacatgtaatattccaggatggtgctcttctgctgcggcttgggcatatgcacct

ggtactggtgcccactggcaggatgcctggtcaaaaacaggagattgtaccggtggtact

cctaatacgagtcctactataagtattacttctccaagtgataatagctcgtttcaagaa

ggagcgtctgtaacaatgtctgctagtgcagcagatacagatggtacagtatctaaagtt

gaattttataacggtagtacaaaattaggagaagatacttctagtccttatgaatatgtt

tggcaaaatgtttctgcaggaaattatgcaataacagccaaagcaaccgataaccaagga

gcatcgaccatatcttcagtaataaatattaccgttaatggagttaataatactcctcca

acagttrcgattacttcaccaaataataatgactcttttaacgaaggaacttcaatctct

rttacggcaaacgcttctgatagtgacgggactattactaaagtagaattctttaacgga

accacaaaactgggagargatactagtagtccttatgcatatacaatttctaacgcatct

gtaggaaattatacattgacagctaaggctactgataatggaggagctacttcaacttct

tctgcgatatcaatttcggtgactacagtaggaaatggtaattgtgatgggttaccacaa

tatgttgcaggaacttcatatagtaaagatcaggaagtacaaaatgaaggagagaagttt

aaatgtaatattccggggtggtgttcttctgcggcagcatgggcatatgcaccaggtact

ggagctcattggcaatcagcttggtcaaagacaggtgattgtggaggaggaacaggaggt

gctcctgtagtaaatatcacatctccttcaaatggtgcaacatatactgctggtagttct

gttgttgttaatgcaaccgcaaccgatgatggaaccgttactaaagtagaattctttaat

ggaactacaaaactaggagaagatactactaatccgtattcttatacaattaccaacgca

caatctggtagttattctttaactgcagtagcaaccgataatgagaataatcaaactact

tctgatccagttgtaattagaagcagtacaggaggaggaaataataatttaccaggaaaa

atactggtagggtactggcataattttgataatggttctactacaccaagattgagcgag

gtatccagagattgggatgtgatttgtgttgcatttgcagaacctaaagcgggaagtaca

gccgatatgctatttagtccatatagtatttataatgggaatactcaggcttttatagat

gatgttgctactgtaaagagtagaggacaaaaagtactaatttctattggaggtgctaat

gcaagagtagaattgaataatgaaactgaaaagaatttattcgtaagttctatgaccaat

ataatcaatacatatggtttcaatggattggatatagatttagaaggaagctccctttca

ctaggtagcggagattctgatttcaggaatccaactacagcaaagatcaaaaatcttatt

gctgctacaaaagccattagaaccaatataggtgcaaatagatttatattaagtatggct

ccagaaacggcgtatgtacaaggtgcatatgggaattattctggaatttttggagcatat

ttacctgttattcatgcactacgtaatgaaatggattatattcatgtacagcactataat

acaggttctatgtttggaggagatggaaaaatataccagccagccactgcagattttcat

gtggctatggcagaaatgttaattactggtttcccagttgcacaaactggacttacattt

cctggacttagagcagatcaggttgctattggattacctgctactactcaagcagcagga

agcggatatacttctgaagctgttgtacaacaagcactggattatttgattaaaggaacc

tcttatccaggaagaacgtatacaactagttctacatatcctagttttagaggtttaatg

acctggtctattaattgggatttggtaaataattctaccttctcttcaagccatagagca

tatttggatggattaggtgccagagctgctaatgcacaaaatagtgtagggaaagtattt

cctaatcctatttctggaaatataattaatgttgccttagatggttctatttctaaatca

ggttctgattatttcagattccagatatttaatactaatggaattgaagtttataatttt

cagaatgacaggcttcaaagaggagaaagtgttaagagttttgatatcggtgaactagaa

tcgggaatgtatttttatacgatttcagtttccaaaaacaaaacaacaggtaaaataatt

agagagtaa

>fig|6666666.195556.peg.219 Chitinase (EC 3.2.1.14) [Aquimarina sp. EL33 clean (20 contigs)]

atgtgcgtttcaagcattgcttttgcacaagtgaacacgggaggaagtgctaccacttct

gatcatcaaaaacagattatagggtatattaccaactgggacgcctggaaaaccaatact

gcaggagtacctgaagccggagctcttacacatttaaatattgattattctaaatacacg

attctaaattattcattttttggagtggctcgtgatggttctttacatagtggcgatcat

agaaataagaatatatatcaagatggtgtaacacaagaacctgctgatttattttataca

gatttgtatagtagttgggatttacacctcttattcggggagttagaatatgtaaactat

gtaaacgctgatataaaagcacgggcagaagctcaaggctttcaagtagaagtaggagct

agtacatggacacatcctgtctggggactaagcggaggattaccattacctcttaaaaaa

gaaaatggagctctggggttactagatcttgcacatcaaaaaggagtaaaagtaatggcc

tctataggaggatggagtatgtgtaaacatttcccagaaatggctgccgatcctgtaaaa

agagcaaaatttattgaagactgtaaaagattaatcaatattggtttcgacggaattgat

ttagactgggaatatccaggaccattttcaggaatgaattttacaggtagtcaggcagat

tttgcgaattttgaatctttactacaagaaattcgtaatgctataggacctacaaaatta

attacatcggctatggctgcagatccaagaaaactggatggatttaattggtctggagta

gttgctaatatggattactttaacatgatgacttatgattataatggtggttggtctaat

aaagcaggacataatgcaccagtatatccgtatacaggagcagaagtacctttctttaac

tggcaatctactttacaaaaacttgttgaagcaggagttcctaaaaacaaaatatgtttt

ggagccccattctacggtaggggtgtggtaacagaaggaactgctgatcttaattcaaca

acagtaaaacgatcagaaactgtacaaccagatggaccaatacaaaccgctgcagattat

acaaattggccgaaagaagtttatgatggtactcctaattacttttttatcaaacaaaaa

gccttatctcctaatagtggttggaccagaaaatgggataatgaagctaaggttccttat

ttggtaaatggtaaatattttttaagctatgatgatgaagagtctattgctattaaagct

cagtttattaatgataatgagcttgccgggactattatttggacagtttatggtgatctt

gaatttggaggtacagcaacttcttttggtagaaaacttaaaagatggtctaatgtaaaa

tctcctttggtgaataaaatcaatgaagtttttgcaaatggagggccaggaggaaatgtt

tctcctacagttaatataacagcaccagctaataattctacttttgctgaaggcgcaaca

atagctataacagctaatgcatctgatagtgatggaactattgctaaagttgaattctat

aatggaaccacaaaattaggagaggacactacatcaccatatgagtattcgtggtctaat

gttcctgccgggagttatacaataacggcaagagcaacagataatggtaatgctagcacg

acttcttctgccgtttctgtatctgttggtaatagtacaaatacaccgccaacagtaaat

attacttcacctaataataacgattcgtttacagcgggagtatcaatagctataactgct

aatgcatctgatagtgatggaacgatctctaaagttgaattctataacggaaccataaaa

ttaggagaagataccagcagcccttatgggtatacaatttctaatgcatcagcaggtgat

tatacattgactgcaaaagcaacagataatgagaatgcaacgacaacttccactactatt

tctataacggttactagtgatacaggaggatgtaccaatataccacaatatgtagcaggt

acttcttatagcaaagatcaggaagtgcaaaacgaaggtggaaaatttacatgtaatgtt

ccgggatggtgttcttctgcagcagcttgggcatatgcaccaggtactggagttcactgg

caagatgcgtggtcaaaaacaggagattgtagtggcggtactcctaatacgagtcctact

gtaagtattacttctccaagcgataatagctcgtttcaagaaggagcctctgtaacaata

tctgctaatgcagcagatgcagatggtacagtatctaaagttgaattttataatggtagt

acaaaattaggagaagatacttctagcccttacgaatatgtttggcaaaatgtttctgca

ggaaattatgcaataacagtcaaagcaaccgataatcagggtgcatcgaccacatcttct

gtaataaatattaccgttaatggagttaataatactcctccaacagttgcgattacttca

ccaaataataatgactcttttaacgaaggaacttcaatctctattacggcaaacgcttct

gatagtgacgggactattactaaagtagaattctttaacggaaccacaaaactgggagaa

gatactagtagtccttatgcatatacaatttctaacgcatctgtaggaaattatacattg

acagctaaggctactgataatggaggagctacttcaacttcttctgcgatatcaatttcg

gtgactacagtaggaaatggtaattgtgaaggattaccacaatatgctgcagggacttca

tatagcaaagatcaggaagtgcaaaatggaggcgaaaagtttaaatgtaatgttccagga

tggtgctcttcagcagcagcatgggcttatgcacctggtacaggagctcattggcaatta

gcttggtcaaagacaggagactgtacagtaagaaattctacggttagcgtatttccgaat

cctactgaaaatggtatcataaatgtgatgataaattctggaaagtctaactctaaattt

agatttgaagtacattcccttaacggaactaaattattagattttgaaaataatgtgatg

aatgggcagaatggtaaaacatttgatattagttctctaaaaactggtttgtacttatat

acgattactataggaaaagaaaaagaatatggtaaaatgaaagtatcaaattaa

>fig|6666666.195556.peg.221 Chitinase (EC 3.2.1.14) [Aquimarina sp. EL33 clean (20 contigs)]

atgaaaaatttttatacatttttatttatgtttttttgtgcgctattgatgcacgcacaa

tacaattttcctacctgctctgcagagtgggatgcaagtaaagtcccttataaacaagga

caagaagtttcttataataatgttaattataagtgcaaatactatacaaatgatgctccg

ggagccggctcctgggaactaataggaccatgtggtgatggaggattaggtccggattat

tctggtaaacaacgaattatagggtatctgcctacctgggttgctgattatgatatcaaa

aataaatttaatccagaggtagtaacacatcttaatatttcttttttgatgtttaagcaa

aataataacgattataatagttcaaattttgcttctatatcttttgatgagtttcaatct

agaaaagtagattctgttcttaacgatttgggagttcttcaaaaagctaaagccaaagga

gtaaaagtatctgttgcattaggaggagctaccgattatgcatttctatggttaatgacc

aaatatcaaaacaatgatagcaaactagatgaaattgcaacattgattgctaattatgtt

actcaaaatgatctggatggtgttgatttggatatggaatgttggtgggcagacccggcc

attagtgggacttcagaccagggaggaagagtaagaggaagcaaatggggagatgctgat

aaaggtcctcaccccgcgggtattgggttaacaaaattaagccagaaattaagagccaaa

atacctaataagttaattacagctgctgtttttggaacttcatggtatggaaataactat

gatgatggtatggcagaccatatggattggattggtcttatgtcttatgattttacagga

tcatgggataaatccccagaagggcctcattcttctctttataaagtagaactagggact

tatcaaggacaaacagcagataatccaatttattctgcacaagatgcattagaatattgg

atgggatttgctccaccagcctggaatcatgcaggaggatttaatgtaccaaaagctaaa

ttagctttcggattaccagtgtatggttatgatttttctgaaaaaaagcctgatggaggg

aatggagctaaatttgttccttataaggatatcataaaagattttgctaatgcagctaca

agttatgaccctaaggatcctaaaaaattaagaggatacataggagaaaatggaaaaaaa

atatactataacacaccaaaattagctgcagaaaaaattaaatattctaagcagtatggg

catcagggattaattatatgggaacttacacaagataccgattataattcgtcttcaagt

attcttaaagcagtaaatgaggctgcaggaaacactgatcctataaacaattcaccaaca

gtagtgtgggaagctcccacaaatggacaagttatcgaattggaagaattgtctccaata

acattaaaagccagcgctacagattcagatgggactattcaatcttttgtgtttaagcac

aatacgactaacattagtgctacagcaaatggaagtagctatacagccagttttactcct

gcagcatttggtgaagtaacgctcattgcttctgctacagataataaaaatgcaatttca

gaaaagacaattgtttttacagtaaagaagaaagtagtgggaggtaatacacctccatcg

ataactctaatcgaaccaaaaaatgcagatgttatagaacaaacagcattgtcatcaatt

cagttaaaagctacagtaacagatgatacccaggtaagctctgttaaatttgttgtaaat

aatattgaaattactccaactgtaaatgggactcaatatgttacagactggactcccagc

gcttttggtgaagtatcctataaaattatgggtacagataatgaaggactatctacagag

gtggttgttacgtttactgtgaaagaaaaagtagtgggaggaagttgtgatggaatagca

gaatggcaatctgataaggtatatgctacagcaggacaaaaagtaagttataacgggaat

ctctataccaataaatggtggacgaaaggggagactccgggaagtagctctgtttgggaa

tttgtttctagttgtaatggtggttctggtggcggagatttttgtggatctccacaatgg

ataacatctattgcttataatagcggggatcaagtataccatagccaaaaaatctataaa

gccaaatggtggacagaaggtgaaactcctggcagtagttctgtgtgggagtttgtttca

gactgtgttcagagtaatccaaatatgtcttctgttgcatttcaaacgttggtagatgat

gttataaaatatcagataacagtttctgaagtatcatggataaaaattgatgtatatgat

atctatggtaaattaatgcacacaaaatctttaagagaatatacaggaagtagagatttt

acacaagatttatcgactcttaaaagtggaatctatatttacaaagtaaatataggcgga

gaagtaatcaccaaaaaagtaattaaaaaataa

>fig|6666666.195556.peg.222 Chitinase (EC 3.2.1.14) [Aquimarina sp. EL33 clean (20 contigs)]

atgacacacaaactcaccactccaaaaaaccaaaaggttttaggtaggaaatcatggatt

atgatttttactgtattactttttactattcacctaaccttttctaacggtgataataca

tatcccgagatatcaaaaacagttgattctttctctctggataaacctttacctgtaaat

ccactatatcaagttaatgatttgataaataaagatttatggaacactttgtttccgtat

cgttttggtgccaaagacacaggaggtggtgtttgggtactagacccaaaagatgatttt

tatactttcgaatcttttatcgaggccattaatagaatgagcaaaatcgaagtaaccttt

gatagaagatgtggtacgaacgcttatagagttacacgtactgataagactacaggtgta

tcaaaagtgattcgaaccgatgtagattttgatgcaccaagaaatgctgataaagaaatt

gttaccaaaaaagtagattatggatcttttttaggagaaggaagtctcgagaccagaaaa

agagaaattacagctttttttgctaatatctctcacgagactaccggggggtggtcaact

gctccaggtggacagttttcttggggtttacactttcgtgaagaacctacagatgcttct

tatgcttctccagatacaaattatcctccaactccggggaaatcatataaaggaagaggc

cccatacagttatcatacaattataattatggccctgccagtgaattcatttttggagat

aagcaaatacttttggatcatccggaaaaagtaatcgaagatgctgctttggcttttcag

actgctatttggttttggatgacgcctcaatatcccaaaccatcagctcatgatgtaatg

gtgaacaaatggactcctaatgagttagacaaaacaaaaaatagaattccgggattaggg

atgactgtaaatattattaatggaggcgtagaatgtggacaaggtacagaaaaacctcag

gtgttagatagaataggatactacgaaaggtttacgggtatttaccaaatagggacagat

atggatggagttcatgatctttctgattgtggatgtaaagatatgtctaagtatggggga

gactctgctgatttaaccgcagaaccctgtgcacaaaaacctcaggtaacatttaccaat

cccagaaataatcaaatgtttgagcaatctacattttctccaatttctgtaagtttatct

atagacgaaaaaaatacaaaactagtaagtgttactaccgctatagggaatcaaactttt

gatggagtaacatttagttggacaccatctagctatgcaagccatgttttaagcgctaat

gcggtattcgaaaatggtataacggcaacatcagaaattaagattattatttgggatgga

gtaaatcttgactgccaggaagtgcccgaatggaatgcttcaaggatttataaggacaag

aataactatgtgaaatataacaataatgtttataaaaataaatggtatgccgatagtagt

aatgtcccgggaagcgatactgtatgggaatttgtaaaagaatgcggtgtttctaatggg

agtagtcctgtgataacttgggaatcgccaggtaatggacaagttatagaacaaaatgaa

cttgcaccgattactttaaaagccagtgctactgataccgatggaactgttcaatctttt

gtctttaagtataataatgctaatattactcctactgcttcaggagatatctataccgct

agttttacgccaactgcatttggagaaattactattacagcatctgccacagatgatcaa

aataatacctcagaaaaagcaatttcttttactgtcaaagaaaaaacaacaggaggaaat

aataaacctcccgttgttagtataacttctccgggagataatgcctcttttgaagaaggc

acttcgattcctgtcacggtaaatgcatctgatagcgacggcaccatcacgaaagtagaa

ttctttaataataatagtaaaataggagaaagtagcgctagcccttttagttatacactc

gaaaacgttatagtaggaaattactcgttaacggcaaaagcaacagacaataaaggggca

ttttcaacctcttcagcaatagcaattacagtaactagtggcggaggaaatgggaattgt

gcagatatacaacagtatgttgcaggtacttcttatggattaaatgatgaggtagtaaat

gaaggagaaaaatttagttgtgatattccgggatggtgttcatctactgcagcatgggca

tatgcccccggtacaggagcgcattggcaaatggcctggacaaaaattggagcttgtggt

aaaaataattctgaaataacttcaaataagtactctgtttttcctacggtaacccaggat

attgtaaactttagaataaaaacagataatacatcatgggttaagattaacctatatcat

ctttctggaaaattgataagcacacaatcatttaatggaatccagacaaagacactaaaa

tcatttacacatgatctgtcaaatctcaaaaatggtctttatgtatttaaaatatatata

aatgacgatgtttattttgagaaaatccttaaaaattaa

>fig|6666666.195556.peg.234 Chitinase (EC 3.2.1.14) [Aquimarina sp. EL33 clean (20 contigs)]

atgaaattacttaaaagattatcagtcgtcttactcatacaggtattcttattttcctgt

acgaatgaaagttttttagaagaaggagccgcagaggttcaaaattctgaaacaaatcta

acagacaaaacagcgctcacagaaccaatagtcttaggatatttcccttcctggtccgaa

agctgggctggccctgggcaaggatcaaaactaagagatatcccagaacacatcactcac

gtttttttggcctttgcaaagcctaatcttcggtatcaaaaaggctctttagatattacc

aatacaggtattcaaactccgtacggaggggatactttaaaagaatctgttgcagctcta

aaatcaaaaggaatcaaagttattctatctgtaggtggagaaacgtactggggtacagat

gcagcttatgatatcaattatcaacagatcaaagatcttgtagatgatatgggattcgaa

ggtatcgattgggattttgaacctaatggtagttttgccactataggtgatcccataaat

gtacaacgctttatcgatttctttacaaattcgagagctattatgcctaaaggacaatat

ctattagcttgtgctcctgccggtgttggagccttaggtggtgcaaataatgatgacccc

tcctctccttttgcctatagcaaaagaaacacagtaacaggagaatctgatactaatctc

tttaatgcaacttctcctaatcaagccattagtttatttggttttgctactaccgggcat

atgatccctgtaatggaggctgttggtgacaaaattgatcttattgcatatcaaggatat

aatacaggagctgccagtaatagaaaaattatgtatgatgcttataaacattattcaagt

cagtatgggttttctattgcagccggtgttcattatcctaacgagccttggggaccatat

tatgaatatacttatcaaaacttagcagagctatctaaccatattgctgtaaataacacc

tctaatgatggagttatgatatggcagttattactgggtaatacgacttcatcggcatat

ggatatcttcatgtcgcgagccaggtactaaatggcacatcacaatctcaagctatagct

aatgcagaaaactatcccgaatcaccatacactggtgatggaggcggaggcggaggatcg

ggctgcgaatctgctccatggaatatttctgttacttataacactggtcaggaggttgtg

cataataataaattatatcgcgcaaaatggtggacccaaaaagataatccatcatcaaat

tctggagacggattaccatgggaattcgttcaggattgtaatggtaacgggggaggaaac

aatcaatctccatctgtatctattacatctcctacaaataatcaatctataaccgaaggg

caatctatcaatattacagcaaatgcttctgatgctgatggaacaattacaaaggttgaa

ttttttcaaggaactactaaactcggagaagacacaacaagcccgtatagctattcctgg

aataatgctcctgtaggaaattatactttaagtgctattgccacagataatgctagtgca

acaactacatcggctacagtatctatcaccattacttcttctggtggtggtaacggtggt

tcttgtagtggtattgcaaattgggaagcatatccaacagtatataatgtgggagacaaa

gtagtatatcaaggtacattatacgaagctcaatcagggccaatatgggtaaccccaggt

agtggagaacactggtggaaaaccataggtacctgtaattaa

>fig|6666666.195556.peg.4616 Chitinase (EC 3.2.1.14) [Aquimarina sp. EL33 clean (20 contigs)]

atgaaattaaaattactcttaattccattcgccttttcctgccttttagggactcagaat

gggtttactcaatccgtatttattaatgaaatacattacgacaatgcaagtacagatgta

gaagaagctgttgaaattgctggaacagcaggaacagatctttctggttggagtattgtt

ttgtataacggttcaaatagtaccgtttataatacaatttctatttcgggagtgattccc

gatcaacaaaatggtttcggaacggtaatagaaattcttcctaccaatggattacaaaat

ggtgctcccgacgggatcgcattaattgataacaacaatgcagttgtacagtttttaagc

tatgaaggcgttattacagcaacaaatggtcctgcatctggattaacaagtaccgatatc

ggtgtgtcagaatctagtagtactcccgttggagcttctttacagttatcaggaacagga

acatcggcaacagaatttacatgggaaatatctacaaccaactcatatggtgccgtaaat

attaatcaggttttgggaacaccagttatcatacccatgattaatgagtttgtatgtaat

cataccggatcagatacagatgaatttgtagaaatattagctggtatagaaacagactta

agcgaatattggttgctagaaattgaaggagatagtaatgcatctggagttgttgacgaa

gtaatacaattaggttctacagatgttaatgggtattttactactgcttttggtagtaat

gtatacgaaaacggtacagtcactctcctcttggtaaagaattttacgggtagcttaggg

caagatcttgataccgatgacgatggtgtttttgatatcactccctgggaagaacgtatt

gatgatgttggagttaatgatggtggtgcatctgatcttaattatgctaatgttacctta

ctacaatcttttgatggaagctcatttactgtaggaggagcatcaagatttccgaatggt

caagataccgatactgtcaccgattggaaaaggaatgattttgatggaagtggattgcca

agttttcctacagttatagcagaaccaggagaagctgttaatacgcctaatagagaaaat

gtagttatcgatgatactaatccaacagcggtaatagtaattaatgaaattgatgcagat

accgatggatctgatgttttagaatttgtagagctgtttgatggtggagccggaaacaca

tctctggatggatacacattagtattctttaacggatctaataatcagagttatgctgcc

tatgatttaacaggttctaccacaaatactaatggatattttgttatcggtaatgcagat

gttgctaatgttgggattacatttcctggtaatgggttacaaaatggagccgatgccgta

gcactttataaaactgcagcctctaattttcctaacggaagtgctgttacaacagaaaat

ctggtagatgcaattgtatacgataccaatgatagtgatgatgcagaactattggtgtta

ttaaatagtggtgagtcacaaataaatgaagacgagaaaaatgataagaacttccattcc

ttacaacgttttcctaatggatctggaggattaagaaatacctcaacatatacacaggca

attcctacaccaggaagtgcaaatacaaatgcaacagaaatagttaaccttattatcaac

gagttggatgcagatactcaaggttctgatgctttagaatttgttgaactctatgatggt

ggagccggaaatacctcacttaacggttacgtattggtaaattataacggaagtaataac

accagttataatgccattgatttagatggttttactaccaatgcagaaggatattttgtt

attggtaatgcagatgttactaatgtaggccttgttgttcccggaaatacattccagaat

ggtgctgatgctgtagtcttatactttggtgatgcgacaagtttccctaacggaacagcg

gttactaccgaaaacattattgatgctattgtatatgataccgatgatgcggatgatgta

gaattattggtacttctaaatacagatcagccacaggtaaacgaaaactcaaacggaaac

aaagatggagaatccttacaacgaagtcctaatggacaaggaggggctcgaaatactact

tcatatgtagccaaagctccaacaccaggcgcagataacgatggtgtaatagtaattcct

ggtgacccaataagcattgcagaagctagagctactgccgagggtacaccagttactatt

gccggagtattaaccgttaccgatagttttaatggtcctgcatttatacaagataccact

ggtggtatcgctgtttttgatgaccaggtacaagctaatgctactttaaaagttggggat

tctattaccataacaggtatcagggctgcttttaatgatcagattcagattagttctgta

acagatgtagtaaataatggtcttcctcaaaacccaatcacaccactagacattacactt

gcagaacttgcagatcatccaggagaattggttcgagtacttaatactacattccctaat

ccgggagatcttttattcggtaactctaacttcactcttaccgatgctagtggaaatgga

gaactacgtgttgataacgacgtagcttctatagtaggtaaggcacaacctgttacttgc

acagaaatcacaggtgtgataggtcgttttagagaattcttccagttactgccaaggcaa

gtttcagatattccttgtgcggtagaatttattcctccaggagacacagtaggttttcct

aaagaagatacttttgatgtagtaacctggaatatcgaatggtttggtgatgaaaacaac

tcgcctgtaggtcaaaaccctatgtctgatgcaattcaaagagatagtaccgctacagta

cttaaaaagctaaaagcagatgtatatgccgtagaagaaattgccgatgatgtattgttt

gaagaattagtaaacctgttaccaggttatgagtatattttatctgatgctgtttctcgc

cctggttctggaggagtatcacaaaaagtaggatttatttataatactgaaaccgtttct

gtggtcgaaacaagagcaatgtttacatctattcatccactatacaacggtggtgatgca

tctgcgcttgtagattatcctagcgaaacagatcgtttctacgctagtggaagattgcct

tttttaatgaccgcagatgtaactattaatggagttacagaacgtattgatttgatcgca

ttacatgccagagcaaatagtagcaatggtcctcaaaatcgatatgatatgcgtaaatat

gatgtagaagtattaaaagattcgctagatgctaattttgcaaacaacaaggttattctt

ttgggagattataatgacgatgtagatgaaaccgtagcagacatcccatcaaccatttct

agttttcaagaatatgtagatgacactactaattacacgattgtatcttctgcattgagc

gaagcaggattgcgatcattcgtttttagagaaaatatgattgatcatattatggtaacc

aatgaacttaatgaggcctatatcgagaattctgtaactgttcattacgacgtatatgat

aatgattatgcattcactacatcagatcacttgcctgtttctgcgcgattcttgctagaa

ccagaatttgtgaacaatgattgttcgggtgcatctgtagtagcctttaatcaaggcaag

agaaaagatggtggaagaatatcccgacttagaagcaagactaagcgtgccctaggaact

cccagagaaaaaaggtattttaattttgtgagtttaggttttggtggatcaatcaccata

gaactaaacaacgaaatttttgataatgctgataccaatgaattcgcagtattcgaatct

acagggttctttgataatatcccttgtaattactatccagaatcggcagaagtatttgct

tcacaagatggtattgagtttgtttctttaggaacaacttgccaggatggagaatttgat

cttgcaacaggtaatttacgttctgcaaaatatatcaaggttgtggatacaagtgataaa

gctaattttccttggtttgcagatggctatgatctagatgctattgtatgtcttgagaat

ggtgagcgtatcgctacaaaaaataccatggtctatgcagaaaaccccactgctctagaa

agtgagttactcaccaaagatttcgggttagaagaaacaagtatttttgtttctccaaac

ccatttaaaaatcagttatctatagattttaaaactctggttgaaggagatgtagatatc

acaattaccgatgtaacaggtaaaacggtatatactcaaacgatacaacttaatgtcgga

caatctaaattatcaatcgatatgaatcgttatacaaaaggattttatgtagtacatgct

tctagtacaaatggaaaacttaatataacccaaaagataattaagaaataa

>fig|6666666.339721.peg.707 Chitinase (EC 3.2.1.14) [Vibrio sp. Vb339]

atgttaaaaatcaaatatttggcgacagtactgggctgcacattagcagcgcaaagtcat

gcgtctttgaacattcaacctgatccgcaaaatccgaatggttaccttgttgaaaagtcg

gccttacaagctgctgaacaagcgaaaacatccgatcctatgtatgctatctggtcacag

gcccttcaaactcgcccgaacaccatcgttgaagcgattgaacctggctccccctcgaat

cctgaaaacgtaaagcgtgtagagcgcgtgttccctcaatctgaatgggacttcctcact

cagatggcagcgccagaatacacctacactcgcttcttacgtgcaattggcaaatttcca

gctttctgtggagagtacaccgatggccgtgactccgacgccatctgtaaaaaatccatt

atcacagcctttgctcatttctcacaagagacgggtgggcacatcgcgatagacaacacc

tctgataatccattagctctcgaagagtggcagcaagcgctggtgcatgttcgtgaaatg

ggttggtctgaaggccaagaaggttacaccacaggttgtggtcagaatgattggcagaat

gcccgctggccatgtgctgcggggcagggttactttggacgtggtgctaaacagctttct

taccattttaattacggcgcgttatctgaagtgatgtttgatggtgatgcgactgtgctt

ctcaacaatccgggcttagtggcggactcttggttgaacttggcttctgctatctggttt

ttccttacccctcaagcccctaaaccagccatgttgcatgttattgaccgtacttggacg

ccatctcaacgcgaattggatgcaggtattggttatggatttggtaccacgatcaacgtg

atcaatggtggtattgaatgtggcgagcagaacaaagacaaaggccaacccgttaaccgt

attcgttactgggaagggctagcggcgcactacgaaattccggtagaagcggatgaagcc

aatacgtgttggcagcaaacgccttacggaagcttaaacctcaacggagcaacggatgtg

ttgtacaccaactgggatggcaactggaaatactacgctgaccgcccagaaggttactca

tttgagtgtgagcttgttggtttccaaacggcttattctgcgctggtggctggagattac

gagaagtgtgtgaccaacttttatggttctcatgcgagttggcctgaagtgaaagtagtc

gataagctggatccggtagaccctggcactgatccgggtggtaatggttggagtgcgacc

aaggtttacaatgcgggtgaccaagtgactcataatggcgcaacctacgaagcgaaatgg

tggacacaaggggataaccctgccaatggcggcccttggaaattgattgctggagagcct

acaccgccagtcgtcaccgatcctacacctgttgatcctgctccggtagacccaacacca

gttgaaccgccagttactgagccgcctgtcgttgttgacccatcggtgtttatcacatgg

caagcgggcgttagccaagtgagtaacggtgataaggtgacacataacggtaaatgcttc

gtggctaaaaacggcccgggtgtatgggaaagccctattcagtcgaattggttctgggat

gaaatcagttgtaattga

>fig|6666666.339721.peg.1356 Chitinase (EC 3.2.1.14) [Vibrio sp. Vb339]

atgattcgtataaatacctgtgctgcaagcattgctctagcgctatctggtacagcatta

gctgctccaacagcacctagcatcgacatgtacggttccaataacctgcaattttctaaa

attgaactcgcgatggaaaccacctctggctacaaccagatggtgaaatatcacgaccaa

gcgaaggtcgacgtcaagttcaaccaatggagcggaacgtcaggaaacacgtacaacatc

tactttgatggcgttaaagtagccaccggcccaatcactggcagccaaaccacggcctca

ttcgaatacggccaaggcggcttattcgatatggaaattgaagcgtgtgacgaaactggt

tgtagcaagagcgcacccgctaaaatcaccatcgcagataccgatggctcccacttagcg

ccacttgcgatgaatgtggatccaaacaacaagtcatacaacacagatccaaacacggta

gtaggtacttactttgttgaatggggtatttatggtcgtgactacacggtagataaccta

cccgctgacaacttgacccatatcctttatggcttcatcccaatttgtggtccgaacgaa

tcagttaaatcagtaggtggtaacagctacaacgcactcatgacggcatgtcagggcgtt

aacgattacgaggtggtgatccatgacccttgggcggctttccagaagagcttccctcaa

gcgggtcacgaatacagctcaccgatcaagggtaactacgcaatgatgatggcgctcaaa

cagcgtaatcctgatttgaagatcatcccatcaattggtggctggacattatctgacccg

ttctttgattttacaaccaaagccaaccgtgacaccttcgttgcatctgttaaaaaattc

ctaaacacatggaaattctacgatggtgtagatatcgactgggaataccaaggtggtggc

ggtgctgcgccagagcttggtgacccagaaaatgatggcccagcctacattgcactgatg

gcagaattacgtgcgatgctcgatgagctagaagcagaaaatagccgtacttacgagcta

acatctgcgataggtgttggccacgataaaattgaagacgtgaactacggcgatgctatc

caatacatggattacatctttgcgatgacttacgacttctacggcggttggaacaacgta

ttaggtcaccaagcagcattacactgcggtaacttcatgcgccctggtcagtgtgatggc

tcaggcattgatgaaaatggcaaaccatacacaggcccagcttacacaacggacaacggc

atccaattgctgctcgaacaaggcgttccagctaacaagcttgtcgtaggtgcagcaatg

tacggtcgtggttgggaaggtgtattaccctcatcactttcggatcctagcgaccctatg

acaggtgttggtaacggcaaactgaaaggtagctctgcgcaaggcgtctgggaagatggc

gttatcgactacaaaggcattaaggcgaacatgcttggtgcgaataaccaaggtatcaat

ggcttcgaatacggttacgacgaaatggcagaagcgccttacgtatggaaccgcacctca

ggtcagctaatcacatttgatgatgaccgctcggtaaaagcaaaaggcgcgtacgttcgt

agtcttggtcttgcaggtctattctcttgggaaattgatgcagataacggcgatatccta

aatgcaatgcatgaaggtctagcaggtggcaccaccgatcctgtaaaccgtaaaccaact

gcagcagcaggtgccgaccaatccgttgaaggcccagcttctgtttctctagatggcagc

gcttcaaaagacagcgacggtacgattgcgagctatgcttggtcacaagtaagcggcacg

gcagtaacgctggcgaacgcgactgcggctgttgcaagcttcgatgtggttgaagtcgcg

caacaagaagcactcacgttcagcctaacgataactgataacgaaggcgctactgcaact

gacaccgttgttgtaacggtaactcctaaagacacaggcccagtcaatacagcaccagtt

gctgtggttacggctccggctgaagttaatgcgggtgacgtagtcgtggttgatgcttcg

gcttcgagcgatgctgaccaagacacattgaccttcacgtgggatatgcctgctggtatt

gatgcaacgattcaaggttcttcagtgagctttgtcgcggcggaatacacgcaagatacg

acgctgaacttctctgtaacggtcagcgatggcactgatacatcggtagcggctacttca

gtgaacgttcttaagaaaaccacaggcggcggtacgtgtactaacgcttgggattcaagt

gcggtctacactggcggcgaccaagtaactcaaggcggtaaggtttgggaagcgaaatgg

tggacaaccggtgaagatccaacaacgacaggccaatggggcgtgtggaaagagatcggt

cctgcaagctgctaa

>AY772396.1 Vibrio sp. CJ11027 chitinase (chiK) gene, complete cds

ATGATTCGATTTAACCTATGTGCAGCTGGGGTTGCCCTAGCATTATCTGGCGCGGCAAACGCGGCTCCTA

CCGCACCAAGTATCGATATGTACGGTTCCAACAACCTGCAATTTTCTAAAATTGAGTTGGCAATGGAAAC

CACATCTGGCTACAACGACATGGTTAAGTACCATGAACTAGCCAAGATCAACGTGAAATTCAACCAGTGG

AGCGGCACATCTGGCGACACTTACAACATCTATTTTGATGGTGTTCAGGTTGCAACAGGCCCTATCACTG

GTAGCCAAACCACAGCGTCGTTTGAGTACGGCCAAGGTGGTTTGTACCAAATGGAAATCGAAGCGTGTGA

TGCAACAGGCTGTGCCAAGAGTGCACCTGTAGAGATTACTATCGCAGATACCGATGGTTCTCACTTACCA

CCTCTAACCATGAATGTTGACCCGAACAACAAAACCTACAACACAGACCCAAGTGTCGTGATGGGTACGT

ATTTTGTTGAATGGGGCATCTACGGCCGTGATTACACTGTCGACAACATGCCAGTCGACAACCTAACGCA

CATCCTTTACGGCTTCATTCCAATTTGTGGTCCAAACGAATCGGTGAAATCAGTKGGCGGTAACAGCTTC

AACGCWCTGCAAACAGCGTGTCGYGGTGTGAACGACTACGAAGTGGTAATCCATGACCCATGGGCTGCGT

ACCAAAAGAGCTTCGCGCAAGCAGGCCATGAGTACAGCACGCCGATCAAGGGTAACTACGCAATGCTAAT

GGCGTTGAAACAACGTAACCCTGATCTAAAAATCATCCCATCTATCGGTGGTTGGACACTTTCTGACCCA

TTCTACGACTTCGTTGATAAAGCCAACCGTGACACGTTTGTCGCGTCAGTTAAGAAATTCCTGAAAACGT

GGAAGTTCTACGACGGTGTGGACATCGACTGGGAATTCCCTGGTGGTGGCGGCGCTGCAGCTGATAAGGG

CGACCCAGTAAACGATGGCCCAGCATACATCGCATTGATGCGTGAACTGCGTGCAATGCTTGATGAACTA

GAAGCAGAAACTGGCCGTACTTACGAGCTAACTTCAGCAATCGGTGTGGGTTACGACAAAATTGAAGACG

TTGATTACGCAGACGCAGTTCAGTACATGGACTACATCTTCGCGATGACATACGACTTCTACGGCGGTTG

GAACAACGTTCCTGGTCACCAAACAGCACTTTACTGTGGTTCATTTATGCGCCCTGGTCAGTGTGACGGC

AGCGGCGTTGATGAAAACGGCGAACCGTACAAAGGCCCAGCTTACACGGCTGACAACGGCATCCAACTAC

TACTAGCGCAAGGCGTTCCTGCGAACAAACTTGTTCTAGGTACAGCAATGTACGGCCGTGGTTGGGAAGG

CGTAACACCGGATACGCTAACCGATCCAAATGACCCAATGACGGGTACTGCAACAGGTAAACCGAAAGGC

AGTACAGCTCAAGGTGTTTGGGAAGATGGCGTGATTGACTACAAAGGCATTAAGTCATTCATGCTAGGTG

CGAACAATACAGGTATCAACGGCTTTGAATATGGCTACGATGCTCAAGCAGAAGCACCTTGGGTGTGGAA

CCGTTCGACTGGTGAGCTGATCACATTTGATGACCACCGCTCTGTATTGGCAAAAGGTAGCTACGCGAAA

TCTCTAGGTCTAGCAGGTCTATTCTCTTGGGAAATTGACGCAGATAACGGCGACATCCTGAATGCAATGC

ACGAAGGTATGGCAGGCAGCGTAGTACCTCAGCCAAACCATAAACCAACGGCTGCAGCAGGTGCAGACCA

AGCAGTAACAGGTCCTGCAAGTGTTGTACTTGATGGCAGCAACTCAACAGACTCTGATGGCACAATCGCA

AGCTACGCTTGGGAACAAGTATCTGGTACTGCAGTGACTCTATCTGGCGCGAACGCAGCGACAGCAAGCT

TCGATGTAGCAGAAGTTGCCGTTGAGGAACAGCTAACGTTCAAGCTAACTGTCACTGATAACGAAGGTGC

AACCGCTTCTGATCTTGTTGTAGTAACGGTTAAGCCAGCAGGCGTGATTGATCCACCAAACACTGCGCCA

GTTGCACAGGTTTCTGCACCAGCAACAGCAAACGCAGGTGACGTGGTTGTGATTGACGCTTCAGCTTCTA

GCGATGCAGACAACGACACGCTAACGTTCGATTGGACGCTACCTCAAGGTCTAAACGCAACGGTTAACGG

TGCAAAAGTGACGTTCACAGCAGCAGAATACCCACAAGACACTGGCCTAAGCTTCACTGTAAGCGTAAGC

GACGGTCAAGCGGCTTCTTCTGCAAGCGCAAGCGTTGTTGTTGCAAAACACACCACAGGCGGCGGTACAT

GTACTAACGCTTGGGATGCAGCAGCGGTTTACACTGGCGGTGACCAAGTGACACATGCTGGTAAGACTTG

GGAAGCGAAATGGTGGGACTCAAGGGCCAAGATCCTAGCCAATCTGGCGAATGGGGTGTTTGGAAAGAAG

TTGGTCCAGCTAACCGTAACTAAACACTGCTAA

>AB299855.1 Vibrio parahaemolyticus Chi1 gene for chitinase, complete cds

ATGATTCGATTTAACCTATGTGCAGCTGGGGTTGCTTTAGCGCTATCAGGTGCTGCAGTCGCAGCTCCGA

CCGCACCAAGTGTCGATATGTACGGTTCTAATAACCTTCAATTTTCTAAAATTGAACTGGCTATGGAAAC

CACATCTGGCTACTACGACATGGTGAAATACCATGACCAAGCTAAAATCACCGTTAAATTCAACCAGTGG

AGCGGTACACCTGGCGACACTTACAACATCTATTTTGATGGCGTAAAAGTCGCAACAGGCGCTATCACTG

GCGGCCAAACCACAGCAACATTTGATTACGGCCAAGGCGGCTTGTACCAAATGGAAATTGAAGCGTGTGA

TGCTACAGGCTGTTCGAAAAGTGCTCCTGCAGAAATCACCATCGCGGATACAGATGGCTCTCACTTGAAG

CCACTAACGATGAACGTTGATCCAAACAACAAAACATACAACACCGACCCTAGCGTCGTGATGGGTACGT

ACTTTGTTGAATGGGGCATCTATGGTCGTAACTACACCGTAGACAACATGCCAGCAGACAACTTAACTCA

CATCCTTTACGGCTTTATTCCAATCTGTGGTCCAAACGAATCTGTTAAGTCTGTTGGTGGTAACAGCTTT

AACGCTCTTCAAACTGCATGTCGCGGTGTGCCTGATTACGAAGTGGTAATCCATGACCCATGGGCCGCTT

ACCAGAAGAGCTTCCCGCAAGCTGGCCACGAGTACAGCACGCCTATCAAGGGTAACTACGCAATGCTAAT

GGCATTGAAACAACGTAACCCTGATCTAAAAATCATCCCATCTATCGGTGGCTGGACACTGTCTGACCCA

TTCTACGATTTCGTTAATAAAGCGAACCGCGACACATTCGTGGCATCGGTTAAGAAATTCCTGAAAACTT

GGAAATTCTACGATGGTGTAGATATTGACTGGGAATTCCCTGGTGGCGGCGGTGCTGCAGCTGATAAAGG

CGACCCAGTAAACGATGGTCCAGCTTACGTTGCATTGATGCGTGAACTGCGCGCAATGCTAGATGAGCTA

GAAGCAGAAACTGGCCGTACTTACGAGCTAACTTCAGCAATTGGTGTCGGCTACGACAAGATTGAAGACG

TAAACTATGCAGATGCCGTTCAATACATGGACTACATCTTCGCGATGACTTACGACTTCTACGGCGGCTG

GAACAACGTCCCTGGTCACCAAACAGCACTTTACTGTGGTTCGTTCATGCGCCCTGGTCAATGTGACGGC

AGCGGCGTAGATGAAAACGGCGAAGCTTACAAAGGCCCTGCGTACACAGCAGATAACGGTATCCAACTTC

TTCTTGCTCAAGGCGTTCCTGCGAACAAGCTAGTCCTTGGTACTGCAATGTACGGTCGTGGCTGGGAAGG

CGTAACACCAGATACACTCACTGATCCAAATGACCCAATGACAGGTACTGCGACAGGTAAACTGAAAGGC

AGCACTGCACAAGGTGTTTGGGAAGATGGCGTAATCGACTACAAAGGCATTAAGTCATTCATGCTAGGTG

CAAACAACACTGGCATTAACGGCTTCGAGTACGGCTACGATGCACAAGCTGAAGCACCATGGGTATGGAA

CCGTACAACGGGTGAGTTGATCACATTTGATGACCATCGTTCAGTGCTAGCAAAAGGTAGCTACGCGAAA

TCTCTAGGTCTTGCGGGTCTATTCTCTTGGGAAATCGACGCGGATAACGGCGACATTCTCAACGCAATGC

ACGAAGGTATGGCAGGCGGTGTGGTTCCTCAACCAAACCGTAAACCAACAGCCGCAGCTGGCGCAGATCA

ATCGGTAACTGGCCCTGCAAGTGTGGTTCTAGATGGTAGCCTATCGAAAGACTCTGACGGCACTATCGCG

AGTTACGCGTGGGAACAAGTATCTGGCACGGCAGTGGTATTAGCAGGTGCAAACACAGCGAAAGCAAGCT

TTGATGCAGCAGAAGTTACTGTTGAAGAGCAATTAACCTTCAAGCTGACAGTGACTGACAACGAAGGTGC

AACGGCATCTGACCTGGTTGTAGTAACTGTGAAGCCAGCAGGTGTGGTTGACCCAGTGAACAACGCACCA

GTAGCGCAAGTTGTTGCTCCAGCAACAGCAAATGCTGGTGACGTTGTGGTTGTAGATGCATCTGCGTCTA

GCGATGCAGACAACGATACTCTTACTTTCAACTGGACTCTGCCTCAAGGTCTGAACGCAACGGTAAACGG

CTCTAAAGTGACGTTCACTGCTGCGGAATACCCGCAAGACACAAGCCTAAGTTTCACTGTAAGTGTGAGC

GACGGTAAAGCGTCTTCATCGGCAAGCGCGACAGTGGTTGTTGCACAGCACACAACCGATCCAAACCCAG

GTACTTGTGACAACGCATGGGATGCGAGCGCAGTGTACACAGGTGGCAACCAAGTGACTCACGCAGGTAA

AACGTGGGAAGCGAAATGGTGGACTCAGGGTGACGATCCAAGCAAGTCTGGCGAATGGGGTGTATGGAAA

GAAGTTGGCGTAGCTAACTGTAACTAA

>AF323180.1 Vibrio carchariae chitinase A precursor (chiA) gene, complete cds

ATGATTCGATTTAACCTATGTGCAGCTGGGGTTGCCCTAGCACTATCTGGCGCTGCAAACGCAGCTCCAA

CCGCACCAAGTATCGATATGTACGGTTCCAATAACCTTCAATTTTCTAAAATTGAATTGGCAATGGAAAC

CACATCTGGCTACAACGACATGGTCAAATACCATGAACTGGCTAAGATCAAAGTGAAATTTAACCAGTGG

AGTGGCACATCTGGCGACACTTACAACGTCTACTTTGACGGTGTAAAAGTGGCAACAGGCGCTATCACTG

GCAGTCAAACCACAGCTTCGTTTGAATATGGTCAAGGCGGCTTGTACCAAATGGAAATCGAAGCGTGTGA

CGCAACAGGTTGTTCTAAGAGCGCTCCGGTAGAAATTACCATTGCAGATACAGACGGCTCACACTTGAAG

CCTCTGACGATGAATGTTGATCCGAACAACAAGAGCTACAACACCGATCCAAGTATCGTGATGGGTACTT

ATTTTGTTGAATGGGGCATCTACGGTCGTGATTACACTGTCGACAACATGCCAGTTGATAACCTAACTCA

CATCCTTTACGGCTTTATCCCAATTTGTGGTCCAAACGAATCAGTAAAATCAGTTGGTGGTAACAGCTTT

AATGCACTGCAAACGGCATGTCGTGGTGTGAATGATTACGAAGTGGTTATCCATGACCCGTGGGCAGCTT

ATCAGAAGAGCTTCCCTCAAGCAGGTCATGAATACAGCACGCCAATCAAGGGTAACTACGCAATGCTAAT

GGCGTTGAAACAACGTAACCCGGATCTAAAAATTATCCCATCTATCGGTGGTTGGACACTTTCTGACCCA

TTCTACGACTTCGTTGATAAGAAGAATCGTGACACGTTTGTCGCGTCAGTTAAGAAATTCCTGAAAACTT

GGAAATTTTACGACGGCGTAGATATTGACTGGGAATTCCCTGGTGGCGGCGGCGCTGCAGCAGATAAGGG

TGACCCTGTAAACGATGGTCCTGCATACATTGCATTGATGCGTGAACTACGTGTAATGCTAGATGAACTT

GAAGCAGAAACAGGTCGTACTTACGAGCTAACTTCAGCAATCGGTGTTGGTTACGACAAGATTGAAGACG

TAGATTACGCAGACGCGGTTCAGTACATGGACTACATCTTTGCGATGACTTACGACTTCTACGGCGGCTG

GAACAACGTTCCTGGTCACCAAACTGCTCTTTACTGTGGCTCATTCATGCGTCCTGGTCAGTGTGATGGC

GGCGGCGTGGATGAAAACGGCGAACCGTACAAAGGTCCAGCATACACTGCAGATAACGGTATCCAGCTTC

TTCTAGCGCAAGGTGTTCCTGCAAATAAACTGGTTCTTGGTACAGCGATGTATGGTCGTGGTTGGGAAGG

TGTAACACCTGATACGCTAACAGATCCAAATGACCCAATGACCGGTACTGCAACAGGCAAACTGAAAGGC

AGCACAGCTCAAGGTGTTTGGGAAGATGGCGTAATCGACTACAAAGGTATTAAGTCATTTATGCTAGGTG

CGAACAACACTGGCATCAACGGCTTTGAATACGGCTATGATGCGCAAGCAGAAGCACCTTGGGTGTGGAA

CCGTTCGACTGGTGAGCTAATCACATTTGACGATCATCGTTCTGTGTTAGCGAAAGGCAACTACGCAAAA

TCTCTAGGTCTAGCAGGTCTATTCTCTTGGGAGATTGATGCAGATAACGGCGACATCCTTAATGCAATGC

ACGAAGGTATGGCTGGCGGCGTCGTTACCCCACCAAACCGTAAACCAACGGCAGCAGCAGGCGCAGACCA

AGCAGTAACAGGTCCTGCAAGCGTTGTGCTTGATGGCAGCAACTCAACTGACTCTGACGGTACAATCGCA

AGTTACGCTTGGGAGCAAGTATCAGGTACTGCAGTCGTTCTATCTGGCGCGAACACAGCAACAGCAAGCT

TTGATGCGGTAGAAGTCGCAGCTGAAGAGCAACTAACGTTTAAGCTGACCGTAACAGATAACGAAGGCGC

AACGGCTTCTGATCTTGTTGTAGTCACCGTTAAGCCAGCAGGCGTGATTGATCCACCAAACACAGCGCCA

GTTGCACAGGTTTCTGCACCAGCAACGGCAAGCGCTGGTGACGTGGTTGTGATTGATGCTTCAGCTTCTA

GCGATGCTGACAACGACACACTAACGTTCGATTGGACGCTACCACAAGGTCTAAATGCTACGGTCAACGG

TGCGAAAGTAACGTTCACAGCAGCAGAATACCCACAAGACACAAGCCTAAGCTTCACGGTAAGCGTAAGT

GATGGTCAAGCAGTATCAACTGCAAGTGCAACCGTTGTTGTAGCGAAGCACAGCACAGGCGGTGGTACTT

GTACTAACGCATGGGATGCAACGGCAGTTTACACTGGTGGTGATCAAGTGACTTACGCAGGTAAAACTTG

GGAAGCGAAATGGTGGGACTCAAGGGTCAAGATCCTAGCAAGTCTGGCGAATGGGGTGTTTGGAAAGAAG

TTGGTCCATCTAACTGTAACTAAACACTGCTAA

>AF323471.1 Vibrio parahaemolyticus chitinase A (chiA) gene, complete cds

ATGATTCGATTTAACCTATGTGCAGCTGGGGTTGCTTTAGCGCTATCAGGTGCTGCAGTCGCAGCTCCGA

CCGCACCAAGTGTCGATATGTACGGTTCTAATAACCTTCAATTTTCTAAAATTGAACTGGCTATGGAAAC

CACATCTGGCTACTACGACATGGTGAAATACCATGACCAAGCTAAAATCACCGTTAAATTCAACCAGTGG

AGCGGTACACCTGGCGACACTTACAACATCTATTTTGATGGCGAAAAAGTCGCAACAGGCGCTATCACTG

GCGGCCAAACCACAGCAACATTTGATTACGGCCAAGGCGGCTTGTACCAAATGGAAATTGAAGCGTGTGA

TGCTACAGGCTGTTCGAAAAGTGCTCCTGCAGAAATCACCATCGCGGATACAGATGGCTCTCACTTGAAG

CCACTAACGATGAACGTTGATCCAAACAACAAAACATACAACACCGACCCTAGCGTCGTGATGGGTACGT

ACTTTGTTGAATGGGGCATCTATGGTCGTAACTACACCGTAGACAACATGCCAGCCGACAACTTAACTCA

CATCCTTTACGGCTTTATTCCAATCTGTGGTCCAAACGAATCTGTTAAGTCTGTTGGTGGTAACAGCTTT

AACGCTCTTCAAACTGCATGTCGCGGTGTGCCTGATTACGAAGTGGTAATCCATGACCCATGGGCCGCTT

ACCAGAAGAGCTTCCCACAAGCTGGCCACGAGTACAGCACGCCTATCAAGGGTAACTACGCAATGCTAAT

GGCATTGAAACAACGTAACCCTGATCTAAAAATCATCCCATCTATCGGTGGCTGGACACTGTCTGACCCA

TTCTACGATTTCGTTAATAAAGCGAACCGCGACACATTCGTGGCATCGGTTAAGAAATTCCTGAAAACTT

GGAAATTCTACGATGGTGTAGATATTGACTGGGAATTCCCTGGTGGCGGCGGTGCTGCAGCAGATAAAGG

CGACCCAGTAAACGATGGTCCAGCTTACGTTGCATTGATGCGTGAACTGCGCGCAATGCTAGATGAGCTA

GAAGCAGAAACTGGCCGTACTTACGAGCTAACTTCAGCAATTGGTGTCGGCTACGACAAGATTGAAGACG

TAAACTACGCAGATGCCGTTCAATACATGGACTACATCTTCGCGATGACTTACGACTTCTACGGAGGCTG

GAACAACGTTCCTGGTCACCAAACAGCACTTTACTGTGGTTCGTTCATGCGCCCTGGTCAATGTGACGGC

AGCGGCGTAGATGAAAACGGCGAAGCTTACAAAGGCCCTGCGTACACAGCAGATAACGGTATCCAACTTC

TTCTTGCTCAAGGCGTTCCTGCGAACAAGCTAGTCCTTGGTACTGCAATGTATGGCCGTGGCTGGGAAGG

CGTACACCAAGATACGCTCACTGATCCAAATGACCCAATGACAGGTACTGCGACAGGTAAACTGAAAGGC

AGCACTGCACAAGGTGTTTGGGAAGATGGCGTAATCGACTACAAAGGCATTAAGTCATTCATGCTAGGTG

CAAACAACACTGGCATTAACGGCTTCGAGTACGGCTACGATGCACAAGCTGAAGCACCATGGGTATGGAA

CCGTACAACGGGTGAGTTGATCACATTTGATGACCATCGCTCAGTGCTAGCAAAAGGTAGCTACGCGAAA

TCTCTAGGTCTTGCGGGTCTATTCTCTTGGGAAATCGACGCGGATAACGGCGACATTCTCAACGCAATGC

ACGAAGGTATGGCTGGCGGTGTGGTTCCTCAACCAAACCGTAAACCAACCGCAGCAGCTGGCGCAGATCA

ATCGGTAACTGGCCCTGCAAGTGTGTGTTCTAGATGGTGA

>fig|6666666.339721.peg.2087 Chitinase (EC 3.2.1.14) [Vibrio sp. Vb339]

atgaaactgtcatttatcacacacgctatttctagcaacagtgaccaatcaagttctatc

tttaaatctaattctatcattgcgcttagcttaatagtcacggcttgtgtgctcacacct

acagttcatgccgatgataaagtcgtcgcagggtattttgcggattggcaatatctgaat

aaagataacccttataccgttgacgacataccggcagacaagctctcacatattatttac

gctttcttgagcatgtgtggtcctcatagcggtgctggtgagcccgtccaaaaacaaatt

cagcagcaatgtgcagacaaacaaccatataccgcgattattgtggataaagaagctgcg

ttagaagtcgatttcggagacgtgggtgttgatgtcgcctataaaggtcactttgcccag

ttagctcaattgaaggccgacaatcaaaatatcaaaatcttgccatcatttggtgggtgg

acaatgtctgaacctttccatgcgatggcgaaagatcctaaagccattgcgcacttttca

aaaactgctgttgagttaatccaaaaatatgacttctttgatggtattgatcttgattgg

gaatacccaggaggcggcggcttaaccacttcgccttggaacccagagactaaactaacg

gatgagcagaaagcgcaagagcgtgaagctttcacacttttagtaaaaacaatcagaagt

gatttggacgctttatcaaagacgactcaaagagattatgagctttcaacggctgtcggt

gttggcgctaaggcagcccaaattgattggaacgcagctagcccatatctgacgaacatg

tttgcgatgacgtatgacttcctcggaggttggggagctcaaacaggtcacacaacaaac

ttacacgccacagagcgcagttggtggggaatgggagctgacgtcttcattaatcagatg

atagagcaaggtattcctagcgagaagctcgtgatcggggcggctttttatggtcgaggc

tggcaagggacaaaagactttgctggagagctgcctaaaggtgacctagtttctgaacaa

ggggcgcagtttggtaccggtgaaaacggttactttatgttctgggaccttatgaataac

tacagcagcaaacaaggttatgagtacaattatgacgagcaagctcaggcaccttatttg

tggaacccagataaaaaagtattcatatcttttgaagatcagcgttctattaaagcaaag

gcagaatgggcgaagaaatcagacctaggcggcatttttacttgggaattatcaggagat

ccttccggacagttgatcgatgttatgcatagtgaaatgaataaataa

>KM596659.1 Vibrio tapetis clone 12854355 putative chitinase gene, complete cds

ATGAGAAAAGCGTTAATTACCGCAGCCGTCGTCGGAGCCATTAGTGCTCCTGCGACGGCCGCTGAAAAAG

TGGTCGCTGGTTACTTTGCCGACTGGCAATACGAAAACCAAGAAAACCCTTATACCGTTCAAGATATTCC

CGCAGACAATCTCACTCACATTATTTACGCATTTTTAAGCATGTGTGGCCCACATAAAGGGGCATCTGAG

CAAGTTCAAAAGCAGGTAGACGCTCAATGTGAGGGCAAGGCTCCGTATACTGCCATCGTTGTTGATACAA

AGTCGGCGCTGGTGCACGACTTTGGCAAGGTTAATGTTGATGTCCCGTACAAAGGCCATTTCGCACAACT

AGCTCAGCTGAAACAAGATCACCCTAAAATAAAAGTGCTGCCTTCGTTTGGCGGATGGACGATGTCGGAA

CCATTTCATGCAATGGCAAAAGACGATGCAGCGATCCGTCACTTTTCTAAAACAGCGGTAGAGTTGATTG

CTCAATACGACTTCTTCGATGGTATAGATTTGGATTGGGAGTACCCTGGCGGTGGAGGGCTGACGACATC

ACCTTGGAACCCTGACACCAAAGTGAATGACGAACAAAAAGCATCAGAACGAGAAGCCTTCACTTTATTA

GTAAAAACCTTACGTAATGATCTCGACGTATTATCCAAAAAAACAGCGCGTGACTATGAACTCTCGACTG

CGGTCGGCGTTGGACCTAAAGCGGCTCAAATTGACTGGAAAAATGCGGCTCCTTATCTAACTAATATGTT

CGCAATGACCTATGACTTTCTAGGAGGCTGGGGAAGTCAAACCGGTCACTTAACGAATTTGCACGCGACA

GAGCGCAGTTGGTGGGGGATGGGAGCGGATGTGTTCATCAACCAGATGATAGAGCAGGGTATCCCTAGTG

AGAAACTGGTGCTGGGAGCGGCATTTTACGGACGTGGCTGGCAAGGCACCGAGAACTTTAATGGTCAGTT

ACCAACATCGGATCTCGTTTCTAAGCAGGGCGCACAATTTGGAACCGGAGAGAATGGTTACTTTATGTTC

TGGGATCTAATGAGAAACTATGGGGAAAGCCAAGGCTACCAATACAAATACGATAAACAAGCGCATGCCC

CTTTTCTGTGGAACGAGGACAAAAAGGTATTCATTTCATTTGAAGATGCACAATCGATTAAAGCTAAAGT

GAAATGGGCTAAAGATGCGAATCTTGGGGGAATATTTTCCTGGGAATTATCAGGAGATCCTAGTGGTGAG

TTAACGGAAACCATGTATAAAGAGATGACTCGAGACTAG

>fig|6666666.339721.peg.3132 Chitinase (EC 3.2.1.14) [Vibrio sp. Vb339]

atgttgaaacgtaaagctctacaattagctgtatcggtcggcatggccgcaatgtctggc

gctgtttatgcgaatggttcagatatgacgaatccagattctggtgtcgtggtcggttat

tggcacaactggtgtgacggtggcggctaccaaggtggtaatgcgccatgcgtgacgctg

gatgaagtaaacccaatgtataacatcgtgaatgtgtcattcatgaaggtttacgatgtg

gctgatggtcgaatcccaacttttaaactggacccaaccgttgggttatcagaagaacaa

tttattgaccaaattgccgagctcaacaagcaaggccgttctgtactgatagcgctgggt

ggtgctgatgcacacgtagaacttgaaacgggtgatgaaagagcctttgctgatgagata

atccgccttactgagcgttacggtttcgatggtctagatatcgaccttgaacaagcagca

gtaaccgcagcaaacaaccagacggttattcctgacgcacttaagctagtaaaagatcac

taccgtgccgaagggaaaaacttccttattactatggcgcctgagttcccgtatctaacg

acaggcggtaagtacgttccttatatcgataatctagaaggttattacgactggattaac

ccacagttctacaaccaaggtggcgacggtatctgggttgacggtgtgggctggattgct

caaaacaacgatgcattaaaagaagaattcatctactacatctctgattcgcttatcaac

ggaacgcgtggcttccacaagatccctcatgacaagctggtgtttggtattccttcaagt

atcgatgctgcagcgacgggctttgtgcaagaccctcaagatttatacgacgcgttcgag

accttaacgactcaaggccaacccctgcgtggtgtaatgacttggtctatcaactgggac

atgggcactaacaaaaacggtcaacaatacaacgagcagtttattaaagactacggccca

tttgtacacggtcaagtgacgccacctccagtcgagggtgagccagttcttaaaggtgtc

gaaaatacgcgtgttcttcacggcacaacatttgatcctatggaaggcgtaacagcaacc

gataaagaagatggcgatctaacatcttctgtcgatgttgaaggttacgtggaaaccagt

gtgattggcacttacgttttgacttaccgagtgaaagacagcgataacaacgaaaccact

aaagcgagaacggtagaggtttacagccagaagccagtatttaatggtgtgtctgacaca

acagttgtgttgggtgctgcgtttgatgctatggcaggcgtgacggcgaatgatgctgaa

gatggcgacctaacaaataccatcacacatactggtagtgttgatgtaaacgaaatcggc

aactacacgcttgtgtatagcgtaacagacagcgcgaaccaaactgtaaccgctgaccgt

aaagtgtctgtgactgatggctctaactgtgccaatgcatgggacagtgaaacagtttat

gtggaaggcgaccaagtctctcacgacggttcaacgtggggagcgggttggtacactcgt

ggcgaagaaccaggaacgacaggtgagtggggcgtttggagaaaagtttcagactcttca

tgtggtggtaacccagatccgggcgatgacttagagcttgcagtttcaggccttcagtca

gaatatgcgccagataacggcaatgttcgtttagacctaacactgacatcaaatgaagcg

ttagatgttaccgctatggtgctcaatagtgcagggactgtggttgagcagactcaggtt

aacttaacggacagccgcgctattaccatggacttgtacgatgtagcggaagggcaatat

gcacttgaagtggtcggtaaagcatctgacggtgaaatggtgatggtaaatgattcgttc

gctgttaaagacggtggcggaacgactccacctccaggtgattacccgccatatgaagca

ggcacaaattacgcagcaggtgacattgtggtcggcagcgataatggtctgtacgaatgt

aaaccttggccgtacacggcatggtgtgcaagcgcgtcttacgcaccagcagaaagccaa

tactggcaagatgcttggacaaagctgtag

>fig|6666666.87819.peg.648 Chitinase (EC 3.2.1.14) [Vibrio sp. Vb258]

atgattcgtataaatacctgtgctgcgagcattgctctagcgctatctggtacagcatta

gctgctccaacagcacctagcatcgacatgtacggttccaacaacctgcaattttccaaa

attgaactcgcgatggaaaccacctctggctacaaccagatggtgaaatatcacgataaa

gcaaaggtcgacgtcaagttcaaccaatggagcggaacgtcaggaaacacgtacaacatc

tactttgatggcgttaaagtagccaccggcccaatcactggcagccaaaccacggcttca

ttcgaatacggccaaggcggcttattcgatatggaaattgaagcgtgtgacgaaactggt

tgtagcaagagcgcacccgctaaaatcaccatcgcagataccgatggctcccacttagcg

ccacttgcgatgaatgtggatccaaacaacaagtcatacaacacagatccaaacacggta

gtaggtacttactttgttgaatggggtatttatggtcgtgactacacggtagataaccta

cccgctgacaacttgacccatatcctttatggcttcatcccaatttgtggtccgaacgaa

tcagttaaatcagtaggtggtaacagctacaacgcactcatgacggcatgtaagggcgtt

aacgattacgaggtggtgatccatgacccttgggcggctttccagaagagcttccctcaa

gcgggtcacgaatacagctcaccgatcaagggtaactacgcaatgatgatggcgctcaaa

cagcgtaatcctgatttgaagatcatcccatcaattggtggctggacattatctgacccg

ttctttgattttacaaccaaagccaaccgtgacaccttcgttgcatctgttaaaaaattc

ctaaacacatggaaattctacgatggtgtagatatcgactgggaataccaaggtggtggc

ggtgctgcgccagagcttggtgacccagaaaatgatggcccagcctacattgcactgatg

gcagaattacgtgcgatgctcgatgagctagaagcagaaaatggccgtacttacgagcta

acatctgcgataggtgttggccacgataaaattgaagacgtgaactacggcgatgctatc

caatacatggattacatctttgcgatgacttacgacttctacggcggttggaacaacgta

ttaggtcaccaagcagcattacactgcggtaacttcatgcgccctggtcagtgtgatggc

tcaggcattgatgaaaatggcaaaccatacacaggcccagcttacacaacggacaacggc

atccaattgctgctcgaacaaggcgttccagctaacaagcttgtcgtaggtgcagcaatg

tacggtcgtggttgggaaggtgtattaccctcatcactttcggatcctagcgaccctatg

acaggtgttggtaacggcaaactgaaaggtagctctgcgcaaggcgtctgggaagatggc

gttatcgactacaaaggcattaaggcgaacatgcttggtgcgaataaccaaggtatcaat

ggcttcgaatacggttacgacgaaatggcagaagcgccttacgtatggaaccgcacttca

ggtcagctgatcacatttgatgatgaccgctcggtaaaagcaaaaggcgcgtacgttcgt

agccttggccttgcaggtctattctcttgggaaattgatgcagataacggcgatatccta

aatgcaatgcatgaaggccttgctggcggtacaacagatcctgtcaaccgcaaaccaacg

gcagctgcaggtgcagaccaatcggttgaaggcccagcttctgtttctctagatggcagc

gcttcaaaagacagcgacggtacgattgcgagctatgcttggtcacaagtaagcggcaca

gcagtaacgctagccaacgctaatgccgctgttgcaagcttcgatgtggttgaagtcgcg

caacaagaagtactcacgttcagcctgacggtaactgacaacgaaggcgctacttctact

gacaccgctgttgtaaccgtaactcctaaggacacaggcccagtcaatacagcaccggtt

gctgtggttacggctccggctgaagttaatgcgggtgatgtagtcgtagttaatgcttcg

gcttcaagcgatgcagaccaagacacattgaccttcacatgggatgtacctgctggtatt

gatgcaacggttcaaggttcttcagtgagctttgtcgcggcggaatacacgcaagataca

acgctgaacttctctgtaacggtcagcgatggcacagagacatcagtagcggctacttca

gtgaaagttattaagaaaaccacaggcggcggtacgtgtactaacgcttgggattcaggc

gcggtttacactggcggcgaccaagtgactcaaggcggtaaggtttgggaagcgaaatgg

tggacaacaggtgaagatccaacaacgacaggccaatggggcgtgtggaaagagatcggt

cctgcaagctgctaa

>fig|6666666.87819.peg.1204 Chitinase (EC 3.2.1.14) [Vibrio sp. Vb258]

atgttaaaaatcaaatatttggcgacagtactgggctgcacattagcagcgcaaagtcat

gcgtctttgaacattcaacctgatccgcaaaatccgaatggttaccttgttgaaaagtcg

gctctacaagctgctgaacaagcgaaaacatctgatcctatgtatgcgatctggtcacag

gcacttcaaactcgcccgaacaccatcgttgaagcgattgaacccggttccccctctaac

cctgaaaacgtaaagcgtgtagagcgcgtgttccctcaatcggaatgggacttcctcact

cagatggcagcgccagaatacacatacactcgcttcttacgtgcgattggtaaattccca

gctttctgtggagagtacacagatggccgtgactccgacgccatctgtaagaaatccatc

attacggcctttgctcacttctctcaagagacgggcggtcacatcgcaatagacaacacc

tctgataatccattagctctagaagagtggcagcaagcgctggtgcatgttcgtgaaatg

ggttggtctgaaggccaagaaggttacaccacaggttgtggtcagaacgattggcagaat

gcccgttggccatgtgctgcgggacagggctacttcggacgtggtgctaaacagctttct

taccattttaactacggcgcgttctctgaagtgatgtttgatggtgatgcgacggtgctt

ctcaagaatccgggcttagtagcggactcttggttgaacttggcttctgctatctggttt

ttccttacccctcaagcccctaaaccagccatgttgcatgttattgaccgtacttggacg

ccatctcaacgcgaattggatgcaggtattggttatggatttggtaccacgatcaacgtg

atcaatggtggtatcgagtgtggcgagcagaacaaagataaaggccaacccgttaaccgt

attcgttactgggaagggctagcggcgcactatgaaattccggtagaagcggatgaagcc

aatacttgttggcagcaaacgccttacggaagcttaaacctcaacggagcaacggatgtg

ttgtacaccaactgggatggcaactggaaatactacgctgaccgcccagaaggttactca

tttgagtgtgagcttgttggtttccaaactgcttattctgctctggtggctggcgattac

gagaagtgtgtgaccaatttttatgggtcacatgcgagttggcctgaagtgaaagtagtc

gataagcttgatccggtagaccctggtactgatccgggtggtaatggttggagtgcgacc

aaggtttacaatgcgggtgaccaagtgactcataatggcgcaacctatgaagcgaaatgg

tggacacaaggggatgaccctgccaatggcggcccttggaaattgattgctggagagcca

acaccgccagttatgacagatccaacgcctgttgatcctgctccggtagacccaacacca

gttgaaccacctgttaccgagccgcctgtcgtcgttgacccatcggtgtttatcacatgg

caagcgggcgttagccaagtgagtaacggtgacaaggtgacacataacggcaagtgtttc

gtggctaaaaacggcccgggcgtatgggaaagccctgttcaatcgaattggttctgggat

gaaattagttgtaattga

>fig|6666666.87819.peg.2697 Chitinase (EC 3.2.1.14) [Vibrio sp. Vb258]

atgaaactgtcatgtatcacacaagctatttctagcaacagtgaccaatcaagttctatc

tttaaatctatttctatctttgggcttagctcaatagccgcggcttgtatgctcacacct

acagcacatgccgatgataaagtcgtcgcagggtattttgcggattggcaatatctgaat

aaagacaactcttataccgttgacgacataccggcagacaagctctcacatattatttac

gccttcttgagtatgtgtggtcctcatagcggtgccggtgagcccgtccaaaaacaaatt

cagcagcaatgtgcagacaaacaaccatataccgcgattattgtggatagagaagctgcg

ttagaagtcgatttcggagacgtggatgttgatgttgcctataaaggtcactttgcccag

ttagctcaattgaaggccgacaatcaaaatatcaaaatattgccatcatttggtggatgg

acaatgtcggaacctttccatgcgatggcgaaagatcctaaagccattgcgcatttttca

aaaactgctgttgagttgattcaaaaatatgacttctttgatggtattgatcttgattgg

gaatacccaggaggcggcggcttaaccacttcgccttggaacccagagactaaactaacg

gatgagcagaaagcgcaagagcgtgaagctttcacacatttagtaaaaacaatcagaagt

gatttggacgctctatcaaagacgactcaaagagattacgagctttcaacggctgtcggt

gttggtgctaaggcagcccaaattgattggaacgcagccagcccatacctgacgaacatg

tttgcgatgacgtatgacttcctcggaggttggggagctcaaacaggtcacacaacaaac

ttacacgccacagagcgcagttggtggggaatgggagctgacgtcttcattaatcagatg

atagagcaaggtattcctagcgagaagctcgtgatcggggcggctttttatggtcgaggc

tggcaagggacaaaagactttgctggagagctacctaaaggtgacctagtttctgagcaa

ggggcgcagtttggtactggtgaaaacggttactttatgttctgggaccttatgaataac

tacagcagcaaacaaggttatgagtataagtacgatgaacaagctcaagcaccttatttg

tggaacccagataaaaaagtattcatatcttttgaagatcagcgttctattaaagcaaag

gctgaatgggcgaagaaatcagacctaggcggcatttttacttgggaattatcaggtgat

ccttccggacagttgatcgatgttatgcatagtgaaatgaataaataa

>fig|6666666.87819.peg.3780 Chitinase (EC 3.2.1.14) [Vibrio sp. Vb258]

atgttgaaacgtaaagctctacaattagcagtatcggtcggtatggcggctatgcctggc

gctgtttatgcgaatggttcagatatgacgaatccagactctggcgtcgtggttggctat

tggcataactggtgtgacggtggcggctaccaaggtggtaatgcgccatgtgtgacgttg

gatgaagtgaacccaatgtacaacatcgtgaatgtgtcattcatgaaggtctacgatgtg

gctgatggtcgaatcccaacttttaaactggacccaaccattgggctttcagaagaacaa

tttattgaccaaatctcggaactcaacaagcaaggtcgctctgtgctcttggctttgggt

ggtgcggatgcacacgtagagctagaaaccggtgatgaaagagcctttgctgatgagatt

atccgtctaactgagcgttacggtttcgatggcctagacatcgaccttgaacaagcagcc

gtaaccgcagcaaacaaccaaaccgtgattccagatgcacttaagctagtgaaagatcac

taccgtgcagaaggtaaaaatttccttattactatggcgcctgagttcccgtatctaacg

acaggtggcaagtacgttccttatatcgataatctagaaggctactacgactggattaac

ccacaattctacaaccaaggtggcgatggtatctgggtggaaggcgtaggttggattgct

caaaacaacgatgcgttaaaagaagagttcatctactacatttctgattctcttatcaac

ggaacgcgtggtttccacaagatcccacatgacaagctcgtgtttggtattccttcaagt

atcgatgccgcagcgacaggttttgtgcaagagccacaagatttatacgacgcatttgat

agcctaacggcgcaaggccaaccgctacgtggtgtgatgacttggtctatcaactgggac

atgggcaccaacaaaaacggccaacaatacaacgagcagttcattaaagactacggcccg

tttgtacacggtcaagtaacgccaccaccagtcgagggtgagccagttcttaaaggtgtc

gaaaatacgcgtgttcttcacggaacaacatttgatcctatggatggcgtgacggcaaca

gacaaagaagacggcgatctaacgtcatcaatcgatgttgaaggttacgttgaaaccagt

gttatcggcacttacgttctaacttaccgagtgaaagacagcgataacaacgaaaccact

aaagcgagaacggtagaggtttacagccagaagccggtatttaatggtgtgtctgacacg

actgttgtgttgggtactgcgtttgatgctatggctggcgtgacggcgaatgatgctgaa

gatggcgacctaacaagcactatcacacatactggcagtgtcgatgtgaacgaaatcggc

aactacacgcttgtgtatagcgtaacagacagcgcgaaccaaactgtaaccgctgaccgt

aaagtgtctgtgactgatggttctaactgtgctgctgcatgggatgcagacaccgtttat

gttgaaggtgatcaagtatctcatgatggttcaacgtggggagctggttggtacactcgt

ggcgaagagccaggaacaacaggtgagtggggcgtttggagaaaagtttcagattcttct

tgtggtggtaacccagatccgggcgatgacttagagcttgctgtttcagggcttcaatca

gaatatgcgccaaataacggcaatgttcgtttagatctaacactgacatcaaatgaagcg

ttagatgttaccgctatggtgatcaacagtgcaggtactgtggttgagcaaactaaggtt

aacgtaacggacagccgcgccattaccatcgacttatacgatgtagcggaagggcaatat

gcacttgaagtggtcggtaaagcatctgacggtgaaatggtgatggtaaacgattcgttc

gctgttaaagacggtggcggtacaacaccacctccaggtgattatcctccatatgaagcg

ggcaccaactacgcagcgggtgacattgtggtcggcagcgataatggtctgtacgaatgt

aaaccttggccatacacggcatggtgtgcaagtgcgtcttacgctccagcagatagccaa

tactggcaagatgcttggactaagttgtag

>fig|6666666.110041.peg.1899 Chitinase (EC 3.2.1.14) [Vibrio Vb278]

atgttaaaaatcaaatatttggcgacagtactgggctgcacattagcagcgcaaagtcat

gcgtctttgaacattcaacctgatccgcaaaatccgaatggttaccttgttgaaaagtcg

gccttacaagctgctgaacaagcgaaaacatccgatcttatgtatgcgatctggtcacag

gcccttcaaactcgcccgaacaccatcgttgaagcgattgaacccggctccccctctaac

cctgaaaacgtaaagcgtgtagagcgcgtgttccctcaatctgaatgggactttctcact

cagatggcagcgccagaatacacctacactcgcttcttacgtgcgatcggtaaattccca

gcgttctgtggagagtacaccgatggccgtgactccgacgccatttgtaaaaaatccatc

attacggcctttgctcacttctcacaagagacgggcggtcacattgcgatagacaacacc

tctgataatccattagctctcgaagaatggcagcaagcgctggtgcatgttcgcgaaatg

ggttggtctgaaggccaagaaggttacaccacaggttgtggtcagaacgattggcagaat

gcccgttggccatgtgctgtgggacagggttacttcggacgtggtgctaaacagctttct

taccattttaattacggcgcgttctctgaagtgatgttcgatggcgatgcgacggtgctt

ctcaacaatccgggcttagtggcggattcttggttgaacttggcttctgctatctggttc

ttccttacccctcaagcccctaaaccagccatgttgcatgttattgaccgcacttggacg

ccctctcaacgcgaattggatgcgggtattggctatggctttggtacaacgatcaacgtg

atcaatggtggtattgaatgtggcgagcagaacaaagacaaaggccaacccgttaaccga

attcgttattgggaagggttagccgcgcactatgaaattccggtagaagcggatgaagcc

aatacttgttggcagcaaacgccttacggaagcttaaacctcaacggagcaacggatgtg

ttgtacaccaattgggatggtaactggaaatactacgcagaccgcccagaagggtattca

tttgagtgtgagcttgttggtttccaaactgcttattctgcgctggtggctggcgattac

gagaagtgtgtgaccaatttttatgggtcacatgcgagttggcctgaagtgaaagtagtc

gataagcttgatccggtagaccctgggacagatcccggtggtaatggctggagtgcgact

aaggtttacaacgcgggtgaccaagtgactcataatggcgcaacctacgaagcgaaatgg

tggacacaaggggatgaccctgctaacggcggcccttggaaattagtggcaggtgagcca

acaccgccggtagtcaccgatcctgcaccagtcgaccctacacctgttgatcctgttccg

gtagacccaacaccagttgaaccacctgttaccgagccgccggtcgtcgttgacccatcg

gtatttatcacatggcaagcgggtgttagccaagtgagtaatggcgacaaggtgacacat

aacggcaagtgcttcgtggctaaaaacggcccgggcgtatgggaaagccctgttcagtcg

aattggttctgggatgaaattagctgcaattga

>fig|6666666.110041.peg.3130 Chitinase (EC 3.2.1.14) [Vibrio Vb278]

atgttgaaacgtaaagctctacaattagcagtatcggtcggcatggcggctatgtctggc

gctgtttatgcgaatggttcagatatgacgaatccagattctggcgtcgtggttggctat

tggcataactggtgtgacggtggcggctaccaaggtggtaatgcgccatgtgtaacgctg

gatgaagtgaacccaatgtacaacatcgtgaatgtgtcattcatgaaggtttacgatgtg

gctgatggtcgaatcccaacttttaaattggacccaaccattgggctttcagaagaacaa

tttattgaccaaatctctgaactcaacaagcaaggtcgttctgtactgttggctttgggt

ggtgctgatgcacacgtagagctagaaaccggtgatgaaagagcctttgctgatgagatt

atccgtcttaccgagcgttacggtttcgacggtctagatatcgaccttgaacaagcagcc

gtaaccgcagcaaacaaccaaaccgtgattcctgatgctcttaagctagtgaaagaccac

taccgcgcagaaggtaaaaacttccttattactatggcgcctgagttcccgtatctaacg

acaggcggtaagtacgtaccttatattgataatctagaaggctactacgactggattaac

ccacagttctacaaccaaggtggcgatggcatctgggttgaaggcgtgggttggattgct

caaaacaacgatgcgttaaaagaagagtttatctactacatctctgattctctgatcaac

ggaacgcgtggcttccacaaaatcccacatgacaagctcgtgtttggtattccttcaagt

atcgatgctgcagcgacaggttttgtgcaagagccacaagatttatacgacgcgtttgat

agcctaacggcgcaaggccaaccgctacgtggtgtgatgacttggtctatcaactgggac

atgggcaccaacaaaaacggtcaacaatacaacgagcagtttattaaagactacggcccg

tttgtccacggtcaagtgacgccaccaccagtagagggtgagcccgttcttaaaggtgtt

gaaaatacgcgtgttcttcacggcacaacgtttgacccaatggaaggcgtaacggcaaca

gacaaagaagacggcgatctaacgtcatcaatcgatgttgaaggttacgttgaaaccagt

gttattggcacttacgttttgacttaccgagtgaaagacagcgataacaacgaaaccaca

aaagcgcgaacggtagaggtttacagccagaagccagtatttactggtgtgtctgacaca

accgttgtgttgggtactgcgtttgatgctatggcaggcgtgacagcgaatgatgctgaa

gatggcgacctaacaagcactatcacacacactggcagtgtcgatgtaaatgaaatcggc

aactacacacttgtgtatagcgtaacagacagcgcaaaccaaactgtaaccgctgaccgc

aaagtgtctgtgactgatggttctaactgtgctgcggcatgggatgccgacaccgtttat

gttgaaggtgatcaagtatctcatgacggttcaacgtggggagcgggttggtacactcgc

ggcgaagagccgggaacaacaggtgagtggggcgtttggagaaaagtttcagactcttca

tgtggtggtaacccagatccgggcgatgacttagagcttgcagtttcaggccttcaatca

gaatatgcgccggataacggcaatgttcgtttagatctgacactgacatcaaatgaagcg

ttagatgttacggctatggtgatcaacagtgcaggtactgtggttgagcaaactaaggtt

aacgtaacggacagccgcgctattaccatcgacttgtacgatgtagcggaagggcaatat

gcacttgaattggtcggtaaagcatctgacggtgaaatggtgatggtaaacgattcgttc

gctgttaaagacggtggcggtacaacaccacctccaggtgattaccctccatatgaagcg

ggcaccaactacgtagcgggtgacattgtggtcggcagcgataatggcttgtacgaatgt

aagccttggccatacacggcatggtgtgcaagcgcgtcttacgctccagcggatagccaa

tactggcaagatgcttggactaagttgtag

>fig|6666666.110041.peg.3649 Chitinase (EC 3.2.1.14) [Vibrio Vb278]

atgaaactgtcatttatcacacacgctatttctagcaacagtggccaatcaagttctatc

tttaaatctaattctgtcattgcgcttagcttaatcaccacggcttgtatgctcacacct

acagcacatgccgatgataaagtcatcgcagggtattttgcggattggcaatatctgaat

aaagataacccttataccgttgacgacatacctgcagacaagctctcacatattatttac

gccttcttgagcatgtgtggtcctcatagcggtgctggtgagcccgtccaaaaacaaatt

cagcagcaatgtgcagacaaagaaccatataccgcgattattgtggataaagaagctgcg

ttagaagtcgattttggagacgtggatgttgatgtcgcctataaaggtcactttgcccag

ttagctcaattgaaggccgacaatcaaaatatcaaaatcttgccatcatttggtgggtgg

acaatgtctgaacctttccatgcgatggcgaaagatcctaaagccattgcgcatttttca

aaaactgctgttgagttgatccaaaaatatgacttctttgatggtattgatcttgattgg

gaatacccaggaggcggcggattaaccacttcgccttggaactcagagactaaactaacc

gatgagcagaaagcgcaagagcgtgaagcttttacacttttagtaaaaacaatcagaagt

gatttggacgctctatcaaagacgactcaaagagattacgagctttcaacggctgtcggt

gttggcgctaaggcagcccaaattgattggaacgcagctagcccatatctgacgaacatg

tttgcgatgacgtatgacttcctcggaggttggggagctcaaacaggtcacacaacaaac

ttacacgccacagagcgcagttggtggggaatgggagctgacgtcttcattaatcagatg

atagagcaaggtattcctagcgagaagctcgtgatcggggcggctttttatggtcgaggc

tggcaagggacaaaagactttgctggagagctgcctaaaggtgacctaatttctgaacaa

ggggcgcagtttggtaccggtgaaaacggttactttatgttctgggaccttatgaataac

tacagcagcaaacaaggttatgagtacaagtatgacgagcaagctcaagcaccttatttg

tggaacccagataaaaaagtattcatatcttttgaagatcagcgctctattaaagcaaag

gcagaatgggcgaagaaatcagacctaggcggcatttttacttgggaattatcaggagat

ccttccggacagttgatcgatgttatgcatagtgaaatgaataaataa

>fig|6666666.110041.peg.4657 Chitinase (EC 3.2.1.14) [Vibrio Vb278]

atgattcgtataaatacctgtgctgcaagcattgctctagcgctatctggtacagcatta

gctgctccaacagcacctagcatcgacatgtacggttccaataacctgcaattttctaaa

attgaactcgcgatggaaaccacctctggctacaaccagatggtgaaatatcacgaccaa

gcgaaggtcgacgtcaagttcaaccaatggagcggaacgtcaggaaacacgtacaacatc

tactttgatggcgttaaagtagccaccggcccaatcactggcagccaaaccacggcctca

ttcgaatacggccaaggcggcttattcgatatggaaattgaagcgtgtgacgaaactggt

tgtagcaagagcgcacccgctaaaatcaccatcgcagataccgatggctcccacttagcg

ccacttgcgatgaatgtggatccaaacaacaagtcatacaacacagatccaaacacggta

gtaggtacttactttgttgaatggggtatttatggtcgtgactacacggtagataaccta

cccgctgacaacttgacccatatcctttatggcttcatcccaatttgtggtccgaacgaa

tcagttaaatcagtaggtggtaacagctacaacgcactcatgacggcatgtaagggcgtt

aacgattacgaggtggtgatccatgacccttgggcggctttccagaagagcttccctcaa

gcgggtcacgaatacagctcaccgatcaagggtaactacgcaatgatgatggcgctcaaa

cagcgtaatcctgatttgaagatcatcccatcaattggtggctggacattatctgacccg

ttctttgattttacaaccaaagccaaccgtgacaccttcgttgcatctgttaaaaaattc

ctaaacacatggaaattctacgatggtgtagatatcgactgggaataccaaggtggtggc

ggtgctgcgccagagcttggtgacccagaaaatgatggcccagcctacattgcactgatg

gcagaattacgtgcgatgctcgatgagctagaagcagaaaatggccgtacttacgagcta

acatctgcgataggtgttggccacgataaaattgaagacgtgaactacggcgatgctatc

caatacatggattacatctttgcgatgacttacgacttctacggcggttggaacaacgta

ttaggtcaccaagcagcattacactgcggtaacttcatgcgccctggtcagtgtgatggc

tcaggcattgatgaaaatggcaaaccatacacaggcccagcttacacaacggacaacggc

atccaattgctgctcgaacaaggcgttccagctaacaagcttgtcgtaggtgcagcaatg

tacggtcgtggttgggaaggtgtattaccctcatcactttcggatcctagcgaccctatg

acaggtgttggtaacggcaaactgaaaggtagctctgcgcaaggcgtctgggaagatggc

gttatcgactacaaaggcattaaggcgaacatgcttggtgcgaataaccaaggtatcaat

ggcttcgaatacggttacgacgaaatggcagaagcgccttacgtatggaaccgcacttca

ggtcagctgatcacatttgatgatgaccgctcggtaaaagcaaaaggcgcgtacgttcgt

agccttggccttgcaggtctattctcttgggaaattgatgcagataacggcgatatccta

aatgcaatgcatgaaggccttgctggcggtacaacagatcctgtcaaccgcaaaccaacg

gcagctgcaggtgcagaccaatcggttgaaggcccagcttctgtttctctagatggcagc

gcttcaaaagacagcgacggtacgattgcgagctatgcgtggtcacaagtaagtggttcg

gcagtaacgttggcgaacgcgaatgccgctgttgcaagcttcgatgtggttgaagtcgct

cagcaagaagtactcacgttcagcctaacggtaactgataacgaaggtgctactgcaact

gacaccgttgttgtaaccgtaactcctaaggacacgggcccagtcaatacagcaccggtt

gctgtggttacggctccggctgaagttaatgcgggtgatgtagtcgtggttgatgcttcg

gcttcgagcgatgctgaccaagacacattgaccttcacatgggatgtacctgctggtatc

gatgcaacggttcaaggttcttcagtgagctttgtcgcggcggaatacacgcaagataca

acgctgaacttctctgtaacggtcagcgatggcacagagacatcggtagcagctacttca

gtgacagttcttaagaaaaccacaggcggcggtacgtgtactaacgcttgggattcaggc

gcggtttacacgggtggcgaccaagtgactcaaggcggtaaagtttgggaagcgaaatgg

tggacaaccggtgaagatccaacaacgacaggccaatggggcgtgtggaaagagatcggt

cctgcaagctgctaa

>fig|6666666.87821.peg.54 Chitinase (EC 3.2.1.14) [Vibrio sp. Vb255]

atgattcgtataaatacctgtgctgcgagcattgctctagcgctatctggtacagcatta

gctgctccaacagcacctagcatcgacatgtacggttccaacaacctgcaattttccaaa

attgaactcgcgatggaaaccacctctggctacaaccagatggtgaaatatcacgataaa

gcaaaggtcgacgtcaagttcaaccaatggagcggaacgtcaggaaacacgtacaacatc

tactttgatggcgttaaagtagccaccggcccaatcactggcagccaaaccacggcttca

ttcgaatacggccaaggcggcttattcgatatggaaattgaagcgtgtgacgaaactggt

tgtagcaagagcgcacccgctaaaatcaccatcgcagataccgatggctcccacttagcg

ccacttgcgatgaatgtggatccaaacaacaagtcatacaacacagatccaaacacggta

gtaggtacttactttgttgaatggggtatttatggtcgtgactacacggtagataaccta

cccgctgacaacttgacccatatcctttatggcttcatcccaatttgtggtccgaacgaa

tcagttaaatcagtaggtggtaacagctacaacgcactcatgacggcatgtcagggcgtt

aacgattacgaggtggtgatccatgacccttgggcggctttccagaagagcttccctcaa

gcgggtcacgaatacagctcaccgatcaagggtaactacgcaatgatgatggcgctcaaa

cagcgtaatcctgatttgaagatcatcccatcaattggtggctggacattatctgacccg

ttctttgattttacaaccaaagccaaccgtgacaccttcgttgcatctgttaaaaaattc

ctaaacacatggaaattctacgatggtgtagatatcgactgggaataccaaggtggtggc

ggtgctgcgccagagcttggtgacccagaaaatgatggcccagcctacattgcactgatg

gcagaattacgtgcgatgctcgatgagctagaagcagaaaatggccgtacttacgagcta

acatctgcgataggtgttggccacgataaaattgaagacgtgaactacggcgatgctatc

caatacatggattacatctttgcgatgacttacgacttctacggcggttggaacaacgta

ttaggtcaccaagcagcattacactgcggtaacttcatgcgccctggtcagtgtgatggc

tcaggcattgatgaaaatggcaaaccatacacaggcccagcttacacaacggacaacggc

atccaattgctgctcgaacaaggcgttccagctaacaagcttgtcgtaggtgcagcaatg

tacggtcgtggttgggaaggtgtattaccctcatcactttcggatcctagcgaccctatg

acaggtgttggtaacggcaaactgaaaggtagctctgcgcaaggcgtctgggaagatggc

gttatcgactacaaaggcattaaggcgaacatgcttggtgcgaataaccaaggtatcaat

ggcttcgaatacggttacgacgaaatggcagaagcgccttacgtatggaaccgcacttca

ggtcagctgatcacatttgatgatgaccgctcggtaaaagcaaaaggcgcgtacgttcgt

agccttggccttgcaggtctattctcttgggaaattgatgcagataacggcgatatccta

aatgcaatgcatgaaggtctagcaggtggcaccaccgatcctgtaaaccgtaaaccaact

gcagcagcaggtgccgaccaatccgttgaaggcccagcttttgtttctctagatggcagc

gcttcaaaagacagcgacggcacaatcgcgagctacgcttggtcacaagtaagcggaact

gccgtaacactggctaatgccaatgcagctgttgcaagcttcgatgttgtggaagttgct

cagcaagagacattaacctttagtctaaccgtaactgataacgaaggtgctactgcaact

gacacggttgttgtaacggtaacccctaaagacacaggcccagtcaacaccgctccggta

gcagtggttacggctccggctgaagtcaatgcgggtgacgttgttgtggttgatgcgtca

gcgtctagcgatgcggatcaagacacattaaccttcacgtgggatgtaccagcaggtatc

aacgcaacagtacaaggcgcttcagtaagctttgtcgcggctgaatacgcgcaagacaca

gtactgaacttctctgtgacagtgagtgatggtaccgatacatcagtcgcggctgcatca

gtgaaagttcttaagaaaaccacaggcggcggtacgtgtactaacgcttgggattcaggc

gcggtttacactggcggcgaccaagtgactcaaggcggtaaggtttgggaagcgaaatgg

tggacaacaggtgaagatccaacaacgacaggccaatggggcgtgtggaaagagatcggc

ccagcaagctgtgctaactaa

>fig|6666666.87821.peg.3380 Chitinase (EC 3.2.1.14) [Vibrio sp. Vb255]

atgaaactgtcatttatcacacacgctatttctagcaacagtgaccaatcaagttctatc

tttaaatctaattctatcattgcgcttagcttaatagccacggcttgtatgctcacacct

acagcacatgccgatgataaagtcgtcgcagggtattttgcggattggcaatatctaaat

aaagataacccttataccgttgacgacataccgacagacaagctctcacatattatttac

gccttcttgagcatgtgtggttctcatagtggtgccggtgagcccgtccaaaaacaaatt

cagcagcaatgtgcagacaaacaaccatataccgcgattattgtggataaagaagctgcg

ttagaagtcgatttcggagacgtggatgttgatgttgtctataaaggtcactttgcccag

ttagctcaattgaaggccgacaatcaagatatcaaaatcttgccatcatttggtgggtgg

acaatgtctgaacctttccatgcgatggcgaaagatcctaaagccattgcgcatttttca

aaaactgctgttgagttgatccaaaaatacgacttttttgatggtattgatcttgattgg

gaatacccaggaggcggcggcttaaccacttccccttggaacccagagacgaaactaacg

gatgagcagaaagcgcaagagcgtgaagctttcacacttttagtaaaaacaatcagaagt

gatttggaagctctatcaaagacgactcaaagagattacgagctttcaacggctgtcggt

gttggtgctaaggccgctcaaattgactggaacgcagctagcccatatttgacgaacatg

tttgcgatgacgtatgacttcctcggaggctggggagctcagactggtcacacaacaaac

ttacatgccacagagcgcagttggtggggaatgggagctgatgtcttcattaatcagatg

atagagcaaggtattcctagcgagaagctcgtgatcggggcggctttttatggtcgaggc

tggcaagggacaaaagactttgctggagagctacctaaaggtgacctagtttctgaacaa

ggggcgcagtttggtaccggtgaaaacggttactttatgttctgggatcttatgaataac

tacagcagcaaacaaggttatgagtataagtacgatgagcaagctcaggcaccttatttg

tggaacccagataaaaaagtattcatatcttttgaagatcagcgttctattaaagcaaag

gcagaatgggctaagaaatcagacctaggcggcatttttacttgggaattatcaggagat

ccttccggacagttgatcgatgttatgcatagtgaaatgaataaataa

>fig|6666666.87821.peg.6562 Chitinase (EC 3.2.1.14) [Vibrio sp. Vb255]

atgttgaaacgtaaagctctacaattagcagtatcggtcggcatggcggctatgtctggc

gctgtttatgcgaatggttcagatatgacgaatccagactctggcgtcgtggttggctat

tggcataactggtgtgacggtggcggctaccaaggtggtaatgcgccttgtgtgacgctg

gatgaagtcaacccaatgtataacatcgtgaatgtgtcatttatgaaggtttacgatgtg

gctgatggtcgaatcccaacttttaaactggacccaaccgttgggttatcagaagaacaa

tttattgaccaaattgccgaactcaacaagcaaggccgttctgtactgatagcgctgggc

ggtgctgatgcacacgtagaacttgaaacgggtgatgaaagagcctttgctgatgagata

atccgccttactgagcgttacggtttcgatggcctagacatcgaccttgaacaagcagcc

gtaaccgcagcaaacaaccaaaccgtgattccagatgcacttaagctagtgaaagatcac

taccgtgcagaaggtaaaaatttccttattactatggcgcctgagttcccgtatctaacg

acaggtggcaagtacgttccttatatcgataatctagaaggctactacgactggattaac

ccacaattctacaaccaaggtggcgatggtatctgggtggaaggcgtaggttggattgct

caaaacaacgatgcgttaaaagaagagttcatctactacatttctgattctcttatcaac

ggaacgcgtggtttccacaagatcccacatgacaagctcgtgtttggtattccttcaagt

atcgatgccgcagcgacaggttttgtgcaagagccacaagatttatacgacgcatttgat

agcctaacggcgcaaggccaaccgctacgtggtgtgatgacttggtctatcaactgggac

atgggcaccaacaaaaacggccaacaatacaacgagcagttcattaaagactacggcccg

tttgtacacggtcaagtaacgccaccactagtcgagggtgagccagttcttaaaggtgtc

gaaaatacgcgtgttcttcacggaacaacatttgatcctatggatggcgtgacggcaaca

gacaaagaagacggcgatctaacgtcatcaatcgatgttgaaggttacgttgaaaccagt

gttatcggcacttacgttctaacttaccgagtgaaagacagcgataacaacgaaaccact

aaagcgagaacggtagaggtttacagccagaagccggtatttaatggtgtgtctgacacg

actgttgtgttgggtactgcgtttgatgctatggctggcgtgacggcgaatgatgctgaa

gatggcgacctaacaagcactatcacacatactggcagtgtcgatgtgaacgaaatcggc

aactacacgcttgtgtatagcgtaacagacagcgcgaaccaaactgtaaccgctgaccgt

aaagtgtctgtgactgatggttctaactgtgctgctgcatgggatgcagacaccgtttat

gttgaaggtgatcaagtatctcatgatggttcaacgtggggagctggttggtacactcgt

ggcgaagaaccaggaacaacaggtgagtggggcgtttggagaaaagtttcagattcttct

tgtggtggtaacccagatccgggcgatgacttagagcttgctgtttcagggcttcaatca

gaatatgcgccaaataacggcaatgttcgtttagatctaacactgacatcaaatgaagcg

ttagatgttaccgctatggtgatcaacagtgcaggtactgtggttgagcaaactaaggtt

aacgtaacggagagccgcgccattaccatcgacttatacgatgtagcggaagggcaatat

gcacttgaagtggtcggtaaagcatctgacggtgaaatggtgatggtaaacgattcgttc

gctgttaaagacggtggcggtacaacaccacctccaggtgattatcctccatatgaagcg

ggcaccaactacgcagcgggtgacattgtggtcggcagcgataatggtctgtacgaatgt

aaaccttggccatacacggcatggtgtgcaagtgcgtcttacgctccagcagatagccaa

tactggcaagatgcttggactaagttgtag

>fig|6666666.87821.peg.10578 Chitinase (EC 3.2.1.14) [Vibrio sp. Vb255]

atgttaaaaatcaaatatttggcgacaatactgggctgcacattagcagcgcaaagtcat

gcgtctttgaacattcaacctgatccgcaaaacccgaatggttaccttgttgaaaagtcg

gccctacaagctgctgaacaagcgaaaacatccgatcctatgtatgcgatctggtcacag

gcacttcaaactcgcccgaacacaatcgttgaagcgattgaacccggttccccctctaat

cctgaaaacgtaaagcgtgtagagcgcgtgttccctcaatcggaatgggacttcctcact

cagatggcagcgccagaatacacatacactcgcttcttacgtgcgattggtaaattccca

gctttctgtggagagtacacagatggccgtgactccgacgccatctgtaagaaatccatc

atcacggcctttgctcatttctcacaagagacgggtgggcacatcgctatagacaacacc

tctgataatccattagctctcgaagagtggcagcaagcgctggtgcatgttcgtgaaatg

ggttggtctgaaggccaagaaggttacaccacaggttgtggtcagaacgattggcagaat

gcccgttggccatgtgctgcgggacagggctacttcggacgtggtgctaaacagctttct

taccattttaactacggcgcgttttctgaagtgatgtttgacggtgatgcgactgtgctt

ctcaacaatccgggcttagtggcggattcttggttgaacttggcttctgctatctggttc

ttcctgacccctcaagcccctaaaccagccatgttgcatgttattgaccgtacttggacg

ccctctcaacgcgaattggatgcgggtattggttatggctttggtaccacgatcaacgtg

atcaatggtggtattgaatgtggcgagcagaacaaagacaaaggccaaccagttaaccgt

attcgttattgggaagggttagcagcgcactatgaaattcctgtagaagcggatgaagct

aatacttgttggcagcaaacgccttacggaagcttgaatctcaacggcgcgaccgatgtg

ttgtacaccaactgggatggaaactggaaatactacgctgaccgcccagaaggctactca

tttgaatgtgaacttgtgggtttccaaactgcttattctgctctggtggctggcgattac

gagaagtgtgtgaccaatttttatgggtcacatgcgagttggcctgaagtgaaagtggtc

gataagcttgatccggtagaccctggtactgatccgggtggtaatggttggagtgcgacc

aaggtttacaatgcgggtgaccaagtgactcataatggcgcaacctatgaagcgaaatgg

tggacacaaggggatgaccctgccaatggcggcccttggaaattgattgctggagagcca

acaccgccagttatgacagatccaacgcctgttgatcctgctccggtagacccaacacca

gttgaaccacctgttaccgagccgcctgtcgtcgttgacccatcggtgtttatcacatgg

caagcgggcgttagccaagtgagtaacggtgacaaggtgacacataacggcaagtgtttc

gtggctaaaaacggcccgggcgtatgggaaagccctgttcaatcgaattggttctgggat

gaaattagttgtaattga

>fig|511678.20.peg.280 Chitinase (EC 3.2.1.14) [Aliivibrio sp. EL58]

atgtttaaaaataaactggggctgtgctccgcggcaattactcttgccctctcagcccca

acttatgcagcaacgccaggacaagccattatttcttggatggaaacagattttgctatt

attgaagtagatcaagctgctacatcatataaagatcttgtcacgctaaaagactttgca

gaagttcctgtaacatgggaccgatggtctggagaatcagcggaagtatggcgcgtgcta

ttaaatggggttgttgttcatgaagaagccgtttctcctgctgcttcacaaaaagcctct

accgttcttcaagttcgtcaaggtggccaatattcgatgacagtacaactttgtaatggc

gctggagcaacagaagaatgtagcaccagtgatgcaaagtcgattgtcgttgctgatact

gatggtagtcatttagatcccctaccaatgaatattgacccaacaaacggaaattacaca

acaccagaaggaatggtatcaggtgcttattttgttgaatggggcgtctatggacgtaaa

tttgcagttgatcagatccctgctcaaaacctcactcatatcctctatggctttgttcca

atttgtggccctaacccctctttaggtgagattgaaaatggcaatagtttagctgctctt

aatcgtgcttgtgctggaactcctgattatgaagtggttatccatgacccatgggcagca

gtacaaatgccacaacctcaatcaggtcatgtccacagcacaccttataaagggacttat

ggtcaaatgatggctcttaaacaacgctatccagacctaaaaattgttccttctattgga

ggttggacattatcagatcctttctatgactttgttgataaatctaaacgtgacatcttt

gtttcttctgttaagcaatttcttaaaacatggaagttttatgatggtgtagatattgat

tgggaattccctggcggcgatggtgcaagtgcaactggtggtgatcctgtaaatgacggc

ccagcttatgtagccctaatgcaagaactaagagcaatgctcgatgagctaagtgcagaa

acaggaaaaacctatgagttaacctctgcaatcggtgctggctatgacaagattgaagat

gttgactatgctgctgcatcgcagtacatggattatatttttgctatgacatacgatttc

ttcggcggttggaacaacgtagttggccatcaaactgcattaaattgtggctcacatatg

tcacaaggtgaatgcgatggtactggtcttgatgataaaggtgaaccgcgtaaaggccca

gcatacaccattacaaatgcgattgacttactacttgcccaaggtgttgacgctaaaaaa

ttagtcgtaggtactgcaatgtatgctcgtggttggacaggcgtcacgcgtgaaagcatg

actgatccaacaaacccaatgactggtgttggtaatggtaaagttgctggttcttgggaa

gcaggtgtaattgactataaagatgtcgtgactcgctacgtagataaagtcggtgttgaa

gttggttatgatgaagtcgctcaagccgcttatgcttatgatcctagtaatggtgatctt

gttacttacgataataagaagtcggtattagctaaaggtgagtatgttcgttcactcggt

ttagctggtttatttgcatgggaaatcgatgcagataatggtgacattctaaatgcaatg

caagaagggcttgctggtgacggtacagttactcctcctgcaaacaaaaaaccagtggct

aatgctggcgtagatgtttcagttatcgcccctgcatcggtacagcttgatgcttcgtca

tcatctgatagtgatggttctattgtttcttatgcatggacgcaagtctctggatctagc

gttacattaaatggtgcagatgcagttaatccaagctttacaacggatacgttaactcag

tctgaaatacttcaattcacgctaaccgttactgatgataaaggtgctactgcatctgac

tcagtagcagtgtccgtaagtgttgaaggtacagggccggtaaataccccacctgtagct

gttatcgttgctccaatatcagtaaacaaaggcgatgttgttacgcttgatgcatcaaca

tcaactgatgcagacagtgatccattaacctattcatgggtaatccctattggtattgat

gctactgttgttggctctcaagcaacctttacagcaggctcttatactgtagatactcct

tttactttctctgtcacagtgaatgatggtcaggcatcagatacttcaaacgttacccta

actgttctgaaagatgcgggagaacctcctatcacttgtgataatgcatgggactcatct

gcaatctataacggcggagaccaagtttcacattcaggcagtgtatgggaagctaaatgg

tggactacaggcgataacccaagccaatctggtgattggggagtatggaaaagcgtcggt

atttctacttgtaactaa

>fig|511678.20.peg.1531 Chitinase (EC 3.2.1.14) [Aliivibrio sp. EL58]

atgttcaaactcgctatactcccatcattacttgcctgcagttttgcagccaatagtttc

actatgaaccctcagcctgatcctgaaaacccaacaggttatattgtttctaaagcagaa

attaaagctgccgaagatgcgaaaacattagaccctatgtatgacgtctgggctaaagca

ttagaaacattaccaaataccgcagtagaagcgattgatgctggtgttgcaacaaatcct

gcgaacgtgaaacgtgttgagcgtgtttttcctcaatctgaatggtttttcttaactcaa

atggcagcgcctgaatacacttatactcgtttcttacgtgcaattggcaagttccctgct

ttttgtggtgactatacggatgggcgtgatgctgatgccatttgtaaaaaatccatcgta

acggcatttgcgcatttctctcaagagactggcggtcacattgccgttgataacgtatct

gacaacccccttgcgcttgaagagtggcaacaagccttagtgcacgttcgtgaaatggga

tggtctgaaggtcaagaaggatacactactggttgtggccaaaatgattggcaaaataaa

cgctggccctgtgccgcaggtgaaggctatttcggccgtggtgctaaacaattatcttac

cactttaactacggcgctttctctgaagttatgtacgatggtgatgcgacagtattgcta

aataatccagggctagtcgctgattcttggttaaatcttgcctctgctatttggttcttt

ttaacaccccaagcaccaaaacctgccatgctacatgttatcgaccgtacttggacccct

tctcaacgagaaactgatgcaggtatcggctacggatttggtacaacgattaacgtgatt

aatggcggtattgaatgtggagagcaaaacaaagataaaggccaacccgttaaccgcatt

cgctattgggagggtttagcaaaacattaccaaattccagtagaagcggatgaagcaaat

acctgttggcaacaaaccccttatggcagtttgaatttaaatggtgcaaccgatgtgctt

tatactaactgggatggtaattggaaatactattctgatcgtccagaaggtgcttctttt

gaatgtgaattagtgggcttccagactgcatattctgcgcttgtccctggtgattatgag

aaatgtgttactaacttctatgagtctcacgctaattggcctgtgactcgtgtggttgat

tctttaccaacagacccgactgatcccaccgatccggtagatccaacaaatacttgggat

gcaaataaggtttataacactggcgatcaagtagttgtaagtggtgtaacttaccaagct

aaatggtggaaccaaggtgataacccagcaacaagtacaactggtgtttgggaagtgatc

agcggaactacaactgaaaattcagtagaaccggctcctattgaaccaactccgcctact

ccagtagagccaacacctccaacagtacccgtagatcctgattcaacatgggatgtaact

gcaagttataatagcggtgaccaagtgaccgttaacggcgtaacttatcaagctcagtgg

tggactcaaggtaataaccctgaagcttctggtgaatggggagtttggaaaagagtgtaa

>fig|511678.20.peg.1798 Chitinase (EC 3.2.1.14) [Aliivibrio sp. EL58]

atggaagaaataaaaattatgaaaaaaataaataaaataactctagcaatattgagttct

atgcttagtggctatgtatatgcatcggatgaaatccaaacaactaatgttgttcctttt

atttctggtgggctatgtgaaagtttcaatgtgtatccagattggacacgtggcgatcat

gcaacaaatggtgacattatggtgcataaaaatatcgcttattcggcagtttactggaca

caaagtattcctggaagtgatagttcatgggctctccacttaaattgtgatggttcagaa

cctggtactgcgccagtcctttctttaccaaatcctctggatcctattcgtttagaagtt

gctggttggccaaatacatttgtggtagctagcccatctacattagcacctgcgacacta

attattcagactaataatagcgatgtgttagctgacgtggaacagcttacgcgtgcgttt

gtgtctattatcgagcaagctgagactgctggcagtacatcggtcattattaattcagat

gtgttggatcttgcaacccgagataaaggcgaatctttcggtgctatagcagtaaaacaa

gcactgattaacgcaattgacatcacgaacagtagcattgatattacagctatcaatgaa

ctatctgatgatctaaaaggttgggctcaagcgcataacctaattcttagcactttggct

cctacaataagttttggttggtcactgagtattggtgattttgtatacgatacacattct

ggtcgtcaatcagtgtgggataaagcgtcagtattcagtgctgatctactcgctacgcta

gatttatacgaactaggatctgtaaacaaagctgattttattgcgtttactaaatcaagt

acaacagcagcactgacaagtgagcaatggcataacgcgttagagtacgtgaagcaagta

tctgattacgttaagagtcctgtgatgttggctaatatgccgacaaaccaagcagtagac

tactttatggggaattctgataataaacctcaactacgtaaagccgcatttagtaatgta

tttgctctaacgtttgatcaagatagccaagatttggcaactaagattgagcattatcaa

agtgcgaaagttccattgtactacgtaggcgaagagctagagaaaggatcattaactcgt

atcgaagcactaaaccaacagttagctgctgctgaaaatgcgatggacaatgaagcgttt

ttatatgaaacaccacaatcacagtgggttccatcaaccgtgtataagtggaatgatttt

ctagatggtttgaatgcaatgcacaacattggtgttgcaggtaacaagttctggttaatg

aatgatgaagttgatgacgaaacaaacatcaaatatgccaaaattgcgattgcagctttc

ttagcacaaagtatgcaagaaaccattcgctacaatgcgtgtgatgaaaataactggtct

gaagtgaagtatggtgcaccaaccgattacccgatgacagctagctgcggtcaacttggt

cagaagtatgcagattacggtgtgaacccagtgtcaggtttagattatgcctactcttgt

cctcgtgacaataagatggaagtcactgccttgacgcatgctaaatggtatggcgctcct

gctccagtctttgctgctccagatgctgtacttgaagagcgtggtttattggtaaatgga

catgctggtcgttggacaaataacggccactgtaatgtagtgccagaaaaagtagatact

tctaaacaagtatgggagcgtgatgaatgtaaaatttacgttggtcaaaaagctggtacg

ttcatttgggatggtagtagccaagagagtgtggaaggttgcggttggtggggacgtggt

gttatccaaactacgggtcgtcaaaacttcggtacacttaaccactacttgggacgctca

catgttgatcccgaaacaattggtacaacgattgatggtgtaatggttgaagcgccacca

gcaaatccactttatgcagagcttgatttttgttcaaatccaggtttgatctgtagctct

gaagagaacaaagaaattaagtggatcgcaggcttgttctactgggtaacatcagtacag

gcatacaatgatgaaggtggtcaatatggtgactggaactattataacgagcttaaaaag

tacgtagatagtggcttgaaaggaactcaatttattgatgatgtttctggtattgtaaac

cgtggttgtcctgatacaacgtgttcaacaggcgatgttcataacattaaagagcgtcaa

gataacttcaaattagtattacaaaaattaggtcttaacccacaataa

>fig|511678.20.peg.2306 Chitinase (EC 3.2.1.14) [Aliivibrio sp. EL58]

atggcattactaacaaaaaaaatgaagctaaacactattgcattagcattattgggagca

ggtttctctgttcaatcacacgcaaatgacatgataaacccagatggcggtattgttgtt

ggctattggcataactggtgcgacggtcgtggttatcaaggtggtaacgctccttgtatg

acattaaaagaaacaaatcctatgtataatgtcgttgatatttcatttatgaaagtctat

gacacttccgaaggtcgtattccgacctttcgccttgacccaacagttggtttgagtgaa

gctgagtttattgaacaaattcaagagttgaatactcaaggtcgctctgtattaatcgca

cttggcggtgcagacgctcatatcgaattaaaacgtggggatgaaataccatttgcagac

gaagttatccgcttagttgaactatatggttttgatggcttagatatcgatttagagcaa

gccgctgtaactgctgccgataaccaatgggtaattccagaagcactaaaaatagtaaaa

gatcattatagatcgcaagggaagaatttccttattacgatggctccagaattcccttac

ctaaaagctggtgacaaatacgttccttatcttgagcgtttagaagggtattatgattgg

attaacccacaattctataaccaaggtggcgatggcatttgggttgatgaagagaacgca

tggattactcaaaacagtgatgcaatgaaagagaagtttatctactacatctctgactca

ttgatcaacggtactcgtggttttcacaaaatccctcacgacaaacttgtttttggtatc

ccaacaaataatgatgctgcagcaacaggatttgttaaagaaccccaagatctttacaac

gcatttgatgctctaaagcagcagggacaacctttacgtggtgtaatgacttggtctatt

aactgggatattggtactgattctgctggtactccatataacagcagctttattaatgct

tacggcccatatattcacagtcaaacgccaccaccaccagtggttggtaaacctgtgttt

acagggttaactgatacacgagttccacacggtagtgtatttgattcattagcgggagtt

accgcaatggataaagaagacggtaacctaacatcatcaattacggttgagggctcagtg

aacacacaggttcttggtgacaatgtacttgcttatagcgtaactgactctgatggtaac

gaaacgaaacaagctcgtaatattgaagtttatagtgcgcttcctgaattaacaggcgtt

gcaaacacaaccattaaaataggctcagatttcaaccctcgaactaacgtaaaagcaact

gatgctgaagatggagacttaacgtctcagatctctattgaaggaacagtaaatacttct

cttgcaggtaagtacacattaacttattcagtaacagacagtgcggaccaaactacaaca

gcacagcgtacaattacggtaaatgatggttcacaagtttgtgttaacccatggaaagcc

gatcaagtttatttaactgacgaaacagtaagccataacggaaaaacatggcaagctggc

tggtggacacaaggtgatgaacccggcacaaccggagaatggggagtttggaaacaaatt

ggtgataatgattgtggtggtgtaactccagaacccggtattgagttaacggcaaaaatc

acagatctcagcaatgaatattcaattgtaaactcaactgctaacgtaacgttttctatt

acaacaaatgataccgcttctgtaacggttgatatcattgaccgtatgggcaacagcatt

gcatcggaatcaacctcaataactggtattcaagctgtttctttaacgttaaacaacgtt

gaatccggttattactcaatgcgtttattagcatcgaacgctgatgactcggtagaagaa

cgttacagctttaatctagtttctgaagatacgccaacaccaccaccaaccgatattcct

gcatatgaagtaggtaaaacatacgcagatggcgaacaagttctaggttctgatggaaac

atttatcagtgtaagccttggccatacacaccatggtgttcaagtgcatcttatgctcca

gcagaaagtcaatactgggctgacgcttgggataaggtataa

>fig|511678.20.peg.3830 Chitinase (EC 3.2.1.14) [Aliivibrio sp. EL58]

gtgataaaaataaataaaataacagccgctattactagcgttatgttcagtggataccta

tatgccgctgatgtaaatttaacgtctaacattgttacttttgttccaggtgagacaaaa

gttcaaaatggagagatggtttctttcaatggcgactgttttgttgccaaaaataaccca

ggcatatgggaatctccaaccgcagattcatggttctgggatactgcagtatgctcaggc

gaacctaatccagagccaacaccggatcccgatccgacgccagatcctgacccaactcca

gatcctgatttaggcgatgttattctatttattcctggtaaaactcaggtttcaaatggt

gatgtggtttcttatgacaaccagtgttttattgcgcaaaataatccgggtgtatgggaa

acgccaagtgctagttcttggttctggtcactaacagaatgttctgacgaacctgtaaat

cctgagcctgagatcactgaactatcgatccttgctccgacgacaggccaagtactaaca

gctaacgaagcaacctcaattactgctcacgttgatggtaacttagccgctaaagttgaa

ttttgggcaaacagcaccaaacttgctgaaaaaacagtaaaccaaaacaacactcaatac

tctcaaacttggacaccaagtaaagaaggtaatgcgacaattaacgtatttgtattcgat

aaaaataaccaaaaaatcgaacaaaaatcagtatctgttgttgtagaagctgaagtaact

gaagatttcgttgcacctgtagttaagtttgttactccaacaaacggctcggcaatcaac

gtaacagaaagcgttgctatctctgttaatgcaacagatgctgataacgatttagcaacg

ctggttgttactgcaaacaacaaagaaatctgttcgtttgacactgcgaataccaacact

tttgcgtgtgattttcagcctactcaagctggtaacgtaacactgaaagctatcgctact

gatgctcaagatctttcatctacagcaagtgtaaaaatcactgttgaagaagatgtggtt

gagccacctgtagagccgcctgtaactccaggtgacttatgtaaagactttaatgtttac

cctgactggacacgtggtgatcacgcgactggcggtgacattatggttaataaaaacatc

gcttactctgcgatctactggactaagtctgttcctggtagcgatgattcatgggcactt

catctaaattgtgatggtacagaaccgggtacagcaccgcttctttctctaccgaaccca

atggatccagttcgtctagaagtcgctggttggccaaacacgttagttgttgctagtcca

tcgtctgcggcaccagcaatgctaactattgaagcaagcaacagcgctgatttagccgat

ttaggtaagcttacaagcacgtttgtatctatcattaatgcagctgcacaagctggctct

gcatcaatcatcatcaacactgatgttcttgatcaagcaacgcaagataaagcattatct

tcaaacagcattgcggttaaagaagcgctaacacaagcgatggacatcactggcaacaag

attgacatcgatgatatcaacgctcttagcaatgacttgaaaggctgggctaatgcgcat

cacctaatcatctctacgctagcaccagaagcaaactacggttggtctctaagcattggt

gatttcgcatttgatactcactctggcagacaatctgtatgggataaagcatcaaactac

agcgctgatctattagacaaactagagttgttcaaagcagatgttgcaactaaagctgat

ttcattgcattcactaagtcaagctcaacagcagcactaaatagcgagcaatggcacaat

gcactagagtacgttaaacaagtttctgactttgttaaaacgcctgtaatgttaaacaat

atccctacggatcaagcttctgcttatttcatgggtgataatgcaagcaaaccacaagtg

cgtaaagctgcattcagcaacgtatttgctatcgtatttgataaagatacagcaaactta

actgctgaaatcgaagaatacaaaaaagctaaaatgccactgtactatgttggtgaatca

acagaaaatggtcaattaactatcattgatgcgttgaaccgtgaattggctgacgctgaa

aatacgatgaacaataccgcattcttgtacgaaacacctgaatcacaatgggttccgtct

actgtttataagtggactgacttcatgactggtctgaatgcaatgcataatgttggtgtt

gctggtaacaagttctggctactagatgaaaacgttgatgatgcaaccaacatcaaatac

gcgaaagtagcgattgctgcgttcttagcacaaagtatgcaagaaacgattcgttacaac

gcatgtgatgaaaacaactgggctgagattagatacggtgctgcaacagattacccaatg

tcagcgagctgtggtcaacttggtcaaaaatatgcagattacggtgtaaatcctgtttct

ggtttagatcacgcttactcttgtcctcgtgataacaaaatggaagtaagtgcactaact

cacgctaaatggtacggtgctccagctccagtatttgctgctccaaatgcagtgcttgaa

gagcgcggtttgcttgttaacggtagcgttggtcgttggactaacagcggtcattgtaac

gacgtacctacttctgtagatacatctaagcaagtatgggaacgtgatacatgtaaaaca

tacgtagagcaaaaagcaggtagctttatttgggatggcagcagccaagctgacgttgaa

ggttgtggttggtggggccgtggtgtaattcaaactactggtcgtcaaaacttcggtacg

cttaaccattacttaggccgctcacacgttgatcctgagacaattggtaaaacaattgat

ggtgttgttgttgaagcaccaccaaagaacccgttatacgctgagttagatttctgttca

aacccaggcttaatttgtagctctgaagagaacaaagaaatcaagtggattgcaggtcta

ttctactgggttacttcagtacaagcctaccctgatgagtctggtcagtacggtaactgg

aactaccacaacgagcttaagaaatatgttgatagcggtatgaaaggcactgactttatc

gatgatgtttctggtatcgtaaaccgtggttgtcctgatttagtgtgttctactggtgaa

gttcataacgtgaaagagcgtcgtgctaactttaaactagtactagagcaattaggtctt

agccctcaataa

>fig|6666666.334509.peg.2692 Chitinase (EC 3.2.1.14) [Aquimarina sp. Aq78]

atgaaattaaaattactcttaattccattcgttatctcctgctttttttggactcagaat

gggtttactcaatccgtatttattaatgaaatacactacgacaatgcaagtacagatgta

gaagaagctatcgaaattgccgggacagcaggaacagacctttctggttggagtattgtt

ttgtataatgggtctaatagcaccgtttataatacaatttctatttcgggagtgattcct

gatgagcaaaatggtttcggaacgatagtagaaattcttcctgccaatggattacaaaat

ggtgcacccgatgggattgcattaattgacaacaataatactgttatccagtttttaagc

tatgaaggtattattacagcaatagatgggcctgcatctgggttaacaagtaccgatata

ggtgtatcagaatctagcagtactcctgttggagcctctttacaactaacaggaacagga

acatctgctacagaatttacctgggaaacatctacaaccaactcatatggtgcgataaat

actaatcaggttttaggaacaccagttatcataccaataattaacgaatttgtatttaat

cataccggatcagatactgatgaatttgtagaaatattagctggtgcagaaacagactta

agcgaatattggttactagaaattgaaggagatagtaatgcttctggagttatcgatgaa

atactacaattaggctctacagatattaatggatatttcactactgcttttggtagtaat

gtatatgaaaatggtactgttactctacttttggtaaagaattttacgggtagtttagga

caagatcttgatacagatgacaatggtatctttgatgttaccccgtgggaagaacgcatt

gatgatattggagttaatgatggtggtgcatctgatcttaactatgccaatgttacctta

ctacaatcctttgatggaagctcatttactgttggaggagcatcgagatttccaaatggt

caagataccaatactgtcaccgattggaaaagaaatgattttgatggaagtggattacca

agcttccccgcagctatagcagaaccgggagaagctgttaatactcctaatcgagaaaat

gtagttattgatgatgttgatcctacagccgttatagtaattaatgaaattgatgcagat

accgatgggtctgatgttttagaatttgtagagctatttgacggcggagccggaaacaca

tctctagatggacacacattagtgttctttaacggatccaataatcagagttatgctgcc

tatgatttaaccggttctaccacaaatgctaatggatactttgttatcggtaatgcagat

gtagctaacgttgggattacgtttccaggtaatggactacaaaatggagcagatgctgta

gcactatataaaactgcaacttctaattttcctaacggaagccctattacaacagaaaat

ttggtagatgctattgtatacgacaccaatgatagtgatgatgtagaactattggtgcta

ctaaataatagtgaaccacaagtaaatgaagacgagaaaaacgataagaatttccattca

ttacaacgtttccctaatggatcaggaggattaagaaatacctcaacatacacacaagca

attcctactccgggaggagcaaataccaatgcaacagagatagttaatcttattattaac

gaactggatgcagatactcaaggctctgatgctttagagtttgtagaactatatgatggt

ggaaccggaaatacctcacttgatggttatgtattggtaaattataacggaagtaataat

accagttataacgccattgatttagatggttttactaccaatgccgaaggatattttgtg

gttggtaatgcagatgttgccaatgtaaaccttgtcgttcccggaaatacgttccaaaat

ggtgcagatgctgtagtcttgtattttggagatgctacaagcttccctaatggaacagcg

gttactaccgaaaatatcattgatgccattgtatatgataccgatgatgctgatgatcta

gaattattagtacttctaaatacagatcagccacaggtgaacgaaaacacaaatggaaac

aaagatggagagtctttacagcgaagtcctaatggacaaggaggagttcgaaatactgct

acatatgtcgctaaagctccaacaccaggagcagataacgatggtgtaatagtaactcct

ggtgacccaataagcattgcagaagctagagctgttgcagagggaactccggtaactatt

gcaggagtactaactgttaccgatagttttaatggtcctgcatttattcaggatgctact

ggtggtatcgctgtttttgatgaccaggtgcaagctaatacagctctaaaagttggagat

tctattaccataacaggagtcagagccgtttttaatgatcagattcaaattagttctgta

gctgaagtagtaaataacggtcttcctcaaaacccaattacaccactagacattacgctt

acacaacttgcagatcatccgggagaattggttcgagtacttaataccacattccctaat

ccaggagatcttttatttggaaactctaacttcacccttaccgatgctagcggaaacgga

gaactacgtgttgataacgacgtagcttctatagtaggtaaagcacaacctgttacctgt

tcagaaatcactggtgtgataggtcgttttagagaattcttccagttactgccaaggcaa

gcttcagatattccttgtgcggtagaatttattccaccaggagacacagtaggttttcct

aaagaagatacttttgatgtagtaacctggaatatcgaatggtttggtgatgaaaacaac

tcacctgtaggtcaaaaccctatgtctgatgcaattcaaagagatagtactgctacagta

cttaaaaaactaaaagcagatgtatatgctgtagaagaaattgctgatgatgtattattt

gaagaattagtaaacctgttaccagggtatgattatattttatccgatgccgtttctcgt

cctggttctggtggagtatcacaaaaagtaggatttatttataatactgaaaccgtttct

gtggtcgaaacaagagcaatgtttacatctattcatccgctatataatggtggagatgca

tctgcgcttgtagattaccctagcgaaacagatcgtttctatgctagtggaagattacca

tttttaatgactgcagatgtaaccattaatggagttacagaacgtattgatttgatcgca

ttacatgccagagcaaatagtagcaatggtcctcaaaatcgatatgatatgcgtaaatat

gatgtcgaagtattaaaagattcgctagatgctaattttgcaaacaacaaggttattctt

ttgggagattataatgacgatgtagatgaaaccgtagcagatatcccatcaaccatttct

agttttcaggaatatgtagatgacactgctaattacacgattgtatcttctgcactaagc

gaagcaggattgcgatcattcgtttttagagaaaatatgattgatcatatcatggtaacc

aatgaacttaatgaagcttatatcgaaaattctgtaactgttcattacgacgtatatgat

aatgactatgcattcactacatcggatcacttacccgtttctgcacgattcttgctagaa

ccagaatttgtaaataatgattgttcgggagcatctgtggtagcttttgatcaaggcaag

agaaaagatggaggaagaatatcccgacttagaagcaagactaagcgtgccctaggaact

cccagagaaaaaaggtattttaattttgtgagtttagggtttggtggatcgatcaccata

gaactaaacaacgaaatttttgataatgctggtactaatgaatttgcagtattcgaatct

acagggttctttgacaacgtcccttgtaattactatccagaatcagcagaagtatttgct

tctcaggatggtattgaatttgtttctttaggaacaacttgccaggatggagaatttgat

cttgcaacgggtaacttacgttctgcaaaatatatcaaagttgtggatacaagtgataaa

gctaattttccatggtttgcagatggctatgacttagatgctattgtatgtcttgaaaat

ggtgagcgtatcgctacaaaaaatactatggtctatacagaaaaccctactgctcttgaa

agtgaattactcaccaaggattttggattagaagaagcaagtgtttttgtttctccaaat

ccatttaaaaatcaattatctatagattttaatactcttgttgaaggagatgtagatgtt

ataattactgatgtaactggcaaaactgtatatactcaaacaatacaacttaatgccggg

caatcaaaattatctattgatatgaatcgttatccaaaagggttttatgttgtacatgct

ttcagtacaaatggaaaacttaatataactaaaaagataattaaaaaataa

>fig|6666666.334509.peg.3525 Chitinase (EC 3.2.1.14) [Aquimarina sp. Aq78]

atgtttaaaaaaagaacaaactcagtaaattttattacaattaattgctcatggaggcag

ttaatttgtacgaccttatttctaacagtatttctatctgtttttccccatggcacagta

acatacccgccaagtcgtgtttggaattgttttcaggaaaaccccgaaagtccagattct

gctgcttgtattgcagcagtagcctctcatggtacgcagccattatatgattggagtgaa

attaatcaagctaatgctaatggtaatcatcagcaatatgtgatggatggaaatctggct

agtggtggcaggcctgataaatatggaggcatggatcaagttagatctgattgggtgtcg

acaaaagtttctccaggcccatttacagttacttggacaaatcatgctcctcatgcaact

gcttattatgaagtttatataaccaaagcaagctggacacctgatcaaccattaacttgg

gatagtcttgaacttttggtacgaacatctccaagtgcaccagaaaggattgttaatatt

ccggtaacacttcctgctagaacaggtagacatgttatttatagtatttggcaaagatca

gacagtccagaggcattttattcgactagtgatatcaattttgatggaggagatactgat

acacaggcaccttctacacctactggtttaggggcatcaaatgctacacaaactacagtc

gatcttgcctggaatgcagctaccgataatgtagctgtaacgggatatgatatataccag

ggaaatactattgttgctaccgcaactggtacttctcatcaagtaactggattaacagca

aacacctcgtattcttttagaataaaagcaaaggatgctgcaaataatcaatctgatttt

agtaataccacaactgcaactacacttcctgatacaggaggaggaaattgtgcaggaata

tcacaatatgtagcaggaacttcttatagcaaagatcaggaagtacaaaatgcaggagaa

aaatttacatgtaatattccgggatggtgttcttctgcagcagcttgggcatatgcacca

ggtaccggagctcactggcaagatgcatggtcaaaaacaggtgattgcggtggcggtact

cctaacagcagtcctactgtaagtattacttcaccaaacgataatagctcgtttcaagaa

ggagtatctgtaacaataactgctaatgcagcagatacagatggggcagtgtctaaagtt

gaattttataacggtagtacaaaactgggagaagatacttctattccttatgaatatgtt

tggcaaaatgtttctgcaggaaattatgtactaacttccaaagcaaccgataaccaagga

gcatcgaccacatcttctgtaataaacattacagttaatggagttaataatactcctcca

tcagttgcaattacttctccaaataataatgattcttttaatgaagggacttcagtttct

atcactgcaaatgcttctgatagtgacggaactatttctaaagttgaattttataatgga

gctacaaaactaggagaagatacttctagcccttatggatatacaatttctaatgcgtct

gtaggaagttatacgttgactgctaaggctactgataatggcggagctacttcaacttct

tctgtagtatcaatttcggttactacagtaggaaatggtaattgcgatggattaccacaa

tatgttgcgggtacttcatatagcaaagatcaggaagtacaaaatgaaggcgaaaaattt

aaatgtaatattccgggatggtgctcttctgcagcggcatgggcatatgcacctggtact

ggagctcattggcaggatgcctggtctaaaacaggtgattgcggaggaggaacaggaggt

tctcctgtagtaaatatcacatctccttcaaatggtgcaacatataccgctggtagttct

gttgttgttaatgctacagcaactgatgatggaactgttactaaagtagaattctttgat

ggaactacaaaactaggagaagatactactaatccgtattcttttacaatttccaatgca

caatctgcaagctattctttaacagccgtagcaacagataatgagaataaccaaaccact

tcagaagttgttactgtaagaaaaagagtagatggtggaaataataacttaccaagtaag

attttggttgggtactggcataattttgataatggatctactacgccaagactgagtgaa

gtatccagagactgggatgtgatttgtattgcatttgcagagcctaaagcaggaagtaca

gccgatatgctatttagtccatatagtatttataatggaaatactcaggcttttatagat

gatgttactactgtaaagagtagaggacaaaaagtactaatttctattggaggtgccaat

gccagagtagaattaaataatgacactgaaaagaatttatttataagttctatgaccaat

atcatcaatacatatggttttaatggattggatatagatttagaaggaagttctctttca

ttaggtagcggagattcggatttcaggaatccgactacaacaaagattaagaatcttatt

gctgccacaaaagccattagaaccaatataggtgcaaatagatttatattaagtatggct

ccagaaacagcgtatgtacaaggtgcttatggaaattattctggaatttttggagcatat

ttacctgtaattcatgcattacgtaatgaaatggattatattcatgtacagcactataat

acaggttctatgtttgggggagatggaaaaatataccagccagctactgcagattttcat

gtggctatggcagaaatgttaattactggtttcccggttgctcaaacagggcttacattc

cctggtcttagagcagatcaggttgctattggattacctgctactacacaagcagcagga

agcggatatacttctgaagctgttgtgcaacaagcactggattatttgattaaaggaacc

tcttatccaggaagaacgtatacaaccagttctacgtatcctagttttagaggtttaatg

acctggtctataaattgggatttggtaaataattctgctttctcttcaagtcatagagca

tatttggatggattaggtgccagaactgctgtaattaatacacaaagtagtgtagggaaa

gtatttcctaatcctatttctggaaatataattaatgttgccttagatggttctatttct

aaatcaggttctgattatttcagattccagatatttaatactaatggagttgaagtttat

aattttcaaaatgacaagcttcaaagaggagaaagtgttaagagttttgatataggtgaa

ctagaatcaggaatgtatttttatacgatttcagtttccaaaaacaaaacaacaggcaaa

ataattagagagtaa

>fig|6666666.334509.peg.3526 Chitinase (EC 3.2.1.14) [Aquimarina sp. Aq78]

atgagaaaattaaaccaaacttttaaggcctggtgttttcaaaaattgcatttgaaaacg

tctttgcttttgatctttatgtgcatttcgagcattgcttttgcacaagtgaacacagga

gggagcgctacgacttctaatcatcagaaacaagttattggctatataactaactgggat

gcatggaaaaccactacagcaggagttcctgctccgggagctttaactcatcttaatatt

gattattctaaatacacgattcttaactattcattttttggagtagccagagatggttca

ttacatagtggagatcacagaaataaaaatatttatcaggatggagcaacacaagcacca

gcagatctattttttacagatttatatagtagctgggatttacacctcttattcggggag

ttagaatatgtaaactatgtaaatgctgatataaaagcacgtgcagaagctcaaggcttt

caagtagaagtaggggctagtacatggacacatcctgtctggggattaagtggcggattg

ccattacctcttaaaaaagaaaatggagcattaggattattagatcttgcccaccaaaaa

ggagtaaaagtaatggcatctataggtggatggagtatgtgtaaacatttcccagaaatg

gctgccgatcctgtaaaaagagcaaagtttattgaagactgtaaaagattaatcaatatt

ggtttcgacgggattgatttagactgggaatatcctgggccattttcaggaatgaatttt

acaggtagtcaggcagattttgcgaattttgaatctttattacaagaaattcgtaatgct

ataggaccttcaaaattaattacatcggctatggctgcagatccaagaaaactggatgga

tttaattggtctggagtagctgctaatatggattattttaacatgatgacttatgactat

aatggtggttggtctaataaagcaggacataatgcaccagtatatccgtatacaggagca

gaagtgcctttctttaactggcaatctacattagaaaaacttgttgaagtgggtgttcct

aaaaacaaaatatgttttggagccccattctacggtaggggtgtagtaacagaaggaaca

gctgatcttaatgcaacaacagtaaaacgatcagaaactgtacaacccgatggaccaata

caaaccgctgcagattatacaaattggccaaaagaagtgtatgatggtactcctaattac

ttttttatcaaacaaaaagccttatctcctaatagtggttggaccagaaaatgggataat

gaagctaaggttccttatttggtaaatggtaaatactttttaagctatgatgatgaagag

tctattgctattaaagctcaatttattaatgataatgggcttgccggaactatagtatgg

acagtttatggtgacttggaacttggaggtacagctacttcttttggtagaaaacttaaa

agatggtcagatgtgaaatctccattagttaacaaaataaatgaagtatttgccaatggt

ggtacaggaggaaatgtttctcctacggttagtataacagcaccagcaaataattctact

tttgccgaaggtgcaacaatagctataacggctaatgcatctgatagtgatggaacgatc

tctaaagttgaattctataatggaaccacaaaattaggagaagatactacatcaccatat

gagtattcgtggtctaatgttccagccgggagttatacaataacggccagagcaacagat

aatggtaatgctagcacgacttcttctgccgtttctgtatctgttggtaatagtacaaat

acaccgccaacagtaaatattacttcacctaataataatgattcttttacagcgggagtg

tcaatagctataacagctaatgcatcagatagtgatggaacgatctctaaagtcgagttt

tataatggaaccacaaagttaggagaagatactagcagcccatatggctatacaatttct

aatgcattagcaggtgattatacattaactgcaaaagcaacagataatgaaaatgcaacg

acaacttctactactatttctataacagttactagtgatacagggggatgtgcaaatata

ccacaatatgctgcgggtacttcatatagcaaagatcaggaagtacaaaacgaaggcgga

aaatttaaatgtaatgttccgggatggtgttcttctgcagcagcttgggcatatgcacca

ggtactggagctcactggcaagatgcctggtcaaaaacaggtgattgtagtggcgggact

cctaataccagtcctactgtaagtattacttcgccaagcgataatagctcgtttcaagaa

ggagtatctgtaacaataactgctaatgcagcagatgcagatggtacagtatctaaagtt

gaattttataacggtagtacaaaactaggagaagatacttctagcccttatgaatatgtt

tggcaaaatgtttctgcaggaaattatgtgctaactgctaaagcaactgataatcaagga

gcatcgaccacatcttctgtaataaacattacggttaatgggactaataactctcctcca

acggttgcaattacttctccaaataataatgattcttttaatgaaggagcttcagtttct

atcactgcaaatgcttctgatagtgacggaactatttctaaagttgaattttataatgga

gctacaaaattaggagaagatactagtagtccttatgcatatacaatttctaacgcatct

gtaggaaattatacattgacggctaaagctactgataatggtggagctacttcaacttct

tctgtgatatcaatttcagtaactacagtaggaaatggtaattgtgaaggattaccacaa

tatactgcaggtacttcttatagcaaagatcaggaagtgcaaaatgaaggcgaaaagttt

aaatgtaatgttccaggatggtgctcttcagcagcaggatgggcttatgcacctggtaca

ggagctcattggcaattagcttggtctaaaacaggagactgtgcagtaagaaattctgcg

gttagagtatttccgaatcctactgaaaatggtatcataaatgtgatgataaattctgga

aagtctaactctaattttagatttgaagtacattcccttagcggagctacattattagat

tttgaaaataatgtgatgaatggacggaatggtaaaacattcaatatcagttctttaaaa

tctggcctgtatttgtatacgattactataggaaatgaaaaggaatatggtaagatgaaa

gtatcaaattaa

>fig|6666666.334509.peg.3528 Chitinase (EC 3.2.1.14) [Aquimarina sp. Aq78]

atgaaaaattattatacatttttattaatgtttttttgtgcgctgtatattcaggcacaa

tacaatttccctacctgttctacagaatgggatgcaagtaaagttccttatacacaaggg

caagaagtttcttataataatattaattataagtgtaaatactataccagtgatgctcct

ggagctggttcctgggaactaataggaccatgtggtgatggaggactaggaccagactat

tctggtaaacaacgaattattgggtatttgcctacctgggttgctgattatgatatcaaa

aataaatttaatccagaagtagtaacacatctgaacatttcttttttaatgtttaaacag

aataataacgattataatagttcaaactttgcttctatatcttttgatgagtttcaatct

agaaaagtagattctgttcttaacgatttaggtgttcttcaaaaagctaaagccaaagga

gtaaaagtatctgttgcattaggtggagctacagattatgcatttctatggttgatgaac

aaataccaaaacaatgatagtaaactagatgaaatagcaacattgattgctaattatgtt

actcaaaatgatctggacggggttgatttggatatggaatgctggtgggcagatccagcc

attagtgggacttcagatcaaggaggaagagtaagaggaagcaaatggggagatgctgat

aaaggtcctcacccagcaggtgttggactaaaaaaattaagtcagaaactaagagctaaa

atacctaataaattaattacagctgccgtattcggaacatcttggtatggaaataactat

gatgatggtatggcagactatatggattggattggtcttatgtcttatgattttacagga

tcatgggataaatcaccagaaggccctcattcttctctttataaagtagaacaaggtact

tatcaaggacaaacagcagataatccaatttattctgcacaagatgcattagaatattgg

atggggtttgctccaccagcctggaatcatgctggaggatttaatgtaccaaaagccaaa

ttagcttttggattgcccgtttatggctatgatttttctgaaaagaaacctaatggaggt

aacggagccaaatttgttccttataaggatatcataaaagattttgctaatgcagctaca

agttatgaccctaaggatcctaaaaagttaagagggtacataggagagaatgggaaaaaa

atatattataacacaccaaaattagctggagaaaaaataaaatattctaagcagtatggg

catcaggggttaattatatgggaacttacacaagatgtcgattataattcgtcttcaagt

attcttaaagctgtaaatgaggctgcaggaaatacagatcctataaacaattcaccaaca

gtagtatgggaagctcccacaaatgagcaagttatcgaattaaccgaattgtctgcgata

gcattgaaagctagtgcaacagattctgatggaaccattcaatcttttacgtttaagcat

aacaatactaacattagtgctacagcaaatggaagtaattatacagccagttttactcct

gcagcatttggtgaagtaacgctcattgcttctgcaacagataataaaaatgcaacttca

gaaaaaacaattgtctttactgtaaaagaaaaggtagtaggaggtaatacacctccatca

ataactctaatcgaaccaaaagattcagatattatagagcaaacaacactgtcatcaatt

cagttaaaagctactgtaacagatgataatcaggttagctctgttaggtttattgtaaat

aatgttgaaattactccatctgcaaatggtactcaatacgttacagattggactcctggt

gcttttggtgatgtatcatataaaattacggctacagataatgaaggattatctacagaa

acagttgttatgtttactgtgaaagaaaaagtcataggaggtaactgtgatgggatagct

gcatggagatctgatcaaatatatgcgacagcgggacaaaaagtaagttataacggtaat

ctctataccaataaatggtggacacaaggagagactccgggaagcagttctgtatggggg

tttgtttctagttgtggtggaggctctggtagtggagatttttgtggatcttcacaatgg

gtagcatctattgcttataatagtggagatcaaatataccatagccaaaaaatttataaa

gccaaatggtggacagaaggcgaaactccaggtagtagttctgtttgggaatttgtttca

gattgtgtcaataataatccaaatatgtcttctgttgcatttcaaacattggtggatgat

gttataaagtataagataacagttccagaagtatcatgggtgaaaattgatgtatatgat

atctatggtaaattaatggatacaaaatctttaaaagagtatagtggaagtagagatttt

acacaagatttatctactctgaaaagtggaatttatatctataaaattaatatcggtgga

gaggtaatcaccaaaaaaataattaaaaaataa

>fig|6666666.334509.peg.3529 Chitinase (EC 3.2.1.14) [Aquimarina sp. Aq78]

atgatttttactgtattactttttgctattaatccaacgttttctaacgatgataataca

tatcccaggatatcaaaatcagattattcttttactccggataaatctttgaccgtaaaa

ccattatttcaagttaatgatttgataaataaagacttatgggatactttgtttccgtac

cgttttggcgccaaagacacaggaggcggtgtttgggtattagatcctaaagatgatttt

tatactttcgaatcttttatcgaggccattaatagaatgagtaaaattgaagtgaccttt

gagagaagatgtggtacgaacgcttatagagttacacgtactgataagactacaggtgta

tcaaaagtgattcgaaccgatgtagattttgatgcaccaagaaatgtagaaaaagaaatt

gtcattaaagaagtagattatggatcctttttgggagaaggaaatctggaaaccagaaaa

agagagattaccgctttttttgctaatatctctcacgagactaccggggggtggtcaact

gctccgggtggacagttttcttggggtttgcactttcgcgaagaacctacagatgcttct

tatgcttctccagatacaaattatcccccaactccgggaaaatcatataaaggaagagga

ccaatacagttatcatacaattataactacggtcctgccagtgaatttatttttggagac

aaacaaatactcttggataatccggaaaaagtaatcgaagatgctgctttagcttttcag

actgctatttggttttggatgacacctcaatatcctaagccatcggctcatgatgtgatg

gtgaacaaatggacccctaatgagttggataaaaccaaaaatagaattccaggacttggg

atgactgtaaatattattaatggaggagtagaatgtgggcaaggtacagaaaaacctcag

gtagtggatagaatagggtactaccaaagattcactgatatttataatataggaaccgat

atggatggagttcatgatctttctgattgtggatgtaaagatatgtctaagtatggtggt

gatgctgctgatcttacagcagaaccctgcgcacaaaaacctcaggtaacatttgctagc

ccgaagaataatcaaatgtttgagcaaactacattttcgccaatttcggtaagcttgtct

atagacgaaaaaaacacaaaattagtaagcgttactacatctgtagaaaatcaaactttt

gatggagtaacattcagttggacaccatctagttatacaagtcacgttttaaccgctaat

gctgtatttgaaaatggtattaccgcaacttcaaccataaagattattatttgggatgga

gtgaatattgactgtcaagaagtgcccgaatggaatgcttcaagaatttataaagacaag

aacaactatgtgaagtacaataacaaagtctataaaaacaaatggtatgcagatagtagt

aatatcccaggaagtgataccgtatgggagtttgtaaaagaatgtggtgcttctaatgga

actaaccctgtaataacatgggaatcaccaggtaatggacaggtcatagaacaaaaagaa

cttacaccaattactttaaaagccagtgctacagataccgatggaactgttcaatctttt

gtctttaaatataacaatactagtattaatcctacctcttcagggaataactatacagct

agttttacgccaactgcatttggagaaattacgattacagcatctgctacagatgatcaa

aataatacttcagaaaaagcaatttcttttactgtgaaagaaaaaacgacaggaggaaat

aataatgctccagttgtaagtataacttctccgggtgataatacttcttttgaagcagga

acttcgattcctatcacggtaagtgcgtctgacaatgatggtacgattacaaaagtaact

ttttttaataatggaaataagataggagaaagcttaagtagcccttatacatatacaatt

actaatgcagtagaaggaaattatgtattaacagccgaagctacagatgatgacggtgct

tcttcaacttcttcttcaatttcagtttcggttactacggtgggtaatggtaactgtgcg

aatgtacctcaatatgtagcaggtacttcttatggattaaatgatgaagtagtaaatgaa

ggagaaaaatttgaatgtaacatcccgggatggtgctcatctgctgctgcttgggcatat

gcacctggtactggagctcattggcaacttgcctggacaaaaacaggaacttgttctaaa

agtaactctgaaataacttcaaatgattactctgtttttcctactgtaactcaggatatt

gtaaactttaggataaaaacagataatacatcctggattaggattaatctataccacctt

tctggaaaattaataagtacacaatcatttaatggaatccagacaaagtcagtaaaatca

tttacacacgatctgtcaaatctcaaaaacggtctttatgtatttaaaatttacataaat

gacgacgtttattttgagaaaatccttaaaaattaa

>fig|6666666.334509.peg.3542 Chitinase (EC 3.2.1.14) [Aquimarina sp. Aq78]

atgaaattacttaaaagattatcagtcgtcttattcattcaggtttttttattttcctgt

acgaatgaaagttttatagaagaaggggctctggaggctcaaaattctgaaacaagtcta

acagacaaggcagcactcacagaacctattgtcttaggatatttcccttcctggtcagaa

agttgggccggacctgggcaaggatcaaaactaagagatatcccagaacatatcactcac

gtttttttggcatttgcaaagcctaatcttcgttatcaaaaaggttctttagatattacc

aataccggtattcaaactccttacggaggggctactttaaaagaatctgttgcagctcta

aaatcaaaaggcatcaaagttattttatctgtaggtggagaaacgtattggggtacagat

gcagcttatgatataaattatcagcagatcaaagatctggtagatgacatgggatttgaa

ggtatcgattgggattttgaacccaatggtagttttgccaccataggagaacctataaat

gtacaacgcttcatcgatttctttacaaattcaagagctattatgccaaaaggacaatac

ttattagcctgtgctcctgcgggagttggagctttaggaggtgcaaataataatgacccc

tcctctcctttcgcctatagtaaaagaaacacagtaactggagagtccgatactaactta

ttcaatgctacttcacctaaccaaggcattagtttatttggttttgctactacagggcat

atgattcctgtaatggaagctgttggtgataaaattgatcttattgcatatcaaggatat

aatacgggagctgccagtaatagaaaaattatgtatgatgcatataaacactactcaaac

cagtatggcttttctattgcagccggagttcattatcctaatgaaccttgggggccttat

tatgaatatacctatcaaaatatagcagagctatctaaccatatttctgtaaataacagc

tctaatgatggagttatgatatggcaattattacttggtaatacgacttcttctgcatat

ggatatcttcatgttgcaagtcaggtacttaatggtgtatcacaatctcaggccatagcc

aatgcagaaaactatccagaatctccatactctggcgatggcggtggaggtggtggatca

ggctgtgaatctgcgccatggaatgcttctgctgtttataacactggtcaggaagttgtg

cataacaataaattatatcgtgcaaaatggtggacgcaaaatgataatccatcatcaaat

tctggagcaggattgccatgggaatttgttcagaattgtaatggtaacggaggaggaaac

aaccaatctccttctgtatctatcacatctcctacaaataaccaatcggtaaccgagggg

cagtctatcaatattacagcaaatgcttctgataccgatgggacaattactaaagttgaa

ttttttcaaggaactactaaacttggagaagacacaacaagtccatatagctattcctgg

aataatgctgctgtaggaagttatagcctaagtgctgttgctacagataatgctaatgca

acatctacatcggctacagtatccattattattacttcttctggtggtggtaacggtggt

tcttgtagcgggattgcaaattgggaagcatatccaacagtatataatgtgggagataaa

gtagtataccaaggtacattatacgaagctcagtcagggccaatatgggtaaccccaggt

agtggtgaacactggtggaaaactataggaacctgtaactaa

>fig|6666666.86154.peg.517 Chitinase (EC 3.2.1.14) [Aquimarina sp 349]

atgaaattacttaaaagattatcagtcgtcttactcatacaggtattcttattttcctgt

acgaatgaaagttttttagaagaaggagccgcagaggttcaaaattctgaaacaaatcta

acagacaaaacagcgctcacagaaccaatagtcttaggatatttcccttcctggtccgaa

agctgggctggtcctgggcaaggatcaaaactaagagatatcccagaacacatcactcac

gtttttttggcctttgcaaaacctaatcttcggtatcaaaaaggctctttagatattacc

aatacaggtattcaaactccgtacggaggggatactttaaaagaatctgttgcagctcta

aaatcaaaaggaatcaaagttattctatctgtaggtggagaaacgtactggggtacagat

gcagcttatgatatcaattatcaacagatcaaagatcttgtagatgatatgggattcgaa

ggtatcgattgggattttgaacctaatggtagttttgccactataggtgatcccataaat

gtacaacgctttatcgatttctttacaaattcgagagctattatgcctaaaggacaatat

ctattagcttgtgctcctgccggtgttggagccttaggtggtgcaaataatgatgacccc

tcctctccttttgcctatagcaaaagaaacacagtaacaggagaatctgatactaatctc

tttaatgcaacttctcctaatcaagccattagtttatttggttttgctactaccgggcat

atgatccctgtaatggaggctgttggtgacaaaattgatcttattgcatatcaaggatat

aatacaggagctgccagtaatagaaaaattatgtatgatgcttataaacattattcaagt

cagtatgggttttctattgcagccggtgttcattatcctaacgagccttggggaccatat

tatgaatatacttatcaaaacttagcagagctatctaaccatattgctgtaaataacacc

tctaatgatggagttatgatatggcagttattactgggtaatacgacttcatcggcatat

ggatatcttcatgtcgcgagccaggtactaaatggcacatcacaatctcaagctatagct

aatgcagaaaactatcccgaatcaccatacactggtgatggaggcggaggcggaggatcg

ggctgcgaatctgctccatggaatatttctgttacttataacactggtcaggaggttgtg

cataataataaattatatcgcgcaaaatggtggacccaaaaagataatccatcatcaaat

tctggagacggattaccatgggaattcgttcaggattgtaatggtaacgggggaggaaac

aatcaatctccatctgtatctattacatctcctacaaataatcaatctataaccgaaggg

caatctatcaatattacagcaaatgcttctgatgctgatggaacaattacaaaggttgaa

ttttttcaaggaactactaaactcggagaagacacaacaagcccgtatagctattcctgg

aataatgctcctgtaggaaattatactttaagtgctattgccacagataatgctagtgca

acaactacatcggctacagtatctatcaccattacttcttctggtggtggtaacggtggt

tcttgtagtggtattgcaaattgggaagcatatccaacagtatataatgtgggagacaaa

gtagtatatcaaggtacattatacgaagctcaatcagggccaatatgggtaaccccaggt

agtggagaacactggtggaaaaccataggtacctgtaattaa

>fig|6666666.86154.peg.3255 Chitinase (EC 3.2.1.14) [Aquimarina sp 349]

ttgacagctaaggctactgataatggaggagctacttcaacttcttctgcgatatcaatt

tcggtgactacagtaggaaatggtaattgtgatgggttaccacaatatgttgcaggaact

tcatatagtaaagatcaggaagtacaaaatgaaggagagaagtttaaatgtaatattccg

gggtggtgttcttctgcggcagcatgggcatatgcaccaggtactggagctcattggcaa

tcagcttggtcaaagacaggtgattgtggaggaggaacaggaggtgctcctgtagtaaat

atcacatctccttcaaatggtgcaacatatactgctgatagttctgttgttgttaatgca

accgcaaccgatgatggaaccgttactaaagtagaattctttaatggaactacaaaacta

ggagaagatactactaatccgtattcttatacaattaccaacgcacaatctggtagttat

tctttaactgcagtagcaaccgataatgagaataatcaaaccacttctgatccagttgta

attagaagcagtacaggaggaggaaataataatttaccaggaaaaatactggtagggtac

tggcataattttgataatggttctactacaccaagattgagcgaggtatccagagattgg

gatgtgatttgtgttgcatttgcagaacctaaagcgggaagtacagccgatatgctattt

agtccatatagtatttataatggaaatactcaggcttttatagatgatgttgctactgta

aagagtagaggacaaaaagtactaatttctattggaggtgctaatgcaagagtagaattg

aataatgaaactgaaaagaatttattcgtaagttctatgactaatattatcaatacatat

ggctttaatggattggatatagatttagaaggaagctccctttcactaggtagcggagat

tctgatttcaggaatccaactacagcaaagatcaaaaatcttattgctgctacaaaagcc

attagaaccaatataggtgcaaatagatttatattaagtatggctccagaaacggcgtat

gtacaaggtgcatatgggaattattctggaatttttggagcatatttacctgttattcat

gcactacgtaatgaaatggattatattcatgtacagcactataatacaggttctatgttt

ggaggagatggaaaaatataccagccagccactgcagattttcatgtggctatggcagaa

atgttaattactggtttcccagttgcacaaactggacttacatttcctggacttagagca

gatcaggttgctattggattacctgctactactcaagcagcaggaagcggatatacttct

gaagctgttgtacaacaagcactggattatttgattaaaggaacctcttatccaggaaga

acgtatacaactagttctacatatcctagttttagaggtttaatgacctggtctattaat

tgggatttggtaaataattctaccttctcttcaagccatagagcatatttggatggatta

ggtgccagagctgctaatgcacaaaatagtgtagggaaagtatttcctaatcctatttct

ggaaatataattaatgttgccttagatggttctatttctaaatcaggttctgattatttc

agattccagatatttaatactaatggaattgaagtttataattttcagaatgacaggctt

caaagaggagaaagtgttaagagttttgatatcggtgaactagaatcgggaatgtatttt

tatacgatttcagtttccaaaaacaaaacaacaggtaaaataattagagagtaa

>fig|6666666.86154.peg.3595 Chitinase (EC 3.2.1.14) [Aquimarina sp 349]

atgaaattaaaattactcttaattccattcgccttttcctgccttttagggactcagaat

gggtttactcaatccgtatttattaatgaaatacattatgacaatgcaagtacagatgta

gaagaagctgttgaaattgctggaacagcaggaacagatctttctggttggagtattgtt

ttgtataacggttcaaatagtaccgtttataatacaatttctatttcgggagtgattccc

gatcaacaaaatggtttcggaacgataatagaaattcttcctaccaatggattacaaaat

ggtgctcccgacgggatcgcattaattgataacaacaatgcagttgtacagtttttaagc

tatgaaggcgttattacagcaacaaatggtcctgcatctggattaacaagtaccgatatc

ggtgtgtcagaatctagtagtactcccgttggagcttctttacagttatcaggaacagga

acatcggcaacagaatttacatgggaaatatctacaaccaactcatatggtgccgtaaat

attaatcaggttttgggaacaccagttatcatacccatgattaatgagtttgtatgtaat

cataccggatcagatacagatgaatttgtagaaatattagctggtatagaaacagactta

agcgaatattggttgctagaaattgaaggagatagtaatgcatctggagttgttgacgaa

gtaatacaattaggttctacagatgttaatgggtattttactactgcttttggtagtaat

gtatacgaaaacggtacagtcactctcctcttggtaaagaattttacgggtagcttaggg

caagatcttgataccgatgacgatggtgtttttgatatcactccctgggaagaacgtatt

gatgatgttggagttaatgatggtggtgcatctgatcttaattatgctaatgttacctta

ctacaatcttttgatggaagctcatttactgtaggaggagcatcaagatttccgaatggt

caagataccgatactgtcaccgattggaaaaggaatgattttgatggaagtggattgcca

agttttcctacagttatagcagaaccaggagaagctgttaatacgcctaatagagaaaat

gtagttatcgatgatactaatccaacagcggtaatagtaattaatgaaattgatgcagat

accgatggatctgatgttttagaatttgtagagctgtttgatggtggagccggaaacaca

tctctggatggatacacattagtattctttaacggatctaataatcagagttatgctgcc

tatgatttaacaggttctaccacaaatactaatggatattttgttatcggtaatgcagat

gttgctaatgttgggattacatttcctggtaatgggttacaaaatggagccgatgccgta

gcactttataaaactgcagcctctaattttcctaacggaagtgctgttacaacagaaaat

ctggtagatgcaattgtatacgataccaatgatagtgatgatgcagaactattggtgtta

ttaaatagtggtgagtcacaaataaatgaagacgagaaaaatgataagaacttccattcc

ttacaacgttttcctaatggatctggaggattaagaaatacctcaacatatacacaggca

attcctacaccaggaagtgcaaatacaaatgcaacagaaatagttaaccttattatcaac

gagttggatgcagatactcaaggttctgatgctttagaatttgttgaactctatgatggt

ggagccggaaatacctcacttaacggttacgtattggtaaattataacggaagtaataac

accagttataatgccattgatttagatggttttactaccaatgcagaaggatattttgtt

attggtaatgcagatgttactaatgtaggccttgttgttcccggaaatacattccagaat

ggtgctgatgctgtagtcttatactttggtgatgcgacaagtttccctaatggaacagtg

gttactaccgaaaacattattgatgctattgtatatgataccgatgatgcggatgatgta

gaattattggtacttctaaatacagatcagccacaggtaaacgaaaactcaaacggaaac

aaagatggagaatccttacaacgaagtcctaatggacaaggaggggctcgaaatactact

tcatatgtagccaaagctccaacaccaggcgcagataacgatggtgtaatagtaattcct

ggtgacccaataagcattgcagaagctagagctactgccgagggtacaccagttactatt

gccggagtattaaccgttaccgatagttttaatggtcctgcatttatacaagataccact

ggtggtatcgctgtttttgatgaccaggtacaagctaatgctactttaaaagttggggat

tctattaccataacagggatcagggctgcttttaatgatcagattcagattagttctgta

acagatgtagtaaataatggtcttcctcaaaacccaatcacaccactagacattacactt

gcagaacttgcagatcatccaggagaattggttcgagtacttaatactacattccctaat

ccgggagatcttttattcggtaactctaacttcactcttaccgatgctagtggaaatgga

gaactacgtgttgataacgacgtagcttctatagtaggtaaggcacaacctgttacttgc

acagaaatcacaggtgtgataggtcgttttagagaattcttccagttactgccaagaaaa

tcttcagatattccttgtgcggtagaatttattcctccaggagacacagtaggctttcct

aaagaagatacttttgatgtagtaacctggaatatcgaatggtttggtgatgaaaacaac

tcacctgtaggtcaaaaccctatgtctgatgaaattcaaagagatagtaccgctacagta

cttaaaaagctaaaagcagatgtatatgccgtagaagaaattgccgatgatgtattgttt

gaagaattagtaaacctgttaccaggttatgagtatattttatctgatgctgtttctcgc

cctggttctggaggagtatcacaaaaagtaggatttatttataatactgaaaccgtttct

gtggtcgaaacaagagcaatgtttacatctattcatccactatacaacggtggtgatgca

tctgcgcttgtagattatcctagcgaaacagatcgtttctacgctagtggaagattgcct

tttttaatgaccgcaaatgtaactattaatggagttacagaacgtattgatttgatcgca

ttacatgccagagcaaatagtagcaatggtcctcaaaatcgatatgatatgcgtaaatat

gatgtagaagtattaaaagattcgctagatgctaattttgcaaacaacaaggttattctt

ttgggagattataatgacgatgtagatgaaaccgtagcagacatcccatcaaccatttct

agttttcaagaatatgtagatgacactactaattacacgattgtatcttctgcattgagc

gaagcaggattgcgatcattcgtttttagagaaaatatgattgatcatattatggtaacc

aatgaacttaatgaggcctatatcgagaattctgtaactgttcattacgacgtatatgat

aatgattatgcattcactacatcagatcacttgcctgtttctgcgcgattcttgctagaa

ccagaatttgtgaacaatgattgttcgggtgcatctgtagtagcctttaatcaaggcaag

agaaaagatggtggaagaatatcccgacttagaagcaagactaagcgtgccctaggaact

cccagagaaaaaaggtattttaattttgtgagtttaggttttggtggatcaatcaccata

gaactaaacaacgaaatttttgataatgcgggtaccaatgaattcgcagtattcgaatct

acagggttctttgataatatcccttgtaattactatccagaatcggcagaagtatttgct

tcacaagatggtattgagtttgtttctttaggaacaacttgccaggatggagaatttgat

cttgcaacaggtaatttacgttctgcaaaatatatcaaggttgtggatacaagtgataaa

gctaattttccttggtttgcagatggctatgatctagatgctattgtatgtcttgagaat

ggtgagcgtatcgctacaaaaaataccatggtctatgcagaaaaccccactgctctagaa

agtgagttactcaccaaagatttcgggttagaagaaacaagtatttttgtttctccaaac

ccatttaaaaatcagttatctatagattttaaaactctggttgaaggagatgtagatatc

acaattaccgatgtaacaggtaaaacggtatatactcaaacgatacaacttaatgtcgga

caatctaaattatcaatcgatatgaatcgttatacaaaaggattttatgtagtacatgct

tctagtacaaatggaaaacttaatataacccaaaagataattaagaaataa

>fig|6666666.86154.peg.5394 Chitinase (EC 3.2.1.14) [Aquimarina sp 349]

atgacacacaaactcaccactccaaaaaaccaaaaggttttaggtaggaaatcatggatt

atgatttttactgtattactttttactattcacctaaccttttctaacgatgataataca

tatcccgagatatcaaaaacagttgattctttctctctggataaacctttacctgtaaat

ccactatatcaagttaatgatttgataaataaagatttatggaacactttgtttccgtat

cgttttggtgccaaagacacaggaggtggtgtttgggtactagacccaaaagatgatttt

tatactttcgaatcttttatcgaggccattaatagaatgagcaaaatcgaagtaaccttt

gatagaagatgtggtacgaacgcttatagagttacacgtactgataagactacaggtgta

tcaaaagtgattcgaaccgatgtagattttgatgcaccaagaaatgctgataaagaaatt

gttaccaaaaaagtagattatggatcttttttaggagaaggaagtctcgagaccagaaaa

agagaaattacagctttttttgctaatatctctcacgagactaccggggggtggtcaact

gctccaggtggacagttttcttggggtttacactttcgtgaagaacctacagatgcttct

tatgcttctccagatacaaattatcctccaactccggggaaatcatataaaggaagaggc

cccatacagttatcatacaattataattatggccctgccagtgaattcatttttggagat

aagcaaatacttttggatcatccggaaaaagtaatcgaagatgctgctttggcttttcag

actgctatttggttttggatgacgcctcaatatcccaaaccatcagctcatgatgtaatg

gtgaacaaatggactcctaatgagttagacaaaacaaaaaatagaattccgggattaggg

atgactgtaaatattattaatggaggcgtagaatgtggacaaggtacagaaaaacctcag

gtgttagatagaataggatactacgaaaggtttacgggtatttaccaaatagggacagat

atggatggagttcatgatctttctgattgtggatgtaaagatatgtctaagtatggggga

gactctgctgatttaaccgcagaaccctgtgcacaaaaacctcaggtaacatttaccaat

cccagaaataatcaaatgtttgagcaatctacattttctccaatttctgtaagtttatct

atagacgaaaaaaatacaaaactagtaagtgttactaccgctatagggaatcaaactttt

gatggagtaacatttagttggacaccatctagctatgcaagccatgttttaagcgctaat

gcggtattcgaaaatggtataacggcaacatcagaaattaagattattatttgggatgga

gtaaatcttgactgccaggaagtgcccgaatggaatgcttcaaggatttataaggacaag

aataactatgtgaaatataacaataatgtttataaaaataaatggtatgccgatagtagt

aatgtcccgggaagcgatactgtatgggaatttgtaaaagaatgcggtgtttctaatggg

agtagtcctgtgataacttgggaatcgccaggtaatggacaagttatagaacaaaatgaa

cttgcaccgattactttaaaagccagtgctactgataccgatggaactgttcaatctttt

gtctttaagtataataatgctaatattactcctactgcttcaggagatatctataccgct

agttttacgccaactgcatttggagaaattactattacagcatctgccacagatgatcaa

aataatacctcagaaaaagcaatttcttttactgtcaaagaaaaaacaacaggaggaaat

aataaacctcccgttgttagtataacttctccgggagataatgcctcttttgaagaaggc

acttcgattcctgtcacggtaaatgcatctgatagcgacggcaccatcacgaaagtagaa

ttctttaataataatagtaaaataggagaaagtagcgctagcccttttagttatacactc

gaaaacgttatagtaggaaattactcgttaacggcaaaagcaacagacaataaaggggca

ttttcaacctcttcagcaatagcaattacagtaactagtggcggaggaaatgggaattgt

gcagatatacaacagtatgttgcaggtacttcttatggattaaatgatgaggtagtaaat

gaaggagaaaaatttagttgtgatattccgggatggtgttcatctactgcagcatgggca

tatgcccccggtacaggagcgcattggcaaatggcctggacaaaaattggagcttgtggt

aaaaataattctgaaataacttcaaataagtactctgtttttcctacggtaacccaggat

attgtaaattttagaataaaaacagataatacatcatgggttaagattaacctatatcat

ctttctggaaaattgataagcacacaatcatttaatggaatccagacaaagacactaaaa

tcatttacacatgatctgtcaaatctcaaaaatggtctttatgtatttaaaatatatata

aatgacgatgtttattttgagaaaatccttaaaaattaa

>fig|6666666.86154.peg.5395 Chitinase (EC 3.2.1.14) [Aquimarina sp 349]

atgcacgcacaatacaattttcctacctgctctgcagagtgggatgcaagtaaagtccct

tataaacaaggacaagaagtttcttataataatgttaattataagtgcaaatactataca

aatgatgctccgggagccggctcctgggaactaataggaccatgtggtgatggaggatta

ggtccggattattctggtaaacaacgaattatagggtatctgcctacctgggttgctgat

tatgatatcaaaaataaatttaatccagaggtagtaacacatcttaatatttcttttttg

atgtttaagcaaaataataacgattataatagttcaaattttgcttctatatcttttgat

gagtttcaatctagaaaagtagattctgttcttaacgatttgggagttcttcaaaaagct

aaagccaaaggagtaaaagtatctgttgcattaggaggagctaccgattatgcatttcta

tggttaatgaccaaatatcaaaacaatgatagcaaactagatgaaattgcaacattgatt

gctaattatgttactcaaaatgatttagatggtgttgatttggatatggaatgttggtgg

gcagacccggccattagtgggacttcagaccagggaggaagagtaagaggaagcaaatgg

ggagatgctgataaaggtcctcaccccgcgggtattgggttaacaaaattaagccagaaa

ttaagagccaaaatacctaataagttaattacagctgctgtttttggaacttcatggtat

ggaaataactatgatgatggtatggcagaccatatggattggattggtcttatgtcttat

gattttacaggatcatgggataaatctccagaaggacctcattcttctctttataaagta

gaacaagggacttatcaaggacaaacagcagataatccaatttattctgcccaagatgca

ttagaatattggatgggatttgctccaccagcctggaatcatgcaggaggatttaatgta

ccaaaagctaaattagctttcgggttaccagtatatggttatgatttttctgaaaaaaag

cctgatggagggaatggagctaaatttgttccttataaggatatcataaaagattttgct

aatgcagctacaagttatgaccctaaggatcctaaaaaattaagaggatacataggagaa

aatggaaaaaaaatatactataacacaccaaaattagctgcagaaaaaattaaatattct

aaacagtatgggcatcagggattaattatatgggaacttacacaagataccgattataat

tcgtcttcaagtattcttaaagcagtaaatgaggctgcaggaaacactgatcctataaac

aattcaccaacagtagtgtgggaagctcccacaaatggacaagttatcgaattggaagaa

ttgtctccaataacattaaaagccagcgctacagattcagatgggactattcaatctttt

gtgtttaagcacaatacgactaacattagtgctacagcaaatggaagtagctatacagcc

agttttactcctgcagcatttggtgaagtaacgctcattgcttctgctacagataataaa

aatgcaatttcagaaaagacaattgtttttacagtaaagaagaaagtagtgggaggtaat

acacctccatcgataactctaatcgaaccaaaaaatgcagatgttatagaacaaacagca

ttgtcatcaattcagttaaaagctacagtaacagatgatacccaggtaagctctgttaaa

tttgttgtaaataatattgaaattactccaactgtaaatgggactcaatatgttacagac

tggactcccagcgcttttggtgaagtatcctataaaattatgggtacagataatgaagga

ctatctacagaggtggttgttacgtttactgtgaaagaaaaagtagtgggaggaagttgt

gatggaatagcagaatggcaatctgataaggtatatgctacagcaggacaaaaagtaagt

tataacgggaatctctataccaataaatggtggacgaaaggggagactccgggaagtagc

tctgtttgggaatttgtttctagttgtaatggtggttctggtggcggagatttttgtgga

tctccacaatggataacatctattgcttataatagcggggatcaagtataccatagccaa

aaaatctataaagccaaatggtggacagaaggtgaaactcctggcagtagttctgtgtgg

gagtttgtttcagactgtgttcagagtaatccaaatatgtcttctgttgcatttcaaacg

ttggtagatgatgttataaaatatcagataacagtttctgaagtatcatggataaaaatt

gatgtatatgatatctatggtaaattaatgcacacaaaatctttaagagaatatacagga

agtagagattttacacaagatttatcgactcttaaaagtggaatctatatctacaaaata

aatataggcggagaagtaatcaccaaaaaagtaattaaaaaataa

>fig|6666666.86154.peg.5397 Chitinase (EC 3.2.1.14) [Aquimarina sp 349]

gtgaacacgggaggaagtgctaccacttctgatcatcaaaaacagattatagggtatatt

accaactgggacgcctggaaaaccaatactgcaggagtacctgaagccggagctcttaca

catttaaatattgattattctaaatacacgattctaaattattcattttttggagtggct

cgtgatggttctttacatagtggcgatcatagaaataagaatatatatcaagatggtgta

acacaagaacctgctgatttattttatacagatttgtatagtagttgggatttacacctc

ttattcggggagttagaatatgtaaactatgtaaacgctgatataaaagcacgggcagaa

gctcaaggctttcaagtagaagtaggagctagtacatggacacatcctgtctggggacta

agcggaggattaccattacctcttaaaaaagaaaatggagctctggggttactagatctt

gcacatcaaaaaggagtaaaagtaatggcctctataggaggatggagtatgtgtaaacat

ttcccagaaatggctgccgatcctgtaaaaagagcaaaatttattgaagactgtaaaaga

ttaatcaatattggtttcgacggaattgatttagactgggaatatccaggaccattttca

ggaatgaattttacaggtagtcaggcagattttgcgaattttgaatctttactacaagaa

attcgtaatgctataggacctacaaaattaattacatcggctatggctgcagatccaaga

aaactggatggatttaattggtctggagtagttgctaatatggattactttaacatgatg

acttatgattataatggtggttggtctaataaagcaggacataatgcaccagtatatccg

tatacaggagcagaagtacctttctttaactggcaatctactttacaaaaacttgttgaa

gcaggagttcctaaaaacaaaatatgttttggagccccattctacggcaggggtgtggta

acagaaggaactgctgatcttaattcaacaacagtaaaacgatcagaaactgtacaacca

gatggaccaatacaaaccgctgcagattatacaaattggccgaaagaagtttatgatggt

actcctaattacttttttatcaaacaaaaagccttatctcctaatagtggttggaccaga

aaatgggataatgaagctaaggttccttatttggtaaatggtaaatattttttaagctat

gatgatgaagagtctattgctattaaagctcagtttattaatgataatgagcttgccggg

actattatttggacagtttatggtgatcttgaatttggaggtacagcaacttcttttggt

agaaaacttaaaagatggtctaatgtaaaatctcctttggtgaataaaatcaatgaagtt

tttgcaaatggagggccaggaggaaatgtttctcctacagttaatataacagcaccagct

aataattctacttttgctgaaggcgcaacaatagctataacagctaatgcatctgatagt

gatggaactattgctaaagttgaattctataatggaaccacaaaattaggagaggacact

acatcaccatatgagtattcgtggtctaatgttcctgccgggagttatacaataacggca

agagcaacagataatggtaatgctagcacgacttcttctgccgtttctgtatctgttggt

aatagtacaaatacaccgccaacagtaaatattacttcacctaataataacgattcgttt

acagcgggagtatcaatagctataactgctaatgcatctgatagtgatggaacgatctct

aaagttgaattctataacggaaccataaaattaggagaagataccagcagcccttatggg

tatacaatttctaatgcatcagcaggtgattatacattgactgcaaaagcaacagataat

gagaatgcaacgacaacttccactactatttctataacagttactagtgatacaggagga

tgtaccaatataccacaatatgtagcaggtacttcttatagcaaagatcaggaagtgcaa

aacgaaggtggaaaatttacatgtaatgttccgggatggtgttcttctgcagcagcttgg

gcatatgcgccaggtactggagttcactggcaagatgcgtggtcaaaaacaggagattgt

agtggcggtactcctaatacgagtcctactgtaagtattacttcaccaagcgataatagc

tcgtttcaagaaggagcgtctgtaacaataactgctaatgcagcagatgcagatggtaca

gtatctaaagttgaattttataacggtagtacaaaattaggagaagatacttctagtcct

tatgaatatgtttggcaaaatgtttctgcaggaaattatgcaataacagccaaagcaacc

gataatcagggtgcatcgaccacatcttctgtaataaatattaccgttaatggagttaat

aatactcctccaacagttgcgattacttcaccaaataataatgactcttttaacgaagga

acttcaatctctattacggcaaatgcttctgatagtgacgggactattactaaagtagaa

ttctttaacggaaccacaaaactgggagaagatactagtagtccttatgcatatacaatt

tctaacgcatctgtaggaaattatacattgacagctaaggctactgataatggaggagct

acttcaacttcttctgcgatatcaatttcggtgactacagtaggaaatggtaattgtgaa

ggattaccacaatatgctgcaggtacttcatatagcaaagatcaggaagtgcaaaatgga

ggcgaaaagtttaaatgtaatgttccaggatggtgctcttcagcagcagcatgggcttat

gcacctggtacaggagctcattggcaattagcttggtcaaagacaggagactgtacagta

agaaattctacggttagcgtatttccgaatcctactgaaaatggtatcataaatgtgatg

ataaattctggaaagtctaactctaaatttagatttgaagtacattcccttaacggaact

aaattattagattttgaaaataatgtgatgaatgggcagaatggtaaaacatttgatatt

agttctctaaaaactggtttgtacttatatacgattactataggaaaagaaaaagaatat

ggtaaaatgaaagtatcaaattaa

>fig|290174.3.peg.968 Chitinase (EC 3.2.1.14) [Aquimarina sp 135]

atggtactagcctttattcttggctacaccagtatcgctcagataaactctggaggaagc

cacacaacagccaaccattctaaacaagtaatagggtatgttcctaattgggacgcatgg

aaagactctaaatttgatgtccttaaaaaatcgttgaatcattataatatagattattca

caatataccattcttaatttttcattctttggagtagcaacagacggatcattacatagt

ggagatttaagaaacaaaaaaatctatgcccctggagaagtacaacagccaggagatatc

ttacatcctgatgccaacagtagccatgacaaagcttttgtattaggccaaccaaaagaa

ttttggggttgggacgaaaaactaagagctttaggttacgagcctcatccagaaggagcc

tataaaggatgggtaaaaacagctacaggagaacaaggaacttggcctctagaagaatac

ctagaagctagtatgattaaaatagcgcatgaaaatggtgtaaaagtaatggcatctatc

ggtggatggagtatgtgtaaacatttcccagagatggctgcagatgttaccaaaagagca

cgatttgttgcagactgcaaaaaaataatcgatgaatacggatttgatggaattgattta

gactgggaatatccaggaccatttgatggaatgaactttactggatctgtagcagattac

aaaaactttacaacccttgtaaaggaaataagagccgccatcggaccagataaactgata

actgctgctatgtctgcctctcctgctaaattaaacggcctagaatgggctgagctagac

aaatacatggattattacaatatgatgacctatgattttaatggtggatggtctaacaaa

gctggacataactctccattatatgactacccagaagcagaatatcaagatttttcatta

gatgctacttacaaaaaattagccgcattaaatgtaaacctatcaaaggtaaatctagga

gttgctttttacggaagagcagttactacagaaggtagcccagtgttaggaggaaaaaca

aaaaaaactcctcaggtatttgcagtagatggccctgtagattcggcctctgaccttgtt

aattttaaagacatggaaggaaccccttactacacaacaatcttaaaagaattagcttcg

ggtaactggacagaaaactgggatgatacggcaaaagtgccttacctaactcataaaaca

tctaactctttcttaagttatgataatgaacgctctttatctttaaaagcagaatatatc

aaaaacaaaaaactagcaggatgtatcgtttgggaagtatttagtgattttgtagttggc

cctgaaaccaaaaaaataggaaagtatccatactgccctgcaacaaaagctcctttggct

aatgtaatcaataaggcttttgcaactggagttattgataccaataaagctcctgttgta

agtatcgtatctcctactgcccagcaaaacattgagcaagatgctccaaaagagattact

cttattgcagaagctagtgatgttgatggaacagtaacatcagtaacttttgaaatcgga

acgcaaacactagcaagtacattagtaaacaaccaatatactgctacatggacacctgta

ggctttggtgactattcattgactgttactgctactgatgacaaacaggcaactaccaaa

aaaacagcagctttttctgtttctaaaacagtgggtacaaccaatcaagctcctgtagta

aaatttatagctcctacagcacagcaagatatcaaacaagctacaatcaatgcaattact

cttattgcagaagctagtgatgttgatggaactattgcttctgtaacctttaaaatagga

acgcaaacactaacagcagctttagtcaacaatcaatacactgcctcttggacaccaaca

gcatttggtgcgcaaacagcaacggtaattgctacagatgacaaaaatgctactgcagag

caaacagtaaactttactgtttctaaaactacaaccactccagaaggagaacacccacta

gtatcaagagctcaatgggatgttttattcccacacagattcgggtcaaaacttgtagga

gatatctatgtaatcgatcctaaagatgatttctatagctatgatgcatttatagaagct

attaaaagaatgaagaatatcgaagtaatgtttgaaagaagatgtggtaccaatgcatac

agaataactcgtacagacaaaactactggagcctctttattactgagaaccgatgttgac

tttgacgcaccaagaaatttagaaaaagagatcattatagataatgttgattatgcttca

ttcttagaagaaggaactttagaaactagaaaaagagagctttctgccttttttgcaaat

atctctcacgaaactacaggaggatgggatacagcaccaggaggaagattctcttgggga

ttgcattttaatgaagaaaaaacagatgctccttatgtagccgcaaacgatcctcactac

ccagcaacaccaggtcaatcatacaaaggaagaggacctatccaactttcttacaatttt

aactatggagctgccagtcatatcatctttggagataaacagatcctattagataatcca

ggtaaagtacttgaagactctgctttagcatttgaaacagctatctggttctggatgaca

cctcaatacccaaaaccatctgcacattcagtaatggcagacacttgggttcctaatgaa

ttagatatcgaaaaaaacagagttcctggactaggaatgactgtaaacataatcaatgga

ggtatcgaatgtggacacggaggtgcttcagaaaaaggacaagtagcagacagaattgga

tattacgaacgttttaccagcatctataatatcggaacagatatggacggcgtaaacgat

ttatcagactgtggttgtaaagatatgactaaatatgcgggtaattctggaggtgacttt

accgctgagccatgtgcacagaaacctgcaattacctttatatctccaacaaacaatcaa

ttaattctacaaaacacctttgctcctgttgctgtaaatatagaagtagatcagaaaaac

tctgttctaaaaacacttacaacaactattggtactcaaacattcaatggtgctactttt

tcatggacacctaatggatacggaagtcatatcatgacttctagtgcaacttttgaaaat

ggaaaaactgccacagctaccaacaaagtaatcatttgggatggaactaatcttgattgt

aatgaaatcccagaatggattaatgttagaatctataaagacaaggataattatgtaaga

tacaataatgcaatctacagaaacaagtggtatgcaccaaacggtagtactccaggagtt

gatggggtttgggaactagtaaaagaatgtactggatcacctagctctgctccttcaata

actgaagtttcacctgctaattcaagtgttatagaacagcctcaactagctagtattgca

ttaaaagcattagtaaacgataatgtatccgtaagtagtgtttcctttatggtaaacaat

actgcagttactgctactcaaagttcttctggtgaatatacagccaactggactcctact

gcttttgggcaagttacttttaaaataactgctaccgatggtgaaggtttatcttcagaa

agtaacactacttttaccgttaagaaaaaaactactggaggaacaacctgtggtgatgtt

actgcatgggaagctaaagtatactcagcttctggtcaatttgtaagttatgaaggaaaa

gtatacaaaaataaatggtatgctgagatcactgatattcctggggtaaatgctgtatgg

agctatgtaagaaactgtaatgaaacaccttcttatacagattattgtggatcacaacca

tggattactacccaagcttatagtaaagatgataaagtatattattcaggaaaaatatac

caagcaaaacattggacaaaagatagcgcaccagacacaagtaacgaatggaattttgtg

tcagattgtgtttctacaggttctattacagctaatctataccctactgttgtctctgat

caggttaatatcagtgtaaattctaaaaagaatagcgctttaaaaatagtactttatgac

tattctggaaaaacagtacgaacccttgtggatcaaaacatttctaaaggagattctact

tttacagaagatctatcttctttaagaaatggtatatacatctataaaatttacatagat

ggtaatgtcaaaacagaacggttaatcaaaaaataa

>fig|290174.3.peg.1127 Chitinase (EC 3.2.1.14) [Aquimarina sp 135]

atgaatccatcaaccaactcacaaccttacggtgcgtgggaatctttaggggcttgtagt

ggcgggaacaacaatattgctcctacaataaatattacatctcctacaaataattcatcc

tttgaagaaggtactacaattaccattacagcaaatgccagtgatcaagatggcagtatt

gcaaaagttgaattctttaacaacaacactttacttggagaagatattactactccttat

acttttagcatcaacactcccatagtggggagttacaatcttactgccgtagcaacagat

aaccaaggagcatcaactacatctatatcagtttctgttatggttacacctgcgggtact

ggaggaaatacttgtaatggtttacctcaatatgtagaaggaaatccttatgaccaagga

gaaagtgtacaaaatgaaaatatgatttacgaatgtaaaatagcaggatggtgctcatct

agctctggttgggcctatgccccaggaacaggagctcactgggaagatgcatgggttaaa

actggaaattgtgggggttctggaggaaacacacctcctaatgtaaatattactgctccc

aataatggaactatttatgctttaggtgacattataacaattactgcaaatgctacagat

aacgacggcactataacaaaagtggagttttttgatggcacaaccaaactaggagaagac

acaactcttccctattcatttaccctaaccactagtgcaaatggttattcacttactgct

attgctactgatgacaaaggcgcatcaactacatcacctatcgtatctataagaagagat

acaggaggaggtaatggcgccttaccagcacgaatactgaatggatattggcataatttt

cagaatggttctggtatcagtaagcttagagatataaatcctaattgggatgtcattaat

gtttcttttgctgtaccaactgtttcgtctactgatggacaaattggttttcagttagat

cctgtattcaacaccataaattatagtgtagctgattttaaatctgatattttattactg

caaagccaaggcaaaaaagttattatttctattggtggggcagaaggaactgtacgactc

aactctatatctgctagagataagtttacaagttcaatgatttctattatcgaggagtat

ggttttgatggaatggacattgattttgaggggcaatcgttatctcttgattttggtgat

aatgattttgctaacccaacaactcctgttatcgtaaataccatcaatgctgtaaaaagt

atttgtactcattttggaaacaattttatcctaacaatggctccagagacattttttgta

caattaggatatgctttttatggtggaatatcacaaggtgcagacaggcgagctggtgct

tatttaccattaatacacgcactacgtgataaattgacttttttacaggtacagtattac

aattctggatctataactgctttggacgatcaggcatatgctatgggaaattcagatttt

tatgtttctctagtagatatgctactcaaaggatttcctattacaaaaaatcaaagttat

tttttcccccctctacgacaggatcaaatattaataggagtccccgcttttgtacaagct

ggtggcgggtatacaggacctgatggtgttataaaagcaatggattatcttataaaagga

acttcttttggaggacagtatcaattaaaccaaacatactccaacctacgaggagttatg

agttggtctataaactgggatgaatatgataatttctctttttctaaccctgtaagaata

tatctagatggacttcaaaacaggtctcctcttggcttacaaagaaataatgaatcatca

aacaatataaatctatacccaaatccaataacggataatcatcttgcagtgtcaattgat

actccaatcaatgattcttcttttactttacaaatttttacccctacaggagtaaaagtg

tttgaacaaactaatgtttcactaaaaaaaggtagtaaaaccacccttacttttgatatg

agtaatttgaaatctggtttatatctatataacctcttattggataacaaaaagactagt

ggaactctttttatagaataa

>fig|290174.3.peg.2789 Chitinase (EC 3.2.1.14) [Aquimarina sp 135]

atgaagttaaaactactcttaattccattggttgtcttttatcttatagggattcaaaat

gggtttgcacaatctgtatttattaatgagatacactacgataactcaagtacagatgta

gaagaagcaattgaaattgcgggaacagctggaacagatcttactggttggagtcttgtt

ttgtataacggatcaaataattctgtatataatacaatttctatttcgggagtgattaca

gatcaacaaaatggttttggaaccatggtaaaaatatttcctaccaatggattgcaaaat

ggctctcctgatgggattgcattggttgatgctactaatacagttgttcaatttttgagt

tatgaaggggtaattactgcaatagatggtccagccttgggattgacaagtacagatatt

ggcgtgtcagaatccagtaatacggctgtagggagttctctgcaattatcaggtactgga

accagtgctgccgcatttgtttgggagactactactgcaaattcgtatggaactgtaaac

acaaatcaaacgtttgaagcttctttggccatgcttgtaattaatgaaattgatgcagat

actcaaggttctgatacattagaatttgtagaattgtatgatggtggtgtagggaataca

ccactagatagttatgtattggtaaattataatggaagtaataatgccagttacaatgct

attaatctagatgggttttctacaaatgcacaaggatattttgtaattggtaatgctggt

gtaaccaatgttgatttggtggttccaggtaatacatttcaaaatggagcagatgcagta

gttttgtattttggagaggcgactaattttcctaatgggactcctgttattacagagaat

ataattgatgcaattgtatatgataccaatgatgctgacgatatggagttattggtatta

ctgaaccctaatcagccacaggtaaatgaaggggtaaatggtaataaggataaagaatca

ttacaacgtattcctaatggagaaggaggaatgcgtaatacttcttcttatgaagcaaag

atgccaactcctggagccagtaatcaaggtgtaattgtgattcctggtgaccctattagt

attagagaagctagggcaacagtagtaggtacagcagtaacaattacaggagtgcttaca

gtcacagatagttttagagggccagcatttatacaagatgagacaggaggtatcgctatt

tttgatgatcagattcaagccaatgcagctttgaaagttggagactctgttaccataaca

ggaattagagccacttttaatgaccaaatacagattagtacagtaacagaagttgtgcat

aatggtctgccacaaaatccaataaccccaatagatattactttggcagcacttgctaat

caccctggcgaattagttagagtgttacaacctgcttttcctaatccaggagatattcta

tttgggaattctaattttacacttactgatacaagcgggagtggacaattgcgtattgat

aatgatgtggcttctgttgtaggaaaagaacaacctgttacctgttcagaaattactgga

gtagtaggaaggtatagagatttttttcaattactacctagacaatcctcagatataccc

tgtgcagtagagtttattcctccaggagatacagtgggttaccccaaagaagatactttt

gatgttgttgcttggaatatagaatggtttggcgatgaaaacaattcgccagtagggcaa

aaccctctttctgatgctattcagagggatagtacagcgactgtactcaaaaaattgaat

gcagatgtatacgcagtagaggaaatcgcagatgatactttgtttgcagaattagtgagt

ttattaccagggtatgattatattctttctgatgctgtttctcgcccaggttctggtgga

gtatcacaaaagctaggatttatttataatacaaaaacagtttcagtaatagaaacaaga

gcgatgtttacatctatccatcctctatataacggaggagatgcatcggcccttgttgat

tatcctagtgaaccatcacgtttttatgccagcggaagagtacctttcttaatgacagca

gatattactatcaatggagtaacagaacgcatggatctaattgcattgcatgccagagca

aatagaggctctggtgctcagaatcgatatgatatgcgtaggtatgatgtagaggtatta

aaagactctttagatactaattttgcaaataataaggtgattcttttaggggattataat

gatgatgttgatcagactgttgcagatattccttctactatttcaagttttcaggcatac

gtagacgatactgtgaattatagtattgtatcttctgcattaagcgatgcaggtttgagg

tcttttgtttttagagaaaatatgatcgatcatattgcaataaccaacgagcttgatagt

gcttatatagcaaactctgtgacggttcattatgaggtatatgataatgattatgcattc

accacctcagatcatttaccagtttctgcacggtttttacttacaccacaatttgtagaa

aatcaatgtatggctgcatcaatagtagcttttgatcaaggaagaagaagagatggtcgt

gagatatcatggcttagagggaaagctaaaaatagtataggtgctccgcatgaaagaagg

tattttaattttgtaagtcttggatttggtgggtcgattactttagaattggataatgaa

atttttgataatccagataccaatgaatttattgtatttgaatctacaggctttttagac

aatgtttcatgtgaatactatccagaatcagcagaagtatttgcatctcaggatgggata

gaatttgtttccttgggaactacttgtcaagatggagaatttgatcttgctactggtaat

ttacgttctgcaaaatatatcaaggtggtagatacaagtgacaaagactcttttccttgg

tttgcagatgggtacgatcttgatgcaattatgtgtattgagtctgatgaacgtatttct

aacaacaagagtgctacaaatatttctaccaaaaagcctaccgctctagaaagtgaatta

cttagtagtgattttggtttagaagaagcccatatcttactttctcctaaccctgttgaa

gaaaaattatctgtagaagttaatacttttacacaaggtgatgtagctatttccattaca

gatataacaggtaaaacaatttatgttcaaacgatgaagcttactgttggaaaatctatt

ttgtctattgatatgaatctgtacccaaaagggttttatgtagttcatatgtctaatact

agtggaactttaaacgttactaaaaaggtaatgaaaaattaa

>fig|290174.3.peg.3725 Chitinase (EC 3.2.1.14) [Aquimarina sp 135]

ttgcttgtacatgtatttttattttcgtgtacaaatgaaaacttagttgaggacgaagtt

ctggaacctcaagattctgtatcaaattcggtcggcaaaaccgctttaaccaaacctatt

gtactaggatattttccttcctggtcagagagttgggcagaaccaggaaaggggtcgaca

cttagagatatccctacccatgtaactcatgtttttttagcatttgctagacctaatctt

agatatcaaaaaggatctctggatataacgggtacaggaatacaaactccttacggagga

gaaactctaagggaatctgttgctgcattgaagtcaaaaggaatcaaagtaattctatct

attggtggagaaacctactggggttctaatgcggcttatgacattaattaccaacaaatc

aaagatttggtagatgacatgggatttgaaggtattgactgggattttgagcctaatggt

agctttgctacaattggtaaccctgttaatgtacaacggtttatcgaattctttacaaaa

tcaagagcaatcatgccaaaagggcaatatctaattgcttgtgcccctgctggagttgga

gccttaggcggtcaaaacaatgatgaccctgcttctccttatgcatacagtaaaagaaat

gctgttacaggagaatctgatgctaatttgtataatgcgactactcctaatcaagcaatt

agtttatatggatttgctactacaggacatatgatccctgtaatggaagctgttggtgat

aaaatagatcttattgcttatcaaggatataatacaggggctgctagtaacagaaaaatt

atgtacgatgcatacaaatactatgcagatagatatggtttttctattgctgccggggta

cattatccgaatgaaccatgggggccatattatgaatatacctaccaaactgtagctgat

ttatcagcccacattgctgtaaataatactgctaatcatggtgtcatgatatggcaatta

ttattgggtaacacctcatcttctgcttatggatatttatacatatctagtcagatatta

agtggtgtatcgcaatctcaagctattgctaatgcagaaaattacccgctttctccttat

ccaggcgatggtaatcaacttccttctgtatctataacgtctccaacaaataatcagagt

gtaactcagggacaaactatcaatattacagcaaatgcttctgataccgatggatctata

agtaaggttgaattctttcaaggaaatacaaaactgggagaagatacctctagcccttat

agttattcttgggctaatgctcctgtgggaagttacaacctaaccgctgtcgcaacagat

aatgctactgctacagctacatctgctgttgtatcagtaacggtgactactagtggtggt

aataaccaagcacctatagtatcaattacatctcctacaaataatcaaagcgtaacccaa

ggacaaacaatcagtattacagcaagtgcatctgattttgacggttctgtaagcaaggtt

gaattctatcaaggtaatacaaaacttggtgaggatacaagtagcccttatagttattct

tgggctaatgctcctgtggggagttataatctaaccgcagttgctaccgataatgaaaat

gcaacgactacttctactggagtatcaattactattactccttctggtggtggaggttct

tgtgctggtgtagcaaattgggctccatatccatctatctataaccaaggagatcgtgca

gtataccaaggtactctatacgaagcacaaactggtcctttatggattaccccaggtagt

ggtgagcattggtggaaaaccataggaacttgtaattaa

>fig|290174.3.peg.3736 Chitinase (EC 3.2.1.14) [Aquimarina sp 135]

atgacacacaaactcattactccaaaaaactatatggttttacgggaaaaatcatggatg

atgctttttgccgtattatttttgacaattaatttaacattttctaaagacatatatcca

gagaaatctaatcaagattatttctctataatggactactcttctacactgccaatccct

gttcaggttaacgaactgatagataaaaatatgtgggatactttgtttccttatcgtttt

ggggccaaggatacagggggtggtgtatgggtgctagaccccaaagacgatttttataca

tttgattcttttgtcgaagctataaatagaatgagtaagataaaagtcatttttgaacga

cgatgtggtacaaatgcatatagaattacacgcatagataaaacaacaaacatatcaaaa

ttactaagaacagatgtagattttgatgcaccaagaaatatagataaagagattattaaa

gaagaaattgattatgcatcttttttagaggaaggaagtttagagactagaaaaagagaa

attactgccttttttgcaaatatttctcatgaaacaactggagggtggtctacagcacca

ggaggacaattctcatgggggcttcattttagagaagaacctacaaatgcatcgtatgct

tatcctgatgttaattacccaccaacacctggtaaatcatataaaggtagaggaccaata

cagttatcatataactataattatggacctgcaagtgaatttatattcggagataagcaa

gtattactagataatccagatttggtgattcaagatgcagcattagcttttcaaacagcg

atttggttttggatgacacctcagtaccctaaaccatctgctcatgatgtaatggtaagt

agatgggtacctaatgaattagatattaccaaaaatagagttccaggattaggaatgaca

gtaaatattataaatggtggagtagaatgtggacaaggaacagagaaacctcaagtatta

gatagaatagggtattatgaaaggtttgctggaatttatggaataggaacagatatggat

ggagtacatgatctttctgattgtggatgtaaagatatgtcaaaatacggtggagatgca

gctgatttaactgctgagccatgtgcccaaaaaccgcaagtaacgttttcttctccaaag

aataatcagatgctagagcaggttacctttgcttcagtaccagtaagtttggtaatagat

gaaaaaaatacgagattaactagtgttaccactacagttggaagtcaatcgtttactggt

gttacgtttaattggacaccatctagctatgcaagtcatattcttacagccaatgctgtt

tttgagaatggtgcaacggcaacgtctagtatcaaagttattgtatgggatggggtaaac

cttaattgtcaagaaattcctgagtggaattctacaagaatttataaagacaaggataac

tatgttagatataacaataaaatatatagaaataagtggtatgcaggtagcggtagtgtt

ccaggaactgcttctgtatgggagtttataaaagattgtgctacatcaggaggaactgca

ccagtaattacatgggagtctcctgcaaacggacagattatagaacaggcaactttatcc

gctgttgcattgagagcaagtgctacagatgatggaacaatacaatcatttacctttaga

cataatacaacagaccttagtactactgcttctggaaatagctatacagcaaattatact

ccaacagcttttggagaagttactataattgcatctgctacagataaccaaaataaaaca

acagagaaaacaattacatttactgtaaaagaaaaagtggcaggaggaaataacaatgca

ccatcggtgaatattacttctccagctaataatgcatcttttactgctggagctgctatt

gatgtaacagccaatgcttctgatacagatggtacaataaccaaagtagaattctttgat

gggaacagtaagataggagagcgtaccacaagtccatacaattatactattactaatgca

gtagtaggtacctatgtgttaacagcaaaagcaacagataacaaaggagcaaccgctact

tcttcagttgttaatgttgtagtttctggcggaggaaatggaaactgtactggcgtacaa

caatatatagctggtacttcttatggattagatgatgaagttgttaatgctggagaaaaa

ttctcatgtgatataccaggatggtgttcatctgctgcagcatgggcatatgctccagga

acaggtgcacattgggaacaagcatggacaaaagtaggagcatgtgctacaactcaagca

agatctaatgattatttcttgtaccctacagttacagaagatatagtaacactaagagtt

aagacaaagaaggtttctcaagtaagagttgatttatatcatatctctggaaagcttgta

gagacacactcttttaaatcaaatcaaacaaaaattacaaattcatttatacaagatctt

tcaaatctcaaaaatggactttatattttcaaaatttatgttgatggcaacatctatttt

gagaaagttctaaaaaagtaa

>fig|290174.3.peg.3737 Chitinase (EC 3.2.1.14) [Aquimarina sp 135]

atgaaaagtttttataccttttttagtctgtttttttgtgtcctgtttgtacaggcgcaa

tataacttcccaacatgcgcacccgcatggagcgcagccaatgttccttatacgcaagga

caggaagcctctgtagatggcaaaaattatagatgcaaatattacaccaacgaccagcca

ggggctgcttcatgggaactagtaagtccttgtggagatggaggtcttggaccaggatat

gcaggtaaacagaaaattattggatatctacctacatggattgccaaatatgatattaag

aacaactttaatccagaagtagttacacatttgaatattgcatttttattatttaaacaa

aacaatcaaaattacaatagtaatgattttgcttcagttgcttttgatcagtttcatgta

agaaaagtagattctgtattaaccgatttaggagtacttcaaaaagctagagcaaaaaat

gtaaaagtttctgtcgctcttggaggagctactgattttgcatttttatggttaatgaat

aagtatcataataatgatgctaaactagagcaaatagctactttgattgcagactatgtt

tctgtaaatcaattggatggtgttgacttggatatggagtgctggtgggcagatccaaat

attgcaggaacttctgatcaaggaggacgtgtacgaggaagcaaatggggagatgccgat

caaggaccacaccctgcaggaattggtttaagaaaattaagccagaaattaagagttaaa

atgccaaataaactgatcacagcagcagtattcggaacttcgtggtatggtaataactat

gatgatggaattgcagattatatggattgggtaggtcttatgtcatacgattttacaggt

tcatgggatgcatctccagaaggaccacattcttctctttataaagtgccaacaggaact

tatcaaggacaaacagcagataaccctatttacagcgcagaagatgcgttagaatattgg

atgggatttgctcctcctgcttggaatcatgctggagggtttaatgtgccaaaagcaaaa

ctagcttttggagtgcctatgtatggatatgatttttctgaaaagaaacctgatggagga

aatggagcaaaatttgttccttataacgaaatattagaagagtttcctaatgcggcaaca

agctacgatcctaaagatactaagaagttaagtggatttataggagaaaaaaataagaag

atatattttgatactcctaagtctgctggagcaaaaatgaaatattctaaagactatgga

caccaagggttaatcatttgggaacttactcaagatgccaattataactcttcttcaagt

atattaaaagcgatcaatgaggcagcaggaaataataatacaatcaataatccaccaaca

gttacttggcaatctccaacaaataaccagataatagaggttgcagagctttcacctgtt

acattacaagcaagtgcaactgactctgacggaactgttcaatcttttacttttaagcat

aataatacccctattagtgctacggcaaatgggagtaattatacagctacttttacacca

acagcatttggagaagttactttggtggcttctgcgacagataacaaaaatgcaacctct

gagaagactattgtttttactgtaaagaaaaaaggaacaggaggaaacaatgtagcacct

actgtaaatataaccgctcctgtagataacgcatcttttaatgtaggagatcctatttct

attacggcaaacgcaacagatagtgatggtacagttaccaaagtagaattttataatggt

acagtaatgttaggcgaggatacgacagctccatatgagtatacatggcaaaatgttgcc

gctgggaattattcaattacagcaaaagcaactgataaccagacagcttctactacatct

actgctattgcaattactgttactgaagtaggaactggtagttgtgctggtattgctcaa

tatgtagcaggaacatcttacggattaaatgaagaagtaaccaatacaggtgagaagttc

tcatgtgatataccaggatggtgttcttctgcagcggcatgggcatatgctcctgggact

ggagcacattgggaaatggcttggacaaaaacaggagtttgtacaacagcactagaagct

agtaagtactctttatatcctactgtgactcaagatgtagtaaaccttagcgtacataca

gaaaatgcttctctaatcaaagttgatgtatatcatatttctggaaaattaattgacaca

cagtcttttaatggaggtaatgcaaaaacagctaagtcattagtaaaagatctttcaaac

ctcaaaaatggactttatatcttcaaaattcatgttgataataacgtttattttgagaaa

gtgcttaagaaataa

>fig|290174.3.peg.3739 Chitinase (EC 3.2.1.14) [Aquimarina sp 135]

ttgaagacattcttacttttaattttgttatgtgcatggagcattggctctgcacaagta

aacacaggaggaactgcaacgacagcaaaccaccaaaaacagataattggttatattacc

aattgggatgcttggaaaacgagtacagcaggtgttccaggcccaggtgcattaacgcac

cttaatattgattattctaagtataccattcttaattattcattttttggcgtagccaga

gatggatcattacatagtggagatcatagaaataaaaagatttaccaagatggcgtttct

caagaacctgctgatatattctttactgatatttacagtagttgggatatgcatattctt

tttggagagattgatccacttcaatatatcaatgaggatgcaaaaagaagagcagaagct

caaggttttcaagttgctgttggcggtagtacgtggacacacccagcatgggggttaagt

ggatcacttcctttaccactgcataaagaaactggagcaccagggcttattgagttagcg

catcaaaaaggagttaaggtaatggcttctattggaggatggagtatgtgtaaacatttt

cctgaaatggctgcagatcctgtaaaaagagcaaagtttgtagaagattgtaaaaaattg

atcgctactggatttgatggaattgatctagattgggaatacccaggaccttttgaagga

atgaattttcttggaactcaggcagattttgcaaatttcgaaacattattgcaagaaata

cgtacagctattgggccagataagttgatcacatcggctatgtctgcagatccaagaaaa

ttagaaggatttaactgggcaaatgttgtaaagaatatggattactttaatatgatgacc

tatgacttcaatggaggatggtctaatattgcaggacataatgcaccagtatacccatat

acaggagcagaagtacctttctttaactggcaatctacattacaaaagctaaacgagtta

ggagtaccttcaaataaaatatgttttggagctccattttatggtagaggagtaattact

acgggtaatgccgatctaaatgttgctacagtaaaaaggtcagaaaatgtacagccagat

ggaccaatagaaacagcagctgattatacaaactggccaaaagaagtatatgatggtacg

cctaattacttttttataaaacaaaaagcattatctggtactggtggatggactagaaaa

tgggatgatgaagcaaaggttccttatttagtaaacggaaaatacttcttaagctatgat

gatgaagaatctattggtatcaaagcccagtttataaaagataataatcttgcaggtact

attgtatggactgtatatggagatttagaaataagtggttctgctacttcatttggacct

aaattaaaaaggtggtctaatgtaaaatctcctttagtaaacaaaatgaatgaagtattt

gcaaatggagtagtagtaaatcctgctcctacagtaaatattacatctcctgcaaataat

gctaattttgctcctggtgcaaccattgcgatcacagcaaatgcatcagattcaaatggt

accgtaaccaaagtagaattttttaacggagcaacaaaattaggagaagatacaactgct

ccttatgagtactcatgggtaaatgttgtagcgggtaactattctattacagcaaaagca

acagataatgaaggtgctacaaaaacatcttcagcagttgctatttctgtaggtagtaca

ccaaatgaaccacctacagtaagtataacatctcctacaaataatgattcttttacagca

ggaacatctatagctatagctgcaacagcagcagatagtgatggtactgtttctaaagta

gaattttttaatggaacaacaaaactaggagaagatactactagtccttattcttatagt

atagctagcgcggtggtaggtacttataccttaacagctgtggcaacagatgataaagca

gcgactacgacttctgctgtggtaacagtgaatgttactactgatggtggtggaggtagt

tgtagtgcttttccaacatggtctgcttcagaaatatatactggaggtatggatgtaaaa

tataacaatgtacactaccaagctagatggtggacacaaggacagaatcctgaaaacagc

tctgtttggaaaaagataggaccttgtggaggaggtggatctaataatgtagctccaaca

gtgagcattacatctcctgctaataatgcatcttttgtagaaggaacttctatttctgtt

actgcagatgcttctgattctgatggtactgttgctaaagtagaattttataatggtgct

actctgttaggagaagatactactagtccttattcttatacaattgcaaatgcaatagtt

ggaaattatacacttactgcaaaggctacagataatcaaggtgctaatacaacctcttca

gcagtgtcagtttcagttacaccaactggtggtggaggtggaaattgtgatggtgtagca

caatatgtagcaggtacttcttatggacaaaatgatgaggtacaaaatgatggagcaaaa

tattcttgtgatgttccaggatggtgctcatctgcagcagcttgggcgtatgcgcctggt

actggggctcactggcaaaatgcatggacaaaaactggtgactgtactggtggcggagga

tcagggacttctcctgtggtaaatattacctctcctcttaacggcgctacatatcctgta

ggtagttctgtaattgtttcagcagatgcaacagatgatggtagtgtaactaaggttgag

ttctttaatggagctactaaattaggagaagatactacaagtccatattcttatacaata

gcaaatgcgcaaacaggaagctattctttgacagcagtagctactgataatgagaataat

caaacaacatctgatgcgattactattagaagtcaagctccaggaggaggaaataataac

cttcctggtaagattcttgtaggatactggcataattttgataattcatcaactactcca

agattgagtgaagtgtctagagagtgggatgtagtttgtgttgcttttgcagaaccagta

agaggtagtgcttctgatatgcaatttagcccatatagcatctatgcaggaaatactcaa

gcatttataaatgatgtagctactttacagagtagaggacagaaagtattgatttctata

ggaggagcaaatgctagagttgagcttactaatgcagcagagaaaaacgagtttattagt

tctatgactaatatcattaatacttatgggtttgatggactggatattgatttagaagga

agttctctttctttaaacccaggtgatactgattttagaagtccaacgaccccaaagatc

atcaatcttatagatgctacaaaagcgatcagatctagtataggagcaaaccgtttcatt

ttgagtatggctccagagacagcttatgtacaaggtgcttatggaaattactctgggata

ttcggagcatatttacctgttgtgcatgcattgcgtaatgagatgaattatatacatgta

cagcactataatactggatctatgtttggtagagacggacaggtatatcaaccagcaacg

gctgattttcatgtagcaatggcagaaatgttgattacaggattccctattgctcaaaca

ggacttacattcccagggcttagagctgatcaagtagcaattggtttaccatctactaca

caggcagcaggaagtggttatacttctgaagcagtagtgcaacaagcattagattatttg

atcaaaggggtgtcttatcctggtagatcatacactactagtgcaacatatcctactttt

agaggattgatgacatggtcaatcaattgggatttagtaaataattctgcattctctaca

agtcatagtgcttacttaaaaggagttgctagtagaaatgctacagctagtcagaatgta

ggtaaagtattccctaaccctctgtctggtaatgtaattaatgtagcattagataatagt

gctatttctaagtctggaggagctaattatttcagattccaagtatttaatactagtgga

atagaagtttttaatttccaaaatgataacctaagatcagggcaagagactatgagtttt

gatataggtgatttagaagcaggaatgtatttttatacaatttcagtagctaaaaataaa

acgactggtaaaataattagaaaataa

>fig|290174.3.peg.4488 Chitinase (EC 3.2.1.14) [Aquimarina sp 135]

atgaccctttttattacctcatgtagtagtgatgatactgtgaatgataaggatcctatt

gatactgttggaggagatggtaatggtggaggtgatacagatacaggtggtgatactgga

ggagatggtaatcaaacaaaaaagcctattattttagggtattttccttcttggtcagaa

aattgggtttcttcaccagatcaaggatcaaaactaagagatattccatcgcatgttacc

catgtgtttttggcatttgcaaaaccgaatttagtgtatatcaagggatcattagatatt

tctaagacaggaatccaaactccgtatgatggtcaaactctcaaagaatctgtagcggta

ttaaaaaagaaaggaaccaaagtgatattatcggtaggcggagaaacttattggggtaca

gaagaagcttataatatcaattaccaacaaataaaagatctagtagatgatatgggattt

gaaggaatcgattgggattttgaacccaatggaagttttcaaaccattggtgatgccata

aatgtagaacgttttattgcattctttaatgaatccagagctattatgcctaaaggagaa

tatatattagcatgtgctcctgctggagtaggggctttggggggagaaaaaaatgacgat

ataagttcaccatatagctactccaaaagaaatacagtcacaggagaatcagatacaaat

ctgtacaaagctacagaacctaatgaaggaattagtttatacggtttttctacaacaggg

catatgatccctgtgctagaagccgtgggtgataaaatagacttaatagcctatcaaggg

tataatacaggagcagcgtcaaacagaaagatcatgtatgatgcatataagtattacgga

gatcgttatggatttgatattgctgcgggaatacattttcctgatgaaccttgggggcct

tattatacctatacctatgagaatgtagcaacactttttgcgcatatagcatcaaaagat

aattcagatggagtaatgatatggcagttacttttggaaaaagaaaacaaatctgcatac

ggatatctaaatgtagcgagtaaagtacttaacggatcttctacaacggaagctataaat

acagctgaaaatttttaa

>LN554846.1:1955753-1958290 Aliivibrio wodanis genome assembly AWOD1, chromosome : 1

ATGTTTAAAAAGAAACTGGGGTTTTGCTCCGCAGCAATCACTCTTGCCCTTTCTGCTCCAACTTATGCAG

CTATACCTGGTCAAGCGATTATTTCTTGGATGGAAACTGATTTTGCTATCATTGAGGTTGATCAAGCTGC

GACATCTTATAAAAGTCTAGTTACAGTAAAAGAGTTCGCAGAAGTTCCTGTCACTTGGGATCGTTGGTCA

GGCGAGCCAGCTGAAAAATGGCGCGTTTTATTAAATGATGTTGTAGTTCATGAAGAAACAATATCTGCTA

CAGCATCTCAGAAAGCCTCTACCATCTTAGAAGTTCGCCAAGGCGGTCAATATGAAATGACTGTACAATT

ATGTAATGGCTCAGGAACTTCTGAAGAGTGTTCAATAAGTGCAGCAAAATCTATTGTTGTAGCTGATACC

GACGGCAGTCACTTAGATCCACTACCAATGAATGTTGATCCAGCAAATGGCAATTACACAACTCCTGTCG

GAATGGTTTCTGGTGCTTATTTTGTTGAATGGGGTGTATATGGTCGTAAATTTGCCGTTGATCAGATCCC

TGCACAAAATTTAACTCACATTCTTTATGGCTTCGTTCCTATTTGTGGTCCAAACCCTTCTTTAGGCGAA

ATTGAAAATGGTAACAGTTTAGCTGCTCTTAATCGCGCTTGTGCTGGAACTCCAGATTATGAAGTGGTTA

TCCATGACCCGTGGGCAGCAATACAAATGCCACAACCTCAATCTGGTCATGCTCACAGTACTCCTTATAA

AGGCACTTATGGCCAAATGATGGCACTCAAACAACGCTATCCTGATCTAAAGATTGTTCCATCAATTGGC

GGCTGGACGTTATCTGACCCTTTCTATGACTTCGTTGATAAACCAAAACGAGACATTTTCGTTGCTTCTG

TTAAGAAATTCCTTAAAACGTGGAAGTTTTATGATGGTGTAGATATTGATTGGGAGTTCCCTGGTGGTGA

CGGCGCAAGTGCAACAGGTGGTGATCCTGTTAATGATGGCCCTGCTTATGTTGCTTTGATGCAAGAACTA

CGAGCGATGCTTGATGAATTAAGCGCTGAAACGGGAAAAACCTATGAGTTAACATCAGCAATAGGTGCTG

GTTATGACAAAATTGAAGATGTTGACTATGCAGCTGCGTCACAATACATGGATTATATTTTTGCTATGAC

ATACGATTTCTTCGGCGGTTGGAATAACGTCGTTGGCCATCAAACTGCATTAAATTGTGGTTCACACATG

TCTCAAGGTGAGTGTGATGGTACTGGTCTTGATGATAAAGGCGAACCTCGTAAAGGCCCTGCTTATACAA

TCACTAATGCGATTGACTTATTACTTGCTCAAGGTGTTGATGCTAAAAAACTCGTGGTTGGCGCTGCTAT

GTATGCGCGTGGTTGGACTGGTGTTACTCGTGAAAGTATGACTGACCCAACCAACCCAATGACAGGTGTT

GGTAATGGTAAAGTTGCAGGTTCGTGGGAAGCTGGTGTTATCGATTATAAAGATGTAATAACTCGCTATG

AGAATAAAGCAGGCGTTGAAGTGGGCTATGATGAAATAGCTCAAGCAGCTTATGCTTATGATCCAAGCAA

TGGTGACCTTGTTACTTACGATAATAAAAAATCAGTATTAGCTAAAGGTGAATACGTTCGTTCATTGGGC

TTAGGTGGTTTATTTGCATGGGAAATCGATGCAGATAATGGTGATATTCTTAATGCTATGCAAGAAGGTC

TTGCTGGCGACGGTACAGTTACACCTCCTGCAAACAAAAAACCTGTTGCTAACGCTGGCGTAGATGTTTC

AGTTATTGCGCCTACACCTGTACAACTTGACGCTTCATTATCATCCGACAGTGATGGCTCTATTGTTTCT

TATGCATGGACACAAACTTCTGGACCGAGTGTTACATTAATTGGTGCAGATTCAGTCAACTCAAGCTTTG

CAACAGATACCTTTACTCAATCTGAAACGCTTCAATTCACACTAACCGTTACTGATAATAAAGGTGCTAC

AGCATCTGATTCAGTGGCTGTATCAGTCACAGTTGAAGGTACAGAACCTGTAAATACACCACCAGTAGCT

GTCATTGTTGCTCCTACATCAGTAAATCAAGGAGATGTTGTTACACTGGATGCATCGACATCTACTGATG

CTGACAATGACTCATTAACATTCTCATGGCTAATACCTACAGGTGTTGATGCTACTGTTATTGGCTCTCA

AGTAACCTTTACAGCAGGATCTTATACTGTTGATACACCTTTCTCCTTCTCTGTAACTGCAAATGATGGT

CAAGCATCAGATACTGCAAGTGTAACAGTTACTGTACTTAAAGATGCTGGAGAACCACCAGTAACTTGTG

ACAATACATGGGATGCTTCAACAGTTTACACTGGTGGTGACCAAGTTTCACAAGCAGGTAAAGTATGGGA

AGCAAAATGGTGGACAAAAGGAGATACACCATCGAAATCTGGTGAATGGGGTGTATGGAAAGAAGTAGGT

ATTTCTACTTGTAATTAA

>CP016228.1:2124736-2126925 Vibrio crassostreae 9CS106 chromosome 1, complete sequence

CTACAGCTTTGTCCAAGCATCTTGCCAGTATTGGCTATCTGCTGGAGCGTAAGACGCGCTTGCACACCAT

GCCGTGTATGGCCAAGGCTTACATTCGTACAAGCCATTATCGCTGCCGACCACAATGTCACCCGCTGCGT

AGTTTGTGCCCGCTTCATATGGAGGGTAATCACCTGGAGGAGGTGTTGTTCCGCCACCGTCTTTAACAGC

GAACGAATCGTTTACCATCACCATTTCACCGTCAGATGCTTTACCGACTACTTCAAGTGCATATTGCCCT

TCCGCTACATCGTATAAGTCGATGGTAATAGCACGGCTGTCCGTTACGTTAACCTTAGTTTGCTCAACCA

CAGTACCTGCACTGTTGATCACCATAGCGGTAACATCTAATGCTTCATTTGATGTCAGTGTCAGATCTAA

ACGCACGTTGCCGTTATCTGGCGCATATTGTGATTGAAGTCCTGAAACCGAAAGCTCTAAGTCATCGCCC

GGATCTGGGTTACCACCACATGAAGAGTCTGAAACTTTTCTCCAAACGCCCCACTCACCTGTTGTTCCTG

GCTCTTCGCCGCGAGTGTACCAACCTGCTCCCCACGTTGAACCGTCATGAGATACTTGGTCACCTTCAAC

ATAAACGGTGTCGGCATCCCATGCAGCAGCACAGTTAGAACCATCAGTTACAGACACTTTACGGTCAACG

GTTACGGTTTGGTTCGCGCTGTCTGTCACGCTATACACAAGTGTGTAGTTGCCGATTTCATTTACATCGA

CACTGCCAGTGTGTGTAATAGTGCTTGTCAGGTCGCCATCTTCAGCATCATTCGCCGTCACGCCTGCCAT

AGCATCAAATGCAGTACCCAACACAACTGTTGTGTCAGATACGCCATTAAATACTGGCTTCTGGCTGTAA

ACCTCTACCGTTCTCGCTTTAGTGGTTTCGTTGTTATCGCTGTCTTTCACTCGGTAAGTCAAAACGTAAG

TGCCAATAACACTGGTTTCAACGTAACCTTCAACATCGATTGATGACGTTAGATCGCCGTCTTCTTTGTC

TGTTGCCGTCACGCCATCCATAGGATCAAACGTTGTGCCGTGAAGAACACGCGTATTTTCGACACCTTTA

AGAACGGGCTCGCCCTCGACTGGTGGTGGCGTTACCTGACCGTGTACAAACGGGCCGTAGTCTTTAATAA

ACTGCTCGTTGTATTGTTGGCCGCTTTTGTTGGTGCCCATGTCCCAGTTGATAGACCAAGTCATCACACC

ACGTAGCGGTTGACCTTGCGCCGTTAGGCTATCAAATGCGTCGTATAAATCTTGTGGCTCTTGTACAAAA

CCGGTTGCTGCAGCATCGATGCTTGAAGGAATACCAAACACGAGCTTGTCATGTGGGATCTTGTGGAAAC

CACGCGTTCCGTTGATAAGAGAATCAGAAATGTAGTAGATGAACTCTTCTTTTAACGCATCGTTGTTTTG

AGCAATCCAACCCACGCCTTCAACCCAGATACCATCGCCACCTTGGTTGTAGAATTGTGGGTTAATCCAG

TCGTAATAGCCTTCTAGATTATCGATATAAGGAACGTACTTACCGCCTGTCGTGAGATACGGGAACTCAG

GCGCCATAGTAATAAGGAAGTTTTTACCTTCAGCACGGTAGTGGTCTTTTACCAACTTAAGAGCATCAGG

AATCACGGTTTGATTGTTTGCTGCGGTTACGGCTGCTTGTTCAAGGTCGATATCTAGGCCATCGAAGCCG

TAACGCTCAGTTAGACGGATAATCTCATCAGCAAAGGCTCTTTCATCACCGGTTTCTAGCTCTACGTGTG

CATCAGCACCACCCAAAGCCAACAGTACAGAGCGACCTTGCTTGTTGAGCTCAGAGATTTGGTCAATAAA

TTGTTCTTCTGAAAGCCCAATGGTTGGGTCCAGTTTAAAAGTTGGGATTCGACCATCAGCCACATCGTAA

ACCTTCATGAATGACACATTCACGATGTTGTACATTGGGTTCACTTCATCCAACGTCACACATGGCGCAT

TACCACCTTGGTAGCCGCCACCGTCACACCAGTTATGCCAATAGCCAACCACGACGCCAGAGTCTGGATT

CGTCATATCTGAACCATTCGCATAAACAGCGCCAGACATAGCCGCCATGCCGACCGATACTGCTAATTGT

AGAGCTTTACGTTTCAACAT

>CP031055.1:1335696-1337885 Vibrio splendidus strain BST398 chromosome 1, complete sequence

ATGTTGAAACGTAAAGCTCTACAATTAGCTGTATCGGTCGGCATGGCCGCAATGTCTGGCGCTGTTTATG

CGAATGGTTCAGATATGACGAATCCAGATTCTGGTGTCGTGGTCGGGTATTGGCACAACTGGTGTGACGG

TGGCGGCTACCAAGGTGGTAATGCGCCATGCGTGACGCTGGATGAAGTAAACCCAATGTATAACATCGTG

AATGTGTCATTCATGAAGGTTTACGATGTGGCTGATGGTCGAATCCCAACTTTTAAACTGGACCCAACCA

TTGGGCTTTCAGAAGAACAATTTATTGACCAAATTTCTGAACTCAACAAACAAGGCCGTTCTGTACTGAT

AGCGCTAGGTGGTGCTGATGCACACGTAGAGCTAGAAACTGGTGATGAAAGAGCCTTTGCTGATGAGATT

ATCCGTCTTACTGAGCGTTACGGTTTCGATGGCCTAGACATCGACCTTGAACAAGCCGCGGTAACCGCAG

CAAACAACCAAACGGTCATTCCTGACGCCCTTAAGCTAGTGAAAGACCACTACCGTGCTGAAGGTAAAAA

CTTCCTAATTACGATGGCGCCTGAGTTCCCGTATCTAACGACAGGCGGTAAGTACGTTCCATATATCGAT

AATCTAGAAGGTTATTATGACTGGATTAACCCACAATTCTACAACCAAGGTGGCGACGGTATCTGGGTTG

ACGGCGTGGGCTGGATTGCTCAAAACAACGATGCATTAAAAGAAGAATTCATCTACTACATCTCTGATTC

GCTTATCAACGGAACGCGTGGCTTCCACAAAATTCCACATGACAAGCTGGTGTTTGGTATTCCTTCAAGT

ATCGATGCTGCAGCGACGGGCTTTGTGCAAGATCCTCAAGATTTATACGACGCGTTCGAGACCTTAACAA

CTCAAGGCCAACCGCTGCGTGGTGTAATGACTTGGTCTATCAACTGGGACATGGGTACTAACAAAAACGG

TCAACAATACAACGAGCAGTTTATTAAAGATTACGGCCCGTTTGTCCACGGTCAAGTGACGCCACCACCA

GTAGAGGGTGAGCCAGTTCTTAAAGGTGTCGAAAATGCCCGTGTTCTTCACGGCACAACGTTTGACCCAA

TGGAAGGTGTAACGGCAACCGATAAAGAAGATGGCGATCTAACATCTTCTGTCGATGTTGAAGGTTACGT

GGAAACCAGTGTGATTGGCACTTACGTTCTAACTTACCGAGTGAAAGACAGCGATAACAACGAAACCACT

AAAGCGAGAACGGTAGAGGTTTACAGCCAGAAGCCTGTGTTTAATGGTGTGTCTGACACTACTGTTGTGT

TGGGAACTGCATTTGATGCTATGGCAGGCGTAACGGCGAATGATGCTGAAGATGGCGACCTAACAAGCAC

CATCACACACACTGGCAGTGTCGATGTAAATGAAATCGGCAACTACACGCTTGTGTATAGCGTAACAGAT

AGCGCGAACCAAACTGTAACCGCTGACCGTAAAGTGTCTGTGACGGATGGTTCTAACTGTGCAGCTGCAT

GGGATGCCGATACCGTTTATGTTGAAGGTGATCAAGTCTCTCATGATGGTTCAACGTGGGGAGCAGGTTG

GTACACTCGCGGCGAAGAGCCAGGAACAACAGGTGAGTGGGGCGTTTGGAGAAAAGTTTCAGACTCTTCA

TGTGGTGGTAACCCAGATCCGGGCGATGACTTAGAGCTTGCAGTTTCAGGCCTTCAGTCAGAATATGCGC

CAGATAACGGCAATGTTCGTTTAGACCTAACACTGACATCAAATGAAGCTTTAGATGTTACGGCTATGGT

GATCAACAGTGCAGGGACTGTGGTTGAGCAAACTCAGGTTAACTTAACGGACAGCCGCGCTATTACCATG

GACTTATACGATGTAGCTGAAGGGCAATATGCACTTGAAGTGGTCGGTAAAGCATCTGACGGTGAAATGG

TGATGGTAAATGATTCGTTCGCTGTTAAAGACGGTGGCGGAACGACTCCACCTCCAGGTGATTACCCGCC

ATATGAAGCCGGCACAAACTACGCAGCAGGTGACATTGTAGTGGGCAGTGACAATGGTTTATATGAATGT

AAGCCTTGGCCATACACTGCTTGGTGTGCAAGTGCATCTTACGCACCAGCAGAAAGCCAATACTGGCAAG

ATGCTTGGACAAAGCTGTAG

>CP016229.1:351026-352390 Vibrio crassostreae 9CS106 chromosome 2, complete sequence

ATGAAACTGTCATTTATCACACGCGCTATTTCTAGCAACAGTGACCAATCAAGTTCTATCTTTAAATCTA

ATTCCGCCATTGCGCTTAGCTTAATCACCACGGCTTGTATGCTCACACCTGAGGCACATGCCGATGATAA

AGTCGTCGCAGGGTATTTTGCGGATTGGCAATATCTGAATAAAGACAACCCTTATACCGTTGACGACATA

CCTGCAGACAAGCTCTCACATATTATTTACGCCTTCTTGAGCATGTGTGGTCCTCATAGCGGTGCTGGTG

AGCCAGTTCAAAAACAAATTAAGCAGCAATGTGCAGGCAAACAACCATATACCGCGATTATTGTGGATAA

AGAAGCTGCGTTAGAAGTCGATTTCGGAGACGTGGGTGTTGATGTCGCCTATAAAGGTCACTTTGCCCAG

TTAGCTCAACTGAAGGCCGACAATCAAAATATTAAAATTTTGCCCTCATTTGGAGGGTGGACAATGTCTG

AACCTTTCCATGCGATGGCGAAAGATCCTAAAGCCATTGCGCATTTTTCAAAAACTGCTGTTGAGTTGAT

TCAAAAATATGACTTCTTTGATGGTATTGATCTTGATTGGGAATACCCAGGAGGCGGCGGCTTGACCACT

TCCCCTTGGAACCCAGAGACGAAACTAACGGATGAGCAGAAAGCGCAAGAGCGTGAAGCTTTCACACTTT

TAGTAAAAACAATCAGAAGTGATTTGGACGCTCTATCAAAGACGACTCAAAGAGATTACGAGCTTTCAAC

GGCTGTCGGTGTTGGTGCTAAGGCAGCTCAAATTGATTGGAACGCAGCTAGCCCATATTTGACGAACATG

TTTGCGATGACGTATGACTTCCTCGGAGGCTGGGGAACTCAAACAGGTCACACAACAAACTTACATGCCA

CCGAGCGTAGTTGGTGGGGAATGGGAGCTGATGTCTTCATTAATCAGATGATAGAGCAAGGTATTCCTAG

CGAGAAGCTCGTGATCGGGGCGGCTTTTTATGGTCGAGGCTGGCAAGGGACAAAAGACTTTGCTGGAGAG

CTGCCTAAAGGTGACCTAGTTTCTGAACGAGGAGCGCAGTTTGGTACCGGTGAAAACGGTTACTTTATGT

TCTGGGACCTTATGAATAACTACAGCAGCAAACAAGGTTATGAGTATAAGTACGATGAACAAGCTCAAGC

ACCTTATTTGTGGAACCCAGATAAAAAAGTATTCATATCTTTTGAAGATCAGCGTTCTATTAAAGCAAAG

GCAGAATGGGCGAAGAAATCAGACCTAGGCGGCATTTTTACTTGGGAATTATCAGGAGATCCTTCCGGGC

AGTTGATCGATGTTATGCATAGTGAAATGAATAAA

>NC_006840.2:c720921-718381 Vibrio fischeri ES114 chromosome I, complete sequence

ATGAAAGGGATATTCATTAAGAGTGCTATTGCTTCTGCTCTCTTTATCTCATATGGAGCAAATGCATTTG

ATTGTAGCTCGACGCCAATTTGGCAAAGTACTCAAGCTTATAGTGGGGGGGATACTGTTCAACAAAACAA

TATTGCTTACCAAGCCCAATGGTGGTCACAAGGAAACGATCCAGAAACACATTCCGGTGAATGGCAAGAA

TGGAAGAATCTTGGTAAATGTAGTACAGGAGGTGAGAATATCGCACCAACAGTGACAATCATATCCCCAA

TAAATAATGCCATAATCCCTGAAGCTAGCCTGATAACACTTCAAGCGAGTGCTGATGATAAAGACGGTTC

CATTACTCAAGTAGAATTCTTATCAAACAATCAATCTATTGCCATTGTTACCAGCCCTCCTTATAACACA

CCTTGGATTGCTATTAAAAACACCACATCAATTATTGTTATTGCCACAGACGATGAAGGTGCCACCACCA

CAGAATCTGTTTCTATAAAGGTTCAATCAGCAGGTGAACTTGTCCCTCCAACAATTGAACTTACCTCTCC

AATAGGAAATGAAAAACTAACAACAGGCGACACATTAGTCGTTTCAGCCAATGCAAATGACAGTGATGGC

GTAATTAATAATGTCGAGTTCTACATTGATAATCAACTTGCTTTTACTGATACCGAAGCACCTTTTGAGT

ATTCATGGTTAGCGACAAGCGGTCCTCACCAATTTAAAGCAAAGGCGACAGATAATGATAATCAGACAAC

CATAAGTAAAGGCGTTACACTTGAGGTATCTGAACCATCCAGTGGTGGGTGTGCAGGCCTCCCTCAATAC

AAGTCTGGAACAAACTATGCGGCTGGTGATGAAGTTCAAAACTTAAATCAAAAATATCGTTGTGATATTG

GTGGTTGGTGCTCATCATCATCAGACTGGGCTTACGCTCCTGGAACGGGATCTAATTGGCAAGATGCATG

GACAGGATTAGGCGTTTGCAGTACACCGCCACAAGTAACGATTACCTCACCAAATGATAATAGTGTAATT

CTTGCTGGTTCTATCGTCGAAGTGAATGCGAGTGCAAGTGATAGTGATGGAAGTGTTATTGGTGTTGAGT

TCTTTGCAAACAATGTCACTCTTGGATCTGATACACAAGCACCATATACCGTTAATTGGGATGCTACAGT

AATTGGAAGTAATACATTAAAAGCAGTAGCAACAGATAATGAAGGTAATAACAGTGAAAATAGTATTTTG

GTTACGGTTAGTGATCAAAATGTGGTTACAGCACTCACCTCCCCAATAACAGGTTCGAGTTTAGTTCTGG

GTAAAATAACTCAACTCAAAGCGACAGCAACATCTCTAAGCAGTTCTATCACCAAAGTAGACTTCTTAGT

TAATGGCGTTATTATCGCCTCAGATACAACATCACCGTATAGTGTCAATTGGACACCAGCAGCAATTGGA

GCTTACACCTTATCGGTCGAAGCTACAGATGCGCAAGGTAATACAGCATTATCTACTGCTTCAAGTGTCT

CTGTTGTAGAACAAACACTAGCGAAACATAAACTTATCGGTTATTGGCACAATTTTGTAAATGGTTCAGG

ATGTCCAATTCGCTTAGCTGATATGTCAGATGAATGGGATATTATTGATATCGCTTTTGCTGACAATGAC

AGAAACAGTAATGGTACCGTGCATTTTAATCTGTACTCAGGAGATATTCATAGCAGTTGTCCAGCATTAG

ATCCAACTCAATTTAAACAGGATATGGCAGCACTACAAGCAAAAGGCAAAGTATTTGTTCTTTCACTTGG

CGGTGCAGAAGGAACCATTACATTAAATACTGATAATGATGAGGTTAACTTTGTAAATAGTCTAACGGCT

ATCATTGATGAATGGGGATTTGATGGACTCGATATTGATTTAGAAAGTGGGTCAAATCTACTTCATGGTA

CTCAAATACAAGCTCGATTACCTACAGCACTTAAACAAATAGAGGCTAATATGGGTGGCGATATGTATTT

AACCATGGCGCCAGAACATCCTTATGTGCAAGGTGGAATGATCGCTTACAGTGGTATTTGGGGTGCCTAT

ATACCGGTGATTAATCAAGTTCGAGATACATTAGATTTATTACACGTTCAACTTTATAACAATGGCGGGC

TACCAAATCCATACATGCCTGGTTCAGCACCTGAAGGTTCTGTCGACATGATGGTAGCGTCAGTAAAAAT

GCTAGTTGAGGGTTTTGAATTAGCAGATGGTACCTTCTTCGCTCCATTAAGGGATGATCAAGTTGCGATT

GGATTACCATCAGGCCCAAGCTCTGCAAACTCAGGTCAAGCTCCAATTCAAAACATTGTCGATGCACTAG

ACTGTGTTACTTATGGCACCGCATGCAGTTCAGTGGTTCCAAGTAAACTCTACTCAAACTTTGGTGGAGT

AATGACATGGTCAATCAATTGGGATTTACATGATGGATTTAATTTTTCCAAACCCGTTGGTGATAAATTA

AATCAAATGAATAATCAATAA

>NZ_CP009355.1:848952-852080 Vibrio tubiashii ATCC 19109 chromosome 2, complete sequence

ATGCATAAATCAACGTCAGGAACGGCTAGAAGAGTATTTACCCTTAGTACTCTTAGCGCGTCATGTTTAA

TGGCATTTAACAGTTACGCCGCAGTTGACTGCAGCCCTCTCGGAGCTTGGGATTCATCGACCATTTATAA

CACTGGTGATCAAGTTCAACACTTGGGCGAGGGTTATCAGGCTAATTATTGGACGCAAGGAAATAACCCA

ACCCAAACGTCGGGCGAATGGGGTCACTGGAAACATTTAGGAGCGTGTGATGCTACTGGAGGAAATGAGC

CTCCAACGGTGTCAGTGACGTCTCCTCTTGCCTCTGATGCAATCACTCAAGGTGACGCTGTCACTATTTC

TGCAAGCGCGTCAGATGTCGATGGAACGGTCACCAAGGTGGAGTTTTCTGTTGATGGTGTTGTGGTTGGT

CAAGCGACACAAGCTCCTTATAGTGTGGCTTGGACCGCTACCGTTGGCAGTCATCAAATCGGTGCGGTTT

CTTATGATGATGCGGGCGCTCAGAGTACAGTCAGTAGCGTGACAATAAATGTCGTCTCTGCAAATCCTGA

TAACATTGCACCAACAGTCGATGTTGCGCTATCGACCAACTCGGTAGATGTAGGAAGTGCAGTGACACTT

ACGGCTAATGCAGCGGATGCGGATGGGTCAGTTGATAAAGTAGACTTTTATGTTGGTGGTAGTTTGGTGG

GTACTGCCGCTACTGCGCCTTACACTTTGAATTACACAACGGTGCAAGCTGGCAATCTTGCGGTGTATGC

CGTTGCAACAGACAATCTAGGGGCGAGTACTAATTCGACGCCTGTTACTCTCGTCGTTAACGGTGTACCG

CCGGTTGCTAATTGTCGTCCAGACGGTTTATATCAGACACAAGGCGTGAATGTTCCTTACTGTACCGTCT

ACGATGGCGAAGGTCGTGAGCAGATGGGAGCGGATCACCCGCGTCGCGTGATTGGATATTTCACTAGTTG

GCGTGCGGGTGATGACCCACAAGCCTCATACCTCGTCAAAGATATTCCATGGGAACAACTGACTCATATT

AATTATGCCTTTGTTGGTATTGGTTCTGATGGGAAAGTGAACTTAGGTGATGTCAACGATCCAAACAATG

CAGCAACCAGTAAAGAATGGCCAGGGGTAGATATTGACCCAACACTGGGTTTTAAAGGGCACTTTGGTGC

GTTAGCGACTTATAAACAGCGCTATGGTGTTAAGACGCTGATTTCTATTGGTGGCTGGGCTGAAACTGGT

GGTCATTTTGATGCCAATGGTAATCGTGTTGCTGATGGTGGTTTCTACACCATGACAACCAATGTCGACG

GATCAATTAACCACGCAGGTATAGAAAAATTTGCCGCTTCAGCCGTTGAAATGATGCGTACCTATAAGTT

CGATGGTCTGGATATTGACTACGAATATCCGACGTCAATGGCAGGTGCAGGAAACCCTTATGATAAAGCC

TTTATGGAGCCGCGACGCCCATATTTGTGGGCTTCTTACCAAGAGTTAATGAAAGTTTTACGTGAGAAGC

TCGACGCAGCTTCAGCGCAAGACGGTATTCATTACATGTTAACTATTGCGGCGCCATCATCTGGATATCT

ATTGCGAGGCATGGAAACTTTTGATATCACCAAATACCTCGATTACGTCAACATCATGTCCTACGACCTA

CATGGAGCATGGAATGATCATGTAGGACACAATGCTGCGTTGTTCGATACAGGAGAAGACTCAGAGTTAG

CGCAGTGGAATGTCTATGGTACGGCCGCCTATGGTGGTATTGGCTACTTGAACACCGACTGGGCTTACCA

CTATTTCCGCGGTTCGATGCCAGCAGGTCGTATCAACATTGGTGTTCCATACTACACTCGCGGTTGGCAA

GATGTGACAGGTGGGACTAATGGTCTTTGGGGCCGCGCTGCCTTACCAAATCAAGCCCAATGCCCTGAAG

GAACAGGGGAAGGCGAGAAGAACAATTGTGGTAATGGTGCGATGGGCATCGATAACATGTGGCATGATAA

AGATCCTCAAGGCAACGAAATGGGGGCGGGCTCAGGCCCAATGTGGCATGCTAAAAACCTAGAGAAGGGA

GTATGGGGTACGTACAGCGCCGCTTATGGCTTAGATCCTGTTAATGATCCGAGTGATCAATTGGTTGGCA

CCTATACAAGACACTATGACAGCGTTGCGGTTGCACCTTGGCTATGGAACGCACAAAAGAATGTTTTCCT

TTCCACTGAAGATGAAGCTTCGGTTTCTGTTAAAGCGGACTACGTCATTAACAAAGAGATCGGTGGGATT

ATGTTCTGGGAGCTAGCAGGGGACTACAACTGTTATCTGATTGAGAATGGACAGCGTACCTCTGTCGACT

TGACTGAGCAAGCCTGTGCAAATGGAACTGGTGAGTACCATATGGGTAACACCATGACGAAAGCTATCTA

CGATAAGTTTAAGTCTGCGACGCCATATGGCAATAAGGTTTCAACGGGCGCAATACCTACCGAGGCCGTT

GATATAGCAGTGACGATCGGCGGCTTTAAAGTGGGTGATCAGAACTACCCAATCAACCCTAAAGTGACCT

TCACGAACAATACAGGGCAAGCCATTCCTGGTGGTACTGAGTTCCAATTCGATATCCCAGTTTCGGCTCC

TGATAATGCTAAAGATCAATCGGGTGGCGGGTTAAAAGTCATCGCGTCTGGCCACACAAGAGCGAACAAC

ATTGGTGGCCTTGATGGACCAATGCACAGAGTCGCATTTAGTCTGCCAAGCTGGAAAGAACTGCCTGTCG

GTGGGACTTACGAGCTTGATATGGTCTACTACCTACCTATCTCTGGGCCTGCGAATTACACTGTAAATCT

GAGCGGAGTAGATTACGCATTTAAATTTGAGCAACCAGACTTACCGCTAGCGGATTTGTCTAATGGTGGA

AACAACGGTGGTAGTAATGGAGGTAACACCGGTACTGTAACAGCATGGGAGCCAGGCGTGACCCAAGCAA

TCAATGGTGACACCTATTCTTACAATGGAAAATGTTTCGTTGCCAAGAATAATCCCGGTACATGGGAATC

ACCGACTCAGTCAAACTGGTTTTGGGATGAGGTTACTTGCCCTCAGTAA

>NZ_LLEI02000016.1:78626-81763 Vibrio bivalvicida strain 605 contig_16, whole genome shotgun sequence chitinase

ATGTACATACAAAGGTCTGGTTCGGCTAAACTTTTTACCCTCAGTACGCTGAGCGTCGCCTGTTTAATGG

CGTTTAACGGCTATGCTGCGGTCGTCTGTGGCAATCTTGAAAACTGGGACTCCTCGGTGGTCTATACGGG

GGGCGACCAGGTCCAACATTTAGGCTCGGGGTATCAAGCGAATTATTGGACTCAAGGAAATGATCCTATA

CAAACCTCAGGAGAGTGGGGGCATTGGAAAAATTTAGGCAGTTGTGATTCGGCAGGCGGTAATGAAGTAC

CAACCGTGTCAATTACTTCACCTTTGGTAACAGATTCAATAACAAAAGGTGACGATGTCACCATTGCTGC

TAATGCAAACGATATTGATGGCAGTGTGGCAAAAGTTGAATTTCTCGTGGATGGAACGCTTGTCGCGCAA

GTGACGGCTGCTCCATACACAACATCCTGGAATGCGACGCTGGGAAACCACCAAATTAGTGCTATCGCGT

ATGATGATGTGGGTGCGCAAAGTTTGGTTAGTCACGTAGAGATCTCTGTTACGGGTTCAAATCCCGATAA

TGTAGCACCAACGGTAGATGTTTCATTGTCGGCGACCAGTGTTGATATAGGATCTGTTGTTACCTTGACC

GCCAACGCTGATGATTCTGACGGGACTGTGAATAAAGTCGACTTTTATGTTGCAGGCCTGTTGGTTGGTA

CTGCTGCGTCGGCACCATACACGCTGGACTACACGACTACTCAAGCTGGCAGCCTTTCTATCTATGCTAA

GGCGACAGATAATCTTGGGGCAAGCACCAACTCCACTATCGCGAGTTTAACGGTGACTGGCGCATCACCT

GAAGCCAACTGCCGCCCTGACGGCCTTTATCAAACTGAAGGCGTTAGCGTACCTTATTGTACCGTGTATG

ACGAACAAGGGCGTGAGCAAATGGGGTCTGATCATCCGCGTCGTGTTATTGGTTATTTCACCAGTTGGCG

AGCAGGTGATGATCCTCAAGCTTCTTACTTAGTTAAAGATATTCCTTGGCAGCAATTGACTCACATTAAC

TATGCATTTGTGGGTATAGGTTCAGATGGCAAAGTTAACTTAGGTGATGTTAACGATCCTAATAACGTAG

CGACTGGCAAAGAGTGGCCTAGTGTTGATATAGACCCAACGCTTGGTTTTAAAGGGCACTTTGGCGCACT

GGCGACTTACAAACAACGTTACGGTGTTAAAACTTTAATTTCGATTGGTGGATGGGCTGAAACCGGCGGA

CATTTTGACGCGGATGGAAACCGTGTTGCTGACGGTGGGTTCTACACCATGACAACAAATGCCGATGGCT

CAATTAATCATGCAGGGATTGAGAAGTTTGTCGCTTCTGCTGTTGATATGATGCGTACTTACAAGTTTGA

TGGTCTTGATATTGACTATGAGTACCCAACTTCAATGGCGGGAGCCGGAAACCCATATGATAAAGCCTTT

ATGGAGCCGCGCCGCCCCTATTTATGGGCTTCTTATCAAGAATTAATGAAGGTACTCCGTGAAAAACTGG

ATGCTGCTTCTGCTCAAGATGGTGTTCACTATATGCTGACCATCGCGGCTCCTTCTTCTGGTTACTTACT

TCGCGGTATGGAGACCTTTGATGTTACCAAGTACTTAGATTACGTCAACATCATGTCTTACGATTTACAT

GGTGCTTGGAATGACCACGTAGGTCACAATGCCGCACTATTTGATACAGGTGAAGATTCGGAACTGGCAC

AGTGGAACGTATATGGAACCGCAGCTTATGGCGGAATTGGATACCTAAACACTGACTGGGCCTACCATTA

CTTCCGAGGTTCAATGCCCGCTGGTCGAATTAATATCGGTGTCCCATATTATACCCGAGGATGGCAAGGA

GTAACGGGTGGTACCAATGGTCTATGGGGGCGTGCGGCATTACAGGATCAAACTCAATGTCCGGCAGGAA

CAGGTGAGGGTGAGAAGAACAATTGCGGTAATGGCGCTTTGGGTATCGACAATATGTGGCATGATAAAGA

TCCACAAGGCAATGAAATGGGAGCGGGTTCAGGTCCTATGTGGCATGCAAAAAACCTAGAGAAAGGGATC

TGGGGGACTTACAGTTCGGCATATGGCTTAGACCCGGCAAATGATCCTACAGATCAATTCGTGGGAAGCT

ACACTAGGCACTATGACAGCGTCGCTGTTGCCCCTTGGTTATGGAATGCAGAAAAGAAAGTGTTCCTATC

TACGGAAGACGAGCCATCTGTCTCGGTGAAAGCGGATTATGTTATCGATAAGGAAATAGGCGGTATCATG

TTCTGGGAACTTGCAGGGGACTATAACTGCTATGTTCTTGACAACAATCAGCGCACTTCAATTGACGTGA

CTGAGCAGGCTTGTGCAAATGGAAATGGTGAATACCATATGGGTAACACCATGACCAAAGCGATATATGA

TAAGTTCAAGTCAGCCACCCCTTATGGCAATAAGGTTGCTACCGGGGCAGTACCGACAGAAGCAGTAGAT

ATTTTAGTCAAGATTAGTGGGTTTAAGGTTGGCGACCAAAATTATCCGATCAATCCGAAAGTGACCTTTA

CCAATAACACGGGGCAGGCGATCCCTGGTGGTACCGAATTCCAGTTTGATATCCCAGTATCGGCGCCAGA

CAATGCTAAAGATCAATCCGTTGGTGGATTAAAAGTCATTGCTTCTGGTCATACGCGAGCGAACAACATT

GGTGGACTAGATGGCCCAATGCATAGAGTCTCGTTTAGCTTACCAAGTTGGAAAGATCTTCCTAGTGGTG

GGACGTATGATCTTGATATGGTTTACTATCTACCTATTTCAGGGCCAGCAAATTACACTGTAAATATTGC

TGGGGTAGAGTATGCATTTAAGTTTGAGCAACCGGAGTTGCCTTTAGCTGATTTGTCCAATGGTGGTAAC

AATGGGGGCAATAACGGTGGCGACAATGGCGGAGATAGTGGAAACGCTACAGCTTGGGAACCCGGTGTTA

CTCAAGCGCAAAATGGCAATACCTATTCACATAACGGTAAGTGCTTTGTCGCAAAGAATAACCCGGGTAG

TTGGGAAACGCCTACTCAGTCAAATTGGTTCTGGGATGAAGTGACGTGCACACAATAA

>NZ_FAUO01000005.1:563387-566554 Vibrio crassostreae strain Evh12, whole genome shotgun sequence

ATGCACCTAAATCAAGGAAGAGTGGCCAAGAAGGTTTTTACACTCAGTACGCTCACCGCGTCATGCTTAA

TGGCGTTCAACAGCTATGCGGCTGTGGATTGCTCAACGTTAGAAACGTGGGACTCTGCCACTGTATACAC

TGGTGGTGATCAGGTTTCACATGACGGCAGTGCTTATACGGCAAATTACTGGAATCAAAATAACAACCCA

AGCCAATTTGAAGGCGACTACGCACAATGGAAGAAAGTCGATGTGTGTAGCGGCGACGGAGGTGGCGGTA

CGCCGAATGAAGTGCCAACAGCTTCATTAACGGCACCCTCTGCTTCTGACGTGATCACTGAAGGCGATAA

TGTTGTATTGAGTGCAACAGCATTAGATTCTGATGGCAGTGTGGCTTCGGTTGAGTTCTTTGTTGATGGC

GCATCTGTTGCGTTAGTCACAGCGGCACCTTTCGAAGCGACTTGGGCTGCAACGTCGGGTAATCATCAGG

TTTCTGTTGTTGCAACGGATAATGAAGGCGCAGCAAGTGTTGCAAGTGCGGTTTCTGTCTCTGTTGATTC

AGCACAACCAGGGAATGAAGCACCGACAGTTTCAGTTGCACTTTCAGTGACATCTGTTGATGTTGGTGGT

GTAGTCACACTGACAGCAACGGCAGCAGATAGCGATGGCACGGTTGATAAGGTTGATTTCTACGTAGCGG

GTGCTCTTGTGGGTACAGCAGCAACGAGCCCTTACACGTTAGATTACACAACGACTCAAGCGGGTTCTTT

AGCGGTTTACGCAAAAGCAACAGACGACCAAGGTGCGACTGCAGATTCTGCGTTGGCATCGTTGACTGTG

AATGGCGCTCCAACAGTGAGCACATGTCGCCCTGATGGCTTATATCAAACTCAAGGTGTCGATGTTCCTT

ACTGTACGATTTACGATGATGAAGGTCGTGAGAAAATGGGCGCGGATCACCCACGTCGTGTTATTGGTTA

CTTCACAAGTTGGCGTGCAGGGGATGACCCACAAGCGGCTTACTTAGTAAATGACATCCCTTGGGAACAA

CTCACACACATTAACTACGCTTTTGTGAGCATCGGCTCAGATGGCAAAGTAAACGTCGGTGATGTGAATG

ATCCTGAAAACGCAGCGGTTGGCAAAGAGTGGCCGGGTGTGGAAGTTGATCCTGCATTAGGCTTCAAAGG

TCACTTTGGTGCATTAGCGACAGCGAAGAAAAAACATGGCGTGAAAACGCTAATCTCAATCGGTGGTTGG

GCAGAAACCGGTGGTCACTTCGCGACTGACGGCAGTCGAGTGGCTGATGGCGGTTTCTACACAATGACAA

CCAATGCCGATGGCTCTATCAATCATCAAGGTATCGAAACATTCGCCACTTCCGCGGTTGAAATGTTGCG

TAAATACCAGTTCGATGGTCTGGATATCGATTACGAATACCCAACCTCAATGGCAGGTGCCGGTAATCCA

TACGACAAAGACTTCATGGAGCCGCGTCGTCAATACCTATGGGCTTCGTACCAAGTGTTGATGAAAGTGC

TACGTGAGAAGCTTGATGCAGCCTCTGCGCAAGATGGCAATCACTACATGTTAACGATCGCGGCGCCTTC

TTCTGGTTACCTGTTACGCGGTATGGAAACATTCGATGTAACCAAGTACCTCGATTACGTGAACATCATG

TCTTACGACCTTCACGGTGCGTGGAACGATCACGTAGGTCATAACGCAGCCTTGTTTGATACAGGTAAAG

ATTCAGAGCTAGCGCAATGGAACGTTTACGGCACGGCGGCTTACGGTGGTATTGGTTATCTGAACACGGA

TTGGGCTTACCATTACTTCCGTGGTTCTATGCCAGCAGGCCGTATTAACATCGGTGTGCCTTACTACACT

CGTGGTTGGCAAGGCGTAACGGGTGGTGAGAATGGTCTTTGGGGCCGAGCAGCACTGCCAAACCAATCTG

AATGTTCAGCGGGCACTGGGGAAGGCGAGAAAAACAACTGTGGCCATGGCGCTATTGGTATCGATAACAT

GTGGCACGATACCGATCCAAAAGGCAACGAAATGGGCGCGGGTTCTAACCCAATGTGGCATGCGAAGAAC

CTAGAGAAAGGTATTTGGGGTTCTTACGCTGAAGCTTACAAGCTTGATCCAGTGAATGATCCTTCAGATG

TTCTAACTGGCACTTACACGAGAAACTATGACAGCGTGGCGGTTGCGCCTTGGTTGTGGAACGCAGAGAA

GGGCGTATTCCTTTCAACGGAAGATAAACAGTCTATCGACGTGAAAGCGGACTACGTTATCGACAAAGAG

ATCGGCGGCATCATGTTCTGGGAACTAGCAGGGGATTACAACTGTTATGTACTCGATGCGAATGGCAACC

GAACCTCTATCGATACGACTGAGCAAGCGTGTAATAGTGGTAACGGTGAGTTCCACATGGGTAACACCAT

GACGAAAGCTATCTACGATAAGTTTAAGTCTGCGACTCCATACGGAAACAAAGTAGCGACGGGTGCTATT

CCAACAGAAGCATTAGATATTTCGGTATCGGTTGGTGGCTTCAAAGTGGGTGACCAAAACTACCCAATTA

ACCCTAAGATCACGTTCACGAACAACACAGGGCAAGCTCTTCCGGGTGGAACTGAGTTCCAATTCGATAT

CCCAGTATCTGCACCTGATAACGCGAAAGATCAATCGGGTGGTGGTTTGACCGTGATTGCTTCAGGTCAT

ACTCGTGCGGATAACATCGGTGGACTAGATGGAACCATGCACCGAGTGGCGTTTACTTTGCCGACATGGG

AAGAGCTTCCAGCTGGTGGTGTGTATGAGCTAGACATGGTGTATTACTTGCCAATTTCTGGCCCTGCTAA

CTACGCGGTGAATGTCGGTGGCGTTGATTACGCGTTTAGCTTTGAGCAGCCAGATCTACCATTAGGTGAT

ATCAGTTCAGGTGGCGGTAACCCTGGCGATGGTGGCACCAACCCAGGTACATGTGATACAGCTGGTTTAG

CGGTTTACCCAGACTTACCTCAGAAAGATTGGGCGGGTAACCCAAGCCACGCAAACACAGGCGACCAAGT

GGTTCATAACGGTAGTGTTTATCAAGCAAACTGGTGGACAAGTTCTGAACCGGGTAGCGACGGTAGCTGG

ACTCAAGTTTGTTCTTAA

>NC_005957.1:3554763-3555845 [Bacillus thuringiensis] serovar konkukian str. 97-27 chromosome, complete genome

ATGTTAAACAAGTTCAAATTCATTTGTTGTACGTTAGTCATTTTTTTACTGCTACCACTAGCGCCCTTTC

AAGCACAAGCAGCAAACAATTTAGGATCAAAACTACTCGTTGGATACTGGCATAACTTTGATAACGGTAC

TGGCATTATTAAATTAAGAGACGTTTCACCAAAATGGGATGTAATCAATGTATCTTTCGGTGAAACTGGC

GGTGATCGTTCCACTGTTGAATTTTCTCCTGTGTATGGTACAGATGCAGAGTTCAAATCAGATATTTCTT

ATTTAAAGAGTAAAGGAAAAAAAGTAGTTCTTTCAATCGGTGGACAAAATGGGGTCGTTTTACTTCCTGA

CAATGCCGCTAAGCAACGCTTTATTAATTCCATACAATCTCTAATCGATAAATACGGTTTTGATGGAATA

GATATTGACCTTGAATCAGGTATTTACTTAAACGGAAATGACACTAATTTCAAAAACCCAACTACTCCTC

AAATCGTAAATCTTATTTCGGCTATTCGAACAATCTCAGATCATTATGGTCCAGATTTTCTATTAAGCAT

GGCTCCTGAAACAGCTTATGTTCAAGGCGGTTATAGCGCATACGGAAGCATCTGGGGGGCATATTTACCG

ATTATTTACGGAGTGAAAGACAAACTAACATACATTCACGTTCAACACTACAACGCTGGCAGTGGTATTG

GAATGGACGGTAATAACTACAATCAAGGTACAGCAGACTACGAAGTCGCAATGGCAGATATGCTCTTACA

CGGTTTTCCTATAGGTGGTAATGCAAATAACATGTTCCCAGCTCTTCGTTCGGATCAAGTCATGATTGGA

CTTCCTGCAACACCAGCGGCTGCTCCAAGTGGTGGGTATATTTCTCCAACTGAAATGAAAAAAGCTTTAG

ATTATATCATTAAGGGAATTCCTTTCGGAGGAAAGTATAAACTTTCAAATGAGAGTGGCTATCCTGCATT

CCGCGGTCTAATGTCTTGGTCTATTAATTGGGATGCAAAAAACAACTTTGAATTCTCTAGTAACTATAGA

ACATATTTCGATGCGATTCCCTTGCAAAAATAA

>NC_004347.2:4238458-4240647 Shewanella oneidensis MR-1 chromosome, complete genome

ATGAAATCTTTTAAATTCAATACACTGTTTAGCTGCATAGCACTCTCCACAGCGGTATTAGCCTCCCCAG

CAAGTGCACAGGGGTCAATGATGGTTAACCCGCAAGCTGGTGTCGTCGTGGGTTACTGGCATAACTGGTG

TGGTGGCGCTGGCTATAAAGGTGGCGTTGCTCCATGTGTTAGCTTGGCGCAGGTGAACCCTCAATATAAT

GTTGTCGATATCTCATTCATGAAGGTATATGGCGCAACAGGAAGTATCCCTACTTTTAAACTCGATCCCG

CTGTTGGCTTATCTGAAGCACAATTTATTGACCAAATTGGTGAACTCAATCGCCAAGGCCGCGCGGTATT

AATCGCATTGGGCGGCGCTGACGCACACATTGAGCTTCGCAAAGGACAAGAACACGATCTGGCCAATGAA

ATTATTCGTCTGGTTGAAAAGTACGGCTTTGATGGACTGGATATCGATTTAGAGCAAGCTGCCGTAACGG

CTGCAGATAACCAAATCGTCATTCCCGCGGCGCTACGGATCGTTAAAGATCATTACCGTAAACAGCAGAA

AAACTTTTTAATCACCATGGCACCCGAATTCCCTTACCTCACTACAGGGGGAAAATACGTGCCTTATATC

AACGCCCTTGAAGGTTATTATGATTGGATTAATCCACAGTTTTACAACCAAGGCGGCGATGGCATTTATA

TCGAAGGCATAGGTTGGATTGCCCAAAACAACGACAGTTTAAAGCAAGAATTTATTTACCATATCTCTGA

CGCGCTAGTGAATGGCACAAACGGCTTCACTAAAATCCCCCATGACAAGCTAGTTTTTGGTATTCCAACC

AATATTGATGCCGCAGCTACAGGGTATGTCAGCGATCCAAATGCATTGTTTGCAGCCTTTGAACAATTAA

AGAACCAAGGACAACCGTTACGCGGCCTTATGACATGGTCAGTGAACTGGGATATGGGCAAAAATGCCGC

TGGGCAGGCCTATAATGAGTCGTTTATTAAGGCATATGGACCCTTTATCCACGGCCAAGATATCACTCCA

CCTGAACCTGAAAACGGTAAACCTATTTTCAGTGGCCTAAGCAATACTCGTGTTAAATTAGGCGGAACCT

TCAATGCTATGGACGGAGTAAGCGCCTATGATGAAGAAGATAAAGATCTGACCCAACAAATCCAAGTCAC

AGGCGACGTTAACACCAACCAGATTGGCCTCTATCCCCTGACATATAAAGTGACAGATTCCGACAATAAC

ACCACTGAACAAGTGCGTACGGTTGAAGTGTATAACACTAAACCGGAGCTATCTGGTGTTAGCAACACCA

CAATCATACTTGCAACCCCATTTGATGCTATGGCTGGCGTCAGCGCCACAGATGCTGAAGATGGCAACTT

AACCCCAAATATCCGTGTTGAAGGCGAAGTGGATAGCAATCGTGTGGGCCAATATCAACTGACCTATAGC

GTTAGCGATAGCGCAGGTGAACAAACCCGCGCAGTACGCCTTGTAACCGTGCAAGAACAAGGTGCTGTGT

GTGAAAATCTATGGAGCGCTAGCAAAATTTACCTAGGTGGCGACACAGTAAATTATGCTGGAAAAATTTG

GTTAGCGGGATGGTGGACTCAAGGTGAACAACCTGATTTGGCAGGCGAGTGGGGTGTTTGGAAGTCTAAG

GGCGACAGCGATTGTGACTCAGGCTTACCTCCGGTTATCACTCCTAACTTTACAGTGAGTGGCTTAGCCT

CAAGCTACATTCTCAATCAAGGTCAAGTTAGCATTAATTTTAACGTTAACACGAACCAAGCAATGGAGTT

AACCGCTAACCTCACTCAAGAGCAACGAATCCTTGCTAGCACGACTGCAACAATAAATGGCAACGGTGCC

ATGGTATTACAAGCGGAATCATTGGAAGCGGGTGCTTATCAAGTTGTGATCAGCGCTAAAGATAAGAATC

TTACGCAGTCCTATAACGTCTCGCTAGTTCAAGGTGATGATGGTAGTGAACCCGTTGAATACCCAGCCTA

TGTCGCAGGTAATCAATATCAAGCGGGGAATATTGTTACAAACGCCGGTGGTGTCTACCAATGCCGACCT

TGGCCAAACAGTGGTTGGTGTAGCGGCTCAGCGCCTTACTATGCGCCAGGTACAGGTAGCGCTTGGCAAG

ATGCGTGGACTAAGCATTAA

>NC_016901.1:c3034373-3030990 Shewanella baltica OS678, complete genome glycosyl hydrolase

ATGTTCTGTAATATACCTTGGGTAATGAAGCAACAGCGATTCGCGTGGTTAATGGTCACAGCTATGGCGG

GTTTCAGTTATTCTCAAACGGCTGCAGCAGTGAGTTGCCAAGACATAGCACCTTGGAACAGTAGCCAAAT

CTATGCAACCGTCACGCCTGTAACCTACGATAATTATCTCTATCAAAATAAGTGGTGGACCCAGAATGAA

AACCCAAGCCAAACAGGGCAGTGGGGCGTATGGGAAAACAAAGGCGTGTGTGATGGAGCGCAAAACCAAG

CGCCTACACTGGTCATCTTACAGCCGCAAAACAATGTGAGTGTGAATTTAGGGGATGTCGTCTTGCTGCA

GGCAGATGCATCTGATGTTGACGGTACCGTTGCGAGCGTAAATTGGTTTGCCAATGGTCAGGCGGTAACG

AGCCCTTGGACGACTAATGCGATAGGCAGTGTCCAGCTTAAAGCGGTAGCGACCGATGACAAAGGTGCGA

CGACCGAGAAAAGTGTTGTGCTAACCGTTATCAATCCTACGAGTGAAAACTTACCGCCTATGATTGAAAT

TTTGTTACTCGTCAATGACAGCGCTGTCAATGTTGGCGACTCAGTAACCATTACCGCGAATGCGAGCGAT

CCTGATACGGGTGATAGCATTACTAAAGTTGAGTTTTATCTTGATAGTCAATTGATTGCGACTGATAACA

GTGCACCCTATTCGGCAACGTGGCAGGCTGCAGGAGTCGGTGTGCATCAATTACAAACCCGTGCCTACGA

TAGTCACAATGCCAGCGGCTTGTCGGCAATCATCAAACTCAATGTTGCCGCAGCTAATCAAGCGCCGAGC

GTGAAATTAACCGCGCCGCAGTCCAACTTTCAAACAGATTTAGGCACGGTGTTATCACTCGCAGCAACTG

CGACTGACAGTGATGGCTATGTGGCCAGCGTGCGCTTTTATGCTAACGTTGTGCTCGTTGCTGAAGATGC

AACCGCGCCATACAGTGCACAATGGACAGCCATTGAAAACGGCGACATTCGTTTCACCGCTGAGGCGATT

GATAATCTTGGCCTGACAACATTATCAACGGCGGTTATTGGCCAAGTAGGTCAAGCACCAACCGATAATG

AGGCTTGCCGTCCTGAAGGTTTAGTGGGTGATTCAGTCTATTGCGATGTGTACGATGAACAGGGCCGCGA

AAAAATGGGTAGCGATCACGCCCGCCGCGTTATCGGTTACTTTACTTCGTGGCGCACGGGTAAAAATGGC

CAGCCGAGTTATCTCGCAAGTGATATTCCTTGGGATAAAATTACCCACATCAATTATGCATTTGCGCATA

TTGATTCGAATAACAAGGTCTCTATTGGCGACCCCAATAGTGCGACGAACGCGGCAACAGGATTAGAGTG

GCCGGGCGTTGCAGGCGCTGAGATGGATCCCGAGTTTAATTACAAAGGCCATTTTAACTTGCTCAATAAG

TATAAAAAACGCCATCCAAACGTTAAGACTTTAATCTCGATTGGTGGTTGGGCTGAAACGGGTGGTTATT

TTGATGATAACGGTAATCGTGTTGCCAGTGGTGGTTTCTATGAGATGACTCAGACGACGCAAGGGATTGA

AACCTTTGCCGATTCTGTGGTGACGTTCTTACGTACTTACAGCTTTGATGGGGCGGATATCGACTATGAG

TACGCGACATCAATGGCTAAATCCGGTAATCCTGATGATTTTTCGATGTCAGAGCCAAAGCGTGCGACCT

TGTTTAAACAGTACGAACTACTGATGAAAACCCTGCGTGAAAAACTCGATGCAGCCAGCAAGCAAGATGG

TAAGCACTATATGTTAACCGTTGCTGCGCCAGCATCGGGCTATTTGTTACGTGGGATGGAGGCGTATCAG

ATGACCCGTTATCTCGATTACGTCAACATTATGAGCTATGACTTGCACGGCGCGTGGAACGATTTTGTCG

GTCACAATGCGGCGCTATTTGACACTGGTACAGATGCAGAACTTGCGCATTGGGATGTTTATAGTACGGC

GCAATATGGCGGCATTGGTTATCTCAATACCGATTGGGCATACCATTATTTCCGTGGTTCTATGCCGGCA

GGGCGGATCAATATCGGTATTCCTTACTACACCCGTGGTTGGCAAAATGTAAGTGGTGGCACCAACGGTC

TATGGGGCTTAGCGGCATATCCGGATCAAACGGCTTGTCCTGTGGGTACGGGTGATGGGGCGTCAAACTG

TGGTTACGGTGCTCAAGGAATTGATAACCTCTGGCACGATAGTGATGTACGTGGCGATGAGATGTTCGCA

GGCTCTAACCCTATGTGGCATGCTAAAAACTTGGAACTCGGTATCAGCGGCAGTTATTTAAGCCTTTATG

GATTAGATCCGCAAACGAATGTCGCCGATCGACTGACGGGTACCTATGAGCGCCACTACGACGCTGTGGC

AGAATCTTCATGGTTATGGAACCCGACCAAAAAGGTGTATCTGTCTACTGAAGACGAGCAAGCTATGGCG

CGTAAAGTGCAATATGTGGTCGATAACGGCATTGGCGGCGTCATGTTCTGGGAATTAGCAGGTGACTTCG

GCTGGCATCCAGAGCGTAACAATGGTCAGGGTGAGTTCTTTGTCGGTGATACTATGACCAGCATTGCGTA

CAACGGATTTGCGCAAGCAACCCCGTATGGTAATCGCACCAGCAATCAGTTAATGCCATCACAAACATTA

GCCTTAACGGCAAGTTTATCTGGCTATAAAGTGGGCGACAGTAACTATCCGATTACGCCAACCTTAACCA

TCACCAATAATAGCCAAGTGACTATACCCGGCGGTGCTGAGTTTGAATTTGATATTTCAACTTCAACTTC

AGCCGACATTGCCGATCAATCGGGTATGGGGTTAACTGTGGTAACGGATGGTTCCAATGTGGCAGGAAAT

AACGTGGGCGGGTTGGAGAATGATTTCCACCATGTTCGCTTTATCTTGCCAACTTGGAAAAGTCTGGCAC

CTGGGGCAACGTTTGACGGCACTATTAAGTACTATTTGCCTGTCTCTATGCCATCAAACTTTACTGTGAG

TTTTAATGGCACTCGATATGGATTTGGTGCGGCGAGTTCCGTTGTTCCACCTGTGGATTGTGCTGCAAAT

CCAAGTTTACCTGAATGCCAAGTACCTGTGGTGGATAGTTGTGAAGCGGCGAGTGTCGATACTGCGGGTA

TTCCTGCTTATCCAAATTTCCCGCAAAAGGACTATCAGGGAAATCCAAGTCATGCAGCAACGGGCGATCG

GATGAAAAACAGTAGCGCTGTTTACCAAGCTAAGTGGTGGACCAACGCGGCACCTGGATCTTCTGCAGAT

TGGGCGTTTGTGTGTAATCTATAA

>NZ_HG326223.1:4538438-4540129 Serratia marcescens subsp. marcescens Db11, complete genome

ATGCGCAAATTTAATAAACCGCTGTTGGCGCTGTTGATCGGCAGTACGCTGTGTTCCGCGGCGCAGGCCG

CCGCGCCGGGCAAGCCGACCATCGCCTGGGGCAACACCAAGTTCGCCATTGTCGAAGTCGATCAGGCGGC

CACCGCTTATAATAATCTGGTGAAGGTAAAAAATGCCGCCGACGTTTCTGTCTCCTGGAATTTATGGAAT

GGCGACGCGGGCACGACGGCAAAAATTTTATTAAATGGCAAAGAGGCGTGGAGCGGCCCTTCAACCGGTT

CTTCCGGTACGGCGAATTTTAAAGTGAATAAAGGAGGCCGTTATCAAATGCAGGTGGCATTGTGCAATGC

CGACGGCTGCACCGCCAGTGACGCCACCGAAATTGTGGTGGCCGACACCGACGGCAGCCATTTGGCGCCG

TTGAAAGAGCCGCTGCTGGAAAAGAATAAACCGTATAAACAGAACTCCGGCAAAGTGGTCGGTTCTTATT

TCGTCGAGTGGGGCGTTTACGGGCGCAATTTCACCGTCGACAAGATCCCGGCGCAAAACCTGACCCACCT

GCTGTACGGCTTTATCCCGATCTGCGGCGGTAATGGCATCAACGACAGCCTGAAAGAGATCGAAGGCAGT

TTCCAGGCCTTGCAGCGCTCCTGTCAGGGCCGCGAGGACTTCAAAGTCTCGATCCACGATCCGTTCGCCG

CGCTGCAAAAAGCGCAGAAGGGCGTGACCGCCTGGGATGACCCCTACAAGGGCAACTTCGGCCAGCTGAT

GGCGCTGAAGCAGGCGCATCCTGACCTGAAAATTCTGCCGTCGATCGGCGGCTGGACGCTGTCCGACCCG

TTCTTCTTCATGGGCGACAAGGTGAAGCGCGATCGCTTCGTCGGTTCGGTAAAAGAGTTCCTGCAGACCT

GGAAGTTCTTCGACGGCGTGGATATCGACTGGGAGTTCCCGGGCGGCAAAGGCGCCAACCCTAACCTAGG

CAGCCCGCAAGACGGGGAAACCTATGTGCTGCTGATGAAGGAGCTGCGGGCGATGCTGGATCAGCTGTCG

GCGGAAACCGGCCGCAAGTATGAGCTGACCTCCGCCATCAGCGCTGGCAAGGACAAGATCGACAAGGTGG

CTTACAACGTTGCGCAGAACTCGATGGATCAAATCTTCCTGATGAGCTACGACTTCTATGGCGCCTTCGA

TCTGAAGAACCTGGGGCATCAGACCGCGCTGAATGCGCCGGCCTGGAAACCGGATACCGCCTACACCACG

GTGAACGGCGTGAATGCGCTGCTGACGCAGGGCGTCAAGCCGGGCAAGATCGTCGTCGGCACCGCCATGT

ATGGCCGCGGCTGGACCGGGGTGAACGGCTACCAGAACAACATTCCGTTCACCGGTACCGCCACCGGGCC

GGTTAAAGGCACCTGGGAGAATGGCATCGTGGACTACCGCCAAATCGCCAGCCAGTTCATGAGCGGCGAG

TGGCAGTATACCTACGACGCCACGGCGGAAGCGCCTTACGTGTTCAAACCTTCCACCGGCGATCTGATCA

CCTTCGACGATGCCCGCTCGGTGCAGGCCAAAGGCAAGTACGTGTTGGATAAGCAGTTAGGTGGCCTGTT

CTCCTGGGAGATCGACGCGGACAATGGCGATATTCTTAACAGCATGAACGCCAGCCTGGGCAATAGCGCC

GGCGTTCAATAA

>NZ_CP013913.1:3959679-3960959 Serratia fonticola strain GS2, complete genome

ATGGCTTTAACCCGTAAACTGCTACCCTTGCTGGTAGCGGTGCAACTCGGTGTGGCTGGCGTGGGCATGG

CTCACGCGGCACCTTATCTCTCTGTCGGCTACTTCAACGGCGGTGGCGACGTAACCGCCGGGCCGGGCGG

TGATATCAACCAGCTTGACGTCAGCCAGATCACCCACCTCAACTACTCTTTTGGCCTGATTTATAACGCT

GAAAAAGAGGAAACCAACCCGGCACTGAAAGATCCCTCCCGCCTACACCAAATCTATCTCTCCCCTAAAG

TCGAAGCAGACTTAAAGCTATTACCCGTATTGCGCCAGCAAAATCCGGCGCTGAAGGTTTTGCTGTCCGT

TGGCGGATGGGGAGCTCGTGGGTTCTCCGGCGCGGCAGCCACACCGGAAAGCCGGGCGGTGTTTATTCGT

TCGGTGCAGGAGGTGATTGCCAAGTATCAGCTAGATGGTATCGATCTGGATTGGGAATACCCGGTTAACG

GTGCCTGGGGATTGGTGGAAAGCCAGCCCACTGACAGAGCCAATTTCACCGCCCTACTGAGCGAACTGCA

TCAGGCGTTGGGTAAAGAAAAACTGCTGACCATCGCCGTCGGGGCTAACGTCAAAAGTCCGCAGGAATGG

GTAGACGTTAAGGCTATCGCGCCCTATCTGAACTACATCAATCTGATGACCTACGACATGGCGTACGGTA

CCCAGTATTTCAATTCCAATCTCTATGACTCCAAACAATGGCCAACCGTGGCCGCCGCCGACAAGTACAG

CGCCGACTTTGTGGTCAACAACTATTTGGCCGCCGGGCTGAAGCCAGCTCAGCTCAATCTGGGGATCGGT

TTCTATGGCCGGGTACCTAAACGCGCCACCGAGCCGGGTATTGATTGGGATGTGGCAGATGCGGCCAAGC

ATCCCGTCACCCAGCCCTATTTCACCACACGTGAAAAAGACGTCTTCAAGTCACTGGGCGTGGATTTGGA

TAAAGACAGTTACATCAAGTACAACGATATTGTGAACAAGATGCTGAAAGACCCACAACGGCGCTTCACC

GCGCATTGGGATAGCGAGGCCAAGGTGCCCTATCTGATGATGAAGTCATCCGCAGGCAAACCACTGTTCG

CGATAAGCTATGAAAACCCGCGCTCGGTAGCCATCAAGGCCGAGTACATCAAGAGCAAAGGATTGGGAGG

GGCGATGTTCTGGGAGTATGGCGCGGACGATAACAACCGCCTGGCCCACCAGTTAGCCGAAAGCTTAGGC

TTGAGCCCGCAGAAGCAGTAA

>fig|100226.1.peg.5328 Chitinase (EC 3.2.1.14) [Streptomyces coelicolor A3(2)]

atgcgcttcagacacaaagccgcggcactcgcagcgaccctggcgcttcccctcgccggt

ctggtcggcctcgcgagcccggcccaggcggccacgagcgcgacggccaccttcgccaag

acctcggactggggcacgggcttcggcggcagctggacggtgaagaacaccggcaccacc

tccctcagctcctggaccgtcgagtgggacttccccaccggcaccaaggtcacctccgcc

tgggacgccaccgtcaccaactccggcgaccactggaccgccaagaacgtcggctggaac

gggacgctcgcccccggcgcctcggtctccttcggcttcaacggcagcgggcccggctcc

ccgtccaactgcaagctcaacggcggcagctgcgacggcacctcggtccccggcgacgcg

gccccctccgcgcccggcacgcccaccgcctccaacatcaccgacacctcggtgaagctg

tcctggtcggcggcgaccgacgacaagggcgtcaagaactacgacgtcctgcgggacggc

gcgaaggtcgccaccgtcaccggcaccacctacacggacaacggcctcaccaagggcacc

gcctactcgtacagcgtcaaggcccgtgacaccgccgaccagaccggcccggccagcggc

gcggtgaaggtcaccaccaccggcggcggggacggcggcaaccccggcaccggtgccgag

gtcaagatgggctacttcaccaactggggcgtctacgggcgcaactaccacgtgaagaac

ctggtcacctccggctccgccgacaagatcacgcacatcaactacgccttcggcaacgtc

cagggcggcaagtgcaccatcggcgactcctacgccgactacgacaaggcctacaccgcc

gaccagtccgtcgacggcgtcgccgacacctgggaccagccgctgcgcggcaacttcaac

cagctgcgcaagctgaaggccaagtacccgaacatcaagatcctctactccttcggcggc

tggacctggtccggcggcttccccgacgccgtgaagaacccggccgcgttcgcgaagtcc

tgccacgacctggtcgaggacccgcgctgggccgacgtcttcgacggcatcgacctggac

tgggagtacccgaacgcctgcggtctcagctgtgacgagaccagcgccccgaacgccttc

agcagcatgatgaaggccatgcgcgccgagttcggccaggactacctgatcaccgcggcc

gtcaccgccgacggctcggacggcggcaagatcgacgccgccgactacggcgaggcctcg

aagtacatcgactggtacaacgtgatgacgtacgacttcttcggcgcctgggcgaagaac

ggcccgaccgccccgcactcgccgctgaccgcgtacgacggcatcccgcagcagggcttc

aacaccgccgacgcgatggcgaagttcaagtccaagggcgtcccggccgacaagctgctg

atcggcatcggcttctacggccgcggctggaccggcgtcacccagtccgcgcccggcggc

accgccaccggcccggcgaccggcacctacgaggcgggcatcgaggactacaaggtcctc

aagaacagctgcccggccacgggcaccatcgccggcaccgcgtacgcccactgcggctcc

aactggtggtcctacgacaccccggccaccatcaagtccaagatggactgggcggagcag

cagggcctcggcggcgccttcttctgggagttcagcggcgacaccacgaacggcgaactg

gtgagcgccatcgacagcggcctcaagtaa

>fig|479432.4.peg.4574 Chitinase III (EC 3.2.1.14) [Streptosporangium roseum DSM 43021]

gtgatgctcctccccctgggggcggtcgtcgcgacggcacccggcgcttccgcggccgtg

accgtgccgctccgggccaacgcgggcgggtccgcgctcaccgacgccgcgggtgaccag

tgggtcgccgacaaggcctactccagcggcgactggggctaccagaccagctacgggagc

ggctcgaccggcggtgcgatcgccgggacgaccgacgactccctctaccagaactacaac

accttcaacagctggggcggctaccgcttcgacgtcccgaacggcacctaccaggtgacg

ctgaagatggtcgaggactgggccaacgcggccggtcagcgcaagttcgacgtccgcgtc

gaaggcgtcaacgcgctgaccgccttcgacatcttcgcctcctgcggcgcgctcaccgcc

tgtgaccggaccttccccgcgacggtcagcgacggcgcgctcaacgtccagttcaacatg

aacggcggcgccaactacgcgaccgtctccgccatctcggtgaccggcggcggcggagga

ggcgacaccacgccccccagcgtccccggcaacctgcgctccaccgccacctcctcctcc

agcgtctccctcgcctggaacgcctccaccgacaacgtcggcgtcaccggctacgagatc

taccgaggcaccaccctcgtcaccaccgtcaccggaaccacccacaccgacaccggcctg

acccccgccaccgcctacagctacaccgtccgcgcccgcgacgccgccggaaaccgctcc

accgccagcaaccaactcaacgtcaccacccaggacggcggcggagggggcggcaacaag

ctgctgggctacttcacccagtggggcatctaccagcgggcctaccacgtcaagaacatc

gtcaccagcggctcggccgccaagctcacccatatcaactacgccttcggcaacgtgcag

aacggccagtgcacgctgggcgacacctacgccgactacgaccgtttctaccaggccggc

gagagcgtggacggcgtcgccgacacctgggacaacggcgcgctccgcggcaacttcaac

cagctccgcaagctgaagaagcagttcccgaacatcaaggtgctgttctccttcggcggc

tggacctggtcgggcggcttcggccaggcggcggccaaccccgcggccttcgccgagtcc

tgctaccgcctggtcgaggacccgcgctgggcggatgtgttcgacggcatcgacatcgac

tgggagtacccgaacgcctgcggcctcacctgtgacaccagcggcttctcctccttcaag

aacctgatgtcggcactgcgctcccgcttcggctccagctacctggtcaccgccgccatc

accgccgacgccacctccggcggcaagatcgacgcggccgactacggcggcgccgcacag

tacctcgactggtacaacgtcatgacctacgacttcttcggtgcctgggcagcccagggc

ccgaccgcgccgcactcaccgctgaacacctacagcggcatccccatcaccggtttcgac

tccgactccgccatccagaagctcaagagcaagggggtgccggcgagcaagctgctgctg

ggcatcggcttctacggccgcggctggaccggcgtgacccaggcgactccgggtggcacc

gccaccggcgccgccccgggcacctatgaggcgggcaacgaggactacaaggtcctcaag

acccgctgcccggcgaccggtacggtcgcgggcacggcctacgcccactgcggcaaccag

tggtggagctacgacacccccgcgaccatcgggggcaagatgagctactcgaagaaccag

ggcctgggaggggccttcttctgggagctcaccggagacacctccaacggtgagctgatc

agcgcgatgaagagcggcctgggctag

>NZ_JHZM01000021.1:c11058-10201 Brochothrix thermosphacta DSM 20171 = FSL F6-1036 strain DSM 20171 Q329DRAFT_scaffold00018.18_C, whole genome shotgun sequence

ATGATCACCACTACCAGTCAGCAGGCAGAAGCTTCTAGCAAAGTCGTTCCAAAAACAGTAATGTATGTAG

AAGTGAATGATCATGAGTTTCGAAATGTTGGAAAATACACACTAGCAAATTCAAAAAAAACGGCGATTGA

TATCGGAATCATTTTCGCAGCTAATATTAATTACGATAAAAATACTAAAAAACCTTATTTATTTTTAAAT

GAACAAGTCAAAAAAACTTTGAATGAAAACGCAACGCAAATCAAACCGATACAAGCACGTGGAACAAAAG

TACTGCTGTCCATTTTAGGTAATCATCAAGGTGCTGGTTTTGCCAACTTCGCTTCTTATGAGAGTGCAGA

TGCTTTTGCCGCGGAACTTGAAAAAGTTGTCAATGCTTATGGTTTAGATGGCATTGATTTTGATGATGAA

TACGCTAAATATGGGGAGAATGGCACACTGCAACCAAATAACTCGTCTTTTATTTGGCTCGTTCAAGCAT

TACGTCAACGACTTGGCGATGATAAATTAATTACACTTTACAACATTGGCCCCGCAGCTTATAACTCTAA

AGCTAACGATAGCCTATCAAAAAGTATTGATTACTCATGGAATCCCTACTATGGCACATGGAGTGCACCA

TCATTCCCAGGAATCGATTCATCGCGTTTAAGTGCAGCTGCAGTAGAAGTAGGTGTCAATAAGGCACAAA

GTGTACAGTTTGCTAAACGCTCTATCAACGAGAACTATGGCCTATTCCTCATGTATAATCTAAGCGGAAA

AAATTCAGCTGATTTTATTTCAGGAATCACACAAGAACTTTATGGTGAAAAAACAATCTACACAGAAACT

GTTCCGTCTGTACGATAG

>NZ_JNYR01000054.1:58-981 Streptomyces rimosus subsp. rimosus strain NRRL ISP-5260 contig54.1, whole genome shotgun sequence

ATGTTCCATCTGATACGGCGCAGAGTGCGGACGGCCGCGCTCGCGCTCTCGGCCATCGCGGCCCTCACCT

TCGGCGCTGCCACCCCGACCGGCGCGGCAGCCGCCACCACCCCCGCTCCCAGGAAGCAGGGGCCGACCTC

AGTGGCCTACGTCGAGGTGAACAACCACAGCATACTCAACGTGGGCAAGTACACCCTCGCCAACGGCGGC

GGCAACGCCTTCGACGTCGCCGTGATCTTCGCAGCGAACATCAACTACAACACGGACACGAAGACGGCGT

ATCTGCACTTCAACGAGAACGTGCAGCGCGTCCTTGACAACGTTGCCACCGAGGTGCGGCCGCTGCAGCG

CAAGGGCATCAAGGTCATCCTCTCGGTGCTGGGCAACCACCAGGGCGCGGGCTTCGCCAACTTCCCGTCG

CAGCAGGCGGCTTCGGCGTTCGCGAAACAGCTGTCGGACGCCGTGGCCAAGTACGGCCTGGACGGCATCG

ACTTCGACGACGAGTACGCCAAGTACGGCACCAACGGCACCGGCCAGCCCAACAGCAGCTCGTTCGTGCA

CCTGGTGACGGCGCTGCGCGCCAACATGCCGGACAAGATCATCAGCCTCTACAACATCGGCCCGTCCGCC

TCCCGCCTCTCCTACGGCGGCGTCGATATCTCCTCCAAGTTCGACTACGCCTGGAACCCGTACTACGGAA

CCTGGCGCGTCCCCGGCATCGCCCTGCCCAAGACGAAGCTTTCCCCGGCGGCCGTCCAAATCGGCCGCAC

CTCGCAGCGCACAGCCGCCAGCCTCGCCCGCCGCACCGTCCGCGACGGATACGGCGTATACCTCACCTAC

AACCTCGACGGCACCGACCGCAGCGCCGATGTCTCCGCGTTCACCAGGGAGCTGTACGGCAGTGACGCCG

TATACACGCCATAA

>NZ_CP019458.1:c2586479-2585553 Streptomyces autolyticus strain CGMCC0516, complete genome

ATGTTCACTCTGGTACGGAGGAGAGTACGGACGGCCGCGTTAGCGCTCTCCGCGGTCGCGGCCCTCGCCT

TCGGCGGGACCGCCGCGACCGCCGCGACCGGCGCGGCAGCGGCCCCCGCCCCCGCGAAACAGGGGCCGAC

CTCGGTGGCGTACGTCGAGGTGAACAACAACAGCATGCGCAACGTCGGCAAGTACACCCTCGCCAACGGC

GGCGGCAATGTCTTCGACGTCGCCGTGATCTTCGCGGCGAACATCAACTACGACACGGGTACGAAGGCCG

CGTATCTGCACTTCAACGAGAACGTGCAGCGCGTCCTCGACAACGCCGCCACGGAGATACGGCCGTTGCA

GCAGAAGGGCATCAAGGTCGTCCTCTCGGTGCTCGGCAACCATGAGGGCGCGGGCTTCGCCAACTTCCCT

TCCCAGCAGGCGGCTTCGGCGTTCGCGAAGCAACTGTCGGACACCGTGGCCAAGTACGGCCTGGACGGCA

TCGACTTCGATGACGAGTACGCCGACTACGGCAACAACGGCACGGGCCAGCCCAACGACAGCTCGTTCGT

GCACCTGGTGACGGCGCTGCGCGCGAATATGCCGGACAAGATCATCAGCCTCTACAACATCGGCCCGGCC

GCGTCGCGCCTCTCCTACGGCGGCGTCGACATCTCGTCCAAGTTCGACTACGCCTGGAACCCGTACTACG

GCACCTGGCAGGTGCCCCGCGTGGCACTGCCCAAGTCGAAGCTGTCGCCGGCTGCCGTCGAGATCGGCCG

GACCTCGCAGAGCACGGCCGCCGACCTCGCCCGTCGCACCGTCAGCGAGGGGTACGGCGTCTATCTGACG

TACAACCTCGACGGCGCCGATCGCAGCGCCGATGTCTCCGCGTTCACCAGGGAGCTGTACGGCAGCGACG

CCGTCTACACGCCGTAA

>NZ_KQ948451.1:c215573-214656 Streptomyces olivochromogenes strain DSM 40451 PRJNA299225_s001, whole genome shotgun sequence

ATGTTCACTCCGGTGCGGAGCAGAATACGGACGGCCGCGCTCGCGCTCTCGGCCATTACGGCCCTCGCCT

TAGGCGCGACCGCCACGACCGGCGCGGCAGCGGCCCCCGCTCCCGCGAAACAGGGACCAACCTCGGTGGC

GTACGTCGAGGTGAACAACAACAGCATGCTGAACGTCGGCAAGTACACCCTCGCCAACGGCGGCGGCAAC

GTCTTCGACGTCGCAGTGATCTTCGCGGCGAACATCAACTACGACACGGGCACGAAGGCGGCGACCCTGT

ACTTCAACGAGAACGTGCAACGCGTCCTCGACAACGCCGCCACGCAGATACGGCCGTTGCAGCAGAAGGG

CATCAAGGTCGTCCTGTCGGTGCTCGGCAACCACCAGGGCGCGGGCTTCGCCAACTTCCCCTCCCAGCAG

GCGGCTTCGACGTTCGCGAAAAAATTGTCGGACACCGTGGCGAAGTACGGCCTCGACGGCATCGACTTCG

ACGACGAGTACGCCGACTACGGCAACAACGGCACCGGCCAGCCCAACGCCAGCTCGTTCGTGTACCTGGT

GTCGGCACTGCGCGCCAACATGCCGGACAAGATCATCAGCCTTTACAACATCGGCCCGGCGGCGTCGCGC

CTGTCCTACGGCGGCGTCGACGTCTCGTCCAAGTTCGACTTCGCCTGGAACCCGTACTACGGCACCTGGC

AGGTCCCCGGCATCGCCCTGCCCAAGTCGAAGCTGTCGCCGGCGGCCGTCCAGATCGGCGGAACCTCACA

AAGCACGGCCGCCAACCTTGCCCGCCGCACGGTCAGTGAGGGGTACGGCGTCTACCTGACGTACAACCTC

GACGGCGCCGATCGCAGCGCCGATGTCTCCGCGTTCACCAGGGAGCTGTACGGCAGTGACGCCCACTACA

CGCCGTAG

>NZ_AFBG01000021.1:60544-62922 Acidovorax radicis N35 contig00021, whole genome shotgun sequence

ATGCCAAACGACGCTGCAGCCAGACCACACTTCGCACCTCGGGTGCGTGCCATAGCAGCCGGTCTGCTCC

TGGCGAGCACCCAGGCGCTTGCGCTCGATTGCGCGCCCGGCCAGTGGCTGGCGGAATACTTCCCCAACAC

CAGCCTGTCCGGGGCCGCCGTGCTGACCCGGTGCGAAAAAGGCCCCATCGACTACTACTGGATGACCAAC

AGCCCCGACGCGCGCCTGCCGGTGGACGCGTTCTCAGCCCGCTGGACGGCAAGCCTGCCGTTCGCCGCTG

GAGCCGCCAAGTTCACCACCTTCACAGACGACGGAGTGCGCCTGTACGTCGATGGGAAACGGATCATCAA

CCACTGGGCTCGGCACAGCATCGCGATGGACTCCGCATCCCTGCGGCTGACGGAAGGCCATCACACCGTG

CGCATGGAGTATTTTGAGGCCGGGGGCGAAGGGCTGGCGCAGCTCTTCATTGAGGGGTTGACGCCTACCA

CAACGCCCCCGCCCCCGCCAGAACCCCCACGCATCGGCGCATTCACGGCCCAGCCAGAAGCCATTGATAT

GGGGGGCAGCAGCATCCTGACATGGACCACCGAAAACACCGTCGCATGCGCCGCCAGCGGCGGCTGGACC

GGCGAACAGGCCCCCAACGGCGGTGCCACCGTGCGGCCTGCCGCAACCACCACCTATGGTCTGAGTTGCC

GCAATGCGGCGGGTGCCACGGCCAGCGCATCCACCACCGTCACGGTGCGCAAGACGCCGCCACCGCCCGT

CACGATCAGCTTGACGGCCACGCCGCCCCGCATTACCGCAGGCCAGAGCGCGCGACTGGCCTGGCAGGCG

AGCAACGCAGCGGGCTGCGCAGCGTCGCGCGGATGGAGCGGCCCGCGCGCCACCGAAGGCAGCGAACTGG

TAGGCCCCACCACAAACACCGCCTACACCCTGACCTGTACCGACAGCGCCGGGAAGAGCACCACTGCCTC

CGCGCAGGTTGCGGTAGAGGCCAGTAACCCGCTTCCCCCGGTCAGCGGGGTAAGTGCGACAGTTTCCGCC

CGCGGGGTGGGCCTGGCTTGGGACCCGGTGGCCCGCAGCGGCAAATGGGTCAACGGCTACTACGCAGGTT

GGTTCTGGGAGACCTATCCGCCCGAAGCCGTGGACATGGGCACGATGACACACTTCGTTTTCGGACGCTA

CGCACCAGCGCTCGGGTCCCTCCCAGGCGGCCGGGCGGGTGATCTGCTCGAAGGAGCGGGCGGAGGGCAC

TCGCCAGAGGTGGAGAATGCGCTGATCGCCAAGGCCCATGCTGCGGGCGTCAAGGCGGTGATGATGGTAG

GGGGCGATAGTGACGGCCCCGGATTTCTGGCCGCCACCGCCGACCCCACGGTTCGTGCACGTTTCATCCA

GAACATTCTGGACAAGGCCGTACAGAAAAACTACGACGGCGTGGACATCGACTGGGAAGAAAACCTGGAC

ACGGCCCCCGCACGCGCCCAGGCCCTGGCACTTCTGCGTGAATTGCGCGTCGCATCCGCGCAAAGGGCCC

GATACCAGGCACCCAACGCGCCGCTGGAACTGAGCTGGCCTGGGTTCTGGGTCAACCAGAACCAGTCCGA

CATCACGCCCTGGCATGTCGAAGTGGCCGCCGCAGTCGATCGCTTCAATTTGATGACTTACAGCATGGCG

GGCGACTGGGGCTGGCTGTCATGGCACCACTCCCCTTTGTTCGGAGCAGGCGAACTGTATCCAACGTCGA

TCCAATCGACAGTCCAGGCCTACCTTGACGCAGGCGTGCCACGCTCCAAGCTGGGCATCGGCATCGGTTT

GTATGGCGCCTACTACAACAAGCCTGTCACCGGACCGCGCCAGAGCTTGAATGGCATGCAAGGCTGGATG

AACAATGGCGATTGGGAAAACAACTACCAGCGCCTGGTTCAAGACAACGCCTTCGGCCGGACGGGCGCCA

CCTACCATTGGGATGACGTGGCAAAACAGGGCTATATCACCTACTCCCCGCCATGGAATCGCGGCCCCAA

CACGCCAGTGACCTACCTGAGCTTCGAAGACGAGCGCTCCATCGCTGCCAAAGGCCAGTGGGTGCGTGAG

CAAGGCTTGGGCGGCACCCTGGTGTGGACCATCAACTACGGGTATTTGCCGCAATCCAGCAGCAATCCGC

AGATGCAGGCGGTCAAGCAGGCGTTCATCGCGTCCGGGGCTGCAGGCTATCGGGTCTACAGAGACGGCGT

GCTGATCGCCACCGTCACCGGAATCACCTTCACAGACACCGCCGCTCCGCGTGGTCATCGGGGTTACCAG

GTCAGCATGCTGGACGCGGCAGGCAACGAAGGCCCGCTGTCTGCGCCAGTGTTTGTGCAGGTCCCCTGA

>NC_010943.1:c704249-702150 Stenotrophomonas maltophilia K279a complete genome, strain K279a

ATGTACGACCCGATTGTGCGCAGTGCCGAACGTGCGACCCGAAGCTGTCGTCCCCGCCGCCTGGCCTGGC

TGCTCGCCGTGGCCGCCGGTGCCGCTGCCCTGCCCGGCCTGGCGCAGGCCGCCAGCTGTGCCGGCGTGGC

CCAGTGGGACCAGGCCAAGATCTACCGGGCCGGCGATACCCTGCAGAAAGGAGGCGTCCTCTATCGGGCG

AACCAGGACATCTGGAACGCGCCGCCGGACCATCCGGCCGGTGCGCCCTATTACACCAACCTCGGTGCCT

GCGATGGCAGCGGCGCCAACCAGCCGCCGGTGGTCAGCCTGACCTCGCCGGCCAACGGCGCCACCTTCAG

CGCCGGCAGCACGGTCAACGTGACCGCTACCGCCAGTGATCCGGACGGCAGCGTCGCCAAGGTCGAGTTC

TTCCGCGATGGCAGCACGCTGGGCGTGGCCACCAGTGCGCCGTATGCGGCCAGCTGGACCAATGCCAGCG

CCGGCAGCCACACCCTGCGCGCCGTAGCCACCGACAACAACAACGCCACCAGCAGCACCGCGACCATCAC

CATCACCGTCAACGCCGCCGGTGGCGATACCACCGCACCGAGCGTGCCGGGCGGACTGGCGGTCGGCACA

CGTACCGCCAACAGCATCGCGCTCAGCTGGAGCCCGTCCACCGACAACACCGGTGGCAGCGGCGTGGCCG

GCTATGACGTGTACCGCAATGGCAGCCTGGTCGGTTCACCATCCAGTGCCAGCTACGTCGATGGCGGCCT

GACTGCATCGACCACCTACCGCTACCGTGTGCGTGCCCGCGACAACGCCGGCAACGCGTCCGCGCAGGGC

ACCGAGATCAGCGCCACCACGCTGGCCGGCGACGGCGGCACTACCGGCAAGCGGGTCATCGGCTACTTCA

CCCAGTGGGGCATCTACGGCCGCAACTACCGGGTCAAGAACATCGACAGCAGCGGTTCGGCCGCGCGCCT

GACCCACATCAACTACGCCTTCGGCAACGTGCGCAACAACCGCTGCGAAGTGGGCGTCACCCAGCCGTCG

GATCCGAACAGCGGTGCCGGTGGTGATGCCTTCGCCGACTACACCAAGGCCTTCAGTGCCGCCGAGAGTG

TCAGCGGCAGCGCCGATACCTGGGACCAGCCGCTGCGCGGCAACTGGAACCAGCTCAAGCAGCTCAAGGC

CAAGTACCCGAGCATGAAGGTGCTGATCTCGCTGGGTGGCTGGACCTGGTCGCGTGGCTTCTCCAGCGCG

GCGCGCCCGGAAAACCGCCAGGCCTTCGTCGCCTCGTGCATCGATGCCTACATCAAGGGCAACCTGCCGG

TAACCGACGGTGCCGGTGGTGCCGGTGCCGCGCTGGGCGTGTTCGACGGCATCGACATCGACTGGGAGTA

CCCGGTGGCGTGCGGCATCGAATGCGGCAAGCCGGAAGACAACGCCAACTTCACCGCGCTGATGGCCGAG

TTCCGCCGCCAGCTCGATGCAGTGCGTCCGGGCCTGCTGCTGACGGTGGCGGTGGGTGCCGGCATCGACA

AGATCCGCGTCACCGACCCGGCCGCGTACCACCCGTACCTGGACTACATCAACGTGATGACCTACGACTT

CCACGGCGCGTGGGATGCGAAGACCAATCACCAGTCGGCGCTGTTTGATTCGCCGAATGATCCGTCCACC

GGCGACCAGAAGCTGTACAACAGCAACGACGCCATCGAGGCCTTCATCAGCCGTGGCGTGCCTGCCGCCA

AGCTCAACCTGGGCATCGGCTACTACGGGCGTGGCTGGACCGGTGTGGCCAACGCCAACAACGGCCTGTA

CCAGACCGCGACCGGCGCTGCCCCGGGCACGTATGAAGCCGGCATCGAGGACTGGAAGGTGCTGAAGAAC

CTGGCGTGGCCGGGCTACACCGACAACACCGCCGGTGCCACCTGGATCTACAACGGCAGCACGCTGTGGA

GCTTCGACACCCCGGCCAACATCACCCGCAAGATGGGTTACGTGAAGACCCAGGGCCTGGGCGGCGCGTT

CGTCTGGGAGTTCAGTGGTGACGATGCGCAGGGCACGCTGACCAAGGCGGTCAGTGATGGCCTGAAGTAA

>NC_005140.1:816901-820083 Vibrio vulnificus YJ016 DNA, chromosome II, complete sequence

ATGCGTTTTAAAAACGGAAAGGCAGCAAAGAATGTGTTTACACTCAGTACTTTGACTGCTTCATGTCTGA

TGGCATTCAATAGCTACGCGGCTGTTGATTGTTCACCTCTACAAGAATGGAACTCTTCCGCCATTTATAA

CGGCGGAGACAAGGTTCAGTATCAGGGTAGCGCTTATGAAGCCCGTTACTGGACACAAAACAATAACCCA

GCAGAATTCTCCGGTGATTGGGCACAATGGAAAGCTCTCGGTGCTTGTGACAATGGCCCGGTTGACCCAG

TTAACCAAGTTCCTACCACCGCTTTGACCTCACCTGTTGCAACGGATGTTGTGAAAGAAGGTGAGGTCGT

TGTTCTTGCAGCAACGGCAGCGGATCAAGATGGCACCGTCGCCAAAGTTGAGTTTTTGGTTGATGGCGTT

GTGGTTGGCCAAGATACTTCGGCACCATTCTCCGCGTCATGGACTGCAACCGCAGGTGCTCATACTTTCA

GCTCTGTCGCTTATGACGATAAAGGCGCGGTAAGCCAACCAAGCAACGTGACGTTGACAGTGGAATCTAC

TCAGCCTGGCAATACGGCGCCAACGGTCGATCTTGCACTTTCTGCAACCACGGTTGAGCAGGGTGCAGTT

GTCACGTTGACCTCAAATGCAGCAGACAGCGATGGTACAGTTGAAAAAGTAGACTTTTTTGTTGGTGGCG

TGCTCGTCGGTACGGCAGCAACCGCTCCTTATACGCTTGACTATACCACCACTCAAGCTGGCTCTTTGTC

GGTCTTTGCTCGTGCGACGGATAACCTTGGTGCAACCACGGATTCCTCTGCACAGACGCTGAAAGTCAAC

GGTGTGGCACCAGTGGCATCGTGTCGCCCTGACGGCCTGTACCAAACAGAAGGCGTTAATGTGCCTTACT

GTACGATTTACGATGAAGAAGGTCGTGAGAAGATGGGGGCAGACCACCCACGTCGTGTGATCGGTTACTT

TACCAGCTGGCGTAGCGGTGATGACCCACAAGCGGCTTATCTGGTTAAAGATATTCCGTGGGAGCAGTTA

ACGCACATTAACTACGCGTTTGTCAGCATTGGCTCTGACGGCAAAGTCAACGTTGGTGACGTGAACGATC

CGACTAACGCTGCAACAGGAAAAGAGTGGCCGGGCGTTGAAATCGATCCGACTCTGGGCTTCAAAGGTCA

CTTTGGTGCGCTAGCCACTTACAAAGAAAAATATGGCGTTAAGACGCTGATTTCTATCGGTGGTTGGGCG

GAAACGGGCGGTCACTTTGGTGCGGACGGCAAACGTGTCGCAGACGGTGGTTTCTACACCATGACAACCA

ATGCTGATGGTTCCATCAACCATGCGGGCATTGAGAAATTTGCTACCTCAGCGGTCGAAATGATCCGTAA

GTACAAGTTCGACGGTGTGGACATCGATTACGAGTACCCGACTTCAATGGCGGGGGCGGGTAACCCAGAT

GATAAAGCCTTCATGGAACCTCGTCGTGCTTATCTATGGGCTTCATACCAAGAGTTAATGCGCGTATTGC

GTGAAAAGCTTGACCAAGCGTCTGCACAAGATGGCGTTCATTACATGCTCACCATCGCTGCACCATCTTC

GGGCTACCTACTGCGTGGTATGGAAACGTTTGATGTGACTAAGTACCTCGATTACGTCAACATCATGTCT

TACGACCTACACGGTGCATGGAACGATCATGTTGGTCATAACGCGGCGCTGTACGATACAGGTGAAGACT

CAGAATTGGCGCAGTGGAATGTTTACGGCACGGCACAATATGGTGGCATCGGTTACCTCAATACAGACTG

GGCATACCACTACTTCCGTGGCTCAATGCCAGCAGGTCGTATTAACATCGGTGTGCCTTACTATACTCGT

GGTTGGCAAGGTGTGACGGGTGGTACCAATGGCCTATGGGGCCGAGCGGCGCTACCAAACCAGGCTGAGT

GTGCTCCGGGTACAGGTGAAGGTGAGAAGAATAACTGTGGCCATGGTGCGATGGGTATCGACAACATGTG

GCACGACACCGATCCAAAAGGTAACGAAATGGGCGCGGGTTCAAACCCAATGTGGCATGCGAAGAACCTA

GAAAAAGGCATCTTTGGCTCCTATGCGTCAGCGTACAAACTTGATCCAGTCAACGATCCGCAAGATAAAC

TGGTTGGTAGCTATGTTCGCAACTATGACAGCGTAGCAGTGGCGCCTTGGTTGTGGAATGCAGAAAAAGC

GGTATTCCTATCAACGGAAGACAAAGCGTCTGTCAGCGTGAAAGCCGACTACGTCATCGATAAAGAGATC

GGCGGTATCATGTTCTGGGAATTGGCGGGTGACTATAACTGTTACGTGCTTGATGCCAATGGCAACCGCA

CGACCATCGATGAAACCGAGCAGGCATGTGCCACGGGCAACGGTGAGTTCCACATGGGTAACTCGATGAC

CAAAGCTATCTACGATAAGTTTAAGTCAGCAACCCCATACGGCAACACGGTCGCAACGGGCGCAATTCCT

TCAGAGACACTGGACATCGCTGTTTCTGTCGGTGGATTCAAAGTGGGTGATCAAAACTACCCAATCAACC

CGAAAGTGACCTTTACCAACAATACCGGTACAGCAATTCCGGGCGGCACTGAGTTCCAGTTCGACATTCC

AGTGTCGGCGCCAGATAACGCGAAAGATCAATCTGGTGGTGGCCTGAAAGTGATTGCTTCTGGTCACACA

CGTGCGAACAACATCGGTGGTCTGGATGGCGTAATGCATCGTGTGGCGTTCTCGCTTCCTGCGTGGAAAG

AACTACCGGCAGGCGGAACTTACGAGTTGGATATGGTTTACTACCTCCCAATTTCTGGTCCGGCAAACTA

CAGCGTGAACATCAACGGTGTGGACTACGCGTTCAAGTTTGAACAACCTGATCTGCCAGTTGCTGATTTG

TCTGCTGGCAACGGTGGCAACACGGGTGGTGGTGATACTGGCGGCGGTAACACCGGAGGTGGTGACACTG

GTGGCAATACAGGTAGCGGTACGGTTATCCAATGGGAACCAGGTGTAACGCAAGTGAATAATGGCGAAAC

AGTCACCTTTAACGGTAAGTGTTTTGTTGCGAAAAACAGCCCAGGGGTTTGGGAGTCTCCAACACAAACC

AACTGGTTCTGGGATGAAGTAACCTGTCCATAA

>NZ_LDRB01000008.1:c13127-12225 Curtobacterium oceanosedimentum strain NS263 contig_8, whole genome shotgun sequence

ATGCAGAAGAAGACGCAGATCGGCATCGTGGCGGCGCTCGCCGCGGTGCTCGCAGCCCCGATGATCCCGG

CGGCAGCATCCGCCGCACCGTCCACCGCGTCCGCGACGAAGTCCGGCCCGACGAGCATCGCCTACGTCGA

GGTCAACAACGACCAGCTCGCGAACGTCGGCCACTACCGGCTCGCCAACGGTGCGAACGCCTTCGACGTC

GCGATCATCTTCGCCGCGAACATCAACCGCGACGCGGACGGCAACGCAGTGCTGTACGCCAACGAGAACG

TGCAGCGCACGCTCGACGACGCCGCCACGCAGATCCGGCCGCTGCAGGCGAAGGGCATCAAGGTGTCGCT

GTCGATCCTCGGCAACCACCAGGGAACCGGCATCGCGAACTTCCCGACGCAGGCCGCGGCCGAGGACTTC

GCCGCACAGGTCTCCGCGACCGTCGCGCGGTACGGCCTCGACGGGGTCGACCTCGACGACGAGTACTCCG

ACTACGGCACGAACGGCACCCCGCAGCCGAACCAGCAGTCGATCGGCTGGCTCGTCAGCGCCCTGCGCGC

GGACATGCCCGGCAAGCTCATCTCGTTCTACGACATCGGCCCGGCGTCCGACGCCCTGTCGTCGTCGAGC

AGCACGATCGGGTCGCAGCTCGACTACGCCTGGAACCCGTACTACGGCACGTACTCGGCGCCCTCGATCC

CGGGGCTCGGCAAGGACCGGCTCTCGGCGGCGGCGGTCGACATCCAGAACACCCCGCGGTCGACGGCGGT

CTCGCTCGCACAGCGTTCGAAGGCCGACGGGTACGGCGTCTTCATGACGTACAACCTGCCGGACGGCGAC

GTCAGTCCGTACGTGTCCGGGTTCACGGACGTGCTGTACGGCCAGAGCGCGCACTACCAGTGA

>NZ_CP021151.1:c1226099-1222938 Photobacterium damselae subsp. damselae strain KC-Na-1 chromosome I, complete sequence

ATGGTTCACCAAAAGCAAGGAACGATAACCAAACGTATTTTCACCCTCAGCACCCTAACGGCATCGTGCT

TACTGGCATTTAATGCTCAGGCAGCCATAGATTGTACCAATCTTGCTGAATGGGAAGCGGGAAAAGTTTA

CGTTGGCGGTGATCAAGTCCAACAAAATAGTAAAGCTTACAAAGCAAACTATTGGACACAAAATAATAGC

CCAGATAAAAATACCGGTCCTTATGACGAATGGAATCTAATCGATAGCTGCGACACTGATACAACCCCTC

CTACAGATAATGTATTACCTTCTATCGATATTACCGCCCCAACCTCTAACCAAGTCTTTACTACGGGCGA

TATTGTTACCTTATCAGCCAACGCGACCGATAGTGATGGCTCCGTTACTAAAGTTGAATTCTTTATTAAT

GGACAATTACATGCAACATCAGCAACGGCTCCATACACATCTCAATGGACCGCAACTGCAGGTCAGCACA

CTCTAGTGGCAAAAGTTTTCGATGACCAAGGTGCAACAACTGAATCGCAACTCTCTTTTAGCGCTCAAGA

TACGGCGAATGTGCCACCAAGTGTTGATTTCACATTATCGGCAACTGAAGTAGATCAAGGGCAAGTTGTT

ACGCTTACTGCAACTGCCACCGACAGTGATGGTACCGTTGAAAAAGTCGATTTTTATGCTAACAACAAAT

TAATCGGAACTGCCGCTACTGAACCTTACACTTTAGATTACACAGCCAATACATCAGGTCAGCTTTCAAT

CACCACAAAAGCCATTGATAACCAAGGTGCGATAACCATTTCTACAGCCCAAACGCTCACGGTTATTGGC

GCGCCAATGGTTAAGAGCTGCCGCCCAGATGGACTCTATCAAACAGAAGGGATTAACGTTCCTTACTGTT

CTATTTATGATGAAGATGGCCGAGAAATGATGGGAGCCGATCACCCTCGACGTGTTATCGGTTATTTCAC

AAGTTGGCGTGCAGGTAATGACCCACAAAGTAGCTATCTTGTCAGTGATATTCCGTGGGAACAGCTTACC

CATATTAACTACGCTTTCGTCAGCATTGGCGCAGATGGTAAAGTGAATATTGGTGATGTCAACGACCCTA

AAAATGCGGCCGTTGGCATGGAGTGGGATGGTGTTGAAATCGACCCAGCTCTTGGCTTTAAAGGCCACTT

TGGAGCACTTGCTACCGCTAAGGTAAAACATGATGTTAAGACGCTCATCTCTATCGGTGGTTGGTCAGAA

ACTGGCGGACACTTTGATACTAATGGCGATCGTGTTGCTGATGGCGGTTTCTATACAATGACCACCAATG

CTGATGGCTCAATTAATCATGCGGGTATCGAGAAGTTTGCGGCATCAGCTGTTGAAATGATGCGTAAATA

CAAATTCGACGGTCTCGACATTGACTACGAATATCCGACCTCAATGGCAGGTGCTGGTAACCCATACGAT

AAAGAGTTCATGGAACCGCGCCGTCCATATCTATGGGCTTCGTACCAGGAATTAATGAAAGTTCTGCGTG

AGAAACTAGATGCGGCGTCAGCTCAAGATGGCATCCATTACATGCTCACTATCGCAGCACCATCTTCAGG

TTATCTATTACGCGGAATGGAAACATTCGATGTTACTCAGTACCTCGACTACGTTAACATCATGTCTTAC

GATCTCCATGGCGCATGGAATGATCACGTCGGTCACAACGCTGCTCTATTTGATACCGGAAAAGACTCAG

AACTGGCGGCATGGAATGTTTATGGCACGGCGGCATATGGCGGTATCGGCTACTTAAATACTGACTGGGC

TTACCATTATTTCCGCGGTTCAATGCCAGCAGGTCGAATTAATATCGGTGTTCCATACTATACTCGTGGC

TGGCAAGGTGTTACGGGCGGAGAAAATGGTCTTTGGGGTAAGGCACCACTACCAAATCAGTCTGAATGTG

CACCAGGTACAGGAGAAGGCGAGAAAAATAACTGCGGTTATGGTGCCATCGGTATTGATAACATGTGGCA

TGACCTCGACCCTCAAGGAAAAGAGATGGGTGCAGGCTCTAACCCTATGTGGCATGCTAAAAACTTAGAG

CATGGCGTATTTGGCTCATATGCTGCGGCCTACGGGTTGGATCCAGCAAATGAGCCTGATGATAAATTGG

TTGGAACATACTCTCGTAACTACGACTCTGTTGCCGTTGCTCCTTGGTTATGGAACGCAGAGAAAAGCGT

ATTCCTATCAACCGAAGATAAAGCCTCTGTCAGTGTAAAAGCAGACTACATTATCGATAAAGAGATTGGC

GGCATCATGTTCTGGGAACTGGCTGGTGACTACAGCTGTTACTTACTTGATGACAATGGCAAGCGTACAG

CAACCATTGATGCAACAGAAAAAGCTTGTGCATCGGGTAACGGTGAATACCACATGGGTAATACCATGAC

GAAAGCCATCTATGAGAAGTTTAAGTCTGCCTCTCCTTATGGCAATACTGTTGCAACTGGCGCTATTCCA

ACTGAGGCAGCCGATATTGCAGTGTCCGTTGGTGGCTTTAAAGTAGGCGATCAGAACTATCCAATTAATC

CTAAGATCACATTTACTAATAATACTGGTGTTGATCTACCTGGAGGTACTGAGTTCCAATTTGATATCCC

TACTTCAGCACCTGATAACGCAAAAGATCAGTCTGGCGGTGGATTGAAAGTTATTGCTTCTGGCCATACA

AAAGCGAATAACATTGGTGGTCTTGAAGGTGCCATGCATCGCGTTGCCTTTACACTACCAAAATGGAAGT

CTCTACCTGCCGGTGAAAGCTATGAGCTGGATATGGTTTACTACTTACCAATTTCTGGACCAGCTAACTA

TGCGGTGATTGTTAATGGTAAAGAGTACGCCTTTAAGTTTGAACAACCAGATTTACCTCTTGGTGATCTA

ACCACTGGTGGTGGCGAAGGAGGCGGCGGTGATGGTGGTACTTGCGATACAGCAGGTGTTAACACTTATC

CCGATTGGCCTCAAGCAGATTGGGCTGGCAACCCATCCCATGCCAATACAGGTGATCAACTAATTTATAA

CGGTGTCATCTATAAAGCTAACTGGTGGACAAGTTCAGTTCCTGGTAGTGATGGCAGTTGGTCAAAAGTG

TGTAAGATTTAA

>NZ_JZSK01000005.1:c17454-14278 Photobacterium leiognathi strain ATCC 25521 CFSAN029437_contig0004, whole genome shotgun sequence

ATGGATAATGGTAAACATGGAACGATGAGGAAAGGGATATTCACTCTTAGTACTCTAACTATATCATGCT

TCTTTGCTCTCAATGCACAAGCTGCAGTTGATTGTACAGACCTTGCAGAATGGGAAAGTAATAAAGTTTA

TACTGGCGGCGACCAAGTTCAGCAAAATGGTGTTGCGTATAAAGCGAATTACTGGACTCAAAATAACGCA

CCGAAAGATTTTAGTGGTCAATATTCACAATGGGTTGAGTTAGGACTTTGTTCTGACACGGAGATCCCTG

TTGAAAATGTTGCACCATCAGCAAGCATCACAGCTCCTACCGCAACAAGCTCAATCACGACCGGTGAAAC

GGTTCTGATCAAAGCAAATGCAACAGACACTGATGGCACGGTTGCTAAAGTAGACTTCCTTGTTGACGGC

ACTGTTGTAGGTAGTTCAACAACAGCTCCTTATGAGTTTGCTTGGGTTGCTACAGAAGGCGCACATAGCC

TAGCAGCTGTTGCTTACGATGATAAGAATGCAGCAAGTAGCCAAGCAACTGTAAACGTTGATGTTGCACC

TGAAGGTCCTGTTAACCAAGCACCGACTGTTTCTGTTGCCGTTTCTGCAGCAACTGTAGAACTTGGTGAA

GTAGTAACGATTACTGCTGACGCAGCTGATGCTGACGGCACAGTAGAGAAAGTAGATTTCTATGTTAATG

GTGCCCTTGTTGGTACAGCAGCAACTGCGCCTTACGTACTTGAATACACAACGGCACAAGCGGGTTCTGC

AAAGATCTTTGCTCGTGCAACTGATGATAAGAACAAAACATCAGATTCTGCAGTAACTGCAGTTACCGTC

AATGGTGCTGTTGTTTCTAACGGTTGTCGTCCTGACGGTTTATACCAAACTGAAGGTGTAAACGTTCCTT

ACTGTTCTATCTACGACGAAGAAGGTCGTGAGAAAATGGGCGCGGATCACCCTCGTCGTGTGATCGGTTA

CTTTACTAGCTGGCGTGCGGGCGATGATCCACAAAGCAGCTACCTTGTTAACGATATTCCATGGGAACAA

CTAACGCACATTAACTACGCATTCGTTAGTATCGGTTCTGACGGTAAAGTAAACATCGGTGATGTTAACG

ATCCATCAAACGCTGCAGTCGGTATGGAGTGGGACGGTGTTGAAATTGATCCAGCACTGGGCTTTAAAGG

TCACTTCGGTGCCCTAGCAACAGCGAAAGCAAAACACGATGTTAAGACATTAATCTCTATCGGTGGTTGG

GCTGAAACTGGCGGTCACTTCGGTACAGATGGCAACCGTGTTGCTGACGGTGGTTTCTACACCATGACAA

CGAACTCAGATGGTTCTATCAACCATGCTGGTATCGAGAAATTCGCAGCATCAGCGGTAGAAATGATGCG

TAAGTACAAGTTCGATGGTCTAGATATCGACTACGAGTACCCGACTTCAATGGCAGGTGCGGGTAACCCA

TACGATAAAGACTTCATGGAACCACGTCGTCCATACCTATGGGCTTCATACCAAGAGCTAATGAAGGTTC

TACGTGAAAAACTGGATGTGGCTTCTGCTGAAGATAACATTCACTACATGTTAACCATCGCAGCACCATC

ATCAGGTTACCTACTACGTGGTATGGAAACATTTGATGTAACGAAATACCTTGATTACGTTAACATCATG

TCATACGACTTACACGGTGCATGGAATGATCACGTTGGTCACAACGCTGCTCTATTCGACACAGGTAAAG

ACTCTGAGCTTGCTCAGTGGAACGTATACGGTACAGCTGCTTACGGCGGTATTGGTTACCTAAATACTGA

CTGGGCTTACCACTACTTCCGTGGTTCTATGCCTGCAGGTCGTATTAACATTGGTGTTCCTTACTACACC

CGTGGTTGGCAAGGCGTTTCTGGTGGTGATAACGGTCTATGGGGTCGTGCAGCACTGCCTAACCAGTCAG

AGTGTCAAGCAGGTACAGGTGAAGGTGAGAAGAACAACTGTGGTTACGGTGCAACGGGTATCGACAACAT

GTGGCACGATCTTGACCCAGATGGTAAAGAAATGGGCGCAGGTTCTAACCCAATGTGGCACGCTAAGAAC

CTTGAGAACGGTGTCTTCGGCTCATACGCATCTGCTTACGGCCTAGATCCAGCAAATGATCCTGAAGATA

AACTAGTAGGTACATATACTCGTAACTACGATGATGTTGCTGTAGCACCATGGCTATGGAACGCAGAGAA

GAGCGTATTTATCTCAACGGAAGATAAAGCCTCTGTAAGCGTGAAAGCTGACTACGTAATCGATAAAGAA

ATCGGTGGTATCATGTTCTGGGAGCTTGCGGGCGACTACAACTGTTACCTACTAGATGCTAACGGTAAGC

GTACAAGCACTATTGATGCAACTGAAAAAGCGTGTGCTTCAGGTAATGGTGAGTACCACATGGGTAACTC

GATGACAAAAGCTATCTACGATAAGTTTAAGTCAGCAACGCCATACGGTAATACTGTTGCTACAGGCGCT

ATTCCAACACAAGCTGTCGATATTGCAGTGAAAGTTGATGGCTTTAAAGTGGGTGATCAAAACTACCCTA

TCAACCCAACAGTAACCTTTACTAACAACACAGGTGTTGACCTACCAGGTGGTACAGAGTTCCAGTTTGA

TATTCCAGTATCTGCTCCAGATAACGCGAAAGATCAATCAGGTGGTGGTCTGAAAGTGATTGCATCAGGT

CATACTCGTAGCGATAACATTGGTGGCCTAGACGGCACAATGCACCGTGTAGCCTTCTCACTACCTAAGT

GGAAGACACTACCTGCAGGTGAAAGTTACGAATTAGACTTCATTTACTACCTACCAATCTCAGGTCCAGC

TAACTACTCTGTTAACGTTGACGGAAATGAGTATGCATTTAAGTTTGAGCAGCCAAATCTACCAATTGGT

GATTTAAATGCAGGTGGCGGTAACGGTGGTGGCGACAATGGCGGTAATAACGGCGATTGTGATACGACAG

GTCTAAATACTTACCCTGAATGGCCACAAACAGACTGGCAAGGTACACCAACACACGCTGGTAGCGGTGA

CCAAATCATCCATGATGGTGCGGTATATAAAGCAAACTGGTGGACATCATCAGTACCAGGAAGTGATGGT

AGCTGGACGAAAGTTTGTAACATCTAA

>NZ_AHCF02000017.1:387224-389854 Pseudoalteromonas undina DSM 6065 strain NCIMB 2128 contig017, whole genome shotgun sequence

ATGAATATCAAACAACTAAGCGCAGCCATCGGTGTTGCTTTATTTGCAAGTACAGCATCTGCTGCACCGT

CTACACCAAGCATTAATTGGGAACCACAGCAATATTCGTTTGTTGAGGTAGACCTTGAGGGAAACGGCTC

TTATAAGCAGCTAGTTACCCGTGTTGAACAAGTTAATATCAACATTCAGTGGAGTGCATGGAGTGGTGAT

GGTGGCGATAGCTACAAAGTGTACTTTGATGACATGCTAGTGAATGAGGGCACGCTTGCTGCTGGCTCTA

AAAGTGGCACTATCACATTCCCTTATGATAAAGCGGGCCGCCATACAATGTATGTTGAGCTATGTGAAGG

TGGCACAACCTGTGCGCGTAGTGCAGGCAAACCAATTGTAATTGCTGACACCGACGGCGGACACCTAGCA

CCACTGCCGATGGATGTAGACCCAAATAACCGTGACATTGGTATCAAACAAGGTTTAGTTACAGGCGCTT

ACTTTGTTGAGTGGGGTATTTATGGCCGTGATTACGATGTAACAAATATGCCAGCACAAAATCTGAGCCA

TATTTTGTATGGCTTTATTCCAATTTGTGGTGAAAACGCATCGTTATCGGGTGGTCCTAAACGTGCACTT

GAAACCGCTTGTGCCGGCTCTGCAGATTACGAAGTGGTTATTCACGACCCATGGGCAGCAGTACAAAAAG

CACTGCCAGGGGTTGATGCAAAAGATCCTATCCGTGGTACTTACTCTCAGCTAATGGCGCTTAAACAGCG

TTACCCAGATATTAAAATTTTACCATCTGTAGGTGGTTGGACGTTATCAGACCCATTCGGTGGCTTTACC

AATAAAGCTAACCGCGACATCTTCGTTGCCTCAATGGAAGAATTTTTAAGAACATGGAAATTTTATGATG

GTGTAGATATTGACTGGGAATTCCCAGGTGGTGACGGCCCCAACCCAGACATTGGCGATCCAATCAACGA

TGGCCCAGCCTATGTGGCGCTAATGCAAGAGCTACGCGCTATGCTTGATAAACTAGAAGCAGAAACAGGT

CGTACTTACGAGTTAACCTCAGCCATTGGTGCAGGTTACGATAAAATTGAAGATGTAGACTACCAAGCAG

CTAGCCAGTATATGGATTACATTTTTGCCATGACCTATGACTTTTATGGTGCATGGAGTAACGTAACCGG

ACACCAAACAGCACTTTACTGTGGCGAACATATGAGTGTTGGTCAATGTAATGGTACCGGGCTTGATGAA

AATGGCGAACCTCGTAAAGGCCCTGCTTATACCACCGATAATGCAGTGCAGCTGTTACTTGCGCAAAATG

TACCATCGAAAAAAATCGTCGTGGGCACAGCTATGTATGGTCGTGGTTGGGAAGGTGTTTACCCGCAAAA

TGCAGCGATTGATGGTAACCCAATGACCGCCCCTGGTAATGGCCCGTTAAAGGGCTCTACGGCACAAGGC

GTATGGGAAGATGGGGTTATTGATTACAAGGGCGTTAAAGCCAATATGATTGGTGCTGCGGGCACCGGTA

TTAATGGTTTTGAAGTGGGTTATGATGAGCAAGCACAAGCCGCTTATGTATGGAATCGTTCAACCGGTAA

ACTAATTACTTATGATAGCCGTAAGTCAGTACTGGCGAAGGGAGCGTATGTTAATCAGTATAATTTAGGT

GGTTTATTTGCATGGGAAATAGATGCGGATAATGGTGATATTCTTAATGCTATGCATGATGGTTTAGGGG

GCGTAGTTGCTCCGCCAACAAATAAAAAGCCAGTTGTTTCTGTGTCTTCATCAGTGTCTGTTAATTCAGG

CGAGAGTATTACAGTAACAGCTTCTGCAACTGATGCAGATAACGATCCACTTAGCTTTAGCTGGAATGCT

GATAGCGCACTGGTGGTATCTGGACAAAATAGTGCATCATTAGTCATTACAGCGCCAACAGTGACTGCAG

ATACACAGTATGTGGCAACTGTTGCTGTATCAGATGGTCAAGCAACTGTTAATCGTGATGTTGTTGTTAA

TGTTATTGCGCCAACATCAGGAGGCGAAAACACAGCACCGAGCGTTGATGCTATCGCTAATATCAGCGTT

GAAGAGGGCGCATCAGCAAGTGTAGCTGTAGTGGCTAATGATGCTCAAAACGACGCCCTTTCGTATACGT

GGACAGTACCAGCAGGCTTAACATTAGTGGGCAGTGATGCAAATGTAACTATTGAGGCTGGCGCTGTAGA

TACCGATACAGACTTCACAGTGTCTGTTGCAGTCAGTGATGGCGCATTAACCACAACGCAAAGCTTTAGC

GTAACAGTTATAAACGTTGATACTACAAATCCACCTACAGGAAGCTGGGATGCGAGTGTTGCATACGTTG

GTGGTGATGTCGTTACTTATAACGGCGTTGAATATAAAGCAAAGTGGTGGACTCAAGGCGAGCGTCCTGA

TTTAGGTGGTGCATGGGAAGCAACAACACAACCTACAGATGGTACGGGTGTCGCAGTTTGGCAGCCAACG

GCTATTTATAACAGTGGTGATGAAGTTTCTTATCAAGGTAATAAGTACCAAGCTAAATGGTGGACTCAAG

GGAACGAACCTGGCTCAACCGATGTATGGTTAGCACTTTAA

>NZ_CP021646.1:2705086-2707638 Pseudoalteromonas piscicida strain DE2-B chromosome 1, complete sequence

ATGAAACCAACTTCTATTTTACGATTAGCTTGGACGAGCGGTGTTCTCATCCTTGCAGGCCAAGCAAACG

CCTACAACTGTAGTAATTTAAGCCCTTGGTCTGAACAACAAGTCTACAGACAGGGGGATATTACACAGTC

CCAAAACCAAGCATATGAAGCTAAGTGGTACAGTCAAAACGACAATCCAGCAGGCCACTCCGGTCCTTAT

GATGTCTGGCGGTCACTGGGAACTTGTGCAACGGGCTCAGGTCAAGCGCCGAGTGTCGTACTCACCAGCC

CAAATAATGGCGCTGTGCTGGGTGAAAATGACAGCGCTGTTTTTTCCGCTAATGCCAATGATGTCGATGG

TGACCTAGCTTATGTCGAATTCTTAGTAAATGGCCAAGTCATCGCAACGGACACTACCGCGCCATTCCAA

ACCACTTGGAGCGCAGTACTTGGCGAATACGATATCAGTGCAATTGCCACCGATGCCCAAGGGCTAACCG

GAAGTTCACAATCAGCACGGATCACAGTAAAACCGAGTGGGGGAAACTTACCACCGAGCGTATCCATTGT

AGCACCAGCCAACGGTCAGCAGTTCTATCAAGGCGATATGGTAGGGATCAGCATTACAGCTGAAGATAAC

GACGGTACTATTGCGGGAGTGGAAATTAGCGTGAACAACCAGCTTGTCGGCAGCCTCATGAATCCTCCCT

TTCAATTTCAATTCCAAGCAGAGCAAATTGGTGTAAACCAACTAAATGTTACAGCGATAGACAATCAAGG

CTTGATAAGTGAGGCAAACCTTGTCATCGACGTATTAACAAAAGTGGGTGGTGGCTGCCAAGGCCTCAGA

GCATATAGCGCAGGACAAAATTACGAGGCTGGTGAACTCGTCGCGCATAATAATCATAAATATCGCTGTG

ATGTCGCAGGTTGGTGTAGCTCAAACGCGCAGTGGGCATACGAGCCAGGTGCGGGCCAGTATTGGAGCGA

TGCATGGAGCGATTTAGGGATCTGCGCAATCGCGCCAACCGTAACGTTCGAAAGCCCGTCCAATAATGGT

ACTGTTTTGGTCAATGCGCCCACCACTATCTCTGTCAATGCTCAAGATACTGACGGCAGTATTGCTACGC

TGTCACTCTATGCCGACAGCACACTCCTCGCAACGGCCAATCAAGGAATGCTTAATCACAGCTGGACACC

GACAGCCCTCAAAACCGTGAGCCTAAAAGCCATTGCCGTCGATAACGAAAGTAATCAAAGCGAGCAAACG

ATCACAGTGAATGTGACTGATAAACCGATTGCCGTTGATTTACTTGCCCCCACTAGCGGCAGCCAAGTTA

CCTTGGGTAATTCCACTCAGTTGATTGCACAAGCATCCTCTTTTGTTGGACAAATCACACAAGTACAGTT

TTATGCTGATGGCGTGTTACTCAATACTGACACAACGCCACCCTACGAATATAGTTATGCACCAGCATCG

ATTGGTACAAAAACACTTTATGCCACAGCAACCAACGACAATAATGATCAGGTGTCTAGTAGCGGCGTGA

CAGTTCAAGTCATTGACAAGCCCATCGGTAAAAAGCACAAACTGATTGGCTATTGGCATAACTTTGTCAA

TCCAGCTGGTTGCCCTATTCCACTTGATCAGATGTCTAATGCTTGGGACATCATCGATATTGCCTTTGCT

GAAAATGATAGAAACTCGAATGGCACAGTGCACTTCACTCCATTTGAAAAAGACATTCGCTCTAACTGCC

CGCCTATCGACCCGGTAAAATTCAAATCCGACATGCAGGCACTACAAGCGCAAGGTAAAGTTTTTGTGCT

AAGCCTTGGTGGTGCAGAAGGCACAATTACCCTCAATACGGATGCCGATGAAGCGGCATTTGTCAGTAGC

CTCACCGATATTATTCAACAATGGGGATTTGACGGACTCGATATCGACCTAGAGAGTGGCTCGAACCTTG

TGCATGGCTCTCAGATCCAAGCTCGGCTGCCAAGAGCATTAAAACAAATAGAACAAAATATTGGTGGCAA

TATGGTACTTACCATGGCCCCTGAGCATCCCTATGTGCATGGTGGCATGATTGCCTATTCAGGTATTTGG

GGCGCTTATATTCCACTTATAAATGAGCTACGTGATACATTAGACTTACTGCACGTGCAGCTATACAACA

ATGGCGGCCTACCTAACCCATATGAACCGGGAAGTGCTCCTGAGGGCTCAGTGAACATGATGGTTGCCCA

TGCAAAAATGCTGATTGAGGGATTTGGTCTTGCTGATGGCACCCGCTTTGCGCCACTTAGGGATGACCAA

GTAGCCATTGGTCTACCTTCTGGTCCACAGTCGGCAAATTCAGGTCAAGCGCCTATCGGCAATATCATCA

ATGCCTTAGATTGTTTAACCAAAGGTACAGGATGTGGCACTATCCAACCCAGTAGGGCTTATCCGAACTT

TGGTGGCGTGATGGCATGGTCGATTAACTGGGACAAGTACGATGGCTATAACTTCTCTCAACCCATTGGC

GATAAATTAACGCAGATGAATCAAGGAAATTAA

>NZ_FOWR01000007.1:16994-20158 Enterovibrio norvegicus DSM 15893, whole genome shotgun sequence

ATGGATGTATCACGCAAGAGCAAAGAGCTCACGCTATCGGCAGTTGCCGTCGCGTGTATGTTTGCTTTCA

ACGCGCAGGCTGCAACCGACTGTACGAATATTGAAGTATGGAAAGCTGATAAAGCTTATGCTGGCGGTTC

AACTGTGCAGCAAAATGACAAGGTGTATAAATCGGGCTGGTGGAGCATGGGCAATAGCCCTGAAACGCAC

TCAGGCGATTACAAAGAATGGAAAAATATCGATGTCTGTGAAACCGACACGCCAGTCGATAACCTTGTCC

CAACCGTTTCTTTAGACTCGCCGACCGACGCTGACGTCGTGAAAGTCGATGACGTCGTCACACTGACCGC

CTCTGCTGCCGATGCTGATGGATCAGTCGCGAGTGTTGAGTTCTTTGTTGATGGCATGTCCGTTGGCAAA

GATACGTCTTCTCCTTACAGCGCTACATACACCCCAACCCAAGAGGGTACGGTCGCTATCGTTGCCAAAG

TGGTAGACAATGAAGGTGCTGTCGCTCAAACGGCCCCAGTATCTCTGGTTGTTGAAAAGGGCGTTATTGA

CCCTGGAAACGTGGCGCCAACCGTTTCATTGGCCGTCTCTTCTAGTGCTGTAGACCAAGGTGCAACTGTT

ACGCTAAGCGCCGATGCTGCTGACTCTGACGGTTCTGTAGCAAGCGTTGATTTTTACGCCGCTGGTCAGT

TGATTGGTTCTGCAAACGCTGCACCGTTTACTTTGGATTGGGTTGCTAACAAAGCGGGCACTATCGCACT

GTATGCAAAAGCAACAGATGACAAAGGCGCATCAACCGATTCAAGCACAGTTTCTGTCACAGTCACTGGT

ACACCAACCGTCTCTAACTGTCGCCCAGAAGGTCTTTACCAAACACCTGGCGTAAACGTGCCTTACTGTA

CGATTTACGATGCGGAAGGCCGTGAAGACATGGGTGCAGACCACCCTCGCCGTATCATCGGTTACTTCAC

AAGCTGGCGTTCAGGTGATGACCCGCAAGCCCAGTACCTTGTTGATGACATTCCTTGGGAACAACTCACA

CACATTAACTACGCCTTCGTGAGCATTGGCTCTGACGGTAAAGTGAACGTGGGTAACGTTAACGATCCAA

ACAACGCAGCAACGGGCAAAGAATGGCCAGGCGTTGAGGTTGATCCTGCATTAGGCTTCAAAGGTCACTT

CGGTGCACTTGCGACGTACAAGCAAAAACATGACGTTAAGACACTGATTTCTATCGGTGGCTGGGCGGAA

ACAGGTGGTCACTTTGCTGAAAACGGCGACCGTATCGCCGATGGTGGCTTCTACTCGATGACCACAAACT

CTGACGGTTCAATCAACCATGCGGGTATCGAGAAATTCGCGGCCTCTGCCGTTGAAATGATTCGTACCTA

CAAGTTTGACGGTATCGACATCGACTACGAATACCCAACCTCGATGGCAGGCGCGGGTAACCCAGATGAC

AAAGCAGTAATGGAACCGCTTCGTCCATACCTATGGGCGTCTTACCAAGAACTGATGCGCGTACTTCGAG

AGAAGATCGACATTGCGTCTGAACAAGACGGTATTCACTACATGCTGACCATCGCGTCACCATCTTCGGG

CTACTTACTCCGTGGTATGGAAACGTTCGATGTCACTAAGTACCTTGATTACGTCAACATCATGTCTTAC

GACTTACATGGTGCGTGGAATGACCACGTTGGTCATAACGCTGCGTTGTTCGATACAGGTAAAGACTCCG

AGCTAGCAGCATGGAACGTTTATGGCACTAGCCAATATGGCGGCATGGGCTACCTCAACACTGACTGGGC

TTACCATTACTTCCGTGGCTCTATGCCAGCAGGCCGTATCAACATTGGTGTTCCTTACTACACCCGCGGT

TGGCAAGGCGTAACAGGCGGTGAAAACGGTCTGTGGGGTAAAGCTGCACTGCCTAACCAAGCTGAATGTG

CTGCTGGTACAGGCGAAGGTGAGAAGAACAACTGTGGTAACGGTGCTATCGGTATCGATAACATGTGGCA

CGACAAGAACTCACTCGGCGAAGAGATGGGTGCAGGTTCTAACCCAATGTGGCACGCGAAGAACCTTGAA

AACGGTGTCTTCGGTTCGTATGCAACGGCTTATCAGCTAGATCCAGCGAACGACCCATCTGACGCACTGA

CAGGTTCTTATGCCCGCAACTACGACGCAGTGGCAGTCGCTCCTTGGTTGTGGAACGCAGAGAAGAAAGT

CTTCCTGTCTACAGAAGATGTCGACTCTGTTTCTGTTAAATCTGACTATATTGTCGATAAAGGCATCGGC

GGCATCATGTTCTGGGAGCTTGCGGGCGACTACAACTGTTATGTGTTTGATGCAAACGGCAACCGTTCAA

CCACCATTGATAAAACCGAAGTGGCTTGTCAAACCGGTAACGGTGAGTTCCACATGGGTAACAGCATGAC

CAACGCGATCTACGAGAAGTTCGCCGCGGCAGCGCCATATGGTAACAAGCTATCTGAAACAGCAATGCCT

GAACAAGCAGTAGACATTGCCGTTAGCGTTGGTGGCTTCAAAGTGGGTGACCAAAACTACCCAATCAACC

CGAAAGTCATCTTCACTAACAACACAGGTACTGACTTACCTGGTGGCACGGAATTCCAATTCGATATTCC

AACCTCAGCACCAGATAACGCGAAGGACCAAAGTGGTGGTGGCTTGAAAGTGATTGCTTCTGGTCACACG

AGAGCTGACAACATCGGCGGCTTGGAAGGTGTTATGCACCGTGCAGCTTTCTCGCTACCGGCTTGGAAAT

CACTACCAGCAGGCGAAAGCTATGAATTGGATATGGTTTACTACCTCCCAATCTCAGGCCCAGCGAACTA

CACAGTTAACATTAACGGCGTAGATTATGCGTTCGCGTTTGAGTACAGCGATTTGCCACTGGCTGACCTG

TCAACACCGACAGATCCAGGTAACCCTGACAATCCAAACCCAGGTACCTGTGACACCACTGGCTTGAATG

TATATCCAAACTGGCCACAAGCCGATTGGCAAGGCACCCCTACCCACGCGGGTACAGGTGACCAAATCGT

TCATGAAGGCAAAGTGTACAAAGCGAACTGGTGGACATCGTCTGTTCCAGGAAGCGATGGCAGCTGGTCT

GTCGTGTGTGGCTAA

>NZ_LN554846.1:c1141870-1138703 Aliivibrio wodanis genome assembly AWOD1, chromosome : 1

ATGGAACATTCAAATTTAACAAAAAAACATAATTATAAATTTGCTCTCTCTACTCTCACACTCTCTTGTT

TAATGGCATTTAATGCACAAGCTTCGGTTAATTGTGACCCACTTCTAGCATGGGACTCAGGTACAACTTA

CAATGGTGGAGAAGAAGTAAAACAAGGCAATAATGCTTATAAGGCAAAGTATTGGACTCAAAATAATGAT

CCTGCAACCGCTGGGCAATGGGGAGCATGGCAAGATTTAGGTGTCTGTTCAGGAGAAGCGATTAACGTTG

CACCAAATGTTGATTTAACCTCTCCTAGCGTGACTGATAACATTACAACGGGAGATATTGTCACATTAAC

CGCTTCAGCGGCTGACAGTGATGGCTCTGTTGCTCTTGTTGAATTTTCCGTTGATGGCGTTATTATTTCT

TCAGTAACAACCTCCCCTTATAGTGCGATTTGGACAGCTACTGAGGGTAATCATACCTTTAGCGCTCAAT

CTTATGATGATAAAGGTGCTGTTAGTCTCATTAGTTCAGTCTCAGTTAATGTGGCAAACGTGCCTACTGA

TAATACCGCACCAACAGCGAGCCTTAGTTTATCAGCATCAAGTGTTGAACTTGGGGCAACGGTTACCTTA

ACAGCTGAAGCTTCCGATTCTGATGGCACTGTCGATAAAGTTGATTTCTATATAAATAATGCGCTTGTCG

GCACTGCAGCAACAGCTCCTTATGTTCTTCAATATAAAACTACAACTGCTGGTTCACTTTCTGTTTATGC

GAAAGCAACGGATAATTTAGGCGCAGTTGCCAACTCAGTGGCTGCCACATTAACCGTAACGAGCTCTTTT

CCTATTGCTGACAATTGTCGCCCTGATGGTTTATACCAAACAGAAGGCGTTAATGTTCCTTATTGTACAA

CTTATGATAAAGAAGGCCGTGAATTAATGGGAGCGGATCACCCTCGTCGTGTGATTGGCTACTTTACAAG

TTGGCGCGATGGTGGCGATGAACAAAACAGCTACCTAGTTAAAGATATCCCTTGGGAGCAATTAACACAC

ATCAACTATGCATTCGTAAGTATTGGCTCTGACGGAAAAGTGAATGTTGGTGATGTAAACGATCCAAATA

ATGCAGCAACAGGTAAAGAATGGGCAGGTGTAGAAATTGACCCAACTCTTGGCTTTAAAGGCCACTTTGG

CGCGTTAGCAACTGCAAAAGAGAAGCACGGCGTTAAAACATTAATTTCTATTGGTGGATGGGCTGAAACA

GGCGGCCACTTCGGCGCAGATGGTAATCGTGTTGCTGATGGTGGCTTTTATAGCATGACAACCAATGCCG

ATGGCAGCATCAACCACGCAGGTATTGAGAAATTTGCAGCATCTGCTGTTGAAATGATGCTTAAATACAA

ATTTGATGGTTTAGATATTGATTACGAATACCCGACTTCTATGGCTGGTGCTGGTAATCCAGACGATAAA

GCATTCATGGAACCTCGTCGTCAATATTTATGGGCATCATACCAAATATTAATGAAGACACTTCGTGAAA

AATTAGATGCTGTTTCAGCTCAAGATGGACAACATTATATGCTGACCATTGCTGCACCATCTTCAGGTTA

TTTATTACGTGGTATGGAAACCTTTGATGTTACTCAGTACCTAGATTACGTCAATATCATGTCTTACGAT

TTACACGGTGCATGGAATGATCACGTAGGCCACAACGCCGCATTATATGACACAGGTAAAGATTCAGAAC

TGGCTCAGTGGAATGTTTATGGCACAGCCGCTTATGGTGGTATTGGTTACTTAAATACCGATTGGGCTTA

CCATTATTTCCGTGGTTCTATGCCAGCTGGTCGTATCAATATCGGTGTTCCTTATTATACTCGTGGTTGG

CAAGGTGTGACAGGTGGTGATAATGGTCTATGGGGACGCGCACCATTACCTGATCAAACTAAATGTGACG

CAGGAACTGGCGAAGGCGAGAAAAATAACTGTGGTTACGGTGCTCTAGGTATCGATAACATGTGGCATGA

TAAAAACTCATACGGGCAAGAAATGGGCGCAGGCTCTAATCCTATGTGGCATGCTAAAAACCTTCAAGAA

GGCATCTTTGGCTCTTACGCAAATATTTACGGTTTAGATCCTGTAAATGACCCTGCTGACCAATTAGTAG

GGACTTATACTCGTCATTACGATAATGTCGCAGTTGCTCCTTGGCTATGGAATGCAGAGAAAAAAGTCTT

CTTATCAACAGAAGATAAAGCTTCAATTAATGTAAAAGCTGACTATGTCATCGATAAAGAAATCGGTGGT

ATCATGTTCTGGGAATTAGCAGGCGATTATAGCTGTTATGTTCTTGATGCTAAGGGGAAACGTACCTCTG

TAGATGCTACTGAAGCTGCTTGTCAAACAGGAAATGGTGAGTACCATATGGGTAATACCATGACTAAAGC

TATTTACGAGAAGTTTAAGTCTGCAACTCCATACGGCAATACTGTCGCTACAGGAGCTATTCCAACGGAA

ACCGTTAATATTGCAGTGAATGTCGGCGGATTTAAGGTTGGTGACAAAAACTACCCAATTAATCCGAAAG

TTACATTCACCAATAATACAGGCCAAGATCTTCCTGGTGGAACAGAGTTCCAATTTGATATTCCAGTATC

AGCACCTGATAATGCGAAAGATCAATCTGGCGGTGGTTTACAAGTCATCGCTTCTGGTCACACTCGTGCA

AACAACATTGGTGGTTTAGACGGCCCAATGCACCGTGTTGCATTTACTCTACCAACATGGAAAGCGCTTC

CTGCAGGTGCTGTTTATGAACTAGATATGGTGTATTACCTACCAATTTCTGGTCCTGCAAATTACTCTGT

TAAAATAAATAACACAGAGTATGCGTTTAGCTTTGAACAACCTGATTTACCTTTAGCTGATTTATCAGCT

GGTGGTAACCCGGGCGGTGGTGATAATGGTGGAAATCCTGACCCAGGTTGTGATGCCGCAGGTGTAGTTA

CTTATCCGGATCTTCCACAAAAAGATTGGGCAGGCAATCCTAGCCATGCGAATACCGGAGATAAAGTTAT

TCACAACAATGTAATTTATCAGGCAAACTGGTGGACAGCAGCGGTACCGGGAAGTGATGGTAGCTGGACT

AAAGTATGTAACCTTTAA

>NZ_FLOD01000003.1:c389471-386367 Vibrio toranzoniae strain CECT 7225, whole genome shotgun sequence

ATGCACCTAAACCAAGGAAGAGTGGCCAAGAAGGTTTTTACACTCAGTACTCTCACCGCGTCCTGCTTAA

TGGCGTTCAACAGCTATGCAGCTGTTGATTGTTCAACCTTAGACACGTGGGACTCTGCCACTGTATATAC

CGGTGGTGATCAGGTTTCACATGACGGCAGTGCTTATACAGCAAATTACTGGAATCAGAATAACAACCCA

AGCCAGTTTGAAGGCGACTATGCACAATGGAAGAAAGTCGATGTGTGTAACGGAGACGGAGGTGGCGGTA

CACCAAATGTAGCACCAACGGCTTCGTTAACAGCGCCAATGGCATCAGATGTGATTGTTGAAGGCGACAA

CGTCGTATTAAGCGCAACAGCACTAGACACTGATGGCAGTGTTGCTTCCGTAGAATTCTTTGTTGATGGG

TCTTCGGTCGCGGTGGTGACAGCGGCACCATTTGAAGCGGCTTGGGTTGCAACGTCTGGTAACCATCAGG

TTTCAGTTGTCGCAACGGACAATGAAGCGCCGACAGTTTCAGTTGCACTTTCAGCGACATCTGTTGATGT

TGGTGGTGTAGTCACACTGACAGCAACGGCAGCAGACAGCGATGGTACAGTTGATAAGGTTGATTTCTAT

GTAGCGGGTGCTCTTGTGGGTACAGCAGCGACAAGTCCATACACGCTAGATCACACAACCACTCAAGCAG

GCTCTCTAGCGGTTTACGCGAAAGCGACAGACAACCAAGGTGCAACGACTGATTCAGCACTGGCTTCTTT

GACTGTGAATGGGGCTCCAACAGTGAGCACTTGTCGTCCTGATGGTTTGTACCAAACACAAGGTATCGAT

GTTCCCTACTGTACGATTTACGATGAAGATGGCCGTGAGAAGATGGGCGCAGATCACCCACGTCGGGTGA

TTGGTTACTTCACAAGCTGGCGTGCAGGAGACGACCCACAAGCCGCTTACTTAGTAAATGACATTCCTTG

GGAACAACTCACACATATTAACTATGCTTTCGTGAGTATCGGTTCAGACGGCAAAGTCAATGTGGGTGAT

GTAAACGATCCAAATAACGCAGCGGTTGGTAAAGAATGGCCGGGCGTGGAAGTTGATCCTGAATTAGGCT

TCAAAGGTCATTTTGGTGCTTTAGCGACAGCGAAGAAAAAACACGGTGTTAAAACACTAATTTCTATTGG

TGGTTGGGCTGAAACCGGTGGTCACTTCGCGACTGACGGAAGCCGAGTGGCTGATGGTGGTTTCTATACC

ATGACCACCAATGCCGATGGTTCAATTAACCATCAAGGCATCGAAACATTCGCAACTTCAGCGGTTGAAA

TGCTTCGTAAATACCAGTTCGATGGTCTAGATATCGATTACGAATACCCAACCTCAATGGCGGGCGCAGG

TAACCCGTACGACAAAGATTTCATGGAGCCACGTCGTCAGCATTTATGGGCTTCATACCAAGTGTTAATG

GAAGTGCTACGTGAGAAGTTAGATGCGGCGTCTGCACAAGATGGTACACATTACATGTTAACTATCGCGG

CTCCTTCTTCGGGTTATTTATTGCGTGGCATGGAAACATTCGATGTGACCAAGTACCTCGATTACGTGAA

TATCATGTCTTACGATCTTCATGGTGCGTGGAACGATCATGTTGGTCATAACGCTGCCTTGTTTGATACA

GGTAAAGATTCAGAGTTAGCACAGTGGAACGTTTACGACACTGCCGCTTACGGCGGTATCGGTTATCTGA

ACACGGATTGGGCTTACCATTACTTCCGCGGTTCAATGCCAGCCGGTCGTATTAACATCGGTGTGCCTTA

CTACACTCGTGGTTGGCAAGGTGTGACCGGTGGTGAGAATGGCCTTTGGGGCCGAGCTGCACTACCAAAT

CAATCTGAATGTGCGGCGGGTACAGGCGAAGGCGAGAAGAACAACTGTGGTCATGGCGCAATTGGTATCG

ACAACATGTGGCATGATACCGACCCGAAAGGCAACGAAATGGGCGCGGGTTCTAACCCAATGTGGCACGC

TAAGAACCTAGAGAAAGGTATTTGGGGTTCTTATGCGGATGCTTACAAGCTTGATCCTGTCAACGATCCA

TCTGATGTTCTAACGGGTACTTACACGCGTAACTACGACAGCGTGGCGGTTGCACCTTGGCTGTGGAATG

CAGAGAAGGGCGTATTCCTTTCTACAGAAGATAAGCAGTCTATCGATGTGAAAGCGGACTACGTTATCGA

TAAAGAAATCGGTGGCATTATGTTCTGGGAGCTAGCAGGGGATTATAACTGTTATGTACTCGATGCGAGT

GGCAACCGAACTTCAATCGATACCACTGAGCAAGCATGTAACAGCGGTAACGGTGAGTTCCACATGGGTA

ACACAATGACGAAAGCTATCTACGATAAGTTTAAGTCTGCAACCCCATATGGAAACAAAGTCGCGACGGG

TGCTATCCCAACAGAAGCGCTAGATATCGCGGTTTCAGTTGGTGGCTTCAAAGTCGGTGACCAAAACTAC

CCAATCAACCCTAAGATCACGTTCACGAACAATACGGGGCAAGCACTTCCGGGGGGCACCGAATTCCAAT

TCGATATCCCAGTATCAGCACCTGATAACGCGAAAGATCAATCGGGCGGTGGTTTGTCAGTGATTGCATC

TGGTCATACTCGTGCGGATAACATTGGCGGACTAGACGGACCCATGCACCGCGTAGCGTTTACGTTACCC

GCGTGGAAAGATCTTCCTGCTGGTGGTGTGTATGAGCTAGATATGGTGTATTACTTGCCAATTTCAGGTC

CTGCGAACTATGCAGTGAGTGTGAATGGTGTCGACTATGCCTTTAGCTTTGAGCAACCAGATTTACCACT

AGGTGATATCAGTTCTGGTGGCGGTAACCCTGGCGATGGTGGTACAAACCCTGGTACTTGTGACACTACA

GGTTTAGCGATTTACCCTGATTTACCTCAAAAAGACTGGGCGGGTAACCCAAGCCATGCAAACACGGGTG

ACCAAGTTGTTTATAACAACGTAGTATACCAAGCGAACTGGTGGACAAGTGCCGAACCGGGTAGTGATGG

TAGTTGGACGAAAGTATGTTCTTAA

>NC_011744.2:616053-619211 Vibrio splendidus LGP32 chromosome 2

ATGCACCTAAATCAAGGAAGAGTGGCCAAAAAGGTTTTTACACTCAGTACTCTCACTGCGTCATGCTTAA

TGGCTTTCAATAGCTATGCGGCTGTGGATTGCTCAACGTTAGAAGCGTGGGATCCTGCCACGGTTTACAC

CGGCGGCGATCAAGTTTCACATGACGGTAGTGCTTACAAAGCCAATTATTGGAATCAAAATAACAACCCA

AACCAATTCGAAGGTGACCATGCACAATGGAAGAAGGTTGATGTGTGTAGCGGTGGTGGTACGCCCAATG

AAGTGCCAACGGCTTCGTTAACGACTCCATCAGCCTCTGACATAATCACTGAAGGCGATAACGTGGTGTT

GAGTGCAACGGCGCTAGATTCTGACGGCAGTGTTACCTCGGTTGAATTCTTCATTGATGGAACTTCAGTT

GCGGTTGTAACAGCTGCACCTTTCGAAGCGGCTTGGGCTGCAACGTCTGGTAGCCATCAGGTTTCGGTTG

TTGCTACAGACAATGAAGGCGCGGCTAGCTTAGCGAGCGCGGTCTCTATTTCTGTCGATTCAGTTACACC

TGGAAATGAAGCGCCGACAGTATCGGTTGCGCTTTCTGCATCTTCGGTTGATGTTGGTGGTGTGGTAACG

CTTACCGCAACGGCAACAGACAGCGATGGTACGGTTGATAAGGTCGATTTTTATGTGGCAGGTTCTTTAG

TCGGAACGGCTGCGACAAGCCCATACACGCTTAACTACACAACCACTCAAGCGGGTTCTTTAGCGGTTTA

CGCAAAAGCGACTGACAATCAAGGCGCAACGACAGATTCAGCGCTGGCTTCTTTGACGGTGAATGGCGTA

CCAACGGTGAGCACTTGTCGACCTGATGGCTTGTATCAAACTCAAGGTGTCGATGTTCCTTATTGTACGA

TTTACGATGATGAAGGCCGTGAGAAAATGGGCGCGGATCACCCACGTCGTGTCATTGGTTACTTCACCAG

CTGGCGTGCAGGGGATGACCCACAAGCTGCTTACCTAGTAAATGACATTCCTTGGGAACAACTCACACAC

ATTAACTACGCTTTCGTGAGCATTGGCTCAGATGGCAAAGTAAACGTGGGTGATGTGAACGATCCAAACA

ACGCAGCGGTTGGTAAAGAGTGGCCGGGCGTGGAAGTTGACCCAGCATTAGGCTTCAAAGGTCACTTCGG

TGCATTAGCGACAGCGAAGAAAAAACACGATGTTAAGACTTTAATTTCAATCGGTGGTTGGGCGGAGACC

GGTGGCCACTTTGCCACTGATGGTAGCCGAGTGGCTGATGGTGGTTTCTATACCATGACGACCAATGCTG

ACGGTTCTATTAACCATCAAGGTATCGAAACATTCGCAACTTCAGCGGTTGAAATGCTTCGTAAATACAA

GTTCGATGGTCTAGATATCGATTACGAATACCCAACGTCAATGGCGGGCGCAGGCAATCCGTACGATAAA

GACTTTATGGAACCACGTCGTCAATACCTATGGGCTTCTTACCAAGTGTTGATGAAAGTGTTGCGTGAGA

AGCTTGATGCAGCCTCTGCGCAAGACGGCAATCACTACATGTTAACTATCGCGGCGCCTTCTTCGGGTTA

CCTACTGCGCGGTATGGAAACATTCGATGTAACCAAATATCTCGATTACGTAAACATCATGTCTTATGAC

CTTCACGGTGCGTGGAACGATCACGTAGGTCATAACGCTGCGTTGTTTGATACAGGTAAAGATTCAGAGT

TAGCACAGTGGAACGTATACGGCACAGCGGCTTACGGCGGTATCGGTTATCTGAATACGGATTGGGCCTA

CCACTACTTCCGTGGTTCTATGCCAGCAGGTCGTATTAACATCGGTGTGCCTTACTACACACGTGGTTGG

CAAGGTGTAACTGGTGGTGAGAATGGTCTTTGGGGCCGAGCACCACTGCCAAACCAAGCTGAATGCTCAG

CAGGTACTGGTGAAGGCGAGAAGAACAACTGTGGCCATGGCGCAATTGGTATCGATAACATGTGGCACGA

TACTGATCCGAAAGGCAACGAAATGGGTGCAGGTTCAAACCCAATGTGGCATGCGAAGAACTTAGAGAAA

GGCATTTGGGGTTCTTATGCAGCAGCTTACAAGCTTGATCCTGTTAACGATCCTTCAGATGTTCTAATGG

GGACTTACACGCGTAACTATGACAGCGTGGCTGTTGCTCCTTGGTTGTGGAACGCAGAGAAGGGCGTATT

CCTTTCAACGGAAGATAAAGATTCTATCGATGTGAAAGCCGATTACGTTATCGACAAAGAAATCGGTGGC

ATCATGTTCTGGGAACTAGCAGGGGATTACAACTGTTACGTACTCGATGCGAACGGTAACCGAACTTCAA

TCGATACAACCGAACAGGCGTGTAATAGCGGAAACGGTGAGTTCCATATGGGTAACACTATGACGAAAGC

TATCTACGATAAGTTTAAGTCAGCTACTCCATATGGAAACAAGGTTGCAACGGGTGCTATCCCAACGGAA

GCATTAGATATTACTGTTTCTGTAGGTGGCTTCAAAGTCGGTGATCAAAACTATCCAATTAACCCTAAGA

TCACGTTTACAAACAATACAGGTCAAGCGCTTCCTGGTGGTACTGAGTTCCAGTTCGATATCCCAGTGTC

TGCACCTGATAACGCAAAAGATCAATCGGGCGGTGGTTTGTCTGTGATTGCTTCTGGTCACACTCGTGCC

AATAATATTGGTGGTCTAGATGGTCCGATGCACAGAGTAGCGTTTACATTACCTGCATGGAAAGAGCTTC

CTGCTGGTGGTGTGTATGAACTCGATATGGTTTATTACTTGCCAATTTCTGGCCCTGCAAACTATACAGT

GAATGTGAATAGTGTCGATTATGCATTTAGCTTTGAGCAGCCGGATCTACCACTGGGTGATATCAGTACT

GGTGGCGGTAATCCTGGTGATGGCGGTACCAACCCAGGTACATGCGATACTGCGGGTTTAGCTGTATATC

CCGACTTACCTCAGAAAGATTGGGCGGGTAACCCAAGCCACGCAAACACGGGCGACCAAGTGGTTCATAA

CGGTAGTGTTTACCAAGCAAACTGGTGGACAAGTGCCGAGCCGGGAAGTGACGGCAGTTGGACCAAAGTG

TGCTCTTAA

>fig|6666666.586707.peg.4039 Chitinase (EC 3.2.1.14) [Cytophagales sp. Alg240_R148]

atgtacacttctacaaatgcaagacttcatcgtcttttaagtgtcggttttattaggaaa

tcgattttgctgctgttcatgtttttcattgtattaactgggttcgctcaagttaatact

ggtggaaccaagaacaagtctgatcataacaaacaaatgatcggttatattaccaactgg

gacgcttggaaagacaccaaagcgggggttccagcacaaggtgccttgacccacctcaat

attgattattcaaaatacactattttgaactattctttttttggggtagccaaagatggt

tctctacacagcggagaccacagaaacaaacaaatttacaaagaaggcgttagccaagaa

cccgcagacatattttttacggatgtttatagcagttgggatttgcatattctttttggg

gaattggaaaatgtcaactacatcaatgaagacatcaaattgagggcagaagcccagggg

tttgaggttacagttggcgctagcacctggacccatccagggtgggggctttccggaccg

cttccattgcctctgaaaaaagaaggcggggctccaggattattggacttggcccatcaa

aataatgtaaaggtgatggcttctttaggtggatggtctatgtgcaaacatttccctgaa

atggctgcagaccctgtgaaaagggcgaaattcatagcagattgtaaaaaactaattgac

tctggttttgatggtattgatttggactgggaatacccaggcccttatgcaggaatgaat

ttcaccggatcagaagctgattttcaaaatttcaccaccctagtacaagaaatacgtacg

gccattggcccagacaaattaattacagccgctttttctgcttcttcacagaaaattcaa

ggattggaatggggccaattgagccaaaccatggattatttcaatttcatgacgtatgac

tttaatggtggttggtccaatattgctgggcacaatgcaccgctttacccttatgatggt

gccgaagcacctgaattcaactggaaaagtactttggatgctttaaaattactgggcgtc

ccaatgaataagatttgttttggtttgcctttctacggaagaggtgttgtaacccaagga

gctgccgctctaaatggtgccaccaacaaaagacaagagaccgtgcagcccgatgggcca

ataacaacttgtgcagactatacgaattggcctaaggaggtttacgatggcacaccaaat

tattttttcattaaacaaaaggcacttggggcaaacagtggttggacccgccattgggac

gaccaggctaaggtgccctacctgaccaatggcaattactttttatcgtatgatgacgaa

gagtcgatcgaacacaaagccaattttattaacgacaacggtttggctggagctattgtt

tggacagtttatggagatttggagttttctggatctgcgactaattttggtaccaaactt

aaacggtggtcttctgttgattctaaattaatcaataagaccaatgaagtgtttgcacaa

gggagtgttggattgccacaagttacacttaccgcaccggtccataatgcacaattcaac

ccaggcagtgatataacattaaccgccaccgcgtccgatgccaatggcagtattaccaaa

gtggagttttttaatgggagtttgaagttaggagaagatgcaaccgctccttatagcttt

gtctggccggctgttgcagaaggaagttatgcgctttcggcgaaagctacagataatgaa

aacaatgtggccaattccaatgtttccaacatcactgtgggtactgatgtacttcctact

gtttcaataaccagccctgccaatggtgcaacttttgatgaaggtgccgttattgcaata

acagccgaaggtacggcgactggggcctcgatttctaaaattggtttttatcaaggcaat

acactattgggagaagatacttccgcaccctatgcgtatgcatggaatacagcattggct

ggaaattacgacctgaccgcagttgtaacggatagtaagggccaaacagcaacttcagct

gtagtggctgtggtggtcagtccggcatcaggaggatgtgatggaatagaaccttgggat

ccagaaaaagtgtatgctgctggcggaaccatagtgagccataaaggtagaaaatatcaa

aatggttggtggacacaaggagaagaaccgggcactacaggagagtggggtgtttggaca

gacatcgggccttgcccagggggtggcaacataccaccaacagttagcattagtagccca

agtcaaaaccaaaattttgacttaggaacggcggtacctattgctgtaaatgcatcggat

tcagatggaactgtaacctcggttgcagtgtacgaaggaaacaatctattggctactttg

actgctgccccttacagttatgtatggaacaatgcaacagttggcaactatagtttaacc

gcagttgccacagacgacaaaaatgctactacaacttcaatggctgttgttattgccata

gtggatggaagcaatgcggcacctgtagtaaccttaaccagcccttccaataatgctcaa

tttacagaaggagatgttatcaatttaagcgccacggcaactgatacagatggaagtata

agttcggttgatttttatcaaggatctgtgttgctgggaacagacaatgcagcgccgtac

gaatggacttggagcaatgcagccataggaacttatgcgcttacagcagtagcaacagac

aatgctggagcaacaggaacatcctcagtaaccaacatggaggtgaaagaggctactact

ggtggatgtacttcgcctcaatacgttgcaggaactacctatgccactggtgcggatgtt

caaaacaatggaaaggaatacaactgtaaagaagggggctggtgttcttctagcgcggct

tgggcctacgaacctggtgcgggtatgtattggcaagatgcctggactttggttggagat

tgcggaccaggaggagatccaaaattacctagtgttgcgatcacatcgccaagcgaaggc

caagtgttcaatgcaggtcagaacattttaattactgccgatgcttcggatgcagatgga

agcattacaaaggtggtgttttatgatggttcggtgttgttaggagaagatttaaccaat

ccttacagccataccattgccaacccatcggttggagtacatgctttgaccgcagtcgca

tttgacaatgaaaatcaatccactacttccagtgttgtaaacgcatcggtcagttccagc

cctcctggtaatcctgggcttccaggtaaaattttggtaggatactggcacaattggcaa

aacaacagtgccccctacattcgattgaatcaagtgtcggataattacgatgttgtgaac

gtttcatttgcagtgcctaggtccttttcagatatgaccatggtattcaacccagaagaa

gtaacggtagctacctttaaatcggatatggcagcattaaaatccaaagggaaaaaagtt

ttgatctctattggtggagccaatgatccggtagaattaaagacaactgccgatcgagat

aaatttgtgagttccatgattggcataattacagaatacggattcgatggttttgacata

gatttagaaggaagttctgttagtttggatcctggcgatacggattttaagaatccaaca

acagttaagatcaacaacctaataacggctacaagagaaatagtaaaccattttggttca

gattttatacttactatggctcctgaaaccttttatgttcaagctgggtttgactcctac

ggcaatggggcaggggcgtatttaccagtaatttatgcgctcagagatagaatgacttat

atccacgtacaacattacaatacaggtagtatgtatggggctgataataaaatttaccag

cctgctacagctgatttccatgtggccatggccgatatgcttttaactggttttccaatt

gcacggaatgaaaacaatcggttccctgcattgcgagcggatcaggtagcttttggtgtt

ccagcttctactggagctgcatcttccggttatacctcgaatgcagatgttcaaaaagcc

atgaattacttaatcaatggcgtttcatttggcggtacctatgtgttaagcaatgcttca

gggtatgcaaactttagaggattgatgacgtggtcgatcaattgggacgctgctactggg

ttgagtttttcaaatgcacatgccgcatttttacatggcgggtctgctgcaggtgttaca

agcagtttggcaacaaatgtttctacaccgcttccatctgtcaacaactatttggtttac

cctaatccaatgagcggagaagtttccattgattttgaactgtcctacgaaggtaaaact

gaaataattgtaatgaaccaactcggtgaagtagttcaaaatgtttcttcaggttatctg

cgcaaaggaaggcataagttttttgtcaattcgcaacaattccctcaagggatctatttg

tttagaattaccacggaccaatctacggaaacggtaaaaatgcttaaacactaa

>fig|6666666.586707.peg.1830 Chitinase (EC 3.2.1.14) [Cytophagales sp. Alg240_R148]

atgaaaccccattttatattgttggtatcttgcatgttattggcaagctgtcaacctagt

gcccagtctgaaacagatgcggccgtccaaaaaaagtttaaagttattgcctacctgttt

ggcaattcgaccaatcttggttctgctgaagtggcttatactggattaacccacctcaac

tactcctttgccaatattgtggatggcaaagctgttattccttccatacaagatagtttg

aaccttttaaagcttcaaaagttaaaatatgagcagcctacgcttaaaatattggtggcc

atcggtggttggacttggtctggtcatttttcagatgtagcctcgagcaaaacatccaga

gctaaatttgtagcttctgctattaaattggtggtggattacgacttggatggaatcgac

atagattgggaatacccgaatttacctggtatcgggaacaaacacgccaaggaagatgtg

aaaaactacacacttttgatggctgcgctaaggaacgcattgaacgagctgtcagaaact

accaatagggaatatgaattgtctgtggccactggtgcaggaaagtcctttttggacaat

acagaaatgaaagaagttcaaaagtatgtagaccatgtcaatataatgacttacgacaac

tatggtgagtgggatccccatacagggcataattctgcactgtatgcttccgatttcgac

caagacaaaaagattagtacggataaatccattaaaatgtacttaaaagcaggggtgcct

gctcaaaaaatcatccttggtttggcattttatgggaaagcttggaaaggtgtaaaccct

tccaataatgggttgtttcagccggcaaaaggtgcttatagaaaacacaggtcttcagaa

gtagcaagcaatccttcagcctatggttttaagtcgatgtatgacagtagcgcccatgcg

ccgtttttatggaatgaagaggaatcggtttttatttcttatgacaacctggcttcttat

cgagaaaaaatggagtacgtaaagtccaatcaattgggaggtaccatgttttgggaatta

aaagcagataaagaggggcagatcgtgtctttgatcaacaatgtacttggaagtaaagaa

taa

>fig|6666666.586710.peg.105 Chitinase (EC 3.2.1.14) [Enterovibrio sp. Alg239-V16]

atgaacaaacgtgtatttcttagaggactagcctctgcatcaattgcgagcgccttatcg

ctgcccgcaatcgctgcgcctggtgcgcctcagatttcttggatggaatcgaacttctcc

atcatcgaagtgaacgacgctgcctctgcatacaaagatcttgttactgtaaaacctttt

gcagaagtgcctgtcacttgggaccgttggtcaggcgagcctggcgatacgtggcgtgta

ttattgaacggcgtcgttgcgcatgaagccagcgtgacgccatccgccagccaaaaagaa

tcaactgttctgcaagttgcaaaaggcggcaagtacgacatggttgtcgaactttgtagc

ggcactggcgcagcacaggcttgtacaccaagtgcgccaaaacaaattcttgttgcagac

actgatggtagccacctcgaaccactcgccatgaacgtcgatccgaacaacggcagctat

gtaacacccgctgataccgtcgttggtgcgtactttgtggaatggggtgtttatggcaga

aaattccctgttgataaaatcccagcgcaaaacctgacacacatcatttatggctttgtg

cctatttgtggcaacaacccttctttagaagacggccctcttgctgcattgaaccgtgct

tgtgcaggcatgcctgactatgaagtggtcatccatgacccgtgggcagcagttcaaatg

cctcacccacaatcaggtcaaagccactcctcttcttacaaaggaacttatggtcagatc

atggcgcttaaacaacgctaccctgatctaaaaatcctcccttctatcggtggctggacg

ctttctgaccctttctatgattttggcgtaaaagcgaaccgcgatgttttcgtagcctct

gtgaaaaacttcctgcaaacgtggaaattctatgacggtgtagacattgattgggaatac

ccaggcggtcagggcgcaaaccctaaccttggcgacccaagcaaagatggcgatacctat

gcaatcttaatggctgaactgcgtaccatgttggacggactttctgctgaaacaggccgt

acttacgaactgacatcggcagtaggtgtgggctacgacaagattgaagacgttaactac

gccgacgctgtgcctcatatggattacatcttcgcgatgacctatgactactatggcgga

tggaataacgttgtagggcaccaagccgcgcttaattgtggtagccacatgtctgcggac

gaatgtgcgggtaaaggcctagatgacgaagggaaaccacgtaaaggacctgcgtacacc

actgctaacggtatcgacctgttaatggcaaaaggtgttcctgcgaataagttggtcgtg

ggtgccgccatgtacggtcgtggctggacaggcgtaacagaagcaagcatgtctgatccc

actaacccaatgacaggcgtcggcaatggcaagattgcaggttcttgggaagcgggtgtc

atcgactacaaagacgttgttacccaatatgaaaataaagcaggcgttgttgtcggttat

gacgaaatcgctgaagcgccttgggcatgggatccgtcaaacggtgacctagtcacttat

gacaacaagcgttctgtgatggcgaaaggtgcttatgtgcgctctctcggtctcgcgggt

ttatttgcttgggaaatcgatgcagataacggcgatatcctaaacgcgatgcaagagtct

cttgccggtgatgttcctccacctaccaacaaagcacccgtcgcaaaagcgggcgcagat

gtggttgttgaggctgctggcgcagtcgcactagacggaagtaactcgtctgatgccgac

ggtcaggttgtttcatacagctgggcacaaacatcgggtccaagtgtgtcactaaccaat

gcgaacgcatcagtcgcttctgcagacattccatcggtcaccgttgatacgcagtttgtt

ttcacgctgaccgtgactgacaacaaaggcgcaacagcgtcagacgttgtgacagtaacc

gcgaagaaacaaggtggcactgatccagttaacactgcacctgttgctgtggtttcggcc

cctagcgcagtaaaagctggcgatgttgtccttgttgatgcaagtgcttcaaccgatgct

gaaaacgataccctaacgtatagctggtacatcccagaaggcgtaaatgcgacggttaac

ggtgcgtctcttacttttgttgcagatagctacgcaacaacgaagaacttcaccttcgca

gtcaccgtgtctgatggcaaactggacgacagtgcctctatcacagttaccgttgcgaaa

aataccactgaccctgtgtgtgctaatgcttggaaagaaggtgttgtatataactctggt

gatgtcgtgactcacaacggaaaagagtactcggcgaaatggtggacgcaaaacgaagag

ccaggcacaaccggtcaatggggtgtttggaaagagttaggtgctgctaactgtcaataa

>fig|6666666.586710.peg.605 Chitinase (EC 3.2.1.14) [Enterovibrio sp. Alg239-V16]

atgaaaactatcctcgcaggcgcggtattgagcgcgtgtatagcgacgccaattttcgcc

gaaaacaacatagtgggtgggtatttcgcagattggcaatatgccaaccttgataatccg

tataccgttgcggatattcccgcggataagatgacgcatgttatctacgcatttttaagt

atgtgtgggcctcacaatagtgcgtcagagttggttcaaaagcaggtagaaactgcgtgc

aaggggaaagcaccattctctgctgtggttgtcgatcaagaagccgcgttggacatcgat

tttggttcggtgtcggtggacgttccgtttaaaggccactttgcacagttagcgcagctt

aaaaaagacaacccagatatcaccattttaccgtccttcggcgggtggaccatgtctgaa

cctttccatgcaatggccaaagatgatgatgctattaagcacttcgctaaatctgcggtg

gcactcattgccgagtatgatttcttcggcggtattgatcttgactgggagtacccgggt

ggtggtggcttaacaacgtctccttggaaccctgaaactaagctaacagatgaacaaaaa

gcctctgagcgtgaagcattcaccacgttaatcacagcattgcgctcggagttagatgca

ttgaaagcagaaaccggccaagactatgaattgtcttctgctgttggcgttgggccaaaa

gcggcacaaatcgattggccgtctgttgcccctatgatggataacatgtttgccatgacc

tatgacttcttgggagggtggggaacccaaactggccatttgacgaacctccacgcaacc

gagcgaagctggtggggaatgggggcggatgtgtttgttaatcagatgattgaaaacggc

attccaaaagagaaattggtattgggtgcggcgttttacggccgtggttgggaaggctct

caatttgatggcagcttgcctacgagcgacttagcgtcggaaaagggggcgagctttgga

acgggtgagccgggttacttcatgtattgggatttgaaaaccaattataccgctgaacaa

ggttatcagtatggctacgatgaagaatcgcaagccccttatttgtggaactctgagaag

aaagtgtttatctcatttgaagatgtgcgttctgtgaaagccaaagctgaatgggcgaag

cagcaggggttagcgggtgtctttacgtgggagttgtcaggcgacccgactgacgaactc

acagacgctatgtatcaagtgttgcacagtaaaaccgccaagaaataa

>fig|6666666.586710.peg.888 Chitinase (EC 3.2.1.14) [Enterovibrio sp. Alg239-V16]

atgaataagaaacttaaggtctgcgctgtagcaacatgtgttgcgagtgcgctagcgatg

agcgcctccctacatgctgcgcctactcacgacaaagccgttatcggttatttgacacag

tgggaagcgtggaaaggcacggatgctggctttagcgtgaaaggcgaggccacacacctc

aacgtagatatggatatctactccattttgaacttcagcttctttggtgttgcgaaagat

ggctccatgcacagtggcgaccttcgtaacaaaaacatttaccaagatggatccgttcaa

gaacccggtccacttttgcacccggatgtttactctagctgggatttccacattctatgg

ggtgagctggaatatgttcacgaatacccaggaaacgagccgtggcaggctgatcaactc

gctaaagtgcaagcacaaggttttgtgaaagacggtcgcggttggaaacatgcaccatca

ggtgtgactggccaaatgccaatcccactgaagaaagaaggtggcgcgccgggcttgatt

gatttggcgaaccaaaaaggcgtgaaggtcatggcgtccattggtggctggagtatgtca

aagcacttccccgaaatggcggctaaccctgtgatgaaagaacgcttcctgaaagacgtc

gatcgtctgatggcattgggtttccacggtattgatattgactgggaattcccgggttct

ggcggcatgaacttcacgggtacggacgtggattacgccaactttgaacagttgatggat

gacattcgtgcgcgcatcggcgatgacaaattgctgacggctgcgtttaaagctgtacct

gcggcgcttgaaggctacgattggaatcgtctgtcgaattcgatggattactttaacatg

atgacttacgaccttaatggtggttggtcaaacgtcacgggtcataactcgccactttac

ccctacccagaagaagagtttgatggattaaccttggatgacttgcgcgtgtggatgcaa

gcacgtggtattccatcgaacaaaattaacttcggtgcggctttctacggtcgtggtgtt

caaacaaaagaaacaacggcttacttgggcgcgccaacggacaagcgcacggtgaacttc

tctgttgatggcccaactgaatcttctgtggatttagataactggaaagcgttcgaaggc

caaccaaactacaactacatcatcaagcaaaacggctgggaacatttctgggatgccaac

gcagaagtgccttatgcggtgaaaggaaagtacttcttgagctatgacgatgaagaggcc

attcgtaagaaagcggagtacgtcgtgaataacgaccttggcggtattatcgtttggcag

gtgcatggtgatattcagtgtgaaggcacctttatcaatcatggttccaagctgaaagaa

tgtaccaaccttaagtcaccattagcacaacaaattgatgatgtgttcagtgcaaatgtg

ttgccaaatgaagcaccggttatctcagtaccaggtgcgcaggcggcagattcaggtcaa

gtgattagctttgcagtatcagcgaaagatgcagacggcgatgcgttgtcgttctctgca

actggcgcggatgtgctcgataatggtgatggcactgcgaccgtcacttacaaagcaccg

aacaccgttacagaccttactgacaccatcactgtgacagtcactgacggtaaaaaatct

gacgttgcaagcgtgaccgtgaatgtgaaaggctcgggtgttgtagaaaacacatcacca

gtattgactgttcctgcctctgtttctgttaacgcgggcgagtctgtcgatatttcggtt

tcagcgtctgacgcagaaggcgatgcattgacctttacagcaagcgaaggtgtggttgct

caaaatggtgattctgcgattgttactgtaacagcaccagcatctgataaagacagcgta

atatcggttgtcgtgacggtgactgatggatcagacgcagactctgcgacagtgactgtt

aacgtgaaaggtgacactggcccgacggacagcacctgggatgctaataaggtctacaac

acgggtgacaaagtcattcataacggcgttgagttcactgcaaaatggtggaccaaaggt

gaagaacccggcaaagcgtctgtttgggcagagtttgacgatggctcactgaaagattgg

cgctctgacaaagtttacaacggtggcgatcaggtaatcttccaaggtgaaacctacaaa

gcgaaatggtggacaaaaggggacacgccaggatctgcaaatggcccttggcagctaatg

taa

>fig|6666666.586709.peg.741 Chitinase (EC 3.2.1.14) [Photobacterium sp. Alg240-V54]

atggaaaagggatttaataaaaccaaagtggcgggcattattgcaggctttctatttgca

agtacggcagctcaagtacaagcagcgccaacacatgataagaacgtaatcggttatcta

actcagtgggaagcatggaaaggtactaatgcaggtttttctgtaaaaggtgaagcgacg

catttgaacgtagatatggatatttactccattcttaatttctcattttttggcgtagca

aaagacggtacgttacatagcggtgatttacgtaataaacaaatttataagccaggtagt

gtgcaagagcctgggccattattacatccagatgtgtattcaagctgggatttccatatt

ctatggggtgaattagagtatattcatcaatatcctgtaaatgaaccgtgggatgctgaa

aatttagccaaagtaaaagcgcaaggttttgtgaaaagtggtaaaggttggcgtcataag

ccaactggtattgaaggtgatatgccgatcccacttaaaaaagaaggtggcgctccgggt

ctgattgatcttgctaatcagaaaggcgttaaggtgatggcatcacttggtggttggagt

atgtctaagcactttcctgaaatggccgccgatcctgttaaaaaagcccgtttccttaaa

gatctcgataagttaatggcattaggtttccacggtattgatattgattgggaataccca

ggttttggcggtatgaactttgcgggtgatcctgctgattttgctaactttgaacagtta

atggaagatattcgtaagcgtattggtcctgataaactactgactgctgcattctctgca

tcaaccgctaaacttgaaggatttaattggccacgccttgttaaatcaatggattacttc

aacatgatgacatacgaccttaacggtgcgtggtcaaatgttacaggccataacgcgcca

ttgtatccatatcctgaagaagaatttaaaggtcttgatttagatacactacgtatttgg

atggcagaaaaaggcattccatcagaaaaaattaactttggtgctgcgttctatggtcga

ggtgtacaaaccacagaagctgaggcttatttaggtgcgccaaccgataagcgtatttat

aatatgtcggttgatgggccattactatcggcagttgatgttgataactggaaagaattt

gaaggttcaccaaactataactacattgttaaaacaaaaggttgggaacataagtgggat

gctagtgctgaagtcccttatgctgtgaaaggaaaatatttcttaagttatgatgatgta

ccttcgatgaagaagaaagcacagtacattgttgatcatgatttaggtggagtgatcgtg

tggcaagtgcatggcgatattaagtgtgaaggcacctttattaatcatggtaccaagcta

aaagagtgtactaagttaagctcaccattagcagaagcgattgatgatgtatttagtgct

gacaccacgccaaatgctgcgccagagttaaccgtaccaagtgctcaatcggcactggct

ggtgagacaattagtttcactgtttcggcaaccgatgctgatggcgatagcttaagcttt

agttctgctgatgcacaaattacagataatggcgacggtactgcaactgtcgtttatacc

gcacctaactcagcaacagatactgtgacatcaatagcgatcaaagtaactgatggtcgt

aaaacagcaactaaatctgtggttgttaatgtgaaagggtctggtgtcgttgaaaatacc

gcaccagaattgacagcgccagcaatggttgatgttgaaagtggtcagaatgtggttatt

acagtaacagcaaccgatgccgatggtgatgcactaacttactctgcatcaacaggtcag

gttacagcgactgcaacgggtgctgatgttactgtcactgcaccaacaacaacgacagat

acaacactcaatgttgtgattactgtgtctgatggtcaagccgcgactcaacaaacagtt

atggttaatgttaaggctgaaggtggagctgttggtgatacgtggaatgccgatactatc

tacaatagcggagacactgtagtttataacggcattacttatactgcgcaatggtggacg

aaaggtgaaactccgggaacatcatcggtatgggtagaagaagataccggtgaagttggt

ggttggtcagcatcgaaaacctataatggtggtgatgaagttatctttgacggtaataat

taccgcgcaaaatggtggacgaaaggtgataaaccgggtaatgcaaatggcccttgggaa

cgcatctaa

>fig|6666666.586709.peg.1777 Chitinase (EC 3.2.1.14) [Photobacterium sp. Alg240-V54]

atgaagccttttttaaccacaacacgtacactcacattatgctcttttttagcttttggg

tttgtctctcaaatgaactttagctatgcagcagatcatgaccaacctgctgttgtggct

gcctactaccctgattggaaagtctatacgcctaataatccttatcctgcgagtaaaatc

cctgcagagaaattaactcatcttatttatgcctttcttgccgtttgtggccctgttgat

tcatcaccgaataacatcaaaaaaataattgccactcaatgtgcgaataaacccacaggc

acagccattattttagatgaatatgcagctttgcatattaaactagcaggagagaccgca

actaacgttagttacaagggtaattttggtcaattaaaagcattatcggataaatacccc

catttaagtattctaccaagctttggtggatggacactttcagaaccttttcataccgtt

gctcttgatccaacctatcgccagacatttataaatactgcagctgagttaatcttaaaa

tacgatttctttgacggcattcaaattgattgggaatatccaggcgggcatggattatca

ggtaaaggtcaaacaaatgccaaactagagcgggaagcctacagtctgttaattaaagaa

ttaaggcttaaattagacacactagaacaacaaaataaccgcgattaccaactttcagct

gctatcaatgctggctctaaaacattaccaggtattaattggtcttatattaaccaatat

ttagaccaaatctatttaatgagctttgatttcttaggcaattggaatcatgtcgttggc

catcacagtaatttatatagtacgccaaacactcctaataatatttctgtcgaaaaccaa

gttaacatacttatcgataagaacatttctaaaaataaaatcattatcggcagccctttt

tatggtcgaggatggcaaggagtcaacacaatctcagccgatagacttgaaaagttaagt

agtaatggcggcttaaaaaaaggttctgacattcaagatcctggctattttaactacagt

gatatcagtaaatacttccttaataaccctaaattaggttatcgctactactacgacgaa

gctgcacaagcggctgtgctttataatcaaaaaaataaagaatatattagctttgaagat

aaacgttcccttaaagcaaaagctgattatgtaaaaaagcatggtcttggtggcgtgttt

gggtgggaaatcacctctgacgtgaatgatgaattaatttcagtgcttgatagcacctta

aacccttaa

**References:**

1. Keller-Costa T, Lago-Leston A, Saraiva JP, Toscan R, Silva SG, Gonçalves J, Cox CJ, Kyrpides NC, Nunes da Rocha U, Costa R: **Functional and taxonomic signatures of the octocoral microbiome in healthy and diseased tissue**. *Microbiome* 2020, **MBIO-D-19-01154 *in revision***.

2. Karimi E, Ramos M, Gonçalves JMS, Xavier JR, Reis MP, Costa R: **Comparative metagenomics reveals the distinctive adaptive features of the *Spongia officinalis* endosymbiotic consortium**. *Frontiers in Microbiology* 2017, **8 10.3389/fmicb.2017.02499**(2499).

3. Keller-Costa T, Eriksson D, Gonçalves JMS, Gomes NCM, Lago-Leston A, Costa R: **The gorgonian coral Eunicella labiata hosts a distinct prokaryotic consortium amenable to cultivation.** *FEMS Microbiology Ecolology* 2017, **93**(12):1–19.

4. Hsu S, Lockwood J: **Powdered chitin agar as a selective medium for enumeration of actinomycetes in water and soil**. *Appl Environ Microbiol* 1975, **29**(3):422-426.

5. Kuddus M, Ahmad I: **Isolation of novel chitinolytic bacteria and production optimization of extracellular chitinase**. *Journal of Genetic Engineering and Biotechnology* 2013, **11**(1):39-46.

6. Suzuki K, Taiyoji M, Sugawara N, Nikaidou N, Henrissat B, Watanabe T: **The third chitinase gene (chiC) of *Serratia marcescens* 2170 and the relationship of its product to other bacterial chitinases**. *Biochemical Journal* 1999, **343**(3):587-596.

7. Hobel CF, Marteinsson VT, Hreggvidsson GO, Kristjánsson JK: **Investigation of the microbial ecology of intertidal hot springs by using diversity analysis of 16S rRNA and chitinase genes**. *Appl Environ Microbiol* 2005, **71**(5):2771-2776.

8. Esteves AI, Hardoim CC, Xavier JR, Gonçalves JM, Costa R: **Molecular richness and biotechnological potential of bacteria cultured from *Irciniidae* sponges in the north-east Atlantic**. *FEMS microbiology ecology* 2013, **85**(3):519-536.

9. Svitil AL, Chadhain SMN, Moore JA, Kirchman DL: **Chitin Degradation Proteins Produced by the Marine Bacterium *Vibrio harveyi* Growing on Different Forms of Chitin**. *Applied and Environmental Microbiology* 1997, **63**(2):408-413.

10. Suginta W, Robertson PAW, Austin B, Fry SC, Fothergill-Gilmore LA: **Chitinases from *Vibrio*: activity screening and purification of chiA from *Vibrio carchariae***. *Journal of applied microbiology* 2000, **89**:96 - 84.

11. Mansson M, Gram L, Larsen TO: **Production of bioactive secondary metabolites by marine *Vibrionaceae***. *Marine drugs* 2011, **9**(9):1440-1468.

12. Yu T, Zhang Z, Fan X, Shi X, Zhang X-H: ***Aquimarina* megaterium sp. nov., isolated from seawater**. *International journal of systematic and evolutionary microbiology* 2014, **64**(1):122-127.

13. Xu T, Yu M, Lin H, Zhang Z, Liu J, Zhang X-H: **Genomic insight into *Aquimarina longa* SW024 T: its ultra-oligotrophic adapting mechanisms and biogeochemical functions**. *BMC genomics* 2015, **16**(1):772.

14. Zhao J-S, Manno D, Beaulieu C, Paquet L, Hawari J: ***Shewanella sediminis* sp. nov., a novel Na+-requiring and hexahydro-1, 3, 5-trinitro-1, 3, 5-triazine-degrading bacterium from marine sediment**. *International journal of systematic and evolutionary microbiology* 2005, **55**(4):1511-1520.

15. Baba A, Miyazaki M, Nagahama T, Nogi Y: ***Microbulbifer chitinilyticus* sp. nov. and *Microbulbifer okinawensis* sp. nov., chitin-degrading bacteria isolated from mangrove forests**. *International journal of systematic and evolutionary microbiology* 2011, **61**(9):2215-2220.

16. Techkarnjanaruk S, Goodman AE: **Multiple genes involved in chitin degradation from the marine bacterium *Pseudoalteromonas* sp. strain S91**. *Microbiology* 1999, **145**(4):925-934.

17. Foley MH, Cockburn DW, Koropatkin NM: **The Sus operon: a model system for starch uptake by the human gut Bacteroidetes**. *Cellular and Molecular Life Sciences* 2016, **73**(14):2603-2617.

18. Shipman JA, Berleman JE, Salyers AA: **Characterization of Four Outer Membrane Proteins Involved in Binding Starch to the Cell Surface of *Bacteroides thetaiotaomicron***. *Journal of Bacteriology* 2000, **182**(19):5365-5372.

19. Silva SG, Keller-Costa T, Bloom J, Costa R: **Comparative genomics reveals complex natural product biosynthesis potential and carbon metabolism across host-associated and free-living *Aquimarina* (*Bacteroidetes*) species**. *Environmental Microbiology* 2019, **21**:4002-4019.

20. Larsbrink J, Zhu Y, Kharade SS, Kwiatkowski KJ, Eijsink VG, Koropatkin NM, McBride MJ, Pope PB: **A polysaccharide utilization locus from *Flavobacterium johnsoniae* enables conversion of recalcitrant chitin**. *Biotechnology for biofuels* 2016, **9**:260.

21. Terahara T, Ikeda S, Noritake C, Minamisawa K, Ando K, Tsuneda S, Harayama S: **Molecular diversity of bacterial chitinases in arable soils and the effects of environmental factors on the chitinolytic bacterial community**. *Soil Biology and Biochemistry* 2009, **41**(3):473-480.

22. Kharade SS, McBride MJ: ***Flavobacterium johnsoniae* chitinase ChiA is required for chitin utilization and is secreted by the type IX secretion system**. *Journal of Bacteriology* 2014, **196**(5):961-970.

23. Lee C-N, Tseng T-T, Chang H-C, Lin J-W, Weng S-F: **Genomic sequence of temperate phage Smp131 of *Stenotrophomonas maltophilia* that has similar prophages in xanthomonads**. *BMC microbiology* 2014, **14**(1):17.

24. Dziewit L, Oscik K, Bartosik D, Radlinska M: **Molecular characterization of a novel temperate *Sinorhizobium* bacteriophage, ФLM21, encoding DNA methyltransferase with CcrM-like specificity**. *Journal of virology* 2014, **88**(22):13111-13124.

25. Cottrell MT, Wood DN, Yu L, Kirchman DL: **Selected chitinase genes in cultured and uncultured marine bacteria in the alpha- and gamma-subclasses of the proteobacteria**. *Applied and Environmental Microbiology* 2000, **66**(3):1195-1201.

26. Hunt DE, Gevers D, Vahora NM, Polz MF: **Conservation of the chitin utilization pathway in the *Vibrionaceae***. *Applied and environmental microbiology* 2008, **74**(1):44-51.

27. Jahn MT, Arkhipova K, Markert SM, Stigloher C, Lachnit T, Pita L, Kupczok A, Ribes M, Stengel ST, Rosenstiel P *et al*: **A phage protein aids bacterial smbionts in Eukaryote immune evasion**. *Cell Host Microbe* 2019, **26**(4):542-550.e545.
